# Bio-inspired lanthanum-*ortho*-quinone catalysis for aerobic alcohol oxidation: semi-quinone anionic radical as redox ligand

**DOI:** 10.1038/s41467-022-28102-4

**Published:** 2022-01-20

**Authors:** Ruipu Zhang, Runze Zhang, Ruijun Jian, Long Zhang, Ming-Tian Zhang, Yu Xia, Sanzhong Luo

**Affiliations:** 1 Center of Basic Molecular Science, Department of Chemistry, Tsinghua University, 100084 Beijing, China; 2 Key Laboratory for Molecular Recognition and Function, Institute of Chemistry, Chinese Academy of Sciences, 100190 Beijing, China; 3 MOE Key Laboratory of Bioorganic Phosphorus Chemistry & Chemical Biology, Department of Chemistry, Tsinghua University, 100084 Beijing, China

**Keywords:** Synthetic chemistry methodology, Homogeneous catalysis

## Abstract

Oxidation reactions are fundamental transformations in organic synthesis and chemical industry. With oxygen or air as terminal oxidant, aerobic oxidation catalysis provides the most sustainable and economic oxidation processes. Most aerobic oxidation catalysis employs redox metal as its active center. While nature provides non-redox metal strategy as in pyrroloquinoline quinone (PQQ)-dependent methanol dehydrogenases (MDH), such an effective chemical version is unknown. Inspired by the recently discovered rare earth metal-dependent enzyme Ln-MDH, here we show that an open-shell semi-quinone anionic radical species in complexing with lanthanum could serve as a very efficient aerobic oxidation catalyst under ambient conditions. In this catalyst, the lanthanum(III) ion serves only as a Lewis acid promoter and the redox process occurs exclusively on the semiquinone ligand. The catalysis is initiated by 1e^-^-reduction of lanthanum-activated *ortho*-quinone to a semiquinone-lanthanum complex La(SQ^-.^)_2_, which undergoes a coupled O-H/C-H (PCHT: proton coupled hydride transfer) dehydrogenation for aerobic oxidation of alcohols with up to 330 h^−1^ TOF.

## Abstract (web summary)

A decade ago the first rare-earth-metal dependent enzyme was discovered, in which a non-redox lanthanide ion is central in the active site of a methanol dehydrogenase. Inspired by this discovery, here the authors show that an open-shell semi-quinone anionic radical species, complexed with lanthanum, could serve as a very efficient aerobic oxidation catalyst under ambient conditions.

## Introduction

**Fig. 1:**
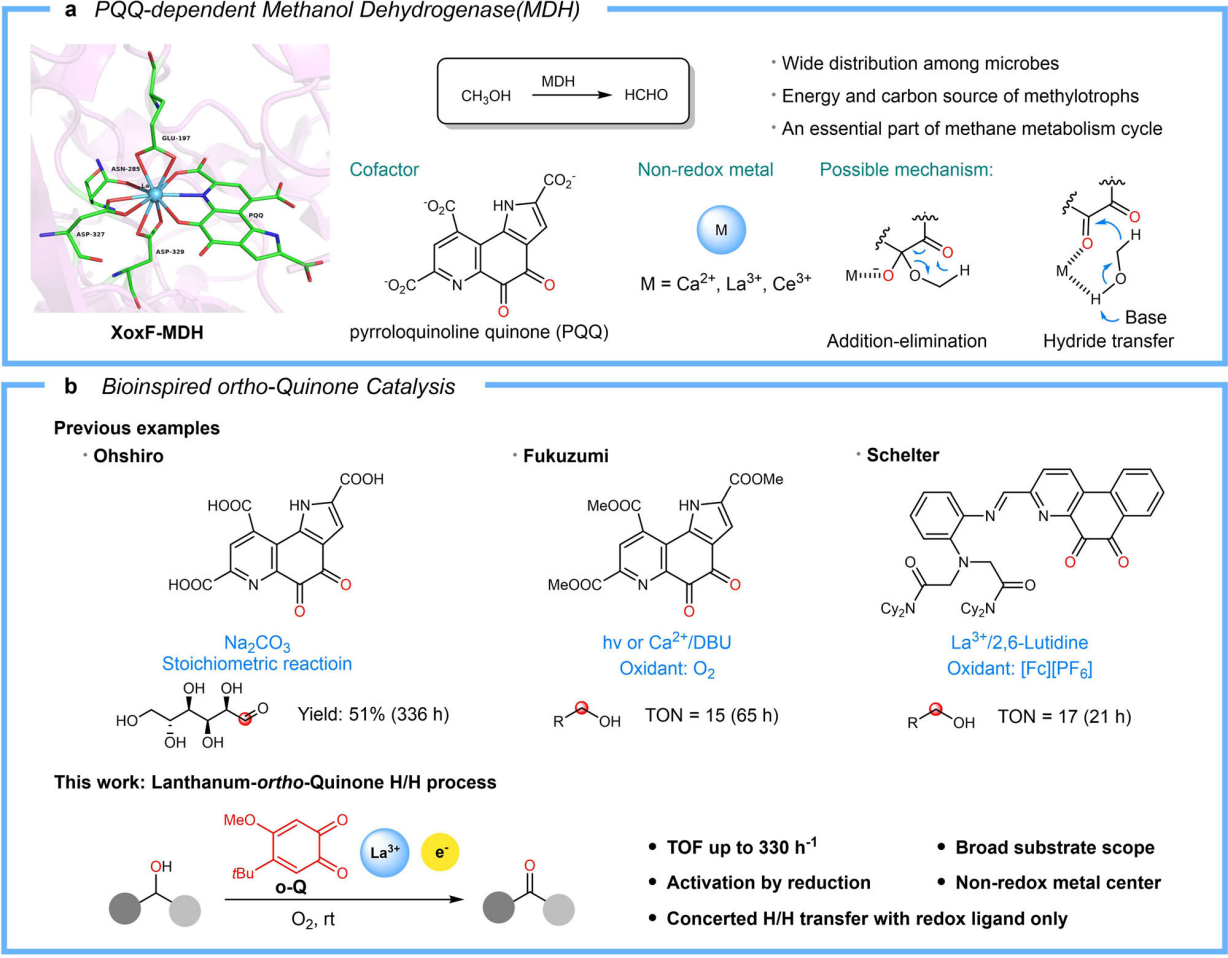
Aerobic oxidation of alcohols. **a** Left: active sites of XoxF-methanol dehydrogenase (La-MDH) (PDB ID: 6adm). Right: overview of methanol dehydrogenase. **b** Bio-inspired *ortho*-quinone catalysis. Upper: previous reports about redox properties of PQQ model compounds. Lower: overview of this work.

Rare-earth metals are in fact not rare but widely distributed in earth crust and lanthanides such as La and Ce are as abundant as Cu and Zn^1^. In the living sphere, nature has evolved effective strategies in utilizing earth crust metals as active centers in enzymes. However, a rare earth-metal dependent enzyme was not discovered till 2011, when the first lanthanide enzyme, Ln-MDH was discovered in methylotrophic bacterial by Kawai^2,3^. Ln-MDH is a lanthanide-dependent methanol dehydrogenase (MDH)^4–6^, closely resembling its calcium counterpart first discovered in 1967^7–9^. Both MDHs contains a redox-active cofactor, pyrroloquinoline quinone (PQQ) where the oxidation reaction takes place. Mechanistically, the redox cycle between PQQ and its natural substrate methanol proceeds through either an addition-elimination process with a hemiacetal intermediate or direct hydride transfer (Fig. 1a). In both cases, the active sites metals are believed to serve as only non-redox Lewis acid promoter^10–15^. This mechanistic scenario of Ln-MDH is distinctive from the established alcohol oxidation catalysts wherein redox-active metals such as Pd^16–20^, Cu^21–28^, and Fe^29–32^ are involved.

The unique mechanistic feature of MDH has drawn significant efforts in pursuing bio-inspired catalysis. Ohshiro demonstrated the synthetic application of PQQ in the oxidation of glucose^33–37^. Fukuzumi reported a model study of PQQ ester-Ca(ClO_4_)_2_ in the aerobic oxidation of alcohols^10,11^. Schelter synthesized a La-MDH model complex and investigated its catalytic performance in the dehydrogenation of benzyl alcohol with ferricenium ion as an oxidant^38^. In the latter two cases, efficient catalytic turnover was only observed in the presence of strong organic base (Note: During the revision of this article, a PQQ biomimetic work (*Chem. Eur. J*. 27, 10087-10098 (2021)) demonstrated the oxidation of 4-methyl benzylalcohol still proceeded without DBU, albeit with lower yield), but the efficiency was still too low to be of any synthetic utility, particularly when comparing with the redox-metal catalysts such as Pd^16–20^ or Cu-nitroxyl^21–28^ catalytic system in aerobic oxidation. To achieve effective quinone catalysis remains an open challenge from the synthetic point of view, in spite of the ubiquitous existence of quinone-enzyme in nature. Recently, we and others have developed bio-inspired *ortho*-quinone catalysts with the cofactor of copper amine oxidase, TPQ^39^ as a blueprint for the oxidation of amines^40–62^.

In this work, we report an *ortho*-quinone/lanthanum complex as highly effective aerobic oxidation catalyst. An open-shell semiquinone anionic radical species in complexing with lanthanum(III) ion is found to serve as the catalytically active species for the oxidation of alcohols under aerobic conditions. The catalysis is initialized by 1e^-^-reduction of lanthanum-activated *ortho*-quinone ***o*-Q** to a semiquinone-lanthanum complex La(***o*-Q**^**-**.^)_2_, which undergo a concerted dehydrogenation for aerobic oxidation of alcohols with up to 330 h^−1^ TOF (Fig. 1b).

## Results and discussion

### Establishment of optimal condition

**Fig. 2:**
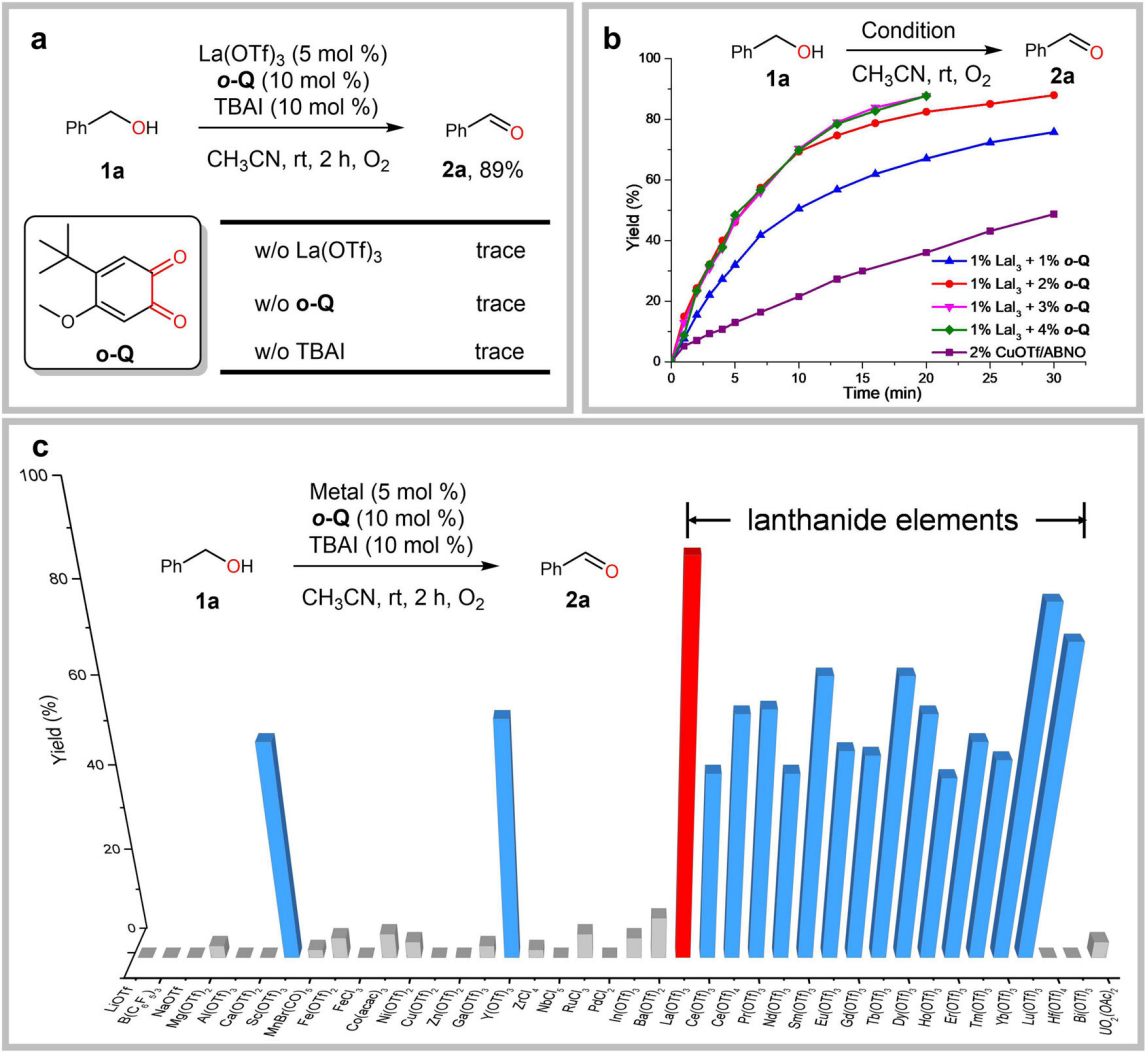
Selected reaction details. **a** Control experiment. **b** Kinetic profile of the reaction. CuOTf/ABNO condition: benzyl alcohol 1a (0.4 mmol), Cu(MeCN)OTf (2 mol %), ^4-MeO^bpy (2 mol %), ABNO (0.4 mol %), NMI (4 mol %), MeCN (4 mL), room temperature, O_2_ balloon. **c** Metal screening (for details, see Supplementary Table 1). Unless noted, reactions were conducted on a 0.4 mmol scale with 0.6 mL MeCN at room temperature under 1 atm O_2_, and yields were determined by GC using 1,3,5-trimethoxybenzene as internal standard. TBAI = Tetrabutylammonium iodide. ABNO = 9-Azabicyclo[3.3.1]nonane N-oxyl.

We initially found that a three-component system composed of La(OTf)_3_, *ortho*-quinone ***o*-Q** (for the performance of other quinones, see Supplementary Fig. 1) and *n*-Bu_4_NI was active for the aerobic oxidation of benzyl alcohol **1a** (Fig. 2a). A survey of different metal salts indicated all rare earth metals (REE) worked well in the reactions with lanthanum(III) ion as the optimal choice in terms of yields (Fig. 2c). In contrast, other metals including base metals such as Mg^2+^, Li^+^, Ca^2+^ or redox metals such as Cu^2+^, Fe^3+^ or Pd^2+^ showed rather poor activity or were even inert (Fig. 2c). We quickly identified LaI_3_ as the optimal choice in lieu of La(OTf)_3_ and iodide additive and the two-component catalyst LaI_3_/***o*-Q** was even more active. The reaction reached to completion in 20 mins with only 1 mol% loading of LaI_3_-(***o*-Q**)_2_ (standard condition) (Fig. 2b), showing significantly improved activity over the best Cu/nitroxyl system (CuOTf/ABNO)^25^. The optimal ratio of La/***o*-Q** was determined to be 1:2 and further increasing the loading of quinone did not lead to any further improvement (Fig. 2b).

### Scopes

**Fig. 3:**
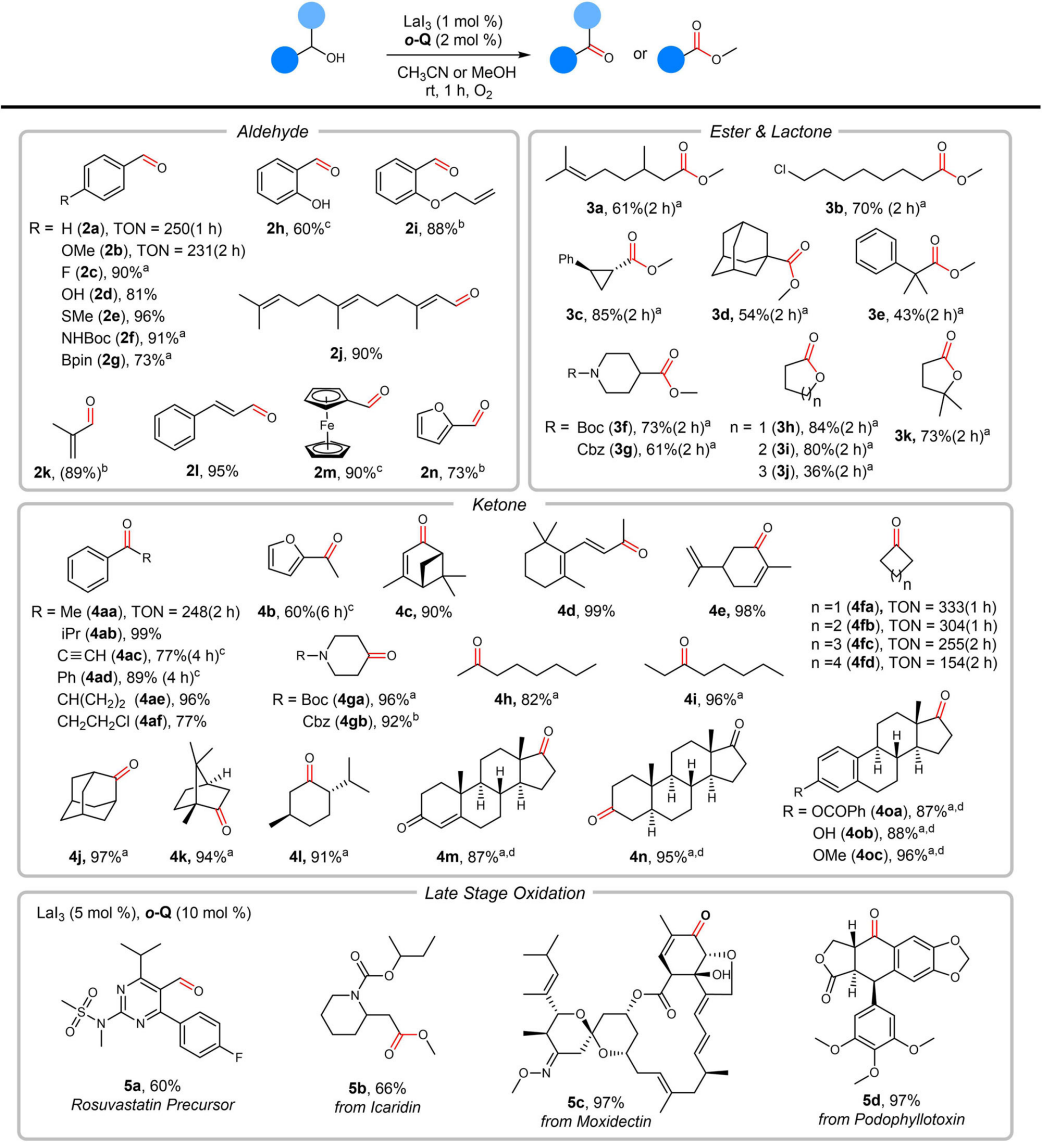
Substrate scope. Unless otherwise noted, reactions were conducted on a 1 mmol scale with 1 mL MeCN (for aldehyde, lactone and ketone) or MeOH (for ester) at room temperature under 1 atm O_2_, and yields were determined by flash column chromatography. Yields in parentheses were determined by ^1^H NMR. For late stage oxidation, reactions were conducted on 0.2 mmol scale with LaI_3_ (5 mol%), ***o*-Q** (10 mol%) at room temperature under 1 atm O_2_ and yields were determined by flash column chromatography (for details, see Supplementary Information). ^a^LaI_3_ (2 mol%), ***o*-Q** (4 mol%). ^b^LaI_3_ (4 mol%), ***o*-Q** (8 mol%). ^c^La(OTf)_3_ (5 mol%), ***o*-Q** (10 mol%), nBu_4_NI (15 mol%) for 2 h. ^d^MeCN/DCM (v/v = 1:1, 0.5 M).

The substrate scope was then explored under the optimal reaction conditions. The reactions worked well with *para*-substituted benzyl alcohols bearing either electron-donating (Figs. 3, **2a**, **b**,** i**) or electron-withdrawing substituents (**2c**). The reaction also tolerated functional groups such as thioether (**2e**), amine (**2** **f**), boronic acid ester (**2** **g**), and free phenols (**2d** and **2** **h**), to note that free phenols didn’t work well in the Cu/TEMPO catalysis system^24^. Heterocyclic aromatic alcohols such as ferrocene methanol (**2** **m**) and furfuryl alcohol (**2n**) and allylic alcohols (**2j-l**) could be equally applied in the current catalysis.

The reactions with aliphatic alcohols have also been examined to give a mixture of aldehyde and ester (from self-condensation). When the reaction was conducted in methanol, a sole formation of methyl ester could be achieved. Selected examples including long-chain alkyl (**3a** and **3b**), cyclopropyl (**3c**), bulky alkyl (**3d** and **3e**) and piperidinyl (**3f** and **3g**) were listed in Fig. 3, showing moderate to good activity. Diols such as 1,4-butanediol, 1,5-pentanediol and 1,6-hexanediol could be converted into the corresponding lactones (**3h-k**) with moderate to high yields. The catalysis also worked extremely well with secondary alcohols including aromatic (**4aa-af**), allylic alcohols such as verbenol and carveol (**4c-e**), acyclic (**4h-i**) and cyclic secondary alcohols (**4fa-gb**). The alcohols oxidation with testosterone (**4** **m**), androsterone (**4n**) and estradiol derivates (**4oa-oc**) went well under this catalysis. Several pharmaceutical intermediates and pesticides were also tested to demonstrate the applicability of our catalysis in the late stage functionalization. Activated benzyl alcohol (Rosuvastatin precursor, **5a**) and primary aliphatic alcohol (Icaridin, **5b**) would be oxidized to corresponding aldehyde and ester with moderate yields. And secondary alcohol (Moxidectin, **5c** and Podophyllotoxin, **5d**) proceeded well with nearly quantitative yields. Large scale oxidations were also conducted and more than 200 turnover numbers could be achieved in one hour for benzylic alcohol (**2a**) and both aromatic and aliphatic alcohols (**4aa** and **4fa**).

**Fig. 4:**
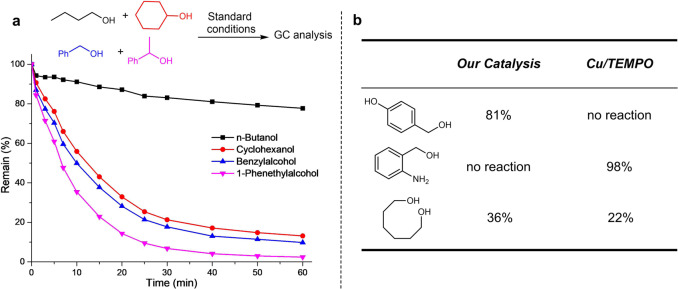
Comparison of our catalyst system with Cu/TEMPO system. **a** Competition experiments monitored by GC analysis. Standard conditions: LaI_3_ (0.004 mmol), ***o*-Q** (0.008 mmol), each alcohol substrates (0.1 mmol), MeCN (0.6 mL), room temperature, O_2_ balloon. **b** Comparison of our catalyst system and Cu/TEMPO system.

A competitive one-pot experiment was conducted to investigate the chemoselectivity (Fig. 4a). It was noted that the reaction rates with secondary alcohol cyclohexanol and those activated primary alcohols such as benzyl alcohol and 1-phenethylalcohol are comparable and they reacted much faster than aliphatic alcohol such as 1-butanol. In comparison, the typical Cu/TEMPO showed significantly preference to primary alcohols over secondary alcohols^24^. Our quinone catalytic system could tolerate free phenol but not free anilines, which is distinctive from Cu-TEMPO system (Fig. 4b). In oxidizing sluggish 1,6-hexanediol, the quinone catalyst performed slightly better than Cu/ABNO system^26^.

### Control experiments

**Fig. 5:**
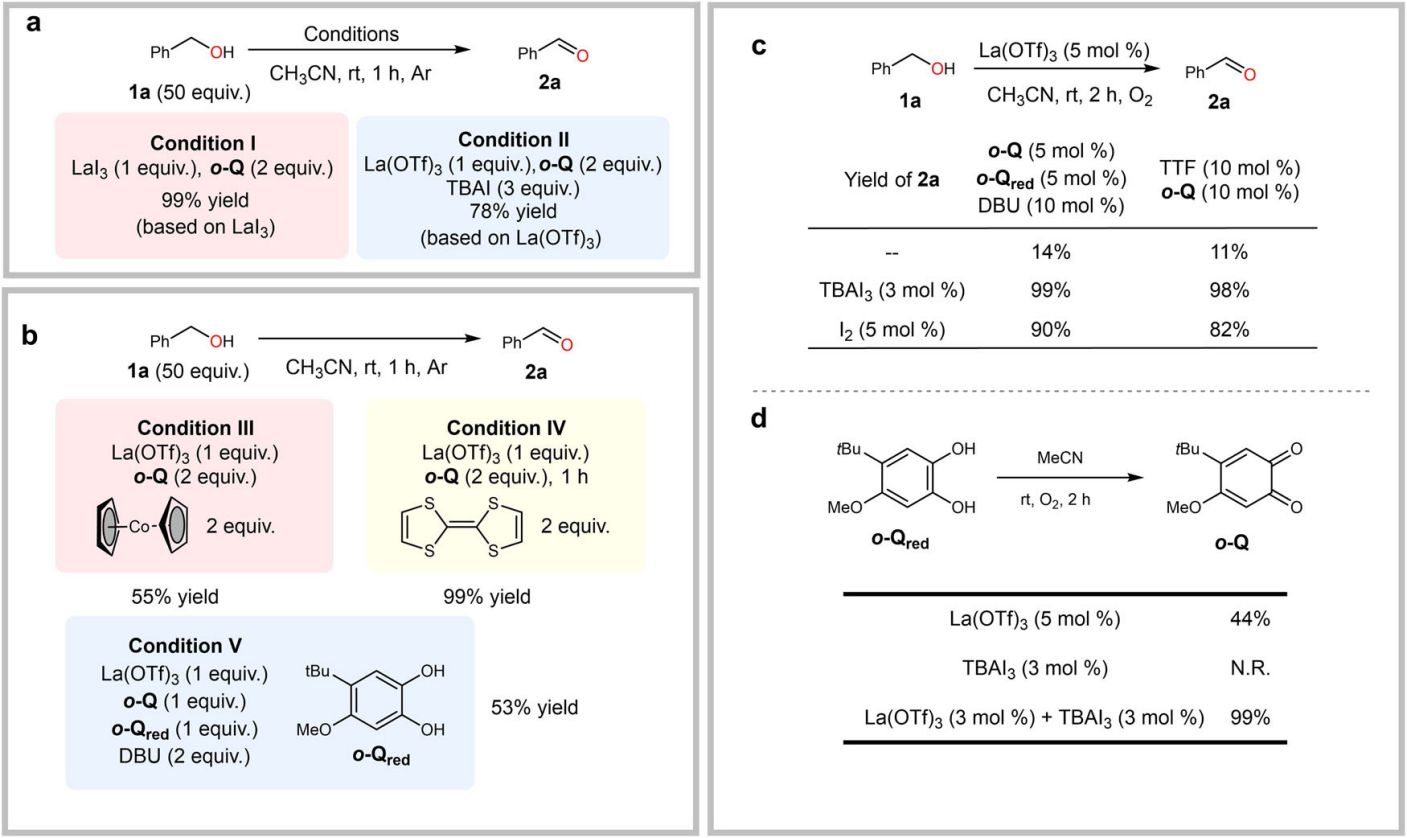
Control experiment. **a** Stochiometric reactions. **b** Other in situ generated semiquinone species control experiments. Yields were based on the amount of La^3+^. **c**, **d** Role of iodine. TTF = tetrathiafulvalene. DBU = 1,8-diazabicyclo[5.4.0]undec-7-ene. TBAI_3_ = tetrabutylammonium triiodide.

The real active catalytic species was first investigated. In control experiments, removing any of the three catalytic species, ***o*-Q**, La(OTf)_3_ or TBAI completely shut down the reaction (Fig. 2a), which implied La^3+^, *ortho*-quinone catalyst and iodide additive were all essential for this aerobic oxidation. Stoichiometric reactions with either LaI_3_-***o*-Q** or La(OTf)_3_-***o*-Q**-TBAI proceeded smoothly under argon (Fig. 5a, Condition **I** and **II**), suggesting that the substrate-oxidizing active specie was generated from LaI_3_ and ***o*-Q**, and oxygen served as the terminal oxidant for the recycling of the catalyst.

### Characterizations and verification of the active species

**Fig. 6:**
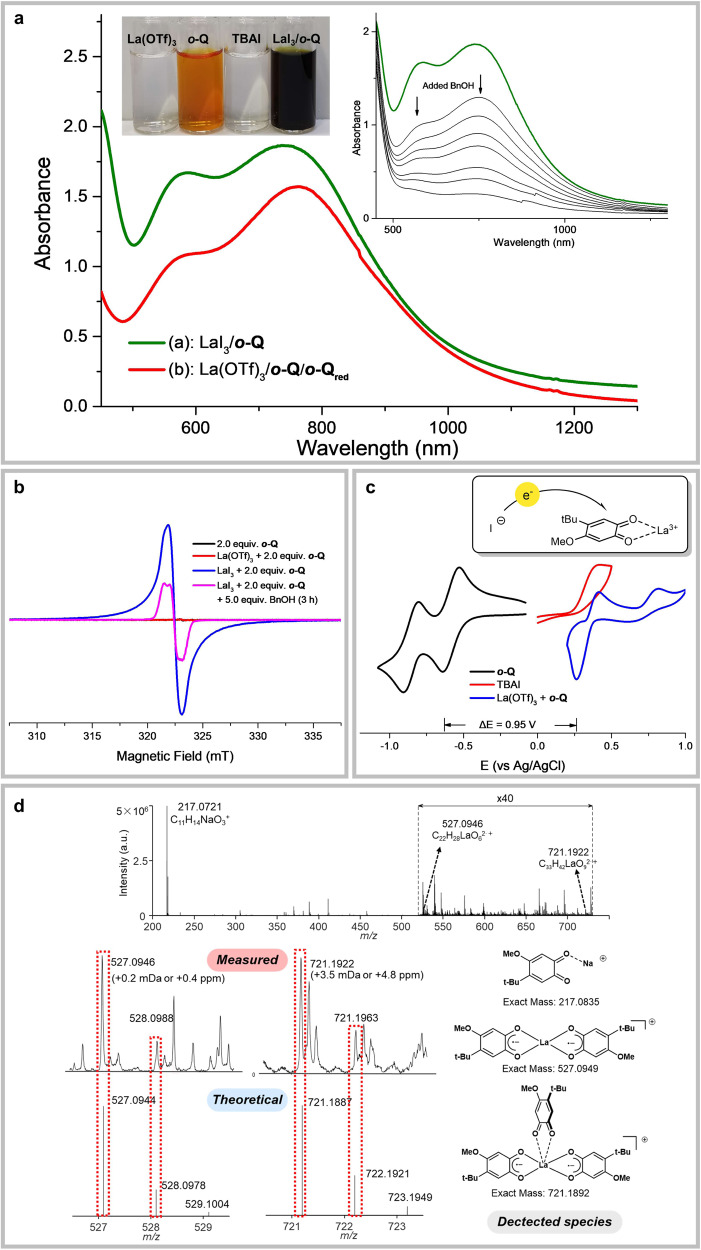
Characterization of active species. **a** UV–Vis spectrum. Sample concentration: 1.0 mM in MeCN. (**a**): LaI_3_ (1.0 mM) and *o*-Q (2.0 mM); (**b**): La(OTf)_3_ (1.0 mM), *o*-Q (1.0 mM), *o*-Q_red_ (1.0 mM), DBU (2.0 mM). Inset: The time profile of stoichiometric benzyl alcohol quenching experiment. (Supplementary Fig. 5). **b** EPR spectrum. Sample concentration: LaI_3_ (0.1 M) and *o*-Q (0.2 M) in MeCN at 298 K. **c** CV test. Sample concentration: 4.0 mM in electrolyte solution (0.1 M nBu_4_NPF_6_ in MeCN). **d** High-resolution mass spectrum of LaI_3_ and *o*-Q in MeCN at 298 K.

Dramatic color change was noted during the reaction process. When iodide additive was added to a solution of La(OTf)_3_ and ***o*-Q**, or upon mixing LaI_3_ with ***o*-Q**, an instant color change from red to dark green was observed (Fig. 6a) and the color changed back to red when the reaction was complete. UV–visible spectrum of the dark green solution indicated a new absorption at 570 and 750 nm. Previously, similar absorptions were reported for semiquinone radical anion in the presence of metal ion such as Sc(III) or Zn(II) (Fig. 6a)^63,64^. It should be noted a Ce-MDH was crystallized with their active site existed in the form of a Ce(III)-semiquinone anionic radical complex^65^. However, its mechanistic relevance remains obscure. EPR spectrum of the catalytic system confirmed the existence of an organic radical species with *g* = 2.003 (peak-to-peak linewidth is 1.2 mT) (Fig. 6b), and the simulation supported a semiquinone radical in coordination with La(III) (Supplementary Fig. 2)^66^. In comparison, no obvious EPR signal was detected with only *ortho*-quinone.

Cyclic voltammograms (CV) of ***o*-Q** showed a reduction peak at −0.63 V (vs Ag/AgCl), and this was shifted to 0.32 V (vs Ag/AgCl) in the presence of La(OTf)_3_, a positive shift as large as 0.95 V (Fig. 6c). All the other REE metals also showed large but varied positive shift of the reduction potential of ***o*-Q** (Supplementary Fig. 3). Hence, the coordination of quinone by coordination to lanthanides^38^ could dramatically facilitate its single electron oxidation of iodide (TBAI) (*E*_ox_ = 0.39 V vs Ag/AgCl). In comparison, the redox potential gap between free ***o*-Q** and TBAI was 1.03 V, largely disfavored for electron transfer^67^, and we did not observe any obvious change when mixing only ***o*-Q** and TBAI. Next, reductive initiators other than iodide have been tested and the addition of cobaltocene or tetrathiafulvalene could also initiate the stochiometric reaction with comparable activity (Fig. 5b, Condition **III** and **IV**).

Furthermore, the oxidation could also proceed when equal amount of ***o*-Q** and hydroquinone ***o*-Q**_**red**_ were employed, such a combination was known to be able to generate semiquinone radical anionic species under basic conditions and this was verified by UV–vis in our case (Fig. 6a and Fig. 5d, Condition **V**)^64^. Moreover, adding benzyl alcohol into the dark green solution of LaI_3_/(***o*-Q**)_2_ showed gradually decrease of adsorption as monitored by UV–visible spectroscopy (Fig. 6a) and increasingly formation of benzaldehyde was observed by GC (Supplementary Fig. 5). Similar quenching of the EPR signal was also clearly noted (Fig. 6b), indicating the consumption of the semiquinone intermediate. Taken together, these results strongly supported the involvement of the reductively generated semiquinone anionic radical in the active catalytic cycle and iodide served as a single electron donor for reductive generation of the active species.

We have tried to elucidate the structure of the La(III)-semiquinone complex in both solid and solution phase. Unfortunately, efforts to determine the solid structure by crystalizing the possible complex have been in vain. We then investigated the solution phase complexation between lanthanum (III) and semiquinones by ESI-MS. The MS spectra showed several signals clearly indicating the existence of La-semiquinone complexes (Fig. 6d). We were able to identify a major peak at 527.0946, ascribing to the expected 1:2 complex of La(***o*-Q**^**-**.^)_2_^+^ (Fig. 6d). A minor peak at 721.1922 could also be assigned as La(***o*-Q**^**-**.^)_2_^+^ binding with an additional neutral *o*-**Q**, an indication of dynamic and multi- binding behavior between La(III) and quinone/semiquinone.

### Proposed catalytic cycle

**Fig. 7:**
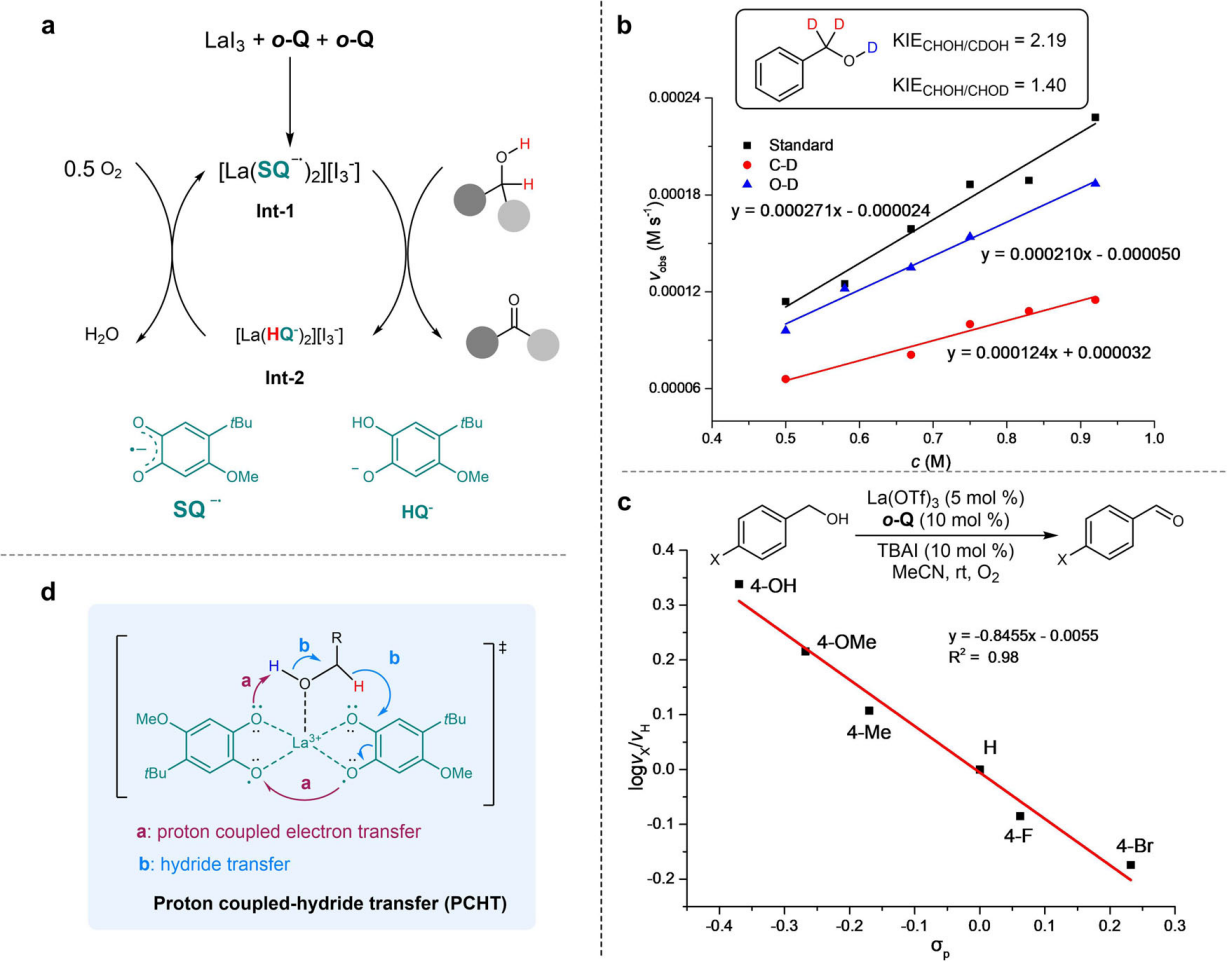
Proposed catalytic cycle and kinetic studies. **a** Proposed catalytic cycle. **b** Kinetic isotope effects of benzyl alcohol 1a. **c** Hammett correlation of *para*-substituted benzyl alcohols. **d** Proposed proton-coupled hydride transfer model.

Based on the above experimental observations, a catalytic cycle was proposed as shown in Fig. 7a. After coordinating with LaI_3_, ***o*-Q** would be reduced by iodide anion via SET to form a radical dianion complex (**Int-1**) involving a La(III) metal center coordinated with at least two molecules of semiquinone radical anion **SQ**^**–∙**^. Alcohol would then coordinate with lanthanum center, following by oxidative dehydrogenation to afford the corresponding aldehyde or ketone. Ester and lactone could be formed via second dehydrogenation from a semi-acetal intermediate. The reduced hydroquinone **HQ**^**–**^ complex (**Int-2**) would be re-oxidized by oxygen to regenerate **Int-1** (Fig. 7a). In the kinetic analysis of the reaction progress, we observed product inhibition effect (Supplementary Fig. 14), adding support to the coordination mode. In addition, the preference for ester formation with aliphatic alcohols can also be explained by the relatively strong binding of aliphatic aldehyde with lanthanum metal comparing with benzaldehyde.

### Mechanism of the dehydrogenation process

In Fukuzumi and Schelter’s biomimic quinone catalysis, organic base was essential to facilitate the oxidative process and the reactions were believed to proceed via a stepwise deprotonation and hydride transfer mechanism, and the latter step may follow either an addition-elimination or direct hydride transfer pathway. In our case, no obvious base effect was observed (Supplementary Table 6) and KIE effect was found on both O–H and C–H of benzylic alcohol (KIE_O–H_ = 1.40, KIE_C–H_ = 2.19) (Fig. 7b)^68–70^. These observations were supportive of a coupled O–H/C–H hydrogen transfers instead of stepwise deprotonation and hydride transfer. Each of the two semiquinone moieties **SQ**^**–∙**^ may concurrently accept a hydrogen (Fig. 7a). A Hammett plot with *para*-substituted benzyl alcohols revealed a preference for electron-rich substitutions with *ρ* = −0.84 (Fig. 7c), suggesting that positive charge was developing during H-transfer, an indication of hydride transfer. At this point, the detailed H-transfer mechanism remains to be elucidated, pending further structural characterization of the active intermediate (e.g. **Int-1** or **Int-2**). We proposed a proton-coupled hydride transfer (PCHT) pathway to account for the experimental observations (Fig. 7d). Concerted H-atom transfer, though can not be completely excluded, was unlikely considering the rather low BDE of hydroquinone ***o*-Q**_**red**_ (75 kcal/mol by DFT vs 96 kcal/mol for C-H bond in methanol). The absence of radical-rearrangement products with radical-probe substrates (e.g. **2i**, **3c**, and **4fa**) also disproved the existence of discrete radical intermediates during hydrogen-transfer, and is in support of hydride transfer. Preliminary DFT calculations were conducted to support the proposed PCHT pathway (Supplementary Fig. 16).

Inspired by natural lanthanide-dependent enzyme, Ln-MDH, we have developed in this work an efficient LaI_3_/*ortho*-quinone aerobic oxidation catalyst for alcohol oxidation. The lanthanide-*ortho*-quinone catalysis demonstrated high activity over a broad range of alcohols, providing practical accesses to aldehydes, ketones as well as esters. Mechanistic studies uncovered that a lanthanum complex with semiquinone radical anion served as the real active catalytic species and the dehydrogenation proceeded most likely via proton coupled-hydride transfer. Though semiquinone radicals are frequently observed in quinoproteins, their function remains obscure. This study implies a possible functioning mode of semiquinone radicals beyond simply positing as a recycling intermediate of quinone cofactors. From the synthetic point of view, the reductive activation strategy as well as the resulted radical anion as redox ligand provides a new twist in exploring aerobic oxidation catalysis.

## Method

### General procedure for alcohol oxidation

A flame-dried 10 mL flask was flushed with O_2_ and equipped with an O_2_ balloon. LaI_3_ (5.19 mg, 0.01 mmol) were added to the solution of ***o*-Q** (3.88 mg, 0.02 mmol) and alcohol (1.0 mmol) in 1.0 mL of CH_3_CN. The reaction was stirred at room temperature for 1 h. After the reaction was completed, the crude reaction product was purified though a silica gel using 1:10-1:5 EtOAc/petro ether to give a pure product. For some volatile aldehyde, yields were determined by ^1^H NMR.

## Supplementary information

Supporting information

## Data Availability

All other data that support the findings of this study are available within the article and its Supplementary Information files. The datasets generated during and/or analyzed during the current study are available from the corresponding author on request. Source data are provided with this paper.

## Source data

Source Data
