# Supplementary material for: Bio-inspired lanthanum-ortho-quinone catalysis for aerobic alcohol oxidation: semi-quinone anionic radical as redox ligand
Source: Nat Commun. 2022 Jan 20;13:428. doi: 10.1038/s41467-022-28102-4 (PMC8776754; doi:10.1038/s41467-022-28102-4)
Supplement: Supplementary file 1 — Supporting information [file 41467_2022_28102_MOESM1_ESM.pdf]

Supplementary Information

Bio-inspired Lanthanum-*ortho*-Quinone Catalysis for Aerobic Alcohol Oxidation: Semi-quinone Anionic Radical as Redox Ligand

Ruipu Zhang, Runze Zhang, Ruijun Jian, Long Zhang, Ming-Tian Zhang, Yu Xia, Sanzhong Luo*

Correspondence to: luosz@tsinghua.edu.cn

Table of Contents

1. Supplementary Notes··2
2. Supplementary Discussion····································3
3. Supplementary Methods······································40
4. Supplementary References···································114

1. Supplementary Notes

Materials and Methods. ^{1}H, and ^{13}C NMR spectra were measured on Bruker AV 400 M and Bruker AVANCE III HD 500MHz (400 MHz or 500 MHz for ^{1}H NMR; 101 MHz, or 126 MHz for ^{13}C NMR). Chemical shifts of ^{1}H NMR spectra were recorded relative to TMS (δ 0.00) or residual protonated solvents ($CDCl_3$: δ 7.26; DMSO-d_6: δ 2.50). Chemical shifts of ^{13}C NMR spectra were recorded relative to solvent resonance ($CDCl_3$: δ 77.16; DMSO-d_6: δ 40.0). ^{13}C NMR spectra were obtained at 101 or 126 MHz using a proton-decoupled pulse sequence and were tabulated by the observed peak. The following abbreviations were used to express the multiplicities: s = singlet; d = doublet; t = triplet; q = quartet; m = multiplet; br = broad. Silica gel (200-300 mesh) was used for column chromatography. Unless otherwise noted, reagents obtained from commercial suppliers were used without further purification. The *ortho*-quinone catalysts were prepared according to the corresponding literature[1]. GC analysis was performed on a Shimadzu GC-2030 instrument equipped with an FID detector using argon as the carrier gas. UV-Vis spectrums were obtained from Hitachi U-3000 and Perkin Elmer Lambda 950. EPR spectrums were collected using JEOL FA-200 instrument. Cyclic voltammograms were collected with a Shanghai Chenhua CHI660E potentiostat.

2. Supplementary Discussion

Kinetic profile of the reaction. To a Schlenk tube sealed with rubber stopper, LaI_3 (0.004 mmol, 2.0 mg), ***o*-Q** (0.008 mmol, 1.6 mg) and 1,3,5-trimethoxybenzene in 0.4 mL of MeCN was added. The reaction tube was flushed with O_2. Then the solution of benzylalcohol **1a** (0.4 mmol, 43.2 mg) in 0.2 mL MeCN was added into the reaction tube. Taking 20 μL reaction solution (filtered through Celite) for GC analysis in every 2-5 mins. The results were presented in **Figure 2b**.

Stoichiometric reaction. In a glove box, benzylalcohol **1a** (2.5 mmol) were added to the solution of ***o*-Q** (0.1 mmol, 19.4 mg) and LaI_3 (0.05 mmol, 26.0 mg) in 1 mL of CH_3CN. The reaction was stirred at room temperature for 1 h. After 1 h, the reaction was quenched by benzoic acid and yields were determined by GC using 1,3,5-trimethoxybenzene as internal standard based on the amount of La^{3+}. The results were presented in **Figure 5a**. Reactions in **Figure 5b** were conducted following the similar experiment procedure.

Intermolecular chemoselectivity experiments. To a Schlenk tube sealed with rubber stopper, LaI_3 (0.004 mmol, 2.0 mg), ***o*-Q** (0.008 mmol, 1.6 mg) and 1,3,5-trimethoxybenzene in 0.4 mL of MeCN was added. The reaction tube was flushed with O_2. Then the solution of benzylacohol (0.1 mmol, 10.8 mg), n-butanol (0.1 mmol, 7.4 mg), cyclohexanol (0.1 mmol, 10.0 mg) and 1-phenethylalcohol (0.1 mmol, 12.2 mg) in 0.2 mL MeCN was added into the reaction tube. Taking 20 μL reaction solution for GC analysis in every 2-5 mins. The results were presented in **Figure 4a**.

In situ detection of active catalytic species. In the glove box, LaI_3 (5.2 mg, 0.01 mmol) and ***o*-Q** (3.88 mg, 0.02 mmol) were dissolved in 1.0 mL of degassed dry MeCN which was used as mother liquor. 20 μL mother solution were diluted to 1.0 mL MeCN for HRMS analyzation. The results were presented in **Figure 6d**. The high-resolution mass spectrums were obtained using a Bruker Maxis Impact Q-TOF mass spectrometer (Bruker Daltonics GmbH, Bremen, Germany) equipped with a home-built nanoESI source. NanoESI tips (~10μm o.d.) were pulled from borosilicate glass capillaries (1.5 mm o.d. and 0.86 mm i.d.) using a micropipette puller (P-1000 Flaming/Brown; Sutter Instrument, Novato, CA, USA).

Optimization of Conditions. A flame-dried 10 mL flask was flushed with O_2 and equipped with an O_2 balloon. Benzylalcohol **1a** (0.4 mmol, 43.2 mg) were added to the solution of $La(OTf)_3$ (0.02 mmol, 11.7 mg), ***o*-Q** (7.76 mg, 0.04 mmol) and TBAI (14.7 mg, 0.04 mmol) in 0.6 mL of CH_3CN. The reaction was stirred at room temperature for 2 h. After the reaction was completed, yields were determined by GC using 1,3,5-trimethoxybenzene as internal standard.

Supplementary Table 1. Screening of Metal

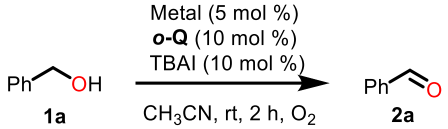

Entry	Metal	Yield (%)	Entry	Metal	Yield (%)
1	LiOTf	0	22	$In(OTf)_3$	5
2	$B(C_6F_5)_3$	0	23	$Ba(OTf)_2$	10
3	NaOTf	0	24	$La(OTf)_3$	89
4	$Mg(OTf)_2$	3	25	$Ce^{III}(OTf)_3$	44
5	$Al(OTf)_3$	0	26	$Ce^{IV}(OTf)_4$	57
6	$Ca(OTf)_2$	0	27	$Pr(OTf)_3$	58
7	$Sc(OTf)_3$	51	28	$Nd(OTf)_3$	44
8	$MnBr(CO)_5$	2	29	$Sm(OTf)_3$	65
9	$Fe(OTf)_2$	5	30	$Eu(OTf)_3$	49
10	$FeCl_3$	0	31	$Gd(OTf)_3$	48
11	$Co(acac)_3$	6	32	$Tb(OTf)_3$	65
12	$Ni(OTf)_2$	4	33	$Dy(OTf)_3$	57
13	$Cu(OTf)_2$	0	34	$Ho(OTf)_3$	43
14	$Zn(OTf)_2$	0	35	$Er(OTf)_3$	51
15	$Ga(OTf)_3$	3	36	$Tm(OTf)_3$	47
16	$Y(OTf)_3$	56	37	$Yb(OTf)_3$	80
17	$ZrCl_4$	2	38	$Lu(OTf)_3$	72
18	$NbCl_5$	0	39	$Hf(OTf)_4$	0
19	$RuCl_3$	6	40	$IrCl_3$	0
20	$PdCl_2$	0	41	$Bi(OTf)_3$	0
21	$AgBF_4$	0	42	$UO_2(OAc)_2$	4

Supplementary Table 2. Screening of Lanthanum Catalysts

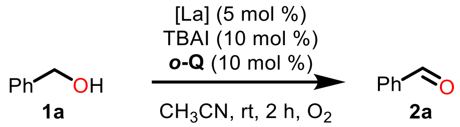

Entry	Lanthanum Catalysts	Yield (%)
1	$La(OTf)_3$	89
2	$La(Cp)_3$	5
3	$La(OH)_3$	3
4	$La(NO_3)_3$ $6H_2O$	47
5	$La(ClO_4)_3$ $6H_2O$	95
6[a]	LaF_3	4
7[a]	$LaCl_3$	4

[a]without TBAI

Supplementary Table 3. Screening of Additive

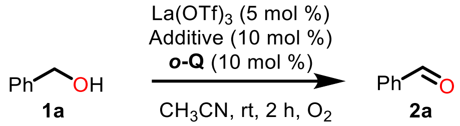

Entry	Additive	Yield (%)
1	TBAI	89
2	TBABr	5
3	$TBAClO_4$	3
4	$TBABF_4$	2
5	TBAOAc	2
6	TBAOH	8

Supplementary Table 4. Screening of Iodine Source

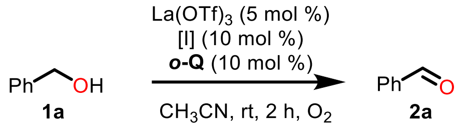

Entry	[I]	Yield (%)
1	TBAI	89
2	NH_4I	80
3	KI	80
4[a]	TEAI	69
5	ZnI_2	67

[a]TEAI = tetraethylammonium iodide.

Supplementary Table 5. Screening of Solvent

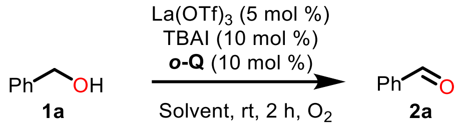

Entry	Solvent	Yield (%)
1	MeCN	89
2	Acetone	35
3	DCM	70
4	$PhCH_3$	76
5	Hexane	70
6	Et_2O	33
7	DMF	5
8	DMSO	3
9	Ethyl acetate	22

Supplementary Table 6. Screening of Acid and Base

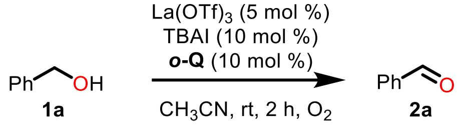

Entry	Deviation from above	Yield (%)
1	--	89
2	Additional DMAP (10 mol %)	87
3	Additional K_2CO_3 (10 mol %)	78
4	Additional HCOONa (10 mol %)	85
5	Additional $KHCO_3$ (10 mol %)	68
6	Additional PPTS (10 mol %)	18
7	Additional m-$NO_2C_6H_4COOH$ (10 mol %)	2
8	Additional AcOH (10 mol %)	2
9	Additional TsOH (10 mol %)	2
10	Additional $PhCO_2H$ (10 mol %)	4

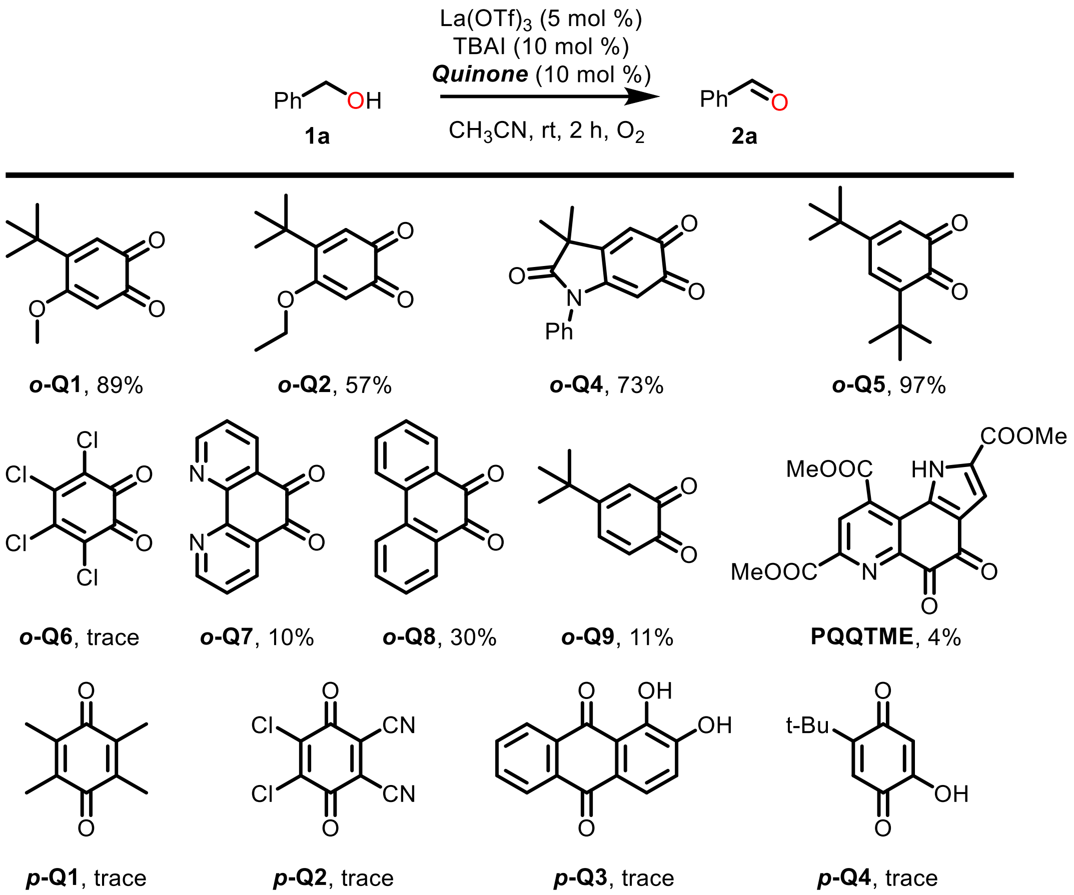

Supplementary Figure 1. Screening of different quinone catalysts.
[a]**1a** (0.2 mmol), La(OTf)$_3$ (2 mol %), PQQTME (2 mol %), TBAI (3 mol %), MeCN (0.5 mL), room temperature, air, 12 h. PPTS = pyridinium *p*-toluenesulfonate

PQQTME were prepared according to the corresponding literature[2]. PQQ was extracted from Doctor's Best Science-Based Nutrition BioPQQ capsules[3]. ^{1}H NMR (400 MHz, DMSO-d$_6$) δ 12.52 (s, 1H), 8.57 (s, 1H), 7.29 (d, *J* = 2.1 Hz, 1H), 4.06 (s, 3H), 3.97 (s, 3H), 3.90 (s, 3H). ^{13}C NMR (101 MHz, DMSO-d$_6$) δ 177.5, 173.8, 167.0, 164.3, 160.3, 149.4, 146.2, 134.6, 134.2, 128.9, 127.1, 126.9, 125.4, 114.3, 54.7, 53.4, 52.8. HRMS (ESI-Orbitrap) *m*/*z* [M + Na]$^+$ Calcd for $C_{17}H_{12}N_2NaO_8$ 395.0491, found 395.0482.

Supplementary Table 7. Control Experiments.

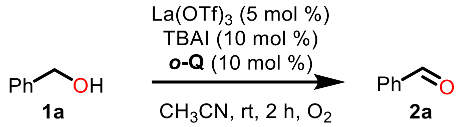

Entry	Deviation from above	Yield (%)
1	--	89
2	with TEMPO (1 equiv.)	90
3	with BHT (1 equiv.)	82
4	with 1,1-Diphenylethylene (1 equiv.)	99
5	with *p*-Benzoquinone (10 mol %)	84
6	with tBuOH (2 equiv.)	90
7	with Anthracene (1 equiv.)	99
8	Under Dark	89
9	10W 395 nm LED	87
10	10W 450 nm LED	91
11	36W CFL	99(99[a])

[a]The reaction tube was wrapped by tin foil. TEMPO = 2,2,6,6-tetramethyl-1-piperinedinyloxy; BHT = butylated hydroxytoluene.

EPR Spectrum.. In the glove box, LaI_3 (51.9 mg, 0.1 mmol) were added to the solution of ***o*-Q** (38.8 mg, 0.2 mmol) in 1.0 mL of degased CH_3CN at 298 K. The solution was stirred at room temperature for 5 min. Using capillarity tube to store the mixture for EPR analysis and sealed with plasticine to avoid the invasion of air. All measurements were performed under Ar. Conditions for EPR measurement: microwave power (0.998 mW), central field (323.4 mT), magnetic width (15.0 mT), modulation width (0.05 mT), time constant (0.03 s), measurement time (2 min). Substrate quenching experiments were conducted under the same condition with UV-Vis reaction monitor experiments.

EPR simulation. All the optimizations of molecular structures were carried out using the Gaussian 09[4] suite of programs employing the popular UB3LYP hybrid functional[5-7] and the def2svp basis set[8]. The effect of solvation on geometry was covered by employing the integral equation formalism variant (IEFPCM) of Tomasi's PCM method[9-11]. The ORCA electronic structure package[12] was used to calculate the g tensors. In these undertakings, the hybrid UB3LYP together with the TZVP basis set[10] were employed. The CPCM solvent model was used in the computations. The *g* tensors were computed using Neese's CPKS method[13] combined with an accurate mean field approximation [RISOMF(1X)][14] to the Breit–Pauli spin–orbit coupling operator[15,16]. The simulation of EPR spectrum was performed on the EasySpin software[17].

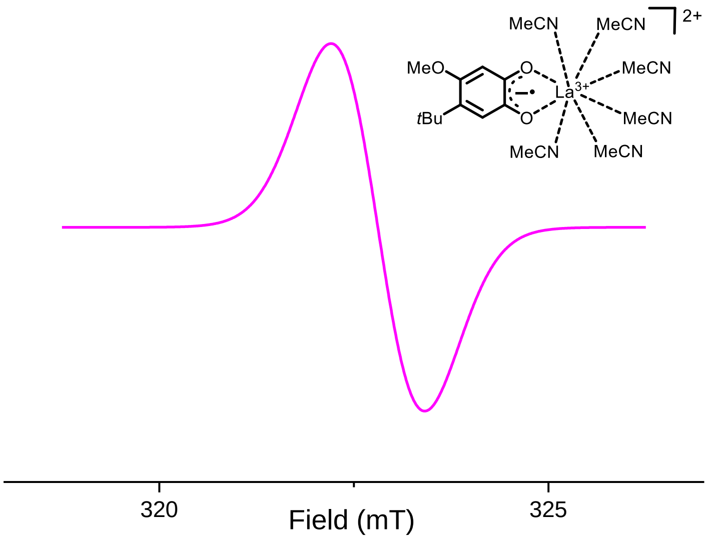

Supplementary Figure 2. EPR Simulation of Semiquinone-La Complex.

The coordinates for calculated structure

La	-1.44835900	0.00196700	0.22023800
O	0.69886300	1.20554500	0.10398100
C	1.78590800	0.53776400	-0.06140000
C	3.07141900	1.14290900	-0.16191000
C	4.23668000	0.41904400	-0.34220500
C	4.10283600	-1.03340500	-0.42473100
C	2.86617700	-1.66875800	-0.32997900
C	1.68029800	-0.92443400	-0.15093600
O	0.50506900	-1.44314600	-0.06105500
H	2.77863800	-2.75202600	-0.39422900
H	3.09101900	2.22910500	-0.09111600
C	5.60886400	1.11545900	-0.45341400
C	5.47600600	2.64685900	-0.33679000
H	4.85025100	3.07197800	-1.13661900
H	5.05422100	2.95507200	0.63231600
H	6.47455200	3.10230300	-0.42181800
C	6.25174000	0.81223200	-1.83016900
H	7.21784500	1.33647300	-1.91152300
H	6.43046100	-0.26026700	-1.97438200
H	5.60602700	1.17024000	-2.64835300
C	6.54249800	0.64525000	0.69007400
H	7.50965500	1.16884500	0.61765600
H	6.10464800	0.88403700	1.67284200
H	6.73296400	-0.43410500	0.64913300
O	5.24453000	-1.72078300	-0.59880700
C	5.22919200	-3.13878100	-0.69666600
H	4.83048800	-3.59735400	0.22250500
H	4.63134000	-3.47194000	-1.56023400
H	6.27293700	-3.44599400	-0.83483400
N	-2.35979500	-2.42740500	1.05710300
N	-2.04641600	-1.43655600	-1.98861500
N	-0.47817800	-0.26820100	2.75318100
C	-2.72124600	-3.47244500	1.39721800
C	-2.26704500	-2.05847300	-2.93872900
C	0.00760400	-0.38176900	3.79705300
C	0.62304500	-0.52325500	5.10492900
H	0.38662000	-1.51307200	5.52209000
H	1.71432200	-0.42023100	5.01304500
H	0.24134200	0.25561400	5.78124700
C	-3.17261300	-4.78629800	1.82025900

H	-2.77800600	-5.00946600	2.82237300
H	-4.27183100	-4.80965800	1.84994100
H	-2.81281500	-5.54695900	1.11181200
C	-2.54426300	-2.83897400	-4.13164700
H	-3.38634600	-3.52070200	-3.94207800
H	-2.80271700	-2.16664800	-4.96290700
H	-1.65609900	-3.42763500	-4.40451400
N	-1.96005100	2.38813300	1.40607000
C	-2.15100000	3.41189100	1.90991500
C	-2.38743000	4.69860000	2.54023500
H	-3.09396000	5.28443000	1.93411100
H	-2.80963400	4.54806700	3.54465600
H	-1.43899000	5.24925900	2.62454600
N	-4.17543200	0.19567300	-0.30925100
C	-5.30671500	0.27966800	-0.53753000
C	-6.72608900	0.38547000	-0.82920200
H	-6.96098500	-0.18275200	-1.74122300
H	-7.30963900	-0.02043300	0.01008700
H	-6.99668100	1.44075500	-0.98064900
N	-1.92711500	1.85026400	-1.70592000
C	-2.08654700	2.65527200	-2.52119000
C	-2.28618900	3.66727100	-3.54375200
H	-1.31825600	4.11070800	-3.81995100
H	-2.74284300	3.21080100	-4.43421900
H	-2.94964200	4.45658100	-3.16079900

CV Test. The sample (0.02 mmol) was added to the 5 mL MeCN solution (containing supporting electrolyte nBu_4NPF_6, 0.1 M). The ratio of metal salts and ***o*-Q** was 1:1. The redox potentials were determined under Ar using glassy carbon as the working electrode, Pt wire and Ag/AgCl (0.1 M) as counter and reference electrode at a 100 mV/s scan rate.

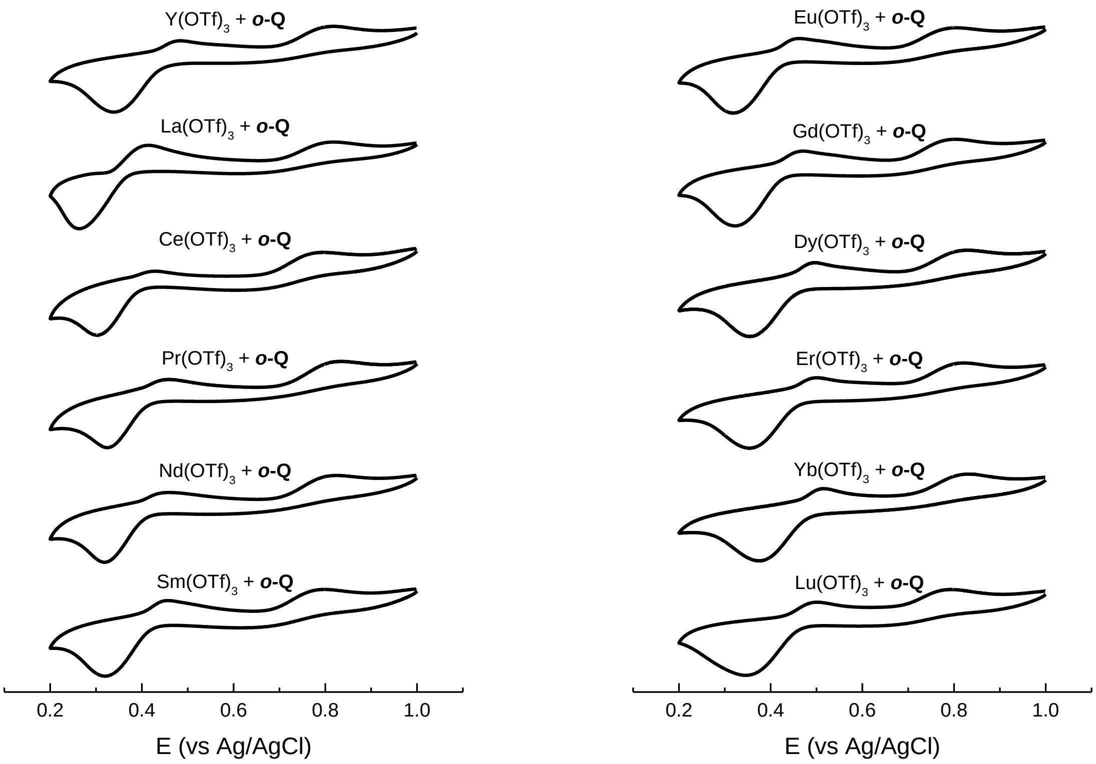

Supplementary Figure 3. The shift of reductive potential of *o*-Q by adding various rare earth elements.

Supplementary Table 8. Reduction potential of *o*-Q by adding various rare earth salts

REE Catalyst	Ep (vs Ag/AgCl)	REE Catalyst	Ep (vs Ag/AgCl)
$Y(OTf)_3$	0.38	$Eu(OTf)_3$	0.36
$La(OTf)_3$	0.31	$Gd(OTf)_3$	0.34
$Ce^{III}(OTf)_3$	0.32	$Dy(OTf)_3$	0.38
$Pr(OTf)_3$	0.32	$Er(OTf)_3$	0.39
$Nd(OTf)_3$	0.34	$Yb(OTf)_3$	0.38
$Sm(OTf)_3$	0.36	$Lu(OTf)_3$	0.41

UV-Vis Analysis. All the sample was added to the degassed MeCN in a glove box. Then the solution was transferred to the cuvette, sealed with parafilm. The UV-Vis spectrum was recorded on the Perkin Elmer Lambda 950 (450-1400 nm) and Hitachi U-3000 (220-600 nm). Unless noted, all the UV analysis were conducted under argon.

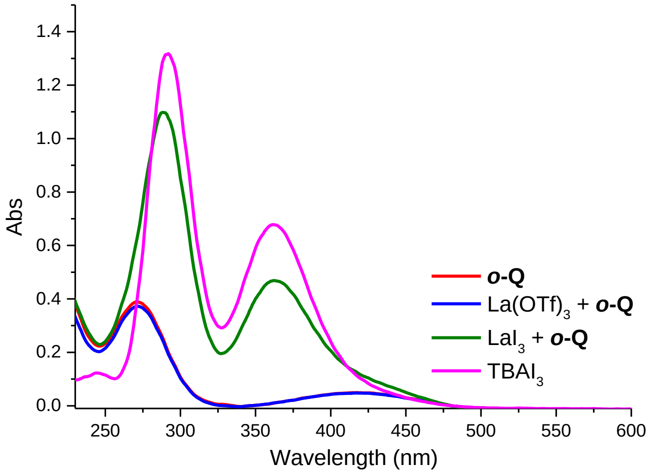

Supplementary Figure 4. UV-Vis spectrum. (0.05 mM in MeCN).

Reaction monitor by UV-Vis spectrum. In the glove box, LaI_3 (103.8 mg, 0.2 mmol) were added to the solution of ***o*-Q** (77.8 mg, 0.4 mmol), and benzylalcohol **1a** (64.8 mg, 0.6 mmol) in 2.0 mL of CH_3CN. The reaction was stirred at room temperature. Taking 20 μL reaction solution for UV-Vis analysis which were diluted to 2.0 mL by degassed MeCN. And taking another 20 μL reaction solution for GC analysis which were quenched by excess benzoic acid. The results were presented in Fig. S5.

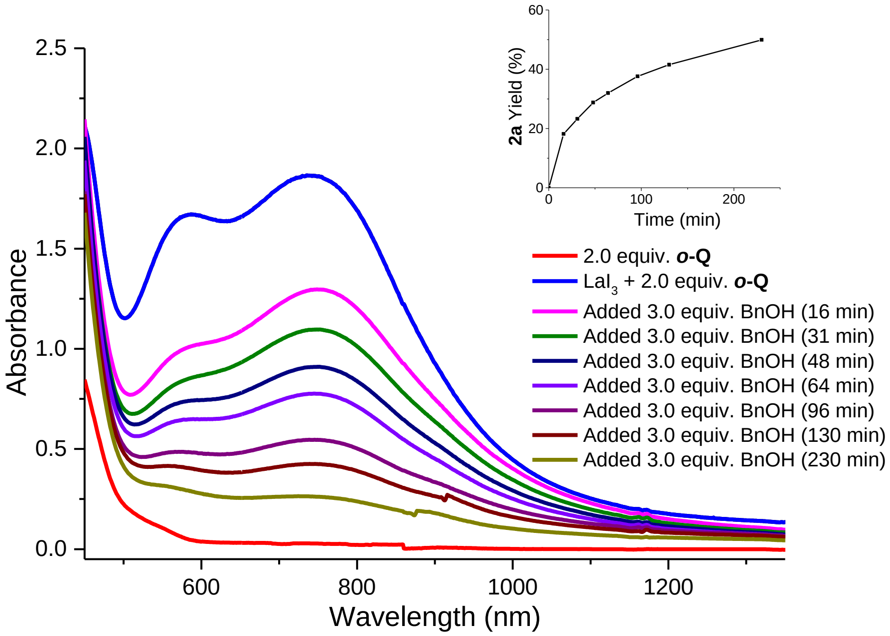

Supplementary Figure 5. Reaction monitor by UV-Vis spectrum. (1.0 mM in MeCN). Inset: product yield monitor by GC analysis (yield based on LaI_3).

Oxidation of Catechol *o*-Q_{red}. A flame-dried 10 mL flask was flushed with O_2 and equipped with an O_2 balloon. Additive were added to the solution of ***o*-Q_{red}** (0.05 mmol, 9.7 mg) in 0.5 mL of CH_3CN. The reaction was stirred at room temperature for 2 h. The yield of ***o*-Q** was determined by ^{1}H NMR.

UV-Vis Ananlysis. In a glove box, La(OTf)$_3$ (0.025 mmol, 14.7 mg), DBU (0.1 mmol, 15.0 mg), TBAI$_3$ (0.025 mmol, 15.6 mg) and ***o*-Q_{red}** (0.05 mmol, 9.7 mg) was added to the volumetric flask which was diluted to 25 mM by degassed MeCN (1 mL). Take 160 μL solution to the volumetric flask and dilute to 1 mM solution (4 mL). Then the solution was transferred to the cuvette, sealed with parafilm. The UV-Vis spectrum was recorded on the Perkin Elmer Lambda 950. Unless noted, all the UV analysis were conducted under argon.

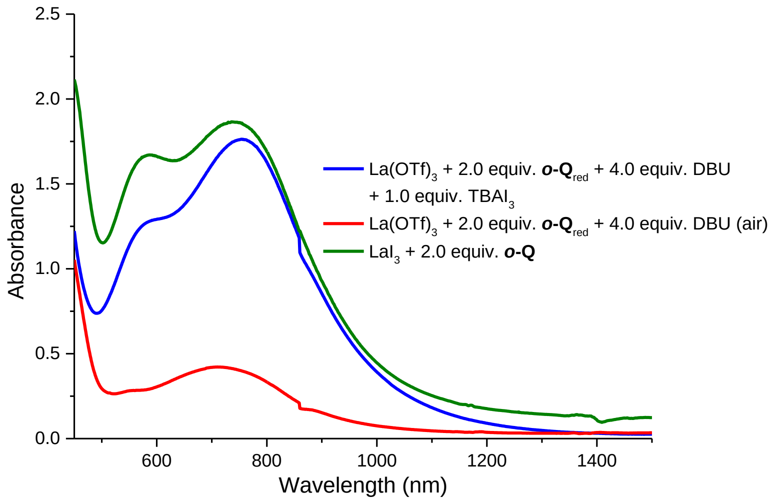

Supplementary Figure 6. UV-Vis spectrum of reoxidation of catechol ***o*-Q_{red}** under basic condition.

La(OTf)$_3$ (0.00125 mmol, 0.7 mg) and ***o*-Q$_{red}$** (0.025 mmol, 4.9 mg) were added into the a flame-dried 10 mL flask. The flask was flushed with O_2 and equipped with an oxygen balloon. Then adding 1 mL MeCN to the flask (Supplementary Fig. 7, 1). The solution of DBU (0.0025 mmol, 0.4 mg) in 0.5 mL MeCN was added into the flask (Supplementary Fig. 7, 2-6).

Condition: ***o*-Q$_{red}$** (0.025 mmol), La(OTf)$_3$ (0.00125 mmol), DBU (0.0025 mmol), O_2, MeCN

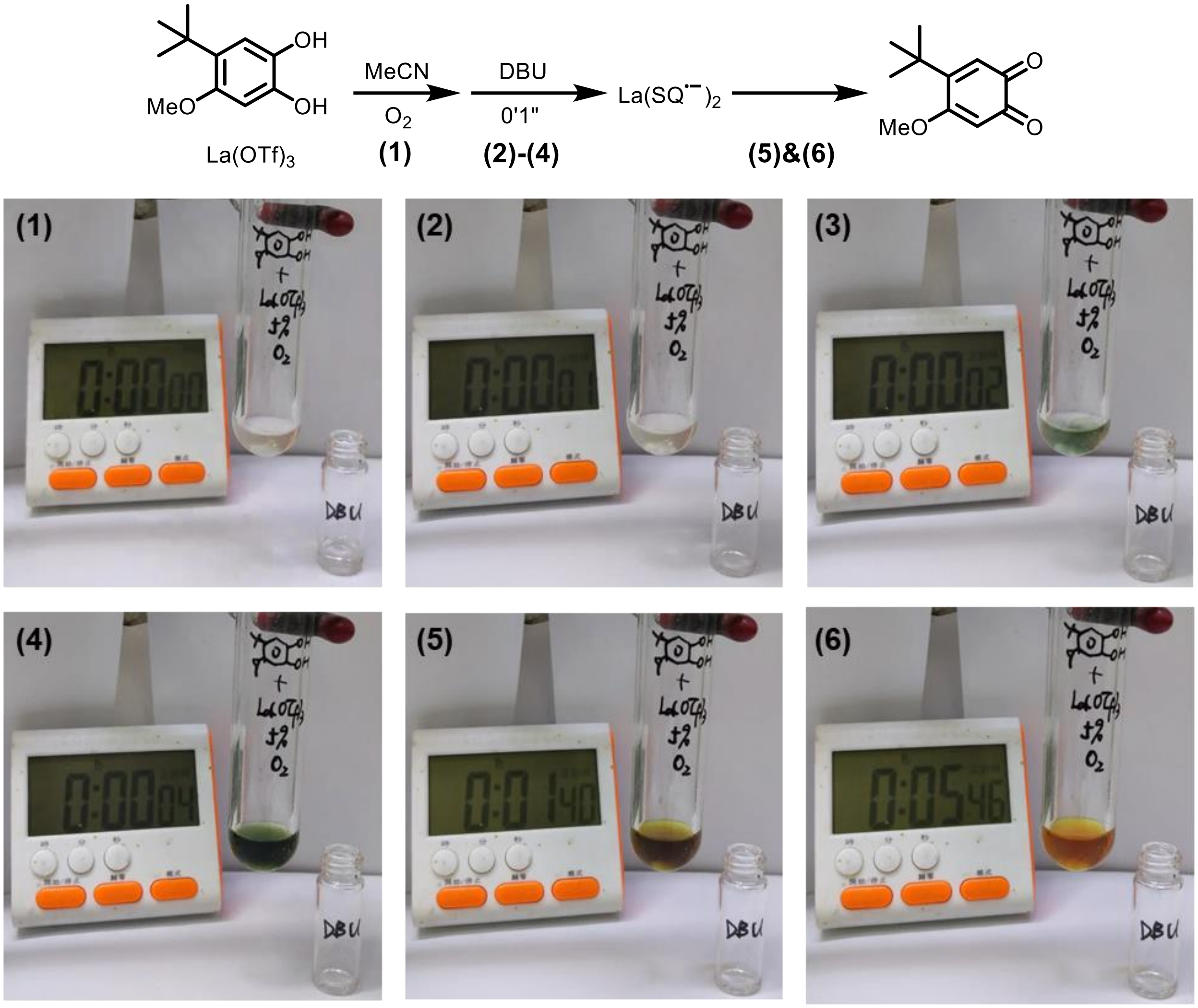

Supplementary Figure 7. Time course of reoxidation of catechol ***o*-Q$_{red}$** with lanthanum under basic condition.

La(OTf)3 (0.0125 mmol, 7.3 mg) and ***o*-Q**$_{red}$ (0.025 mmol, 4.9 mg) were added into the a flame-dried 10 mL flask. The flask was flushed with Ar. Then adding 1 mL MeCN to the flask (Supplementary Fig. 8, 1). Adding the solution of TBAI3 (0.025 mmol, 15.6 mg) in 0.5 mL MeCN to the flask (Supplementary Fig. 8, 2). The solution of DBU (0.025 mmol, 3.8 mg) in 0.5 mL MeCN was added into the flask (Supplementary Fig. 8, 3 and 4).

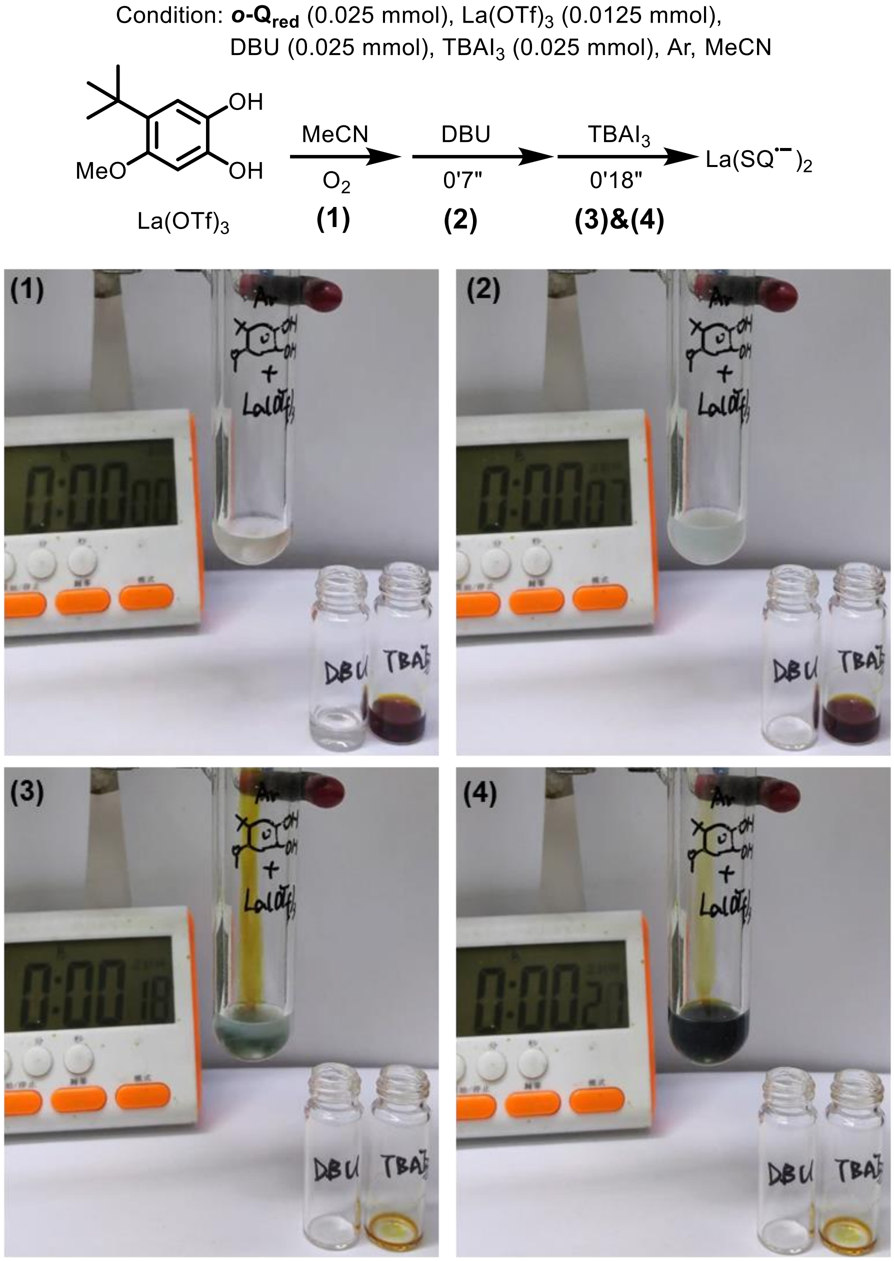

Supplementary Figure 8. Time course of reoxidation of catechol ***o*-Q**$_{red}$ with lanthanum under basic and anaerobic condition.

***o*-Q_{red}** (0.025 mmol, 4.9 mg) was added into the a flame-dried 10 mL flask. The flask was flushed with O_2 and equipped with an oxygen balloon. Then adding 1 mL MeCN to the flask (Fig. S9, 1). The solution of DBU (0.05 mmol, 7.5 mg) in 0.5 mL MeCN was added into the flask (Fig. S9, 2-4).

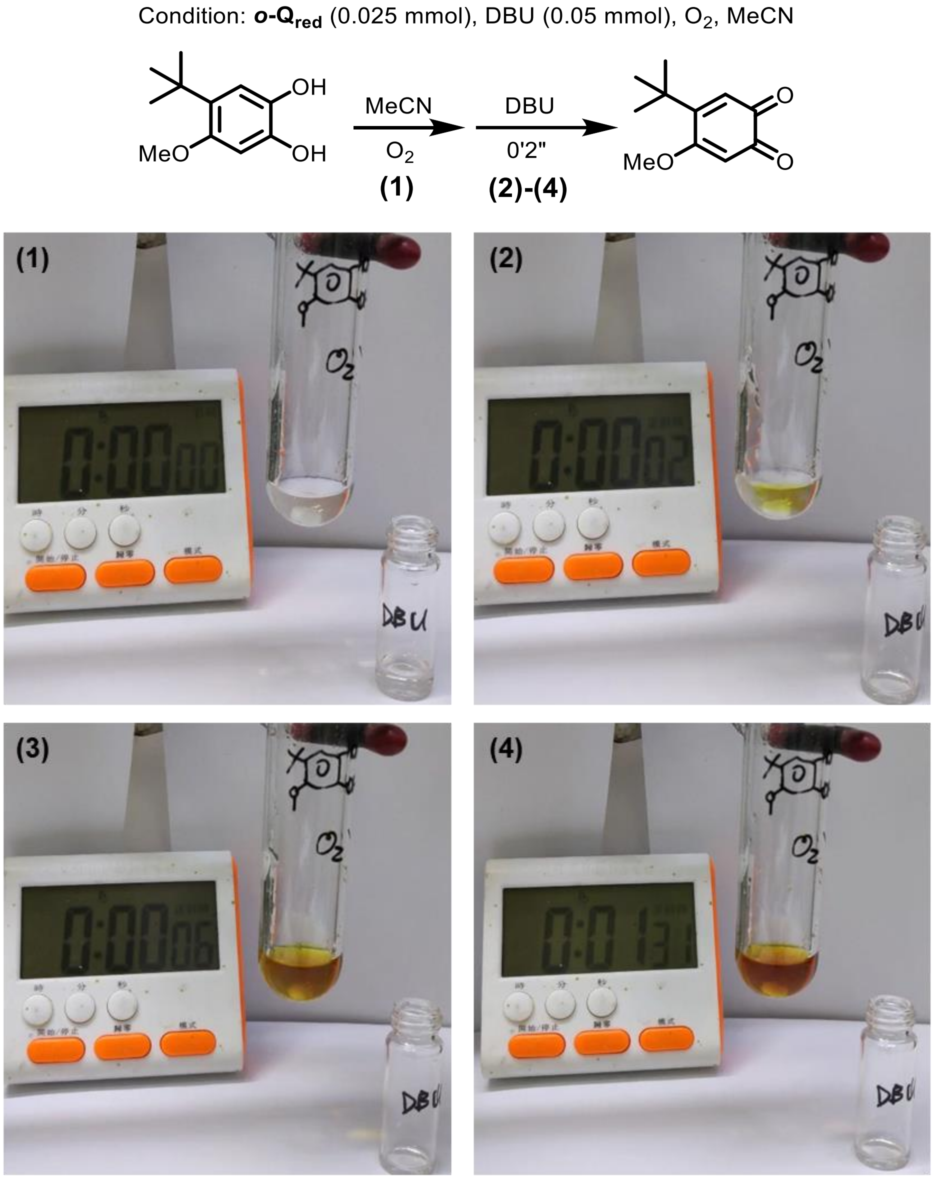

Supplementary Figure 9. Time course of reoxidation of catechol ***o*-Q$_{red}$** without lanthanum under basic condition.

Kinetic Isotope Effect Experiment. To a Schlenk tube sealed with rubber stopper, $La(OTf)_3$ (11.7mg, 0.02 mmol), ***o*-Q** (7.76 mg, 0.04 mmol), TBAI (14.7 mg, 0.04 mmol) and 1,3,5-trimethoxybenzene (11.2 mg, 0.06 mmol) in 0.4 mL of MeCN was added. The reaction tube was flushed with O_2. Then the solution of BnOH or $PhCD_2OH$ or BnOD (0.4 mmol) in 0.2 mL MeCN was added into the reaction tube. Taking 20 μL reaction solution for GC analysis every two minutes (<10% conversion). The initial rates of varied concentration of alcohol were measured, respectively, and every concentration was repeated for twice. The rate constant was determined by plotting initial rates vs concentrations. The KIE was determined to be 2.19 (C-H) and 1.40 (O-H) on the basis of the comparison of rate constant (k_H/k_D). $PhCD_2OH$ was prepared according to the literature[18], the deuterated ratio of C-H was more than 95%. BnOD was prepared by H/D exchange from excess D_2O and BnOH, the deuterated ratio of O-H was more than 80%. And $KIE_{O\text{-}H}$ (1.40) was already recalculated according to the 80% deuterated ratio of O-H.

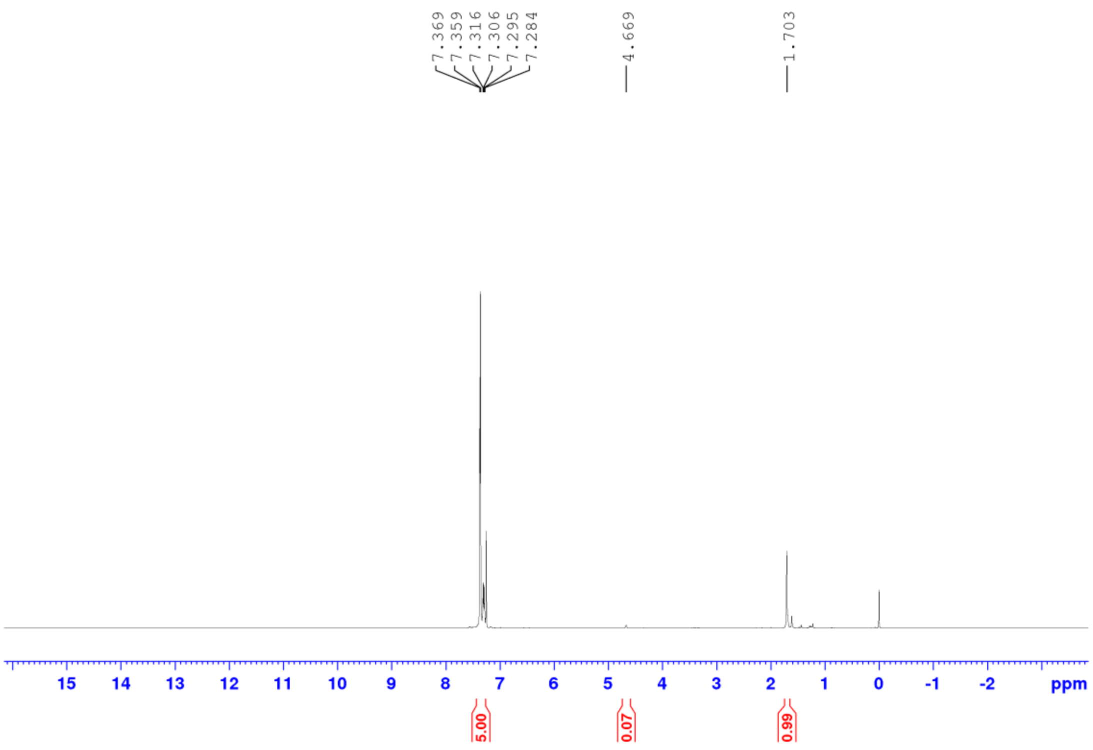

Supplementary Figure 10. ^1H NMR of $PhCD_2OH$ in $CDCl_3$.

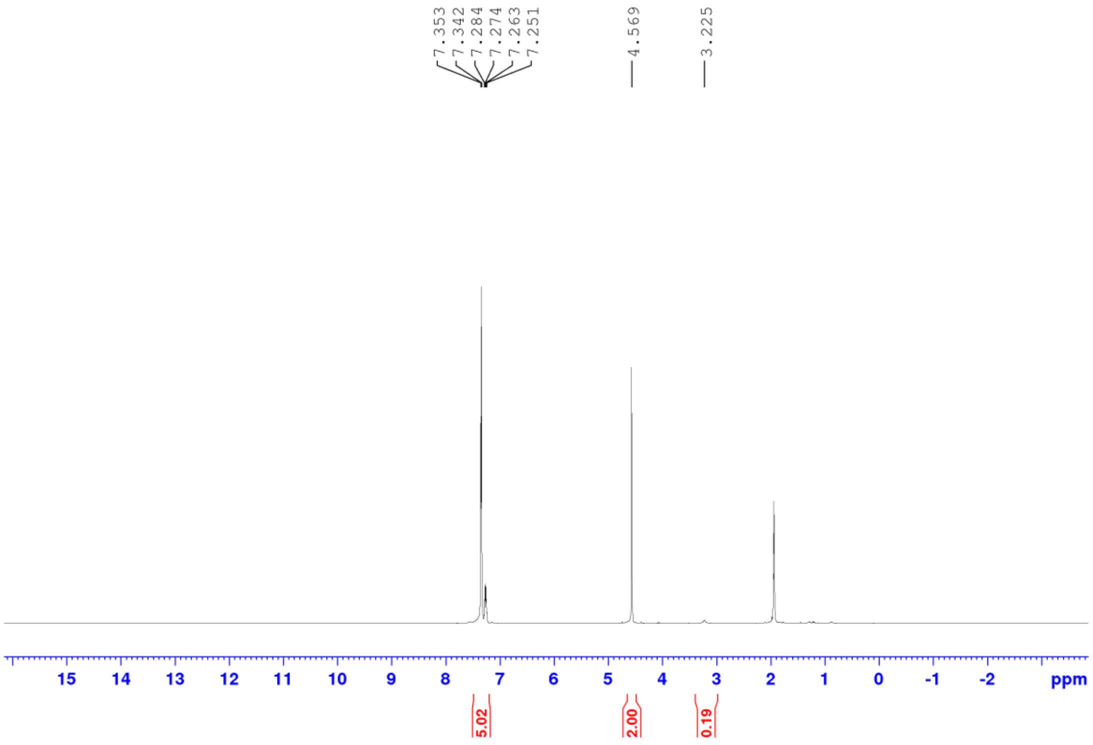

Supplementary Figure 11. ^{1}H NMR of BnOD in CD_3CN.

Supplementary Table 9. Kinetic data for KIE experiments.

$PhCH_2OH$		$PhCD_2OH$		$PhCH_2OD$	
c (M)	v_{obs} (M s^{-1})	c (M)	v_{obs} (M s^{-1})	c (M)	v_{obs} (M s^{-1})
0.50	0.000114	0.50	0.000066	0.5	0.000096
0.58	0.000125	0.67	0.000081	0.58	0.000122
0.67	0.000159	0.75	0.000100	0.67	0.000135
0.83	0.000189	0.83	0.000108	0.75	0.000154
0.92	0.000228	0.92	0.000115	0.92	0.000187

Hammett Correlations. To a Schlenk tube sealed with rubber stopper, $La(OTf)_3$ (11.7mg, 0.02 mmol), ***o*-Q** (7.76 mg, 0.04 mmol), TBAI (14.7 mg, 0.04 mmol) and 1,3,5-trimethoxybenzene (11.2 mg, 0.06 mmol) in 0.4 mL of MeCN was added. The reaction tube was flushed with O_2. Then the solution of *para*-substituted benzylalcohols (0.4 mmol) in 0.2 mL MeCN was added into the reaction tube. Taking 20 μL reaction solution for GC analysis every two minutes (<10% conversion). The initial rates of alcohol were measured, respectively, and every concentration was repeated for twice.

Supplementary Table 10. Kinetic data for Hammett plot.

Substituent	σ_p	v_{obs} (M s^{-1})	$\log v_X/v_H$
4-OH	-0.37	0.000366	0.33812
4-OMe	-0.268	0.000276	0.21560
4-Me	-0.17	0.000215	0.10713
4-H	0	0.000168	0
4-F	0.062	0.000138	-0.08543
4-Br	0.232	0.000113	-0.17416

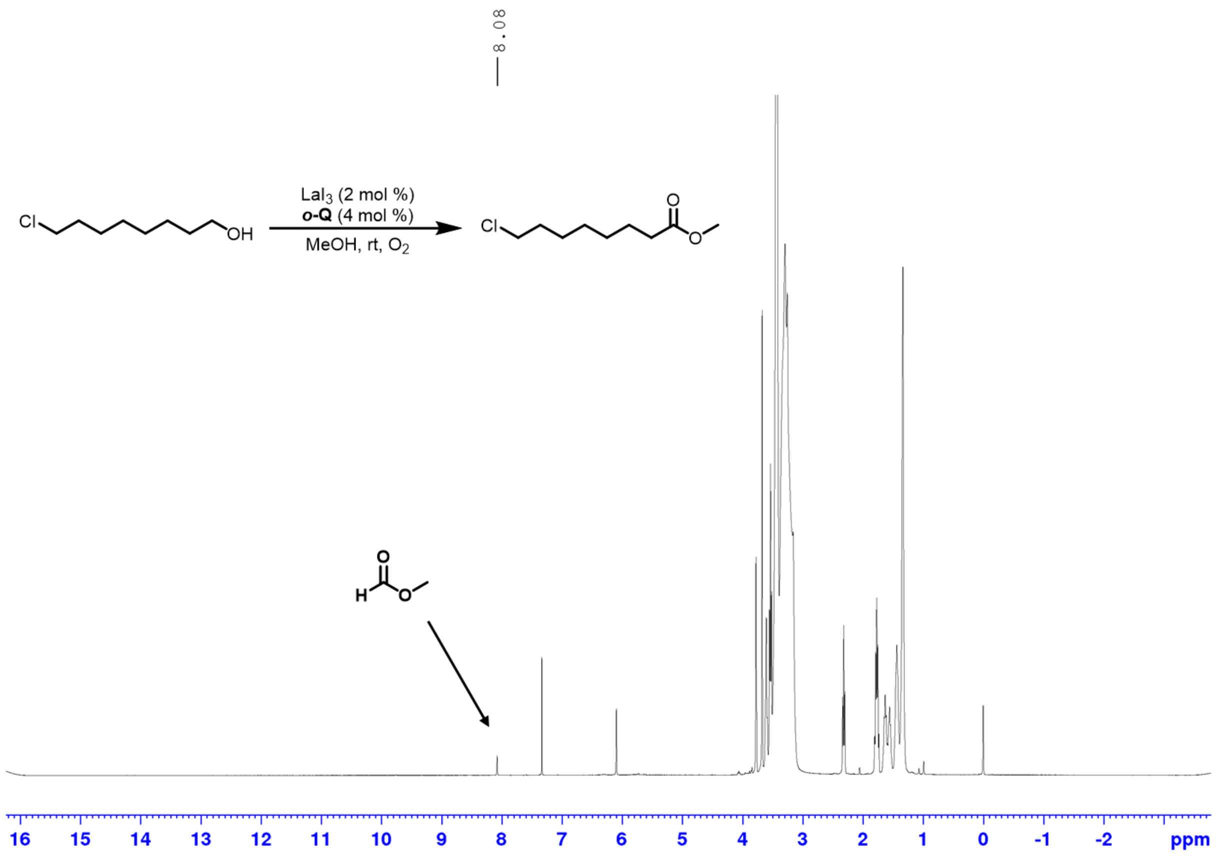

Supplementary Figure 12. NMR spectroscopy of the crude reaction mixture of aliphatic alcohol oxidation.

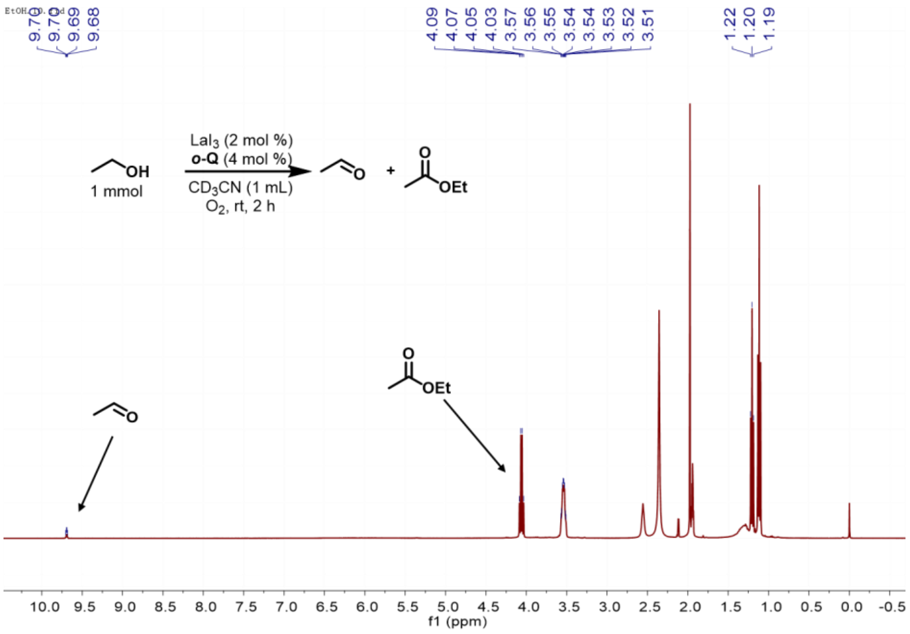

Supplementary Figure 13. NMR spectroscopy of the crude reaction mixture of ethanol oxidation.

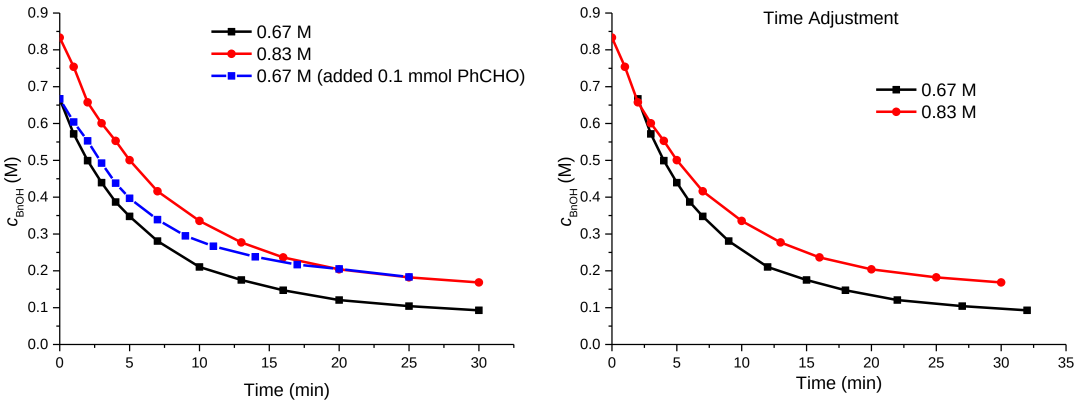

Supplementary Figure 14 Visual Kinetic analysis[a]

[a]General Condition: Benzylic alcohol **1a** (0.40 or 0.50 mmol), LaI_3 (0.004 mmol, 1 mol %), ***o*-Q1** (0.008 mmol, 2 mol %) and MeCN (0.6 mL), with O_2 balloon at room temperature, yields were determined by GC using 1,3,5-trimethoxybenzene as the internal standard.

The reaction became slower when benzaldehyde was added at the very beginning, suggesting a product-inhibition effect (Supplementary Figure 13, left). In addition, no overlay was observed by reaction progress kinetic analysis (right). This could be explained by the loss of lanthanum salt in the in-situ generated water during oxidation process due to its high aqueous solubility.

Computational Mechanism Investigation. We have investigated the key dehydrogenation step with methanol by DFT calculations. All of the geometry optimizations, vibrational frequency calculations were conducted using the Gaussian 09 program.[19] Images of minima and transition state geometries were generated using CYLview.[20] Spin density images were rendered using Multiwfn.[21] Geometries of ground state minima and transition states were optimized without constraints using the M06-2X functional[22] with the SDD(46) for La and 6-31G(d) for all other atoms in the SMD[23-24] solvation model of acetonitrile. Frequency calculations were performed on the resultant geometries to verify the nature of the optimized stationary points. Geometries with zero imaginary frequencies were deemed minima whereas those with exactly one imaginary frequency along the chemical path of interest were deemed transition states. The single point energy was calculated with def2-tzvpp[25-26] basis set using M06-2X functional with SMD solvation model. The PCET step activation barrier was calculated based on Marcus theory-based estimation, as illustrated below.

A) Marcus theory-based estimation of activation barrier for the pyridine-assisted proton-coupled electron transfer

The activation barrier for the proton coupled electron transfer (PCET) process were calculated using Marcus-Hush theory[27-34] in conjunction with the Savéant model[35-36]:

$$\Delta G^{\neq}_{PCET} = \Delta G^{\neq}_{0} \left(1 + \frac{\Delta G_r}{4\Delta G^{\neq}_{0}}\right) \qquad (1)$$

The intrinsic barrier, $\Delta G^{\neq}_{0}$ is estimated by calculating λ_{PCET}, the sum of internal and solvent reorganization energies:

$$\Delta G^{\neq}_{0} = \frac{\lambda_{PCET}}{4} = \frac{\lambda_i + \lambda_0}{4} = \frac{\lambda_i + (\lambda_0^{ET} + \lambda_0^{PT})}{4} \qquad (2)$$

The internal reorganization energy, λ_i, is calculated using the Savéant model as:

$$\lambda_i = \frac{\lambda_i^R + \lambda_i^P}{2} \qquad (3)$$

Where λ_i^R and λ_i^P are the difference between distorted and equilibrium geometries for reactants and products, respectively. The solvent reorganization energy, λ_0, is separated into two components, one related to electron transfer (4) and the other related to proton transfer (5):

$$\lambda_S^{ET} = \frac{e_0^2}{4\pi\varepsilon_0}\left(\frac{1}{\varepsilon_{op}} - \frac{1}{\varepsilon_s}\right)\frac{1}{2a} \qquad (4)$$

$$\lambda_S^{PT} = \frac{1}{4\pi\varepsilon_0}\left[\left(\frac{\varepsilon_S-1}{2\varepsilon_S+1}\right) - \left(\frac{\varepsilon_{op}-1}{2\varepsilon_{op}+1}\right)\right]\frac{(\mu_R-\mu_P)^2}{a^3} \qquad (5)$$

Where a is the radii of the H-Bonding complex. ε_{op} is the square of the refractive index and ε_s the dielectric constant; $\mu_R - \mu_P$ is the difference between reactant and product dipole moments, ε_0 is the permittivity of vacuum with the value of 8.8542 $\times$ 10^{-12} F·m^{-1}; e_0 is the elementary charge with its value is 1.6022 $\times$ 10^{-19} C.

λ_i^R	λ_i^P	λ_i	ε_{op}	ε_s	a	μ_R	μ_P	λ_s^{ET}	λ_s^{PT}
20.46	27.39	23.93	2.03	8.93	5.53	1.06	9.80	11.45	1.41

$$\Delta G_0^{\neq} = \frac{\lambda_i + (\lambda_0^{ET} + \lambda_0^{PT})}{4} = 10.17$$

$$\Delta G_{PCET}^{\neq} = \Delta G_0^{\neq}\left(1 + \frac{\Delta G_r}{4\Delta G_0^{\neq}}\right) = 10.17 \times \left(1 + \frac{12.25}{4 \times 10.17}\right)^2 = 13.2\ kcal/mol$$

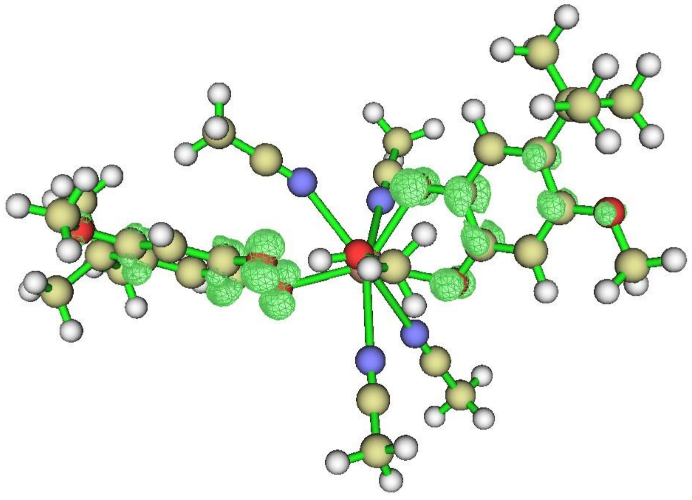

Supplementary Figure 15. The spin density of the $[La(SQ)_2]^+$ complex (Int-1).

Based on a known crystal structure of semiquinone-Lanthanum complex (CCDC: 1891306)[37], a possible structure for **Int-1** was generated (**Supplementary Figure 15**). We chose a 2:1 quinone/La ratio as this was experimentally determined as the optimal ratio. DFT calculations were

carried out to probe the key dehydrogenation step with methanol[38] as a model substrate (**Supplementary Figure 16**). As I_3^- showed only minor effect in the dehydrogenation, it was omitted in our calculation at this moment. The triplet semiquinone-La diradical species (**Int-1**) would acquire alcoholic O-H proton *via* a PCET or ECPT process leading to a high-lying **Int-3**. Hydride-transfer and subsequent aromatization then completes the dehydrogenation process. The hydride transfer was rate-limiting with an activation barrier of 28.9 kcal/mol, in consistence with the KIE and Hammett plot. In the key transition state **TS2**, the transferred proton (in red) still contacts closely with methanol O with a distance of 1.90 Å, accompanying the hydride transfer, a unique scenario of proton-coupled hydride transfer (PCHT). This may explain the observed primary KIEs for both C-H and O-H. It's also possible the dehydrogenation may proceed with the close-shell state of **Int-1** *via* a concerted PCHT. However, the close-shell state was disfavored by 16.5 kcal/mol, and we were unable to locate such a concerted process. The requirement of an open shell semiquinone anionic radical species could be understood by its ability in shuttling both proton and electron as well as its enhanced coordination capability toward lanthanum metal. In this regard, the SET activation opens a unique mechanistic scenario wherein two semiquinone anionic radicals are juxtaposed *via* lanthanum coordination for cooperative hydrogen transfer. The discrepancy in activation energy is possibly arisen from the neglect of anionic species or the involvement of a third quinone in coordination.

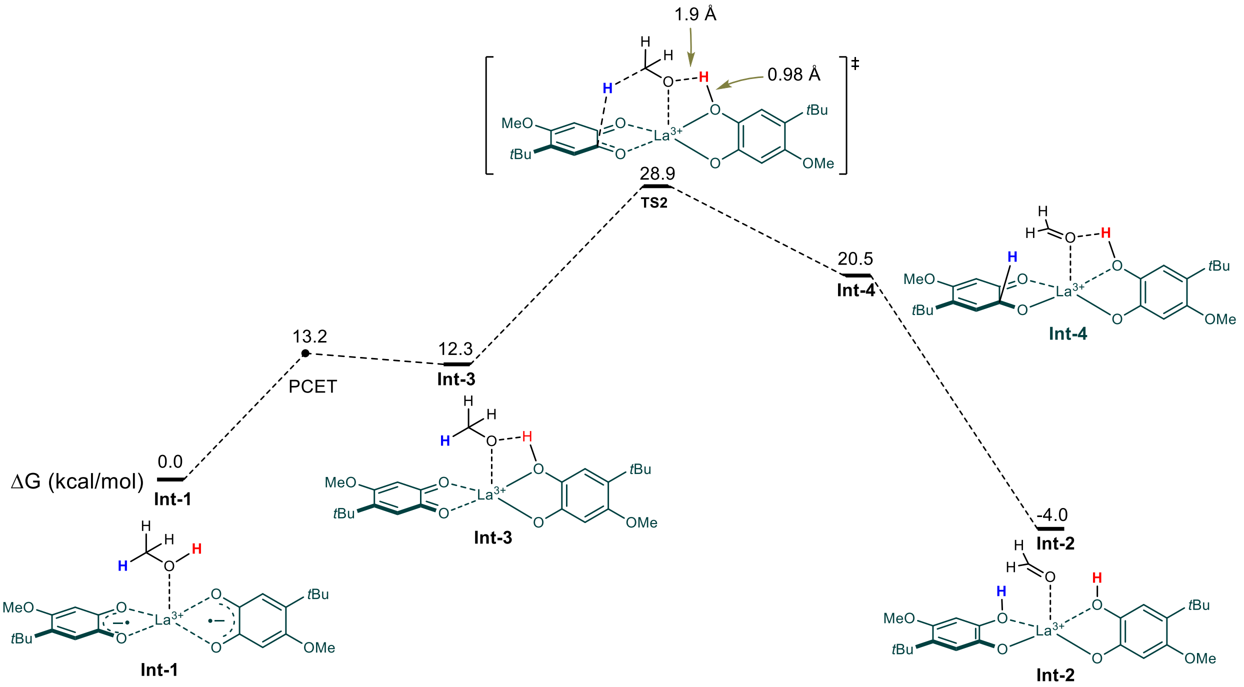

Supplementary Figure 16. The reaction profile for the proposed dehydrogenation process (Calculation method: M06-2X/def2-tzvpp//M06-2X/6-31G(d) (MWB46 for La)).

The energy and coordinate of the optimized structures:

(1) **Int1**-singlet

E(M062X) = -1984.931543

Zero-point correction= 0.714945 (Hartree/Particle)

Thermal correction to Energy= 0.770047

Thermal correction to Enthalpy= 0.770991

Thermal correction to Gibbs Free Energy= 0.619087

La	-0.03894400	-0.39416400	-0.21630200
O	-2.25734500	-0.97012700	-0.76597700
C	-3.23139700	-0.31737800	-0.12565000
C	-4.57070700	-0.35587700	-0.51772700
C	-5.60025700	0.35138200	0.13424700
C	-5.22388400	1.12713300	1.24669000
C	-3.88889100	1.18032700	1.67363900
C	-2.88153000	0.47484200	1.00690400
O	-1.60256100	0.52375500	1.36530600
O	2.42895500	-1.31113400	0.38891500
C	3.43695500	-0.60648100	0.29761600
C	4.72700300	-0.96539600	0.77959900
C	5.77810200	-0.10130700	0.63377000
C	5.67084300	1.24390000	-0.03416500
C	4.46167900	1.62151200	-0.49799700
C	3.28698800	0.77107100	-0.37239900
O	2.18174900	1.10466200	-0.77888500

H	-3.59696800	1.78172000	2.52771600
H	-4.79982200	-0.96748100	-1.38491800
H	4.29684700	2.57270300	-0.98828100
H	4.83656000	-1.92775600	1.26248900
C	6.90225600	2.13828300	-0.17409400
C	-7.05436400	0.28620800	-0.35831300
C	-7.20343100	-0.63437500	-1.57654200
H	-6.60858100	-0.28607600	-2.42792200
H	-6.90753900	-1.66441100	-1.34946900
H	-8.25384700	-0.64929600	-1.88789000
C	-7.53591900	1.68456300	-0.78489000
H	-8.57346500	1.63573800	-1.13851500
H	-7.48671000	2.39534500	0.04249000
H	-6.91971200	2.06676100	-1.60783900
C	-7.97568200	-0.26798700	0.74336700
H	-9.00770100	-0.32724500	0.37597200
H	-7.66355000	-1.27870600	1.03152000
H	-7.96244000	0.36287700	1.63402500
C	7.45262100	2.51426000	1.21640100
H	7.83003000	1.65179500	1.76619900
H	8.27634800	3.22367400	1.08400700
H	6.67910600	3.00439700	1.81734000
C	6.54764400	3.44543600	-0.89436900
H	7.45468900	4.05129500	-0.97747800
H	6.17124400	3.26529700	-1.90672300
H	5.80481800	4.02929200	-0.34104400
C	7.98272200	1.42843400	-1.01481800
H	7.58584700	1.15183600	-1.99759600
H	8.81508600	2.12299100	-1.17011000
H	8.37209400	0.53307200	-0.52945000
O	-6.21457100	1.83272600	1.89277000
O	6.99077500	-0.37516300	1.07132900
C	7.23434400	-1.62092200	1.73327400
H	6.62588700	-1.69008000	2.63881000
H	7.01535200	-2.45466900	1.06125200
H	8.29201400	-1.61033000	1.98887900
C	-5.84657100	2.63648700	2.99188200
H	-5.41467600	2.04055100	3.80510100
H	-5.13111900	3.41609400	2.70199100
H	-6.76549900	3.10820300	3.34418400
N	0.20252900	-2.90904900	-1.23015600
N	-0.18190400	-2.31186000	1.70642500
N	0.17472500	-0.32226800	-2.96705800
N	-1.09277300	1.92667700	-1.31810600
C	0.17459000	-0.26891300	-4.11989700
C	-0.37529200	-3.16040900	2.46437200

C	0.27654800	-3.97525700	-1.66503200
C	-2.12954100	2.43877900	-1.30308700
C	-3.43774700	3.07718300	-1.27549400
H	-4.20289600	2.30795400	-1.12554600
H	-3.47283500	3.79458100	-0.45224500
H	-3.61264200	3.59605400	-2.22088800
C	0.16998500	-0.19785700	-5.57361200
H	-0.33633800	-1.07748300	-5.97683000
H	-0.35727800	0.70533300	-5.88855700
H	1.19901700	-0.16750300	-5.93764900
C	-0.61757700	-4.23251900	3.41849000
H	0.17048700	-4.22765800	4.17487000
H	-1.58684300	-4.07839800	3.89760700
H	-0.61579500	-5.18968900	2.89274700
C	0.36943400	-5.32080500	-2.21179100
H	0.61632600	-6.02006900	-1.41014900
H	-0.58894400	-5.59624800	-2.65722700
H	1.14989700	-5.34662200	-2.97551000
C	1.76052200	0.15075300	3.04894200
H	1.67212200	-0.93275900	3.17977100
H	2.78187300	0.39677100	2.74956500
H	1.55003500	0.65318500	3.99690600
O	0.87889100	0.62239600	2.03323700
H	-0.03253800	0.72043700	2.38040600

(2) **Int1**-triplet

E(M062X) = -1984.957308

Zero-point correction= 0.714341 (Hartree/Particle)

Thermal correction to Energy= 0.769026

Thermal correction to Enthalpy= 0.769970

Thermal correction to Gibbs Free Energy= 0.618505

La	0.14574500	1.22605400	0.42424600
O	-2.11096000	0.81673600	-0.57937800
C	-3.13746200	0.06416900	-0.68877000
C	-4.35636300	0.34672600	-0.00203600
C	-5.48912200	-0.42953500	-0.09181400
C	-5.41749700	-1.59638900	-0.95455800
C	-4.26978600	-1.92298200	-1.63916400
C	-3.07901100	-1.13931900	-1.53365000
O	-2.01520900	-1.45717300	-2.13993000
O	2.43028400	1.36197100	-0.61071200
C	3.27187700	0.42891300	-0.37468600
C	4.51152600	0.30770000	-1.04727600
C	5.37084300	-0.73852700	-0.76819800
C	5.06000400	-1.75433700	0.22261000
C	3.85953200	-1.63567800	0.88108600

C	2.93008100	-0.58167100	0.62938100
O	1.81181500	-0.48566900	1.23783500
H	-4.21641900	-2.80018500	-2.27275300
H	-4.34308700	1.23672200	0.61761200
H	3.55901100	-2.35837600	1.63087400
H	4.75300100	1.05650800	-1.79189500
C	6.02372500	-2.91342000	0.50447100
C	-6.76653200	-0.08862100	0.68342700
C	-6.59110400	1.18354300	1.52191100
H	-5.79348900	1.07717800	2.26511000
H	-6.37020300	2.05622700	0.89812500
H	-7.52347700	1.38452600	2.05970800
C	-7.11966900	-1.23050600	1.65485100
H	-8.02868100	-0.97119000	2.21020000
H	-7.29303300	-2.17374100	1.13432300
H	-6.31279900	-1.37599000	2.38280700
C	-7.93356900	0.16449400	-0.28921800
H	-8.82577000	0.45281700	0.27866600
H	-7.69038900	0.98570800	-0.97299900
H	-8.17445900	-0.71958100	-0.88104800
C	6.22917400	-3.75666400	-0.76811400
H	6.69346200	-3.18137900	-1.57051600
H	6.87505800	-4.61217800	-0.53887000
H	5.26998400	-4.14547300	-1.12860000
C	5.46737700	-3.85009700	1.58443600
H	6.18610700	-4.65808700	1.75582400
H	5.31438500	-3.33142400	2.53679100
H	4.51874000	-4.30538700	1.28059500
C	7.37605500	-2.38084800	1.01398900
H	7.23849300	-1.80406900	1.93561300
H	8.03745000	-3.22486500	1.24162800
H	7.87140600	-1.74594300	0.27794000
O	-6.54727600	-2.33532300	-1.03518500
O	6.55180400	-0.88589900	-1.40208900
C	6.90136000	0.04753300	-2.41321100
H	6.16457900	0.04055200	-3.22300200
H	6.98711900	1.05879900	-2.00245700
H	7.86964500	-0.27612200	-2.79444300
C	-6.53433900	-3.49560000	-1.85088900
H	-6.32709400	-3.24109800	-2.89568300
H	-5.79070900	-4.21718600	-1.49572100
H	-7.53154300	-3.92814000	-1.77039700
N	1.04616600	3.89848200	0.42710700
N	0.06147700	2.34779000	-2.09207000
N	0.56108300	1.32473400	3.23833500
N	-1.18149600	-0.97309800	1.49694100

C	0.79603700	1.29446400	4.36791600
C	0.07698000	2.73491800	-3.17888600
C	1.45828100	4.97597100	0.39357300
C	-1.97523600	-1.80912800	1.56208500
C	-2.99133800	-2.85005500	1.62452400
H	-3.97875200	-2.38869600	1.53015000
H	-2.83749100	-3.55504900	0.80437000
H	-2.91942000	-3.37605300	2.57906400
C	1.09575000	1.25111400	5.79206100
H	0.82849300	2.20635100	6.24878400
H	0.51984200	0.44878100	6.25822500
H	2.16258800	1.06336300	5.93146400
C	0.10574100	3.21776900	-4.55259300
H	0.88557400	2.68814000	-5.10427800
H	-0.86302100	3.03579500	-5.02275900
H	0.31914000	4.28879800	-4.55632300
C	1.98008400	6.33439100	0.34717100
H	2.57422100	6.46270600	-0.56011900
H	1.14819300	7.04212500	0.34375500
H	2.60708800	6.51096100	1.22375700
C	1.17021000	-0.90443800	-2.34917200
H	1.52889700	0.00638300	-2.84135400
H	2.02827600	-1.44377800	-1.93524800
H	0.68265100	-1.54538000	-3.09090700
O	0.25665800	-0.59223500	-1.31033100
H	-0.68023900	-0.81609100	-1.61941900

(3) Int2

E(M062X) = -1984.938610

Zero-point correction= 0.713699 (Hartree/Particle)

Thermal correction to Energy= 0.768560

Thermal correction to Enthalpy= 0.769505

Thermal correction to Gibbs Free Energy= 0.619334

La	0.01474600	1.23031700	0.47250300
O	-2.16985600	0.92420500	-0.33276500
C	-3.19656400	0.11604800	-0.54955700
C	-4.45709500	0.34016200	0.02498400
C	-5.58515400	-0.46642300	-0.18526500
C	-5.41148700	-1.57832100	-1.03448300
C	-4.16760200	-1.84155000	-1.61589500
C	-3.06279500	-1.02223200	-1.37959800
O	-1.89562800	-1.33411500	-1.98110300
O	2.56205100	1.46610500	-0.45247400
C	3.37403800	0.54767200	-0.32911700
C	4.62645400	0.46337900	-1.00801100
C	5.43665000	-0.62181000	-0.81849000

C	5.09857700	-1.77044100	0.08973100
C	3.92754800	-1.72065700	0.75712900
C	3.00759400	-0.59654100	0.62392600
O	1.95523700	-0.52039900	1.24046400
H	-4.01784800	-2.69886400	-2.26257800
H	-4.53189800	1.21726200	0.66015400
H	3.60889900	-2.50649000	1.43058500
H	4.89479700	1.27231000	-1.67505400
C	6.05561900	-2.95463000	0.23328400
C	-6.93736800	-0.14827800	0.47073300
C	-6.86709700	1.11341400	1.34005500
H	-6.14356200	1.00780200	2.15587400
H	-6.59800800	1.99921600	0.75450100
H	-7.85053300	1.29495300	1.78759400
C	-7.37445400	-1.30747900	1.38412000
H	-8.34343200	-1.07859800	1.84473700
H	-7.46895900	-2.24279000	0.82879400
H	-6.64717400	-1.45469000	2.19168800
C	-8.00917500	0.09885400	-0.60634600
H	-8.96754900	0.34376900	-0.13244800
H	-7.72407900	0.94380600	-1.24405700
H	-8.15130800	-0.77855400	-1.24001600
C	6.23637000	-3.65899300	-1.12623100
H	6.72013500	-3.02218200	-1.86748000
H	6.85831900	-4.54838200	-0.97878800
H	5.26819100	-3.98566800	-1.52118400
C	5.49396400	-3.99285800	1.21327900
H	6.20487100	-4.82117200	1.28671500
H	5.35918900	-3.57709900	2.21734400
H	4.53766700	-4.40204700	0.87124800
C	7.41494500	-2.48502600	0.78851500
H	7.28320600	-1.97059900	1.74671900
H	8.04454500	-3.36424700	0.96196200
H	7.93991000	-1.81915500	0.10301900
O	-6.50228700	-2.38149400	-1.26394300
O	6.60033500	-0.75945200	-1.43053900
C	7.04696900	0.26545700	-2.32176800
H	6.34541000	0.37847100	-3.15263300
H	7.15449200	1.21165300	-1.78487500
H	8.01464900	-0.07085500	-2.68934700
C	-6.33509300	-3.50237700	-2.10568400
H	-6.03042400	-3.20765000	-3.11721900
H	-5.59752400	-4.20592500	-1.70010100
H	-7.30845300	-3.99301900	-2.15438700
N	0.92889900	3.93821100	0.56519300
N	0.17262400	2.45249400	-1.99003200

N	0.70090200	1.04798100	3.37218700
N	-1.17289300	-0.99282700	1.65807600
C	0.93579500	0.83695600	4.48257200
C	0.24806100	2.86374300	-3.06555000
C	1.33867800	5.01719700	0.57438800
C	-2.03618600	-1.76086000	1.64722400
C	-3.12728600	-2.72495100	1.62953700
H	-4.04860500	-2.21323600	1.33239900
H	-2.90193200	-3.51478200	0.90946300
H	-3.24845000	-3.15975100	2.62438100
C	1.23210800	0.56386400	5.88260000
H	0.95899300	1.43041200	6.48851300
H	0.65964600	-0.30651400	6.21062900
H	2.29934000	0.36132200	5.99546500
C	0.34802100	3.38004800	-4.42361900
H	1.12935300	2.83701300	-4.95979200
H	-0.60772800	3.24423200	-4.93445300
H	0.59779300	4.44270400	-4.39088800
C	1.85704600	6.37802500	0.58190000
H	2.46837900	6.53734000	-0.30894200
H	1.02324900	7.08349700	0.58454000
H	2.46613800	6.52846300	1.47580500
C	1.26328000	-0.85884200	-2.05848500
H	1.60483600	-0.03166200	-2.70610000
H	2.17799300	-1.32055600	-1.62928900
H	0.82435300	-1.62937700	-2.71463700
O	0.37002800	-0.44974500	-1.08936500
H	-1.10932600	-0.85554400	-1.56597900

(4) TS2

E(M062X) = -1984.912710

Zero-point correction= 0.710716 (Hartree/Particle)

Thermal correction to Energy= 0.763534

Thermal correction to Enthalpy= 0.764478

Thermal correction to Gibbs Free Energy= 0.619906

La	0.03890600	1.05346700	0.51082400
O	-2.03802200	0.78236700	-0.59898500
C	-3.16684800	0.10184100	-0.72504000
C	-4.34212600	0.39964100	-0.02211500
C	-5.54643900	-0.30667700	-0.16658100
C	-5.55062800	-1.37307100	-1.08950400
C	-4.38971500	-1.71512700	-1.79285400
C	-3.21047100	-1.00498800	-1.59911800
O	-2.08720600	-1.37843200	-2.26310400
O	2.57095800	1.56578200	-0.14325100
C	3.36552500	0.60822600	-0.18072800

C	4.73788100	0.70976100	-0.55136100
C	5.55081100	-0.39653100	-0.50710100
C	5.08852900	-1.74710800	-0.10192000
C	3.78192400	-1.87738700	0.20404600
C	2.80224100	-0.76797400	0.09231400
O	1.69679600	-0.80318900	0.75835400
H	-4.37233300	-2.54810700	-2.48652000
H	-4.28660400	1.23870200	0.66420800
H	3.35579700	-2.82461900	0.51525900
H	5.11791000	1.68732000	-0.82029200
C	6.06443800	-2.92945100	-0.04924900
C	-6.79412600	0.05926900	0.65260600
C	-6.52884400	1.23487000	1.60141400
H	-5.73581700	1.00574800	2.32181500
H	-6.24927500	2.14483900	1.05951000
H	-7.44154300	1.45053500	2.16763400
C	-7.22527400	-1.13561600	1.52226900
H	-8.11534700	-0.87283200	2.10712200
H	-7.45858800	-2.01225300	0.91493800
H	-6.42864500	-1.40241700	2.22716900
C	-7.94859400	0.47220800	-0.27780100
H	-8.82881900	0.74635600	0.31640800
H	-7.66434000	1.34414300	-0.87837200
H	-8.22802400	-0.33662100	-0.95565100
C	6.63143700	-3.21318100	-1.45323100
H	7.22203400	-2.38116300	-1.83949500
H	7.27593700	-4.09833600	-1.40968300
H	5.81987700	-3.42213900	-2.15932900
C	5.34711400	-4.20304800	0.41528100
H	6.07031000	-5.02415400	0.44214200
H	4.92759700	-4.09218400	1.42095500
H	4.54089600	-4.48921200	-0.26840100
C	7.20585400	-2.65858200	0.94740800
H	6.80392400	-2.46810000	1.94886300
H	7.84922900	-3.54371800	1.00569300
H	7.82300200	-1.80855600	0.65320000
O	-6.72767100	-2.05737000	-1.26730100
O	6.83952200	-0.33918600	-0.82912100
C	7.40194800	0.90341300	-1.24781600
H	6.89470300	1.26877500	-2.14519200
H	7.33390700	1.64601500	-0.44763200
H	8.44643600	0.69037400	-1.46989800
C	-6.71436600	-3.18716000	-2.11408200
H	-6.46622800	-2.91652400	-3.14754700
H	-6.00506800	-3.94598500	-1.76145200
H	-7.72471800	-3.59801900	-2.08587000

N	0.77710700	3.85416700	0.71697300
N	0.33297100	2.45972100	-1.86474500
N	0.47368700	0.61301300	3.32026200
N	-1.22477000	-1.26029700	1.30451200
C	0.71125700	0.20608100	4.37406500
C	0.42827300	3.07590100	-2.83563000
C	1.17471200	4.92799600	0.86153600
C	-2.09018100	-2.02460500	1.26636700
C	-3.18692000	-2.98062000	1.21529200
H	-4.12298300	-2.43678500	1.05083000
H	-3.02270700	-3.67654300	0.38947300
H	-3.23793500	-3.53241700	2.15656700
C	1.01391400	-0.31685100	5.69900200
H	0.92215200	0.48287800	6.43700100
H	0.31321700	-1.11946000	5.93883900
H	2.03364100	-0.70775700	5.70818100
C	0.55325700	3.85842500	-4.05795400
H	1.34473600	3.43507800	-4.68008800
H	-0.39259100	3.83354200	-4.60310300
H	0.80148000	4.89106300	-3.80317600
C	1.68247200	6.28127900	1.04197000
H	2.14097300	6.62513600	0.11249100
H	0.85881700	6.94572700	1.31152700
H	2.42886500	6.28276100	1.83954400
C	1.50988100	-0.61991400	-2.15957600
H	2.53960400	-0.92943700	-1.19564500
H	1.62867900	-1.55666800	-2.72276300
H	2.05191500	0.25149800	-2.56033800
O	0.45917000	-0.44316600	-1.47917700
H	-1.34467400	-0.84088300	-1.91565400

(5) Int3

E(M062X) = -1984.930484

Zero-point correction= 0.713901 (Hartree/Particle)

Thermal correction to Energy= 0.768451

Thermal correction to Enthalpy= 0.769396

Thermal correction to Gibbs Free Energy= 0.620489

La	0.08201200	1.03074300	0.45367900
O	-1.98716700	0.78870300	-0.69606400
C	-3.12349900	0.11206200	-0.77688100
C	-4.28945600	0.43235400	-0.07061400
C	-5.48625600	-0.29686200	-0.16094400
C	-5.49041500	-1.41016100	-1.02654600
C	-4.34137400	-1.76456500	-1.74515200
C	-3.17468800	-1.02245800	-1.61308100
O	-2.05530200	-1.38277200	-2.29409800

O	2.47735700	1.44474000	-0.57102700
C	3.19433300	0.43121800	-0.68138000
C	4.56688400	0.48327100	-1.08266200
C	5.36738900	-0.61745300	-0.90994600
C	4.89106500	-1.88659000	-0.30846200
C	3.56855000	-1.97299400	-0.07344000
C	2.56604500	-0.92984600	-0.47695400
O	1.41988400	-0.88853000	0.26503200
H	-4.32814700	-2.62317300	-2.40673700
H	-4.23279300	1.30058700	0.57824700
H	3.12797800	-2.86400000	0.36209100
H	4.96717400	1.43383800	-1.41275100
C	5.87696200	-3.00981500	0.03969000
C	-6.72690100	0.09648500	0.65658100
C	-6.47030400	1.33666300	1.52198700
H	-5.66359200	1.17123800	2.24457400
H	-6.21552900	2.21340100	0.91664500
H	-7.37896300	1.57355000	2.08626100
C	-7.11967000	-1.04758700	1.60877700
H	-8.01195900	-0.76908600	2.18289300
H	-7.33444900	-1.96942700	1.06457600
H	-6.31096800	-1.24476300	2.32277600
C	-7.90392700	0.42842600	-0.27804200
H	-8.77845600	0.72744400	0.31260200
H	-7.64403600	1.26327100	-0.93931600
H	-8.18184100	-0.42709500	-0.89643800
C	6.53123400	-3.57323200	-1.23529400
H	7.13305600	-2.82677900	-1.75616500
H	7.18318300	-4.41342700	-0.96955700
H	5.76716000	-3.94679600	-1.92639500
C	5.15060500	-4.17044400	0.73086700
H	5.88111400	-4.94633000	0.98174500
H	4.66614700	-3.85097000	1.65987800
H	4.39295900	-4.62258600	0.08216300
C	6.95629200	-2.50214200	1.01308700
H	6.49570300	-2.11398400	1.92873100
H	7.61371900	-3.33315400	1.29293600
H	7.57233100	-1.71513400	0.57472800
O	-6.65552000	-2.12916200	-1.13388500
O	6.65761300	-0.61755600	-1.24155100
C	7.22794800	0.55585600	-1.81564900
H	6.70321700	0.83061200	-2.73532200
H	7.19534000	1.38625300	-1.10432700
H	8.26275300	0.30049800	-2.03960400
C	-6.64498500	-3.28886100	-1.93924400
H	-6.43588300	-3.05136300	-2.98919300

H	-5.90855700	-4.01963500	-1.58272100
H	-7.64443400	-3.71967600	-1.86257700
N	1.42322600	3.38600700	1.47036000
N	0.14547800	3.12996800	-1.40153900
N	0.69866500	0.31837200	3.33449900
N	-1.35492100	-1.16003100	1.39941800
C	1.01088700	-0.13651600	4.34870100
C	0.13281800	3.97982300	-2.18223300
C	1.99305000	4.31902000	1.84051200
C	-2.20360000	-1.94333900	1.40471900
C	-3.28220600	-2.92087200	1.40751800
H	-4.21407400	-2.41811300	1.12990900
H	-3.06366600	-3.70852400	0.68292300
H	-3.37975000	-3.35626800	2.40461300
C	1.40635500	-0.71458700	5.62629700
H	1.36885100	0.05419900	6.40116100
H	0.72402900	-1.52731900	5.88389800
H	2.42388400	-1.10360300	5.54814700
C	0.12054300	5.04669400	-3.17327500
H	0.63966500	4.70963700	-4.07326600
H	-0.91286900	5.29926700	-3.42021600
H	0.62565800	5.92558200	-2.76711500
C	2.71292000	5.49682500	2.30543000
H	3.23847100	5.95616000	1.46560000
H	2.00377800	6.21174500	2.72821700
H	3.43404000	5.20167400	3.07060900
C	1.00586300	0.63015200	-3.11621900
H	2.35916400	-1.20814500	-1.54667000
H	0.86728000	0.17326500	-4.10547400
H	1.77932200	1.40308500	-3.00469000
O	0.31839600	0.28740600	-2.17431200
H	-1.38467200	-0.69430400	-2.10788800

(6) Int4

E(M062X) = -1984.965826

Zero-point correction= 0.713958 (Hartree/Particle)

Thermal correction to Energy= 0.768670

Thermal correction to Enthalpy= 0.769614

Thermal correction to Gibbs Free Energy= 0.620689

La	0.05234600	1.12226800	0.61464200
O	-1.95858900	0.71812200	-0.62198400
C	-3.09175500	0.04556900	-0.74774100
C	-4.28749400	0.37636800	-0.09640200
C	-5.48889100	-0.33533700	-0.25018700
C	-5.46602700	-1.44604500	-1.11877100
C	-4.28720000	-1.81527100	-1.77789600

C	-3.11843800	-1.09100400	-1.58478200
O	-1.97397400	-1.47491700	-2.20699200
O	2.38909000	1.42205900	-0.05723400
C	3.28130300	0.46106900	-0.11592400
C	4.59256900	0.63775900	-0.59535100
C	5.50707900	-0.41850300	-0.63488000
C	5.16162800	-1.72059700	-0.20635700
C	3.85017000	-1.88984600	0.25993400
C	2.94910700	-0.84139200	0.29967900
O	1.65757100	-0.95382500	0.79041400
H	-4.25098800	-2.67505200	-2.43716900
H	-4.25411200	1.24671200	0.55128800
H	3.51603400	-2.86123500	0.61237400
H	4.86933400	1.63156000	-0.92796400
C	6.16312500	-2.88376600	-0.22753600
C	-6.76722000	0.07788600	0.49600300
C	-6.53944900	1.31014500	1.38027900
H	-5.78006000	1.12718600	2.14856400
H	-6.23412900	2.18438400	0.79507900
H	-7.47530900	1.56163400	1.89126500
C	-7.23548300	-1.06232000	1.41796000
H	-8.14972700	-0.76615100	1.94683800
H	-7.44266000	-1.97542300	0.85653300
H	-6.46986600	-1.28317500	2.17146000
C	-7.88164900	0.43402300	-0.50454500
H	-8.78261900	0.74989000	0.03528900
H	-7.56757000	1.26351800	-1.14879100
H	-8.14043200	-0.41558200	-1.13903300
C	6.64511600	-3.15884400	-1.66318800
H	7.14284000	-2.28864300	-2.09508400
H	7.35180400	-3.99755100	-1.66506100
H	5.80004500	-3.42921500	-2.30689500
C	5.53233600	-4.18032800	0.29617100
H	6.27926600	-4.98062500	0.26036200
H	5.19828000	-4.08318900	1.33521500
H	4.67753200	-4.49460900	-0.31294000
C	7.36886900	-2.56602500	0.67554200
H	7.04106500	-2.40111600	1.70851800
H	8.07020800	-3.40928200	0.67448200
H	7.90161700	-1.67551100	0.33661800
O	-6.63475900	-2.14525700	-1.29195600
O	6.78415400	-0.23918700	-1.09276600
C	7.17524700	1.05671800	-1.49999000
H	6.57869300	1.40948900	-2.34934800
H	7.09497300	1.77843400	-0.67867900
H	8.21927800	0.97425900	-1.80519600

C	-6.60408700	-3.29292000	-2.11430800
H	-6.33564000	-3.04346200	-3.14799200
H	-5.90221100	-4.04404200	-1.73151800
H	-7.61461500	-3.70388500	-2.09719300
N	0.94717000	3.79874000	1.13266700
N	0.05790800	2.78280300	-1.61462400
N	0.43842600	0.15596800	3.29927200
N	-1.07803300	-1.39710700	1.11923700
C	0.56816800	-0.29213800	4.35498900
C	0.05369800	3.40770000	-2.58475800
C	1.38278600	4.85950100	1.26297900
C	-2.00491000	-2.08376800	1.19112600
C	-3.17848700	-2.93968800	1.28631400
H	-4.07199000	-2.33622500	1.09523200
H	-3.11171500	-3.73324200	0.53834900
H	-3.23205300	-3.37804500	2.28536200
C	0.73005600	-0.86291100	5.68497800
H	0.34680600	-0.16156200	6.42920100
H	0.17405900	-1.80093700	5.74634200
H	1.78920500	-1.05255500	5.87186300
C	0.05136400	4.19185800	-3.81173200
H	0.94160400	3.95010500	-4.39668700
H	-0.84333600	3.95528000	-4.39173700
H	0.05580700	5.25522600	-3.56310300
C	1.93668900	6.19708400	1.42059500
H	2.60462300	6.41326100	0.58405200
H	1.12422300	6.92686800	1.43768600
H	2.49551500	6.25031000	2.35734100
C	1.43068800	0.03302700	-2.49610600
H	2.11678800	0.88042500	-2.37511600
H	1.37865200	-1.87863300	0.90561800
H	1.54142800	-0.61791800	-3.37327500
O	0.55049300	-0.17884400	-1.68100500
H	-1.27801900	-0.85701300	-1.90700900

3. Supplementary Methods

For active primary alcohol:

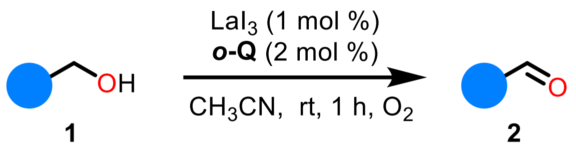

A flame-dried 10 mL flask was flushed with O_2 and equipped with an O_2 balloon. LaI_3 (5.19 mg, 0.01 mmol) were added to the solution of ***o*-Q** (3.88 mg, 0.02 mmol) and alcohol (1.0 mmol) in 1.0 mL of CH_3CN. The reaction was stirred at room temperature for 1 h. After the reaction was completed, the crude reaction product was purified though a silica gel using 1:10-1:5 EtOAc/petro ether to give a pure product. For some volatile aldehyde, yields were determined by ^1H NMR.

For aliphatic primary alcohol:

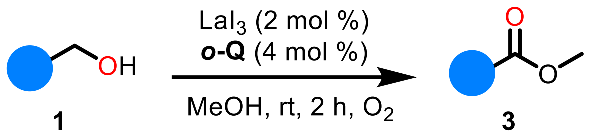

A flame-dried 10 mL flask was flushed with O_2 and equipped with an O_2 balloon. LaI_3 (10.4 mg, 0.02 mmol) were added to the solution of ***o*-Q** (7.76 mg, 0.04 mmol) and alcohol (1.0 mmol) in 1.0 mL of MeOH. The reaction was stirred at room temperature for 2 h. After the reaction was completed, the crude reaction product was purified though a silica gel using 1:10-1:5 EtOAc/petro ether to give a pure product.

For aliphatic primary diol:

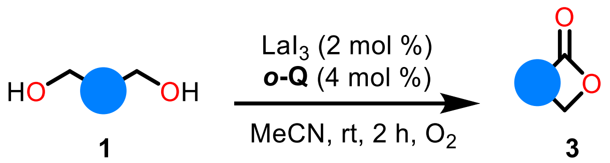

A flame-dried 10 mL flask was flushed with O_2 and equipped with an O_2 balloon. LaI_3 (10.4 mg, 0.02 mmol) were added to the solution of ***o*-Q** (7.76 mg, 0.04 mmol) and alcohol (1.0 mmol) in 1.0 mL of MeCN. The reaction was stirred at room temperature for 2 h. After the reaction was completed, the crude reaction product was purified though a silica gel using 1:10-1:5 EtOAc/petro ether to give a pure product.

For secondary alcohol:

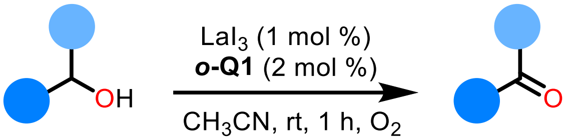

1 4

A flame-dried 10 mL flask was flushed with O_2 and equipped with an O_2 balloon. LaI_3 (5.19 mg, 0.01 mmol) were added to the solution of ***o*-Q1** (3.88 mg, 0.02 mmol) and alcohol (1.0 mmol) in 1.0 mL of MeCN. The reaction was stirred at room temperature for 1 h. After the reaction was completed, the crude reaction product was purified though a silica gel using 1:10-1:5 EtOAc/petro ether to give a pure product. For some volatile ketone, after reaction completed, 2 equiv. 2,4-dinitrophenylhydrazine, 1 mL 4 M HCl and 1 mL THF were added, which stirred at room temperature for further 2 h. The crude reaction product was purified through a silica gel using 1:5 EtOAc/petro ether to give a hydrazone product.

For large scale reaction, substrate (10 mmol), LaI_3 (0.01 mmol) and ***o*-Q** (0.02 mmol) in MeCN (1.0 mL) was stirred at room temperature for 1 h. TON was determined by ^{1}H NMR by the ratio of product and LaI_3.

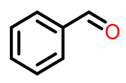

2a Colorless liquid (100 mg, 95%). Characterization was in consistent with previous literature[39]. ^{1}H NMR (400 MHz, $CDCl_3$) δ 9.99 (s, 1H), 7.87-7.85 (m, 2H), 7.63-7.58 (m, 1H), 7.52-7.48 (m, 2H). ^{13}C NMR (126 MHz, $CDCl_3$) δ 192.3, 136.4, 134.4, 129.7, 128.9.

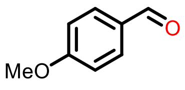

2b Colorless liquid (134 mg, 99%). Characterization was in consistent with previous literature[39]. ^{1}H NMR (500 MHz, $CDCl_3$) δ 9.88 (s, 1H), 7.84 (d, J = 8.7 Hz, 2H), 7.01 (d, J = 8.7 Hz, 2H), 3.88 (s, 3H). ^{13}C NMR (126 MHz, $CDCl_3$) δ 190.8, 164.7, 132.0, 130.0, 114.4, 55.6.

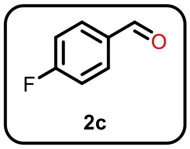
Colorless liquid (111 mg, 90%). Reaction condition: LaI_3 2%, ***o*-Q** 4%. Characterization was in consistent with previous literature[40]. ^{1}H NMR (400 MHz, $CDCl_3$) δ 9.97 (s, 1H), 7.94 (m, 2H), 7.24 (m, 2H). ^{13}C NMR (100 MHz, $CDCl_3$) δ 190.5, 167.8 (d, J = 256.3 Hz), 133.0 (d, J = 2.3 Hz), 132.3 (d, J = 9.7 Hz), 116.5 (d, J = 22.3 Hz).

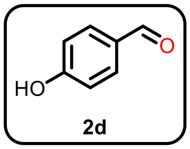
Grey solid (99 mg, 81%). Characterization was in consistent with previous literature[41]. ^{1}H NMR (500 MHz, $CDCl_3$) δ 9.87 (s, 1H), 7.83 (d, J = 8.6 Hz, 2H), 6.99 (d, J = 8.6 Hz, 2H), 6.31 (br, 1H). ^{13}C NMR (126 MHz, $CDCl_3$) δ 191.1, 161.7, 132.7, 130.0, 116.2.

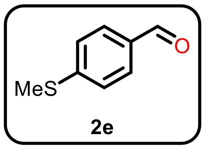
Yellow liquid (146 mg, 96%). Characterization was in consistent with previous literature[42]. ^{1}H NMR (400 MHz, $CDCl_3$) δ 9.89 (s, 1H), 7.73 (dd, J = 5.0, 8.4 Hz, 2H), 7.27 (d, J = 8.4 Hz, 2H), 2.50 (s, 3H). ^{13}C NMR (101 MHz, $CDCl_3$) δ 191.1, 147.8, 132.8, 129.8, 125.1, 14.5.

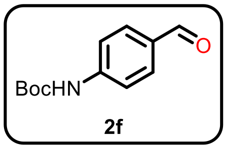
White solid (201 mg, 91%). Reaction condition: LaI_3 2%, ***o*-Q** 4%. Characterization was in consistent with previous literature[43]. ^{1}H NMR (400 MHz, $CDCl_3$) δ 9.89 (s, 1H), 7.81 (d, J = 8.6 Hz, 2H), 7.58 (d, J = 8.6 Hz, 2H), 7.40 (br, 1H), 1.52 (s, 9H). ^{13}C NMR (101 MHz, $CDCl_3$) δ 191.3, 152.3, 144.6, 131.3, 131.2, 117.9, 81.4, 28.3.

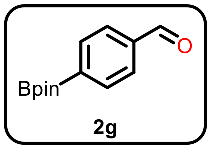
White solid (169 mg, 73%). Reaction condition: LaI_3 2%, ***o*-Q** 4%. Characterization was in consistent with previous literature[41]. ^{1}H NMR (400 MHz, $CDCl_3$) δ 10.04

(s, 1H), 7.96 (d, *J* = 7.9 Hz, 2H), 7.85 (d, *J* = 8.0 Hz, 2H), 1.36 (s, 12H). ^{13}C NMR (101 MHz, $CDCl_3$) δ 192.7, 138.2, 135.3, 128.8, 84.4, 25.0.

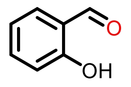

2h

Colorless liquid (73 mg, 60%). Reaction condition: $La(OTf)_3$ 5%, ***o*-Q** 10%, TBAI 15%, 2 h. Characterization was in consistent with previous literature[44]. ^{1}H NMR (400 MHz, $CDCl_3$) δ 11.02 (s, 1H), 9.89 (s, 1H), 7.57 (m, 2H), 7.04 (m, 2H). ^{13}C NMR (101 MHz, $CDCl_3$) δ 196.7, 161.7, 137.1, 133.8, 120.8, 119.9, 117.7.

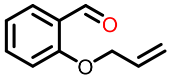

2i

Colorless liquid (142 mg, 88%). Reaction condition: LaI_3 4%, ***o*-Q** 8%. Characterization was in consistent with previous literature[45]. ^{1}H NMR (400 MHz, $CDCl_3$) δ 10.54 (s, 1H), 7.85 (dd, *J* = 1.8, 7.7 Hz, 1H), 7.54 (m, 1H), 7.04 (m, 2H), 6.12 (m, 1H), 5.48 (m, 1H), 5.34 (m, 1H), 4.67 (m, 2H). ^{13}C NMR (101 MHz, $CDCl_3$) δ 189.8, 161.0, 135.9, 132.5, 128.5, 125.2, 120.9, 118.1, 113.0, 69.3.

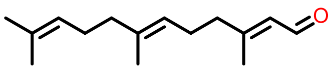

2j

Colorless liquid (198 mg, 90%). Characterization was in consistent with previous literature[46]. ^{1}H NMR (400 MHz, $CDCl_3$) δ 10.00 (d, *J* = 8.0 Hz, 1H), 5.89 (dd, *J* = 0.8, 8.0 Hz, 1H), 5.10 (m, 2H), 2.26 (m, 4H), 2.17 (m, 3H), 2.09 (m, 2H), 2.00 (m, 2H), 1.67 (s, 3H), 1.61 (s, 3H), 1.59 (s, 3H). ^{13}C NMR (101 MHz, $CDCl_3$) δ 191.1, 163.7, 136.5, 131.4, 127.4, 124.2, 122.5, 40.6, 39.6, 26.6, 25.7, 17.7, 17.6, 16.0.

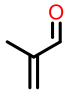

2k

Colorless liquid (NMR yield: 89%). Reaction condition: LaI_3 4%, ***o*-Q** 8%. Characterization was in consistent with previous literature[47]. ^{1}H NMR (400 MHz, $CDCl_3$) δ 9.56

(s, 1H), 6.30 (m, 1H), 6.00 (m, 1H), 1.85 (m, 3H). ^{13}C NMR (101 MHz, $CDCl_3$) δ 194.6, 146.0, 134.3, 13.8.

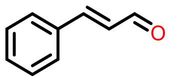
2l

Yellow oil (125 mg, 95%). Characterization was in consistent with previous literature[39]. ^{1}H NMR (400 MHz, $CDCl_3$) δ 9.71 (d, J = 7.7 Hz, 1H), 7.46 (m, 2H), 7.39 (d, J = 2.1 Hz, 1H), 7.57 (m, 2H), 7.46 (m, 4H), 6.74 (dd, J = 7.7, 16.0 Hz, 1H). ^{13}C NMR (101 MHz, $CDCl_3$) δ 193.8, 152.9, 134.1, 131.4, 129.2, 128.7, 128.6.

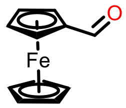

2m

Red solid (193 mg, 90%). Reaction condition: La(OTf)$_3$ 5%, ***o*-Q** 10%, TBAI 15%, 2 h. Characterization was in consistent with previous literature[48]. ^{1}H NMR (400 MHz, $CDCl_3$) δ 9.95 (s, 1H), 4.78 (s, 2H), 4.60 (s, 2H), 4.26 (s, 5H). ^{13}C NMR (101 MHz, $CDCl_3$) δ 193.5, 79.4, 73.2, 69.7.

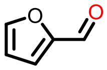
2n

Colorless liquid (70 mg, 87%). Reaction condition: LaI_3 4%, ***o*-Q** 8%. Characterization was in consistent with previous literature[39]. ^{1}H NMR (400 MHz, $CDCl_3$) δ 9.67 (s, 1H), 7.70 (m, 1H), 7.26 (m, 1H), 6.62 (m, 1H). ^{13}C NMR (101 MHz, $CDCl_3$) δ 177.8, 152.8, 148.1, 121.2, 112.5.

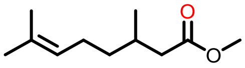
3a

Colorless liquid (112 mg, 61%). Reaction condition: 2 h. Characterization was in consistent with previous literature[49]. ^{1}H NMR (400 MHz, $CDCl_3$) δ 5.11 (t, J = 7.1 Hz, 1H), 3.66 (s, 3H), 2.34 (dd, J = 5.9, 14.7 Hz, 1H), 2.15 (m, 1H), 2.00 (m, 3H), 1.68

(s, 3H), 1.60 (s, 3H), 1.39 (m, 2H), 0.95 (d, J = 6.6 Hz, 3H). ^{13}C NMR (101 MHz, $CDCl_3$) δ 173.8, 131.6, 124.4, 51.4, 41.7, 36.9, 30.1, 25.8, 25.5, 19.7, 17.7.

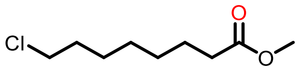

3b

Colorless liquid (134 mg, 70%). Reaction condition: 2 h. Characterization was in consistent with previous literature[50]. ^{1}H NMR (400 MHz, $CDCl_3$) δ 3.66 (s, 3H), 3.54 (t, J = 6.7 Hz, 2H), 2.32 (t, J = 7.5 Hz, 2H), 1.80 (m, 2H), 1.64 (m, 2H), 1.45 (m, 2H), 1.35 (m, 4H). ^{13}C NMR (101 MHz, $CDCl_3$) δ 174.2, 51.5, 45.1, 34.1, 32.6, 29.0, 28.6, 26.8, 24.9.

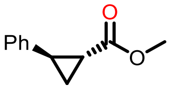

3c

Colorless liquid (150 mg, 85%). Reaction condition: 2 h. Characterization was in consistent with previous literature[51]. ^{1}H NMR (400 MHz, $CDCl_3$) δ 7.29 (m, 2H), 7.21 (m, 1H), 7.10 (m, 2H), 3.71 (s, 3H), 2.54 (m, 1H), 1.92 (m, 1H), 1.62 (m, 1H), 1.34 (m, 1H). ^{13}C NMR (100 MHz, $CDCl_3$) δ 173.9, 140.1, 128.6, 126.6, 126.3, 52.0, 26.4, 24.0, 17.1.

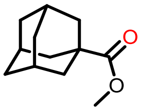

3d

Colorless liquid (105 mg, 54%). Reaction condition: 2 h. Characterization was in consistent with previous literature[50]. ^{1}H NMR (400 MHz, $CDCl_3$) δ 3.65 (s, 3H), 2.01 (m, 3H), 1.89 (m, 6H), 1.75 (s, 6H). ^{13}C NMR (101 MHz, $CDCl_3$) δ 178.3, 51.6, 40.8, 39.0, 36.6, 28.0.

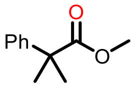

3e

Colorless liquid (77 mg, 43%). Reaction condition: 2 h. Characterization was in consistent with previous literature[52]. ^{1}H NMR (400 MHz, $CDCl_3$) δ 7.33 (m, 4H), 7.25 (m, 1H), 3.64 (s, 3H), 1.58 (s, 6H). ^{13}C NMR (101 MHz, $CDCl_3$) δ 177.4, 144.8, 128.5, 126.8, 125.7, 52.3, 46.6, 26.7.

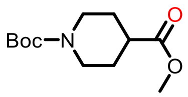

3f Yellow oil (177 mg, 73%). Reaction condition: 2 h. Characterization was in consistent with previous literature[53]. ^{1}H NMR (400 MHz, $CDCl_3$) δ 4.00 (d, *J* = 10.6 Hz, 2H), 3.69 (s, 3H), 2.83 (t, *J* = 11.7 Hz, 2H), 2.44 (m, 1H), 1.85 (m, 2H), 1.57 (m, 2H), 1.45 (s, 9H). ^{13}C NMR (101 MHz, $CDCl_3$) δ 178.3, 51.6, 40.8, 39.0, 36.6, 28.0.

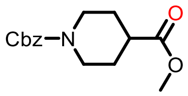

3g Yellow oil (169 mg, 61%). Reaction condition: 2 h. Characterization was in consistent with previous literature[54]. ^{1}H NMR (400 MHz, $CDCl_3$) δ 7.35 (m, 5H), 5.12 (s, 2H), 4.07 (m, 2H), 3.67 (s, 3H), 2.93 (t, *J* = 11.7 Hz, 2H), 2.47 (m, 1H), 1.90 (m, 2H), 1.63 (m, 2H). ^{13}C NMR (101 MHz, $CDCl_3$) δ 174.8, 155.2, 136.8, 128.5, 128.0, 127.9, 67.1, 51.8, 43.3, 40.8, 27.9.

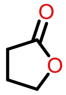

3h Colorless liquid (NMR yield, 84%). Reaction condition: 2 h. Characterization was in consistent with previous literature[55]. ^{1}H NMR (400 MHz, $CDCl_3$) δ 4.31 (m, 2H), 2.49 (m, 2H), 2.24 (m, 2H). ^{13}C NMR (101 MHz, $CDCl_3$) δ 177.8, 68.5, 27.7, 22.1.

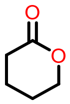

3i Colorless liquid (NMR yield, 80%). Reaction condition: 2 h. Characterization was in consistent with previous literature[55]. ^{1}H NMR (400 MHz, $CDCl_3$) δ 4.35 (t, *J* = 5.6 Hz, 2H), 2.56 (t, *J* = 6.8 Hz, 2H), 1.88 (m, 4H). ^{13}C NMR (101 MHz, $CDCl_3$) δ 171.5, 69.5, 30.0, 22.5, 19.2.

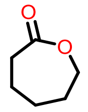

3j

Colorless liquid (NMR yield, 36%). Reaction condition: 2 h. Characterization was in consistent with previous literature[55]. ^{1}H NMR (400 MHz, $CDCl_3$) δ 4.24 (m, 2H), 2.64 (m, 2H), 1.86 (m, 2H), 1.77 (m, 4H). ^{13}C NMR (101 MHz, $CDCl_3$) δ 176.1, 69.2, 34.4, 29.1, 28.7, 22.8.

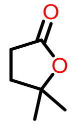

3k

Colorless liquid (NMR yield, 73%). Reaction condition: 2 h. Characterization was in consistent with previous literature[56]. ^{1}H NMR (400 MHz, $CDCl_3$) δ 2.62 (t, J = 8.2 Hz, 2H), 2.06 (t, J = 8.2 Hz, 2H), 1.43 (s, 6H). ^{13}C NMR (101 MHz, $CDCl_3$) δ 176.8, 84.7, 34.8, 29.5, 27.9.

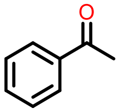

4aa

Colorless liquid (119 mg, 99%). Characterization was in consistent with previous literature[57]. ^{1}H NMR (400 MHz, $CDCl_3$) δ 7.96 (m, 2H), 7.58 (m, 1H), 7.47 (m, 2H), 2.60 (s, 3H). ^{13}C NMR (101 MHz, $CDCl_3$) δ 198.2, 137.2, 133.2, 128.6, 128.6, 26.7.

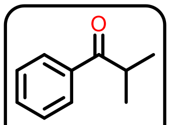

4ab

Colorless liquid (147 mg, 99%). Characterization was in consistent with previous literature[58]. ^{1}H NMR (400 MHz, $CDCl_3$) δ 7.96 (m, 2H), 7.46 (m, 3H), 3.55 (m, 1H), 1.20 (d, J = 6.8 Hz, 6H). ^{13}C NMR (101 MHz, $CDCl_3$) δ 204.5, 136.2, 132.8, 128.6, 128.3, 35.4, 19.2.

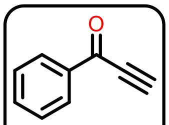

4ac

Colorless oil (100 mg, 77%). Reaction condition: La(OTf)$_3$ 5%, ***o*-Q** 10%, TBAI 15%, 2 h. Characterization was in consistent with previous literature[59]. ^{1}H NMR (400 MHz, $CDCl_3$)

δ 8.17 (m, 2H), 7.65 (m, 1H), 7.52 (m, 2H), 3.46 (s, 1H). ^{13}C NMR (101 MHz, $CDCl_3$) δ 177.5, 136.2, 134.6, 129.8, 128.8, 80.9, 80.4.

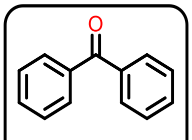
4ad

White solid (162 mg, 89%). Reaction condition: $La(OTf)_3$ 5%, ***o*-Q** 10%, TBAI 15%, 2 h. Characterization was in consistent with previous literature[40]. ^{1}H NMR (300 MHz, $CDCl_3$) δ 7.81 (d, *J* = 7.7 Hz, 4H), 7.60 (m, 2H), 7.49 (m, 4H). ^{13}C NMR (75 MHz, $CDCl_3$) δ 196.7, 137.6, 132.5, 130.1, 128.3.

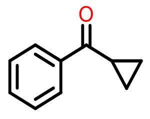
4ae

Colorless liquid (140 mg, 87%). Characterization was in consistent with previous literature[60]. ^{1}H NMR (400 MHz, $CDCl_3$) δ 8.02 (d, *J* = 7.5 Hz, 2H), 7.56 (m, 1H), 7.47 (m, 2H), 7.38 (t, *J* = 7.4 Hz, 1H), 2.70 (m, 1H), 1.26 (m, 2H), 1.05 (m, 2H). ^{13}C NMR (101 MHz, $CDCl_3$) δ 200.6, 138.1, 132.8, 128.5, 128.1, 17.2, 11.7.

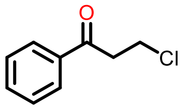
4af

Colorless oil (130 mg, 87%). Characterization was in consistent with previous literature[58]. ^{1}H NMR (400 MHz, $CDCl_3$) δ 7.96 (m, 2H), 7.60 (m, 1H), 7.49 (m, 2H), 3.93 (t, *J* = 6.8 Hz, 2H), 3.47 (t, *J* = 6.8 Hz, 2H). ^{13}C NMR (101 MHz, $CDCl_3$) δ 196.8, 136.4, 133.6, 128.8, 128.1, 41.3, 38.8.

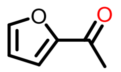
4b

Yellow oil (66 mg, 60%). Reaction condition: $La(OTf)_3$ 5%, ***o*-Q** 10%, TBAI 15%, 2 h. Characterization was in consistent with previous literature[61]. ^{1}H NMR (400 MHz, $CDCl_3$) δ

7.59 (s, 1H), 7.19 (d, *J* = 3.5 Hz, 1H), 6.55 (q, *J* = 1.9, 4.8 Hz, 1H), 2.48 (s, 3H). ^{13}C NMR (101 MHz, $CDCl_3$) δ 186.9, 153.0, 146.5, 117.3, 112.3, 26.1.

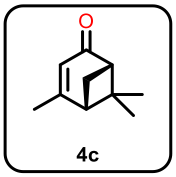

Colorless liquid (135 mg, 90%). Characterization was in consistent with previous literature[62]. ^{1}H NMR (400 MHz, $CDCl_3$) δ 5.73 (m, 1H), 2.84 (m, 1H), 2.66 (m, 1H), 2.44 (m, 1H), 2.10 (d, *J* = 9.2 Hz, 1H), 2.02 (m, 3H), 1.50 (s, 3H), 1.01 (s, 3H). ^{13}C NMR (101 MHz, $CDCl_3$) δ 204.0, 170.2, 121.1, 57.5, 54.0, 49.6, 40.8, 26.5, 23.6, 22.0.

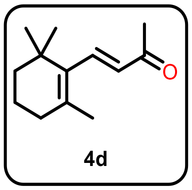

Colorless liquid (190 mg, 99%). Characterization was in consistent with previous literature[63]. ^{1}H NMR (400 MHz, $CDCl_3$) δ 7.25 (d, *J* = 16.0 Hz, 1H), 6.10 (d, *J* = 16.4 Hz, 1H), 2.30 (s, 3H), 2.07 (t, *J* = 6.0 Hz, 2H), 1.76 (s, 3H), 1.62 (m, 2H), 1.48 (m, 2H), 1.07 (s, 6H). ^{13}C NMR (101 MHz, $CDCl_3$) δ 198.8, 143.2, 136.1, 136.0, 131.7, 39.8, 34.1, 33.6, 28.9, 27.2, 21.8, 19.0.

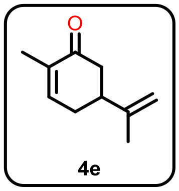

Colorless liquid (147 mg, 98%). Characterization was in consistent with previous literature[46]. ^{1}H NMR (400 MHz, $CDCl_3$) δ 6.77 (m, 1H), 4.81 (d, *J* = 19.6 Hz, 7H), 2.72 (m, 1H), 2.61 (m, 1H), 2.47 (m, 1H), 2.38 (m, 2H), 1.79 (s, 3H), 1.76 (s, 3H). ^{13}C NMR (101 MHz, $CDCl_3$) δ 199.9, 146.7, 144.7, 135.5, 110.5, 43.2, 42.5, 31.3, 20.6, 15.8.

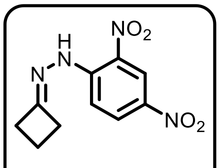

4fa

Red solid (248 mg, 99%). Characterization was in consistent with previous literature[64]. ^{1}H NMR (400 MHz, $CDCl_3$) δ 10.64 (s, 1H), 9.02 (d, J = 2.6 Hz, 1H), 8.23 (dd, J = 2.4, 9.6 Hz, 1H), 7.79 (d, J = 9.6 Hz, 1H), 3.16 (m, 4H), 2.25 (m, 2H). ^{13}C NMR (101 MHz, $CDCl_3$) δ 161.8, 144.7, 137.4, 129.8, 128.6, 123.4, 115.9, 34.0, 31.7, 13.9.

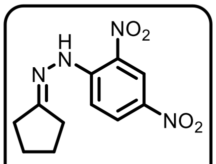

4fb

Red solid (251 mg, 95%). Characterization was in consistent with previous literature[65]. ^{1}H NMR (400 MHz, $CDCl_3$) δ 10.78 (s, 1H), 9.09 (d, J = 2.5 Hz, 1H), 8.28 (dd, J = 2.5, 9.6 Hz, 1H), 7.90 (d, J = 9.6 Hz, 1H), 2.60 (t, J = 7.3 Hz, 2H), 2.49 (t, J = 7.3 Hz, 2H), 2.03 (m, 2H), 1.92 (m, 2H). ^{13}C NMR (101 MHz, $CDCl_3$) δ 168.6, 145.1, 137.6, 130.0, 128.9, 123.7, 116.3, 33.7, 28.2, 25.0, 24.9.

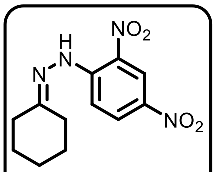

4fc

Red solid (272 mg, 98%). Characterization was in consistent with previous literature[65]. ^{1}H NMR (400 MHz, $CDCl_3$) δ 11.19 (s, 1H), 9.11 (d, J = 2.5 Hz, 1H), 8.29 (dd, J = 2.5, 9.6 Hz, 1H), 7.97 (d, J = 9.6 Hz, 1H), 2.65 (m, 4H), 1.87 (m, 8H). ^{13}C NMR (101 MHz, $CDCl_3$) δ 164.3, 145.2, 137.7, 130.0, 129.1, 123.6, 116.5, 37.3, 30.9, 30.4, 30.3, 27.7, 24.4.

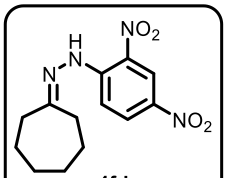

4fd

Red solid (286 mg, 98%). Characterization was in consistent with previous literature[65]. ^{1}H NMR (400 MHz, $CDCl_3$) δ 11.01 (s, 1H), 9.10 (d, J = 2.5 Hz, 1H), 8.28 (dd, J =

2.3, 9.6 Hz, 1H), 7.97 (d, *J* = 9.6 Hz, 1H), 2.50 (m, 4H), 1.80 (m, 6H). ^{13}C NMR (101 MHz, $CDCl_3$) δ 161.6, 145.5, 137.6, 130.0, 128.9, 123.7, 116.4, 35.7, 27.3, 27.2, 26.1, 25.6.

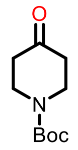

4ga

White solid (191 mg, 96%). Reaction condition: LaI_3 2%, ***o*-Q** 4%. Characterization was in consistent with previous literature[66]. ^{1}H NMR (400 MHz, $CDCl_3$) δ 3.72 (t, *J* = 6.2 Hz, 4H), 2.44 (t, *J* = 6.2 Hz, 2H), 1.50 (s, 9H). ^{13}C NMR (101 MHz, $CDCl_3$) δ 207.9, 154.6, 80.6, 43.1, 41.3, 28.5.

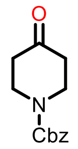

4gb

White solid (224 mg, 96%). Reaction condition: LaI_3 4%, ***o*-Q** 8%. Characterization was in consistent with previous literature[66]. ^{1}H NMR (400 MHz, $CDCl_3$) δ 7.35 (m, 5H), 5.18 (s, 2H), 3.79 (t, *J* = 6.2 Hz, 4H), 2.45 (m, 4H). ^{13}C NMR (101 MHz, $CDCl_3$) δ 207.2, 155.2, 136.4, 128.7, 128.4, 128.1, 67.7, 43.2, 41.1.

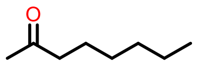

4h

Colorless liquid (105 mg, 82%). Reaction condition: LaI_3 2%, ***o*-Q** 4%. Characterization was in consistent with previous literature[42]. ^{1}H NMR (400 MHz, $CDCl_3$) δ 2.44 (t, J = 7.5 Hz, 2H), 2.13 (s, 3H), 1.60 (m, 2H), 1.30 (m, 6H), 0.90 (t, *J* = 6.8 Hz, 3H). ^{13}C NMR (101 MHz, $CDCl_3$) δ 209.4, 43.9, 31.7, 29.9, 29.0, 23.9, 22.6, 14.1.

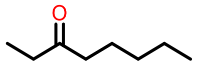

4i

Colorless liquid (123 mg, 96%). Reaction condition: LaI_3 2%, ***o*-Q** 4%. Characterization was in consistent with previous literature[67]. ^{1}H NMR (400 MHz, $CDCl_3$) δ 2.44

(m, 4H), 1.61 (m, 2H), 1.34 (m, 4H), 1.07 (t, J = 7.3 Hz, 3H), 0.90 (t, J = 7.0 Hz, 3H). ^{13}C NMR (101 MHz, $CDCl_3$) δ 211.9, 42.4, 35.9, 31.5, 23.7, 22.5, 13.9, 7.9.

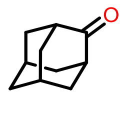

4j

White solid (146 mg, 97%). Reaction condition: LaI_3 2%, ***o*-Q** 4%. Characterization was in consistent with previous literature[42]. ^{1}H NMR (400 MHz, $CDCl_3$) δ 2.54 (m, 2H), 2.10 (m, 12H). ^{13}C NMR (101 MHz, $CDCl_3$) δ 218.4, 47.0, 39.3, 36.4, 27.5.

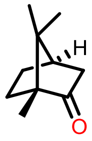

4k

White solid (143 mg, 94%). Reaction condition: LaI_3 2%, ***o*-Q** 4%. Characterization was in consistent with previous literature[42]. ^{1}H NMR (400 MHz, $CDCl_3$) δ 2.38 (dt, J = 10.4, 18.2 Hz, 1H), 2.10 (t, J = 4.5 Hz, 1H), 1.99 (m, 1H), 1.87 (d, J = 18.2 Hz, 1H), 1.74 (m, 1H), 1.44 (m, 2H), 0.96 (d, J = 18.8 Hz, 6H), 0.84 (s, 3H). ^{13}C NMR (101 MHz, $CDCl_3$) δ 219.8, 57.8, 46.9, 43.4, 43.2, 30.7, 27.2, 19.9, 19.3, 9.4.

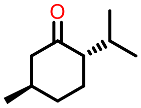

4l

Colorless liquid (140 mg, 91%). Reaction condition: LaI_3 2%, ***o*-Q** 4%. Characterization was in consistent with previous literature[42]. ^{1}H NMR (400 MHz, $CDCl_3$) δ 2.38 (m, 1H), 2.16 (m, 1H), 2.08 (m, 3H), 1.92 (m, 2H), 1.41 (m, 2H), 1.01 (d, J = 6.3 Hz, 3H), 0.92 (d, J = 6.8 Hz, 3H), 0.86 (d, J = 6.8 Hz, 3H). ^{13}C NMR (101 MHz, $CDCl_3$) δ 212.6, 56.0, 51.0, 35.6, 34.1, 28.0, 26.0, 22.4, 21.4, 18.8.

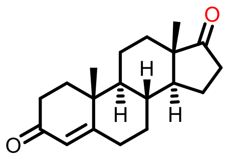

4m

White solid (125 mg, 87%). Reaction condition: 0.5 mmol scale, LaI_3 2%, ***o*-Q** 4%, DCM/MeCN 1:1, 1 ml. Characterization was in consistent with previous literature[68]. ^{1}H NMR (400 MHz, $CDCl_3$) δ 5.75 (s, 1H), 2.32 (m, 5H), 1.95 (m, 4H), 1.84 (m, 1H), 1.67 (m, 3H), 1.52 (m, 1H), 1.41 (m, 1H), 1.26 (m, 2H), 1.22 (s, 3H), 1.07 (m, 1H), 0.97 (m, 1H), 0.92 (s, 3H). ^{13}C NMR (101 MHz, $CDCl_3$) δ 220.3, 199.3, 170.4, 124.2, 53.9, 50.9, 47.5, 38.7, 35.8, 35.7, 35.2, 33.9, 32.6, 31.3, 30.8, 21.8, 20.4, 17.4, 13.7

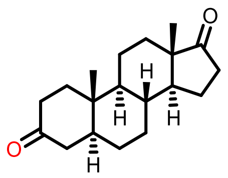

4n

White solid (137 mg, 95%). Reaction condition: 0.5 mmol scale, LaI_3 2%, ***o*-Q** 4%, DCM/MeCN 1:1, 1 ml. Characterization was in consistent with previous literature[69]. ^{1}H NMR (400 MHz, $CDCl_3$) δ 2.20 (m, 4H), 1.96 (m, 3H), 1.87 (m, 1H), 1.76 (m, 2H), 1.48 (m, 4H), 1.21 (m, 6H), 0.95 (m, 4H), 0.73 (m, 4H). ^{13}C NMR (101 MHz, $CDCl_3$) δ 220.8, 211.4, 53.9, 51.2, 47.7, 46.6, 44.6, 38.4, 38.0, 35.8, 34.9, 31.5, 30.5, 28.6, 21.8, 20.7, 13.8, 11.4.

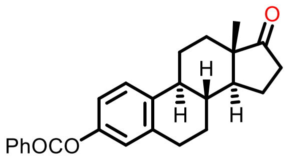

4oa

White solid (113 mg, 87%). Reaction condition: 0.5 mmol scale, LaI_3 2%, ***o*-Q** 4%, DCM/MeCN 1:1, 1 ml. Characterization was in consistent with previous literature [70]. ^{1}H NMR (400 MHz, $CDCl_3$) δ 8.18 (m, 2H), 7.62 (m, 1H), 7.50 (m, 2H), 7.32 (d, J = 8.5 Hz, 1H), 6.95 (m, 2H), 2.92 (m, 2H), 2.40 (m, 1H), 2.29 (m, 1H), 1.97 (m, 4H), 1.44 (m, 6H), 0.92 (s, 6H). ^{13}C NMR (101 MHz, $CDCl_3$) δ 220.8, 165.5, 149.0, 138.2, 137.6, 133.6, 130.2, 129.8, 128.6, 126.6, 121.8, 119.0, 50.6, 48.1, 44.3, 38.2, 36.0, 31.7, 29.5, 26.5, 25.9, 21.7, 14.0.

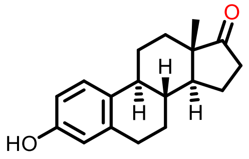
4ob

White solid (112 mg, 88%). Reaction condition: 0.5 mmol scale, LaI_3 2%, ***o*-Q** 4%, DCM/MeCN 1:1, 1 ml. Characterization was in consistent with previous literature[71]. ^{1}H NMR (400 MHz, DMSO-d$_6$) δ 9.00 (s, 1H), 7.02 (d, *J* = 8.5 Hz, 1H), 6.50 (m, 1H), 6.45 (m, 1H), 2.73 (m, 2H), 2.40 (dd, *J* = 8.4, 18.8 Hz, 1H), 2.29 (m, 1H), 1.87 (m, 4H), 1.72 (m, 1H), 1.43 (m, 3H), 1.26 (m, 3H). ^{13}C NMR (101 MHz, DMSO-d$_6$) δ 220.1, 155.5, 137.6, 130.4, 126.5, 115.4, 113.3, 50.1, 47.8, 43.9, 38.5, 35.8, 31.8, 29.5, 26.6, 26.0, 21.6, 14.0.

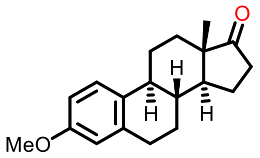
4oc

White solid (136 mg, 96%). Reaction condition: 0.5 mmol scale, LaI_3 2%, ***o*-Q** 4%, DCM/MeCN 1:1, 1 ml. Characterization was in consistent with previous literature [71]. ^{1}H NMR (400 MHz, $CDCl_3$) δ 7.17 (d, *J* = 8.6 Hz, 1H), 6.69 (dd, *J* = 2.7, 8.6 Hz, 1H), 6.62 (d, *J*= 2.5 Hz, 1H), 3.76 (s, 3H), 2.87 (m, 2H), 2.45 (dd, *J* = 8.7, 18.7 Hz, 1H), 2.35 (m, 1H), 2.19 (m, 1H), 1.92 (m, 4H), 1.40 (m, 6H), 0.89 (s, 3H). ^{13}C NMR (101 MHz, $CDCl_3$) δ 220.7, 157.6, 137.7, 132.0, 126.3, 113.9, 111.6, 55.1, 50.4, 48.0, 44.0, 38.4, 35.8, 31.6, 29.7, 26.5, 25.9, 21.6, 13.8.

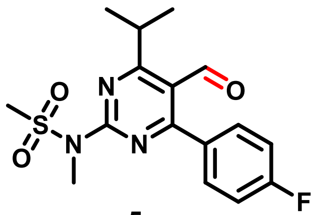
5a

White solid (42 mg, 60%). Reaction condition: 0.2 mmol scale, LaI_3 5%, ***o*-Q** 10%, MeCN 0.4 ml, 12 h. Characterization was in consistent with previous literature[72]. ^{1}H NMR (500 MHz, $CDCl_3$) δ 9.97 (s, 1H), 7.62 (m, 2H), 7.21 (m, 2H), 4.01 (hept, *J* = 6.7 Hz, 1H), 3.64 (s, 3H), 3.55 (s, 3H), 1.32 (d, *J* = 6.7 Hz, 6H). ^{13}C NMR (126 MHz, $CDCl_3$) δ 190.6, 179.1, 169.9, 163.6 ($J_{C–F}$ = 252.8 Hz), 158.9, 132.7 ($J_{C–F}$ = 8.8 Hz), 132.2 ($J_{C–F}$ = 3.1 Hz), 119.6,

116.0 ($J_{C–F}$ = 21.9 Hz), 42.6, 33.2, 32.1, 21.8. HRMS (ESI-Orbitrap) *m/z* $[M + H]^+$ Calcd for $C_{16}H_{19}FN_3O_3S$ 352.1125, found 352.1121.

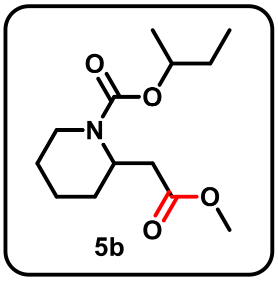

Yellowish oil (34 mg, 66%). Reaction condition: 0.2 mmol scale, LaI_3 5%, ***o*-Q** 10%, MeOH 0.4 ml, 12 h. ^1H NMR (300 MHz, $CDCl_3$) δ 4.71 (m, 2H), 4.02 (m, 1H), 3.66 (s, 3H), 2.78 (m, 1H), 2.51 (m, 2H), 1.67 (m, 8H), 1.21 (dd, *J* = 6.2, 2.2 Hz, 3H), 0.90 (t, *J* = 7.4 Hz, 3H). ^{13}C NMR (126 MHz, $CDCl_3$) δ 171.8, 155.4, 73.2, 51.7, 48.0, 39.4, 35.1, 29.1, 28.2, 25.3, 19.8, 18.9, 9.7. HRMS (ESI-Orbitrap) *m/z* $[M + Na]^+$ Calcd for $C_{13}H_{23}NO_4Na$ 280.1525, found 280.1517.

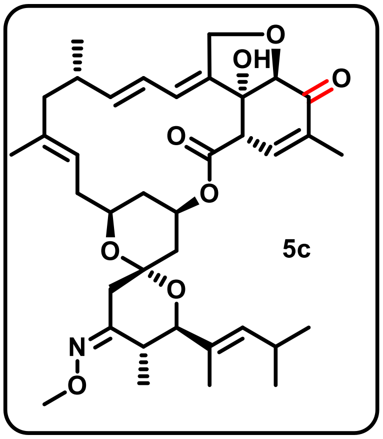

White solid (62 mg, 97%). Reaction condition: 0.1 mmol scale, LaI_3 5%, ***o*-Q** 10%, MeCN 0.4 ml, 4 h. ^1H NMR (500 MHz, $CDCl_3$) δ 6.59 (s, 1H), 5.86 (dd, *J* = 11.3, 3.1 Hz, 1H), 5.74 (dd, *J* = 14.7, 11.3 Hz, 1H), 5.43-5.34 (m, 2H), 5.19 (d, *J* = 9.0 Hz, 1H), 4.93 (m, 1H), 4.74 (qd, *J* = 14.3, 2.5 Hz, 2H), 3.83 (m, 5H), 3.63 (d, *J* = 10.3 Hz, 1H), 3.60 (m, 1H), 3.49 (m, 1H), 3.30 (d, *J* = 14.7 Hz, 1H), 2.65-2.55 (m, 1H), 2.44 (m, 1H), 2.31 (m, 1H), 2.27 -2.15 (m, 4H), 1.93-1.80 (m, 6H), 1.66 (s, 3H), 1.43 (m, 2H), 1.05-0.81 (m, 16H). ^{13}C NMR (126 MHz, $CDCl_3$) δ 192.2, 172.2, 155.9, 144.0, 138.4, 138.2, 137.7, 137.1, 136.8, 130.5, 123.4, 121.9, 121.0, 98.7, 82.0, 81.9, 81.0, 70.0, 69.1, 68.2, 61.4, 48.5, 46.7, 40.8, 37.5, 36.1, 35.9, 35.6, 34.4,

27.0, 22.9, 22.9, 22.3, 15.6, 15.6, 11.1, 11.0. HRMS (ESI-Orbitrap) *m*/*z* [M + H]$^+$ Calcd for $C_{37}H_{51}NO_8$ 638.3687, found 638.3679.

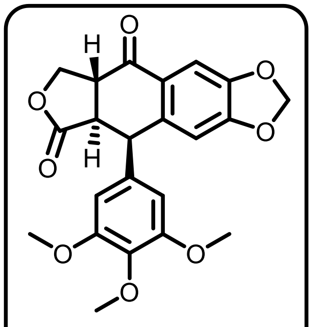

5d

Yellowish solid (80 mg, 97%). Reaction condition: 0.2 mmol scale, LaI_3 5%, ***o*-Q** 10%, MeCN 0.4 ml, 4 h. Characterization was in consistent with previous literature[73]. ^{1}H NMR (400 MHz, $CDCl_3$) δ 7.55 (s, 1H), 6.70 (s, 1H), 6.39 (s, 2H), 6.12 (m, 2H), 4.85 (d, *J* = 4.3 Hz, 1H), 4.56 (t, *J* = 9.2, 1H), 4.35 (t, *J* = 9.8 Hz, 1H), 3.82 (s, 3H), 3.75 (s, 6H), 3.52 (ddd, *J* = 15.7, 10.5, 7.6 Hz, 1H), 3.29 (dd, *J* = 15.5, 4.3 Hz, 1H). ^{13}C NMR (101 MHz, $CDCl_3$) δ 192.5, 173.2, 153.3, 153.2, 148.2, 141.6, 137.8, 132.2, 128.3, 109.8, 107.8, 106.2, 102.5, 67.1, 60.9, 56.4, 46.8, 44.8, 43.6. HRMS (ESI-Orbitrap) *m*/*z* [M + H]$^+$ Calcd for $C_{22}H_{21}O_8$ 413.1231, found 413.1227.

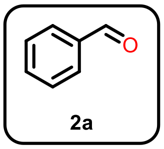

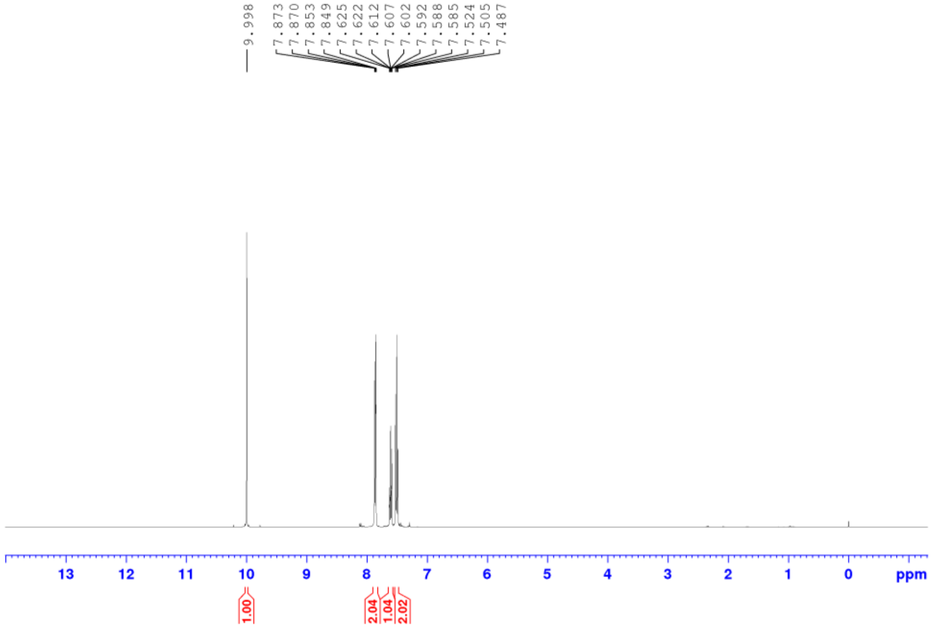

Supplementary Figure 17. ^{1}H NMR spectra of compound **2a**.

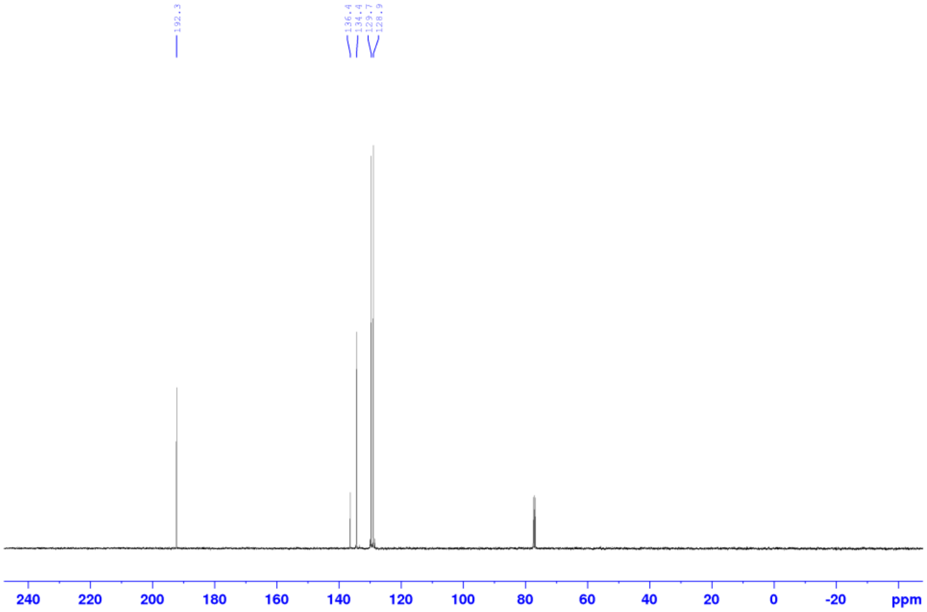

Supplementary Figure 18. ^{13}C NMR spectra of compound **2a**.

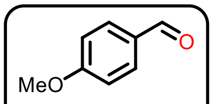

2b

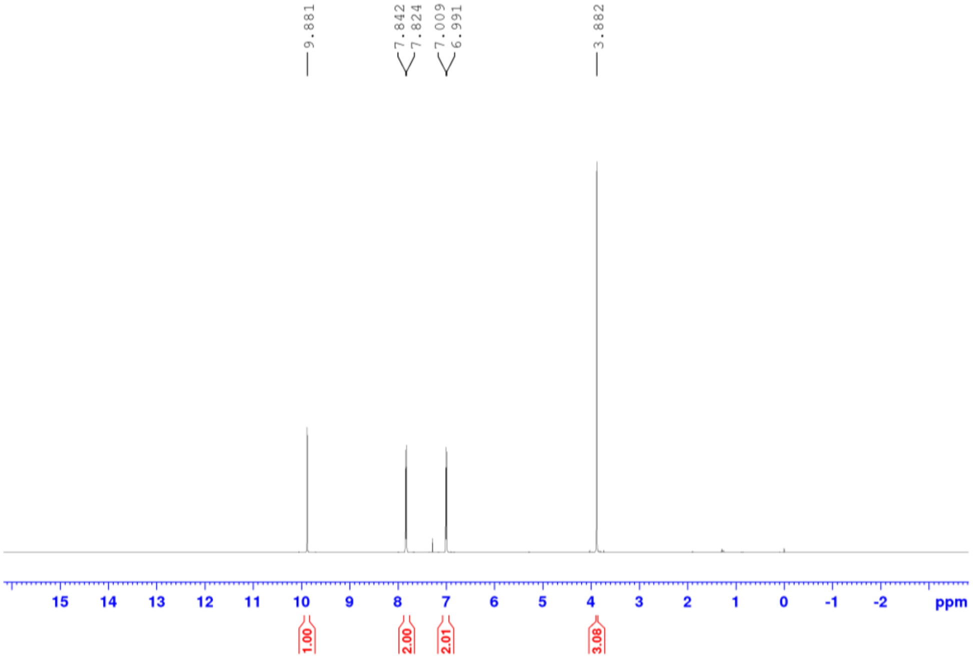

Supplementary Figure 19. ^{1}H NMR spectra of compound **2b**.

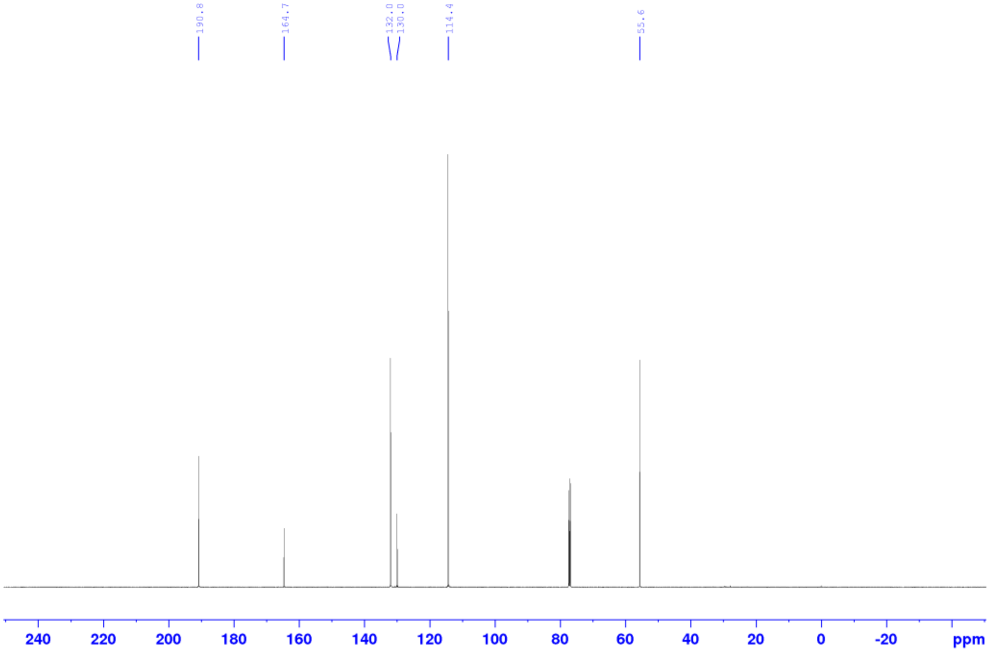

Supplementary Figure 20. ^{13}C NMR spectra of compound **2b**.

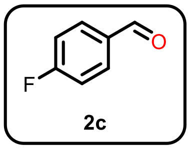

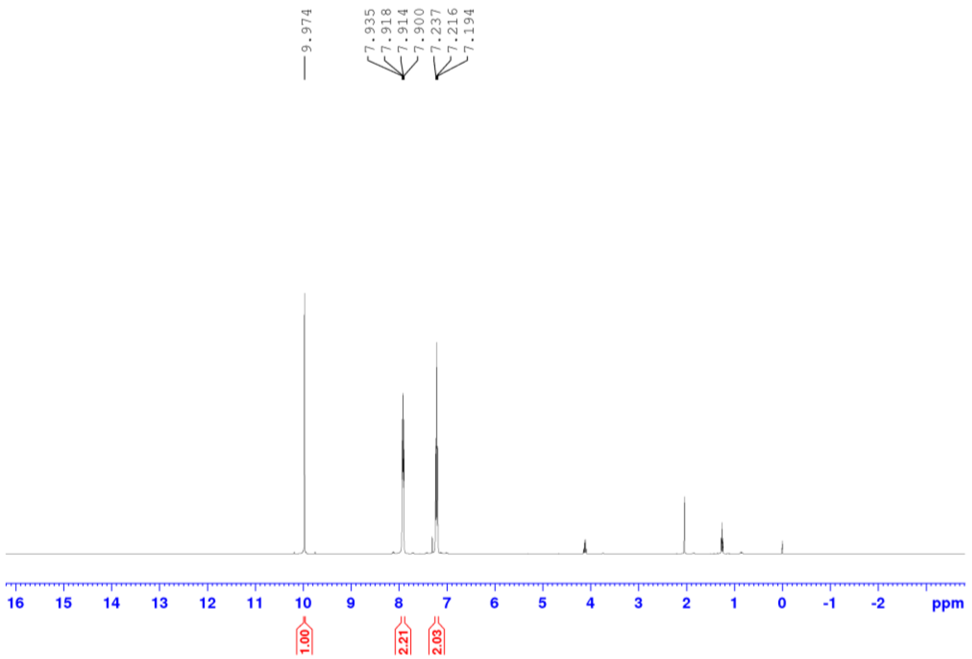

Supplementary Figure 21. ^{1}H NMR spectra of compound **2c**.

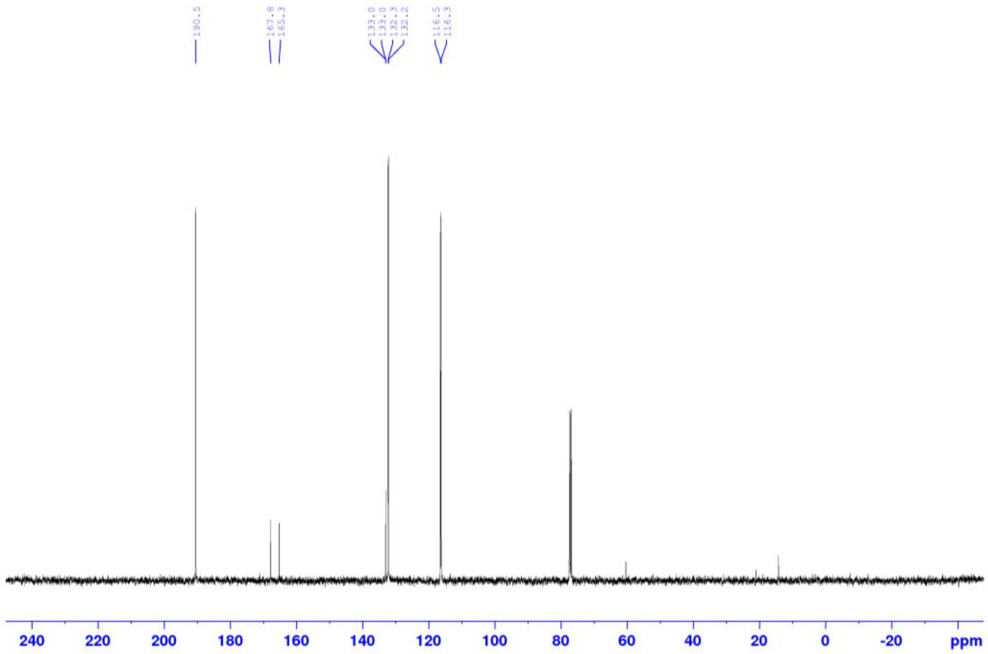

Supplementary Figure 22. ^{13}C NMR spectra of compound **2c**.

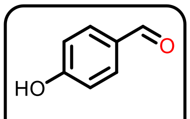

2d

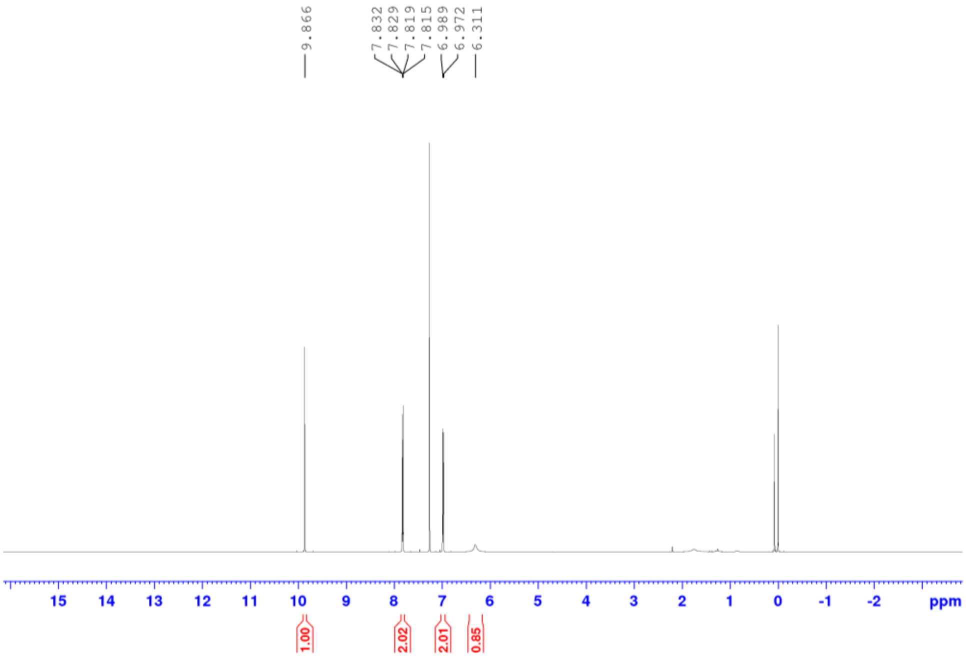

Supplementary Figure 23. ^{1}H NMR spectra of compound **2d**.

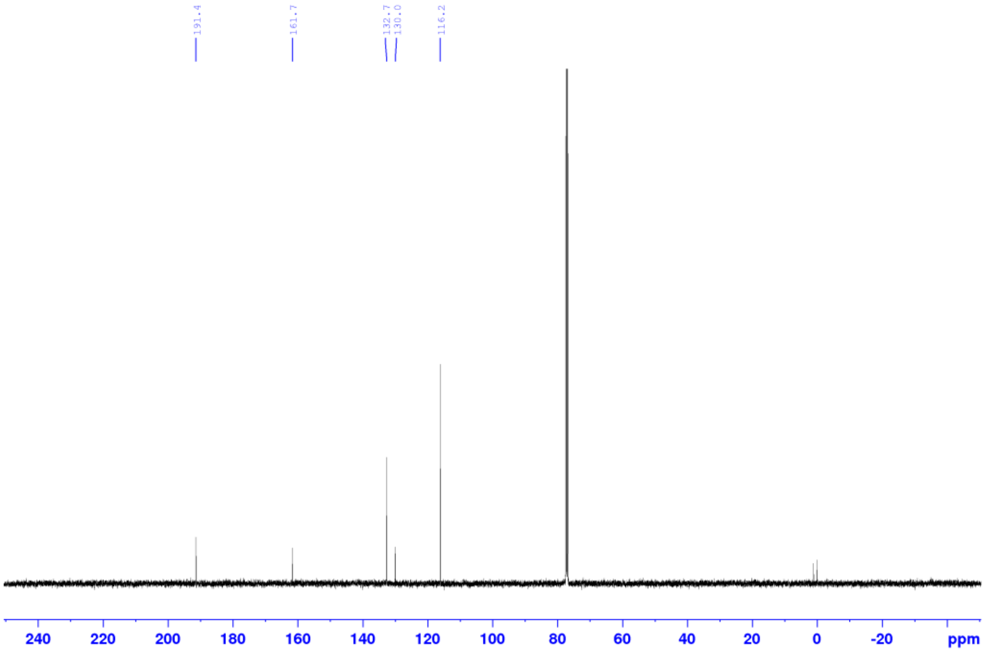

Supplementary Figure 24. ^{13}C NMR spectra of compound **2d**.

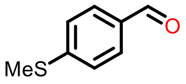

2e

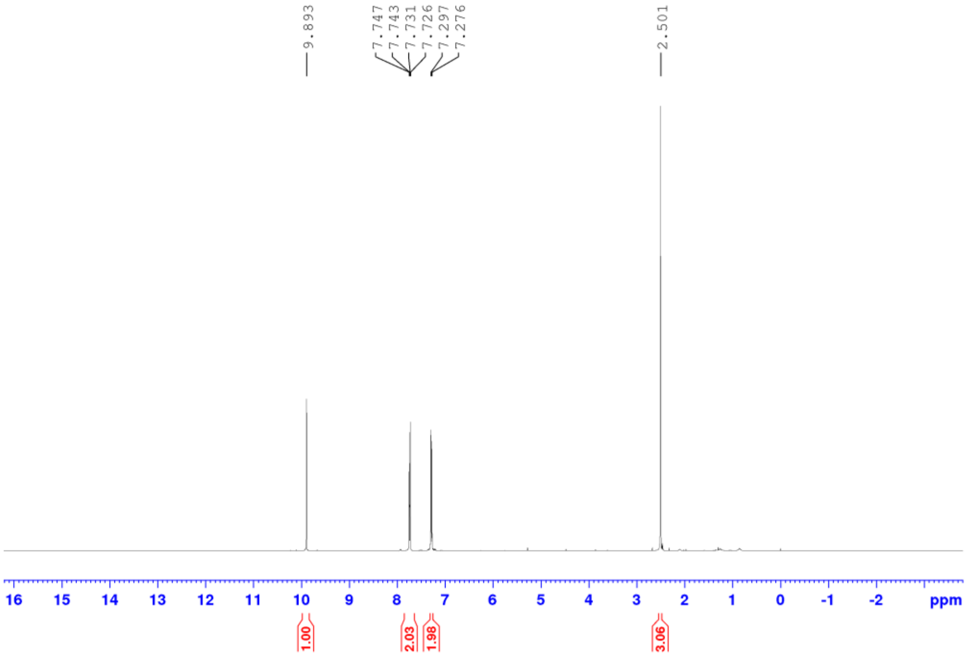

Supplementary Figure 25. ^{1}H NMR spectra of compound **2e**.

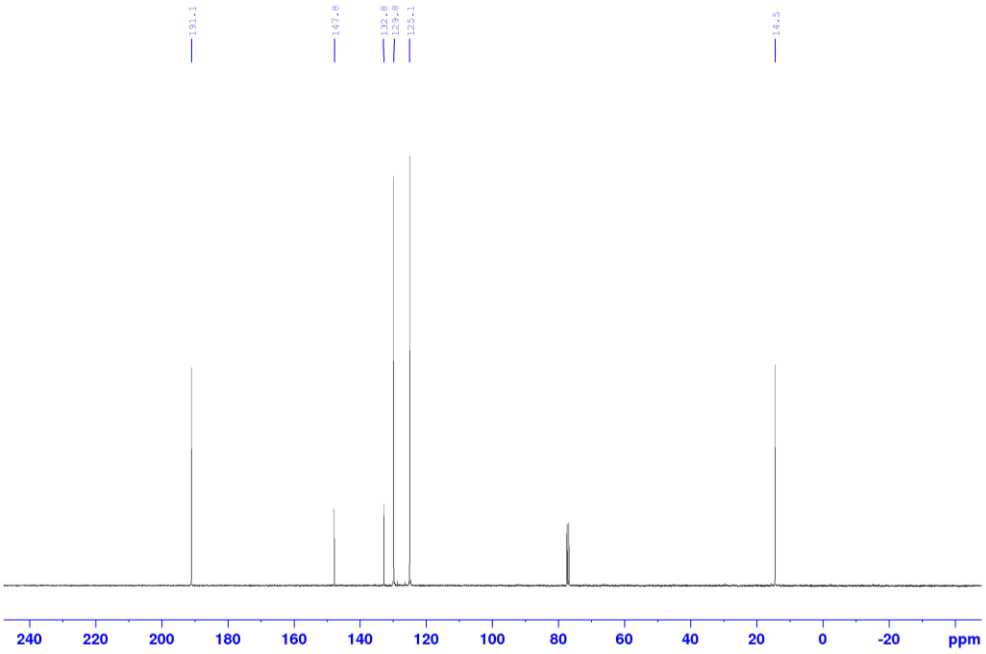

Supplementary Figure 26. ^{13}C NMR spectra of compound **2e**.

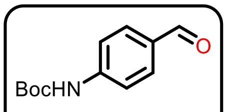

2f

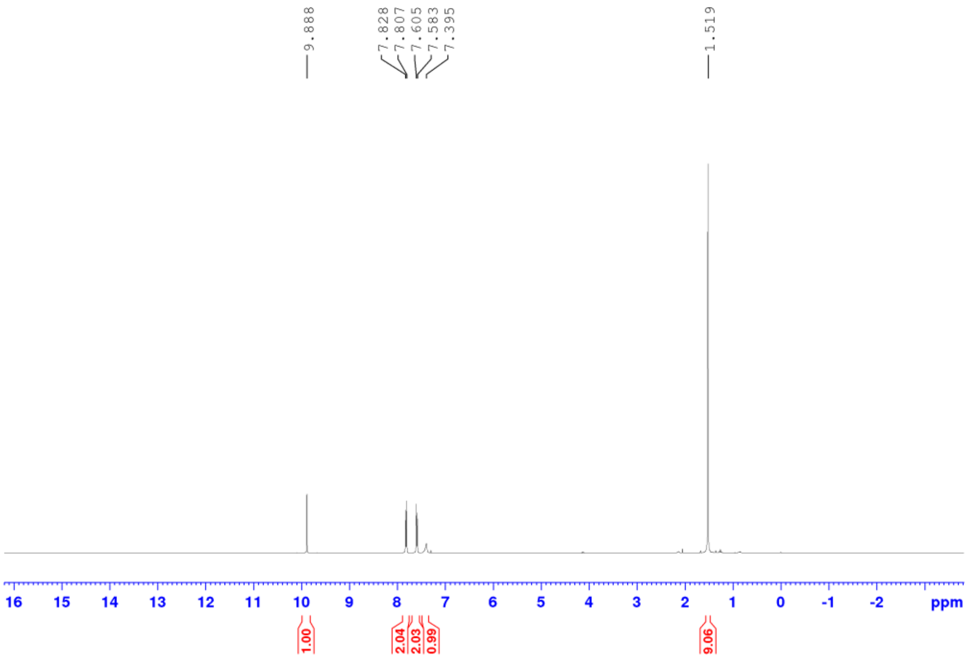

Supplementary Figure 27. ^{1}H NMR spectra of compound **2f**.

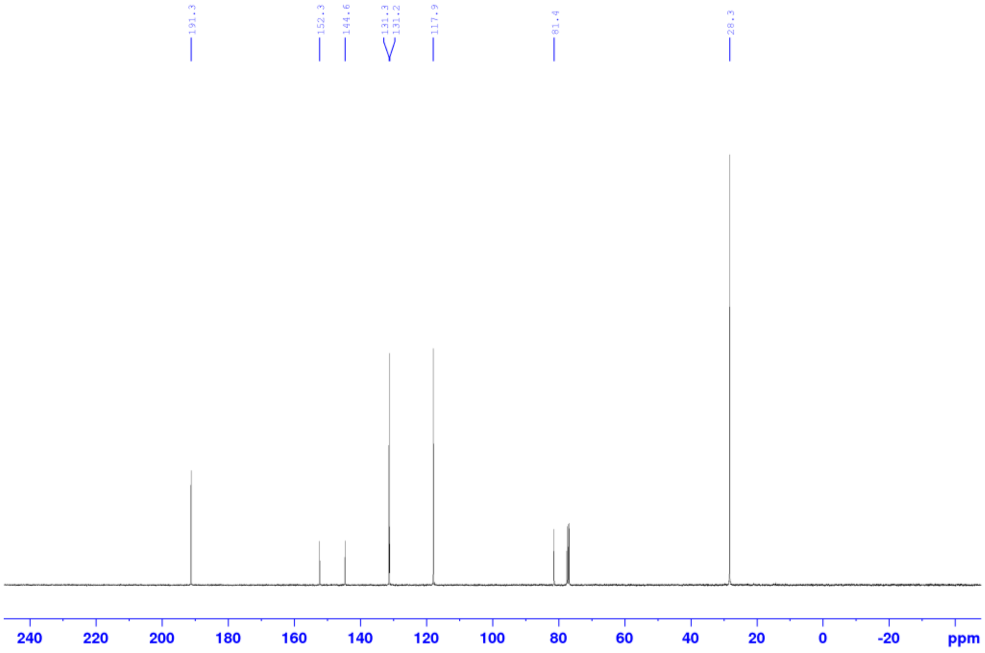

Supplementary Figure 28. ^{13}C NMR spectra of compound **2f**.

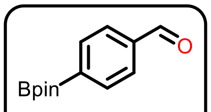

2g

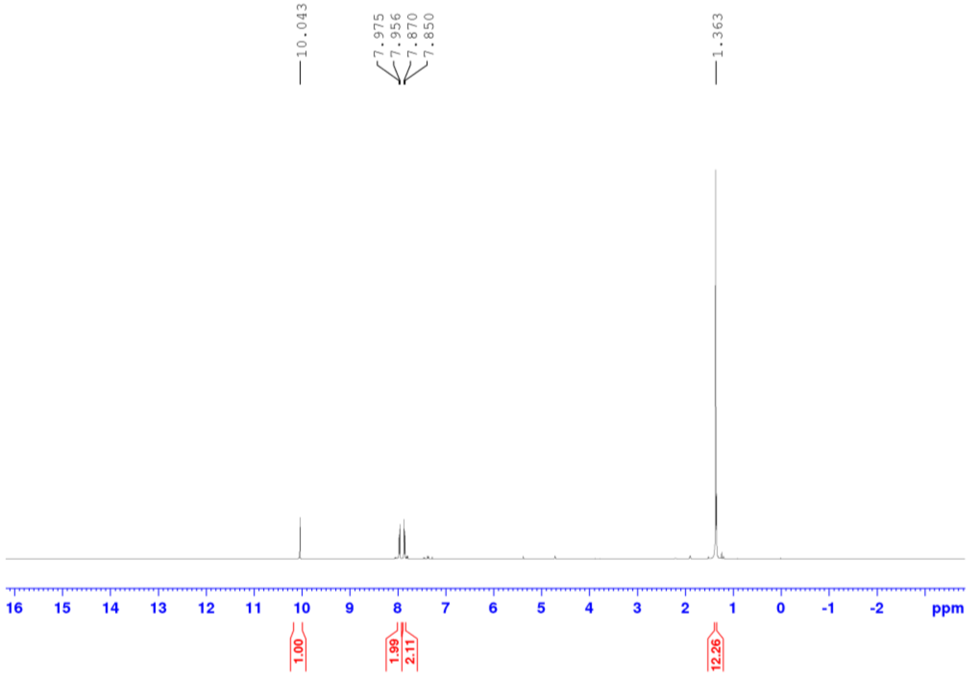

Supplementary Figure 29. ^{1}H NMR spectra of compound **2g**.

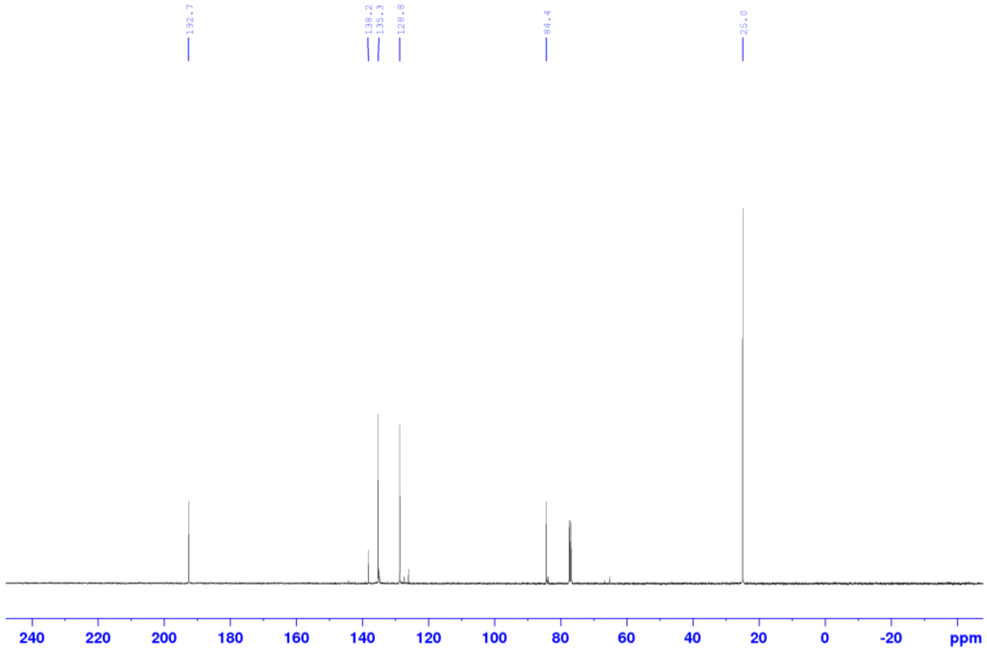

Supplementary Figure 30. ^{13}C NMR spectra of compound **2g**.

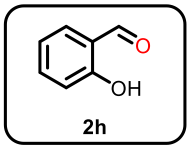

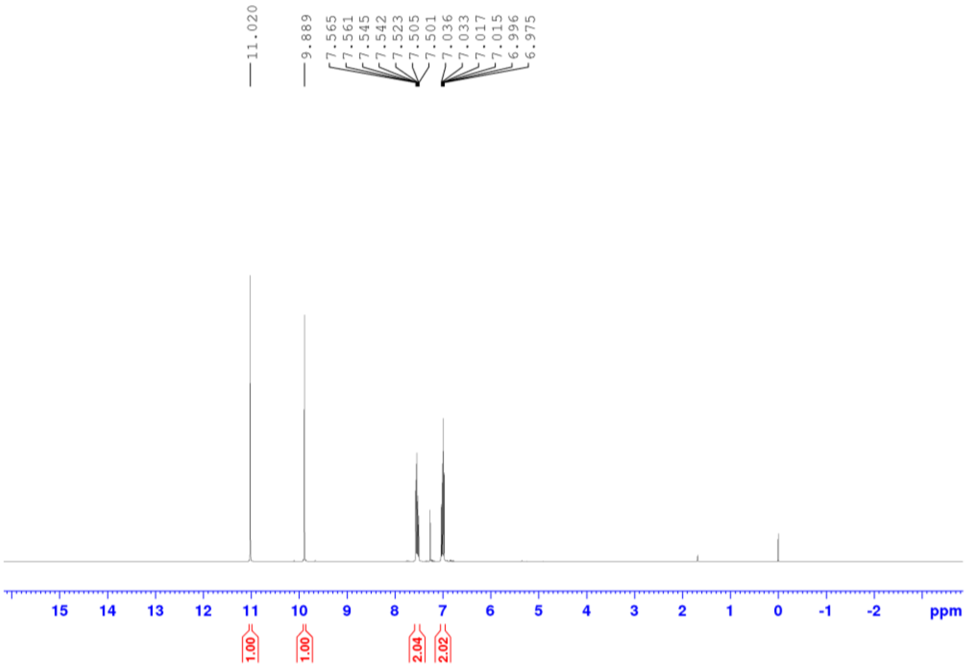

Supplementary Figure 31. ^{1}H NMR spectra of compound **2h**.

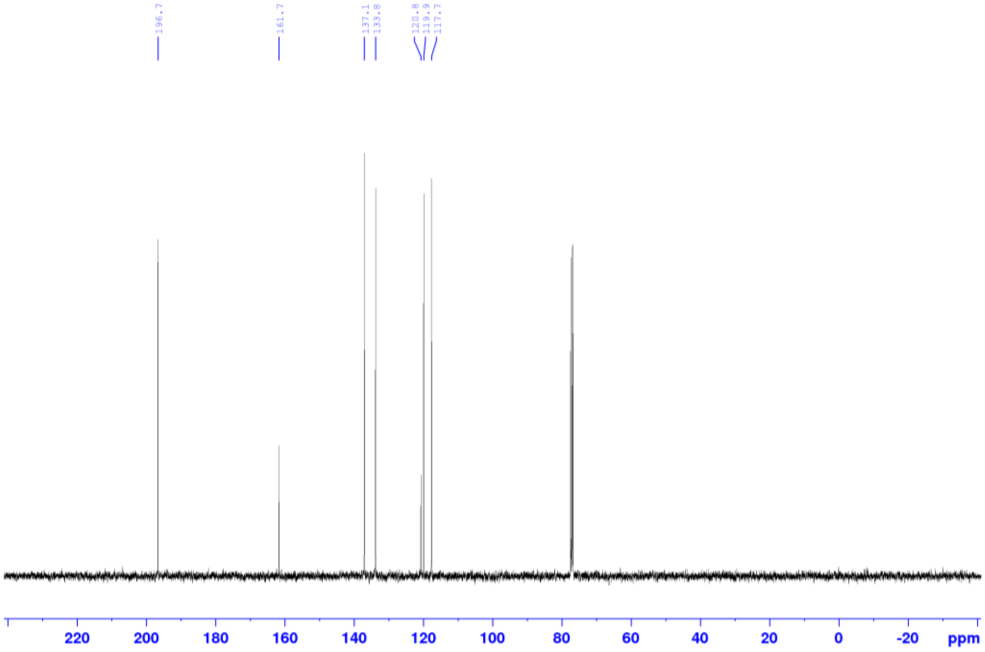

Supplementary Figure 32. ^{13}C NMR spectra of compound **2h**.

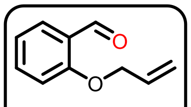

2i

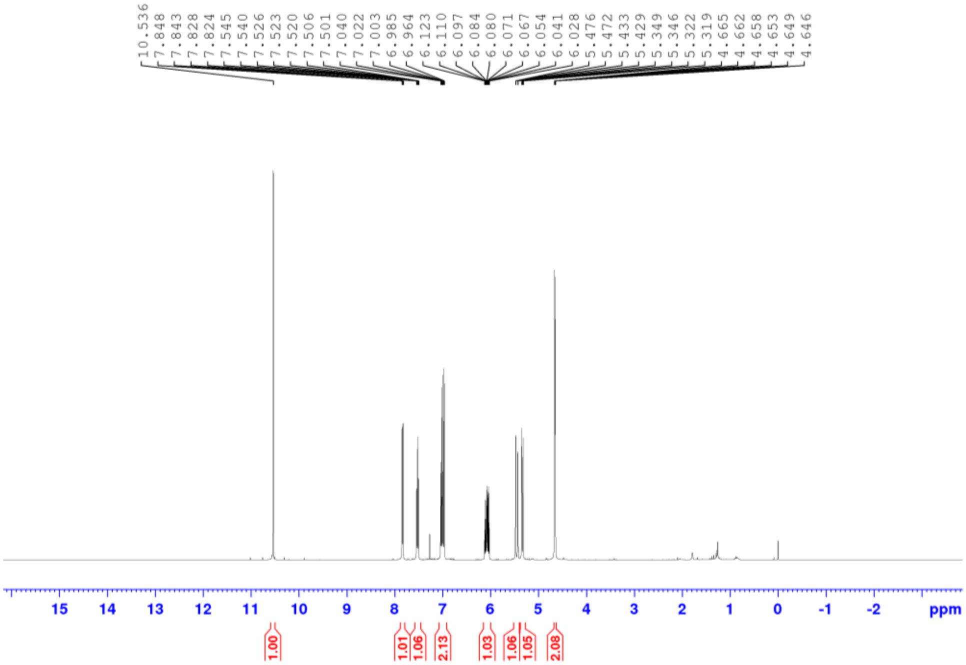

Supplementary Figure 33. ^{1}H NMR spectra of compound **2i**.

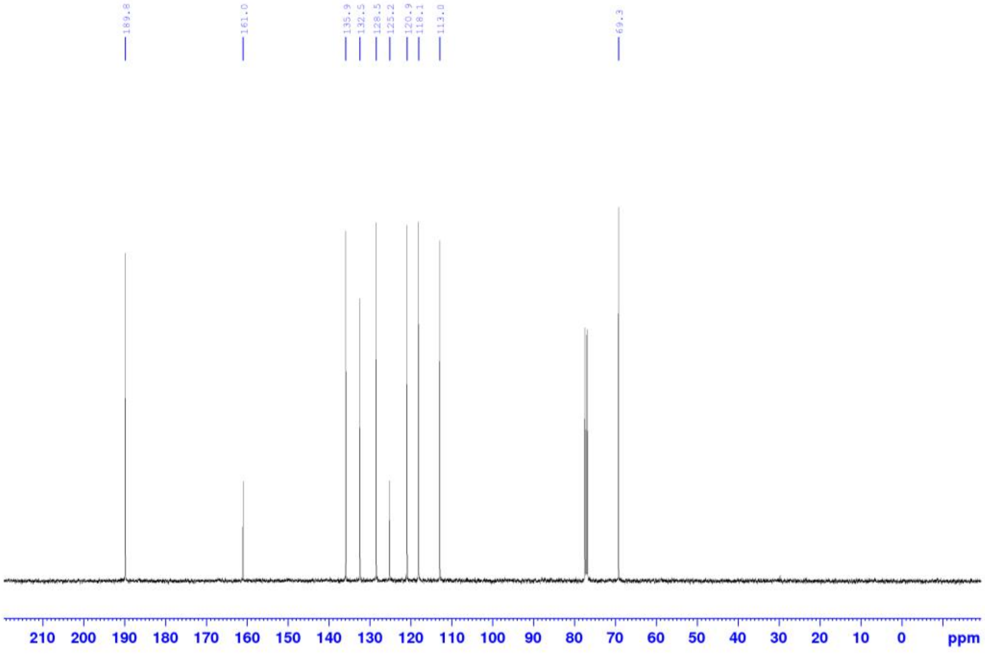

Supplementary Figure 34. ^{13}C NMR spectra of compound **2i**.

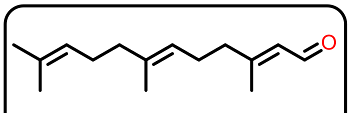
2j

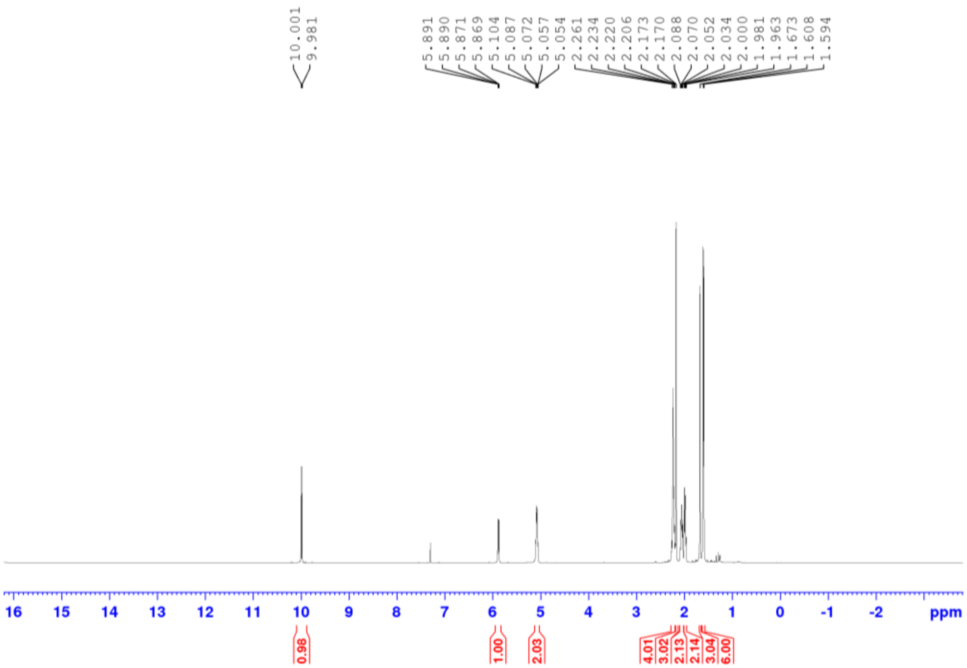

Supplementary Figure 35. ^{1}H NMR spectra of compound **2j**.

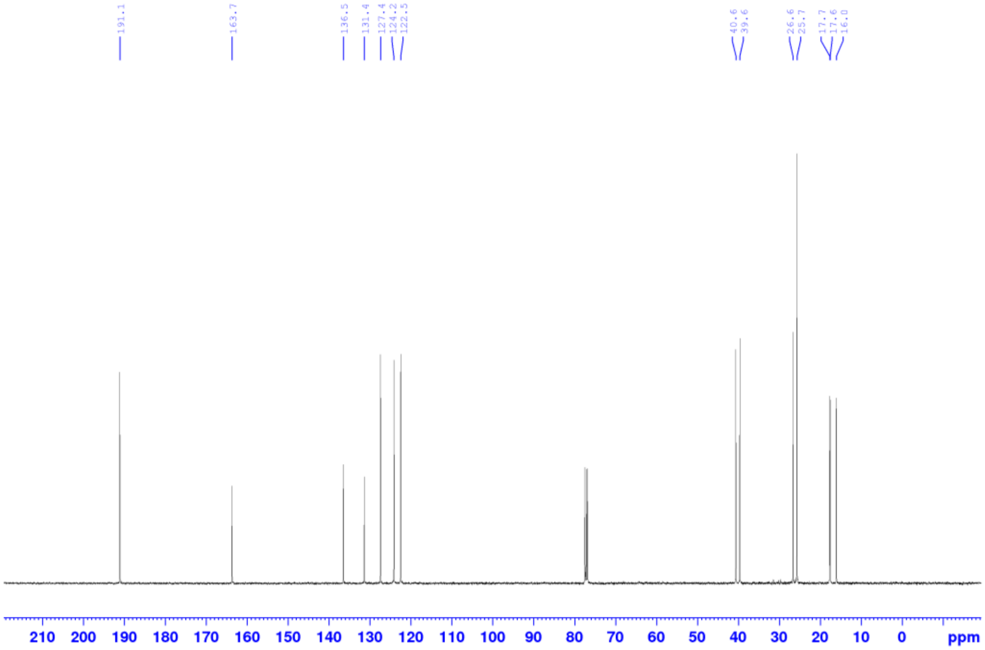

Supplementary Figure 36. ^{13}C NMR spectra of compound **2j**.

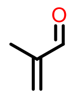

2k

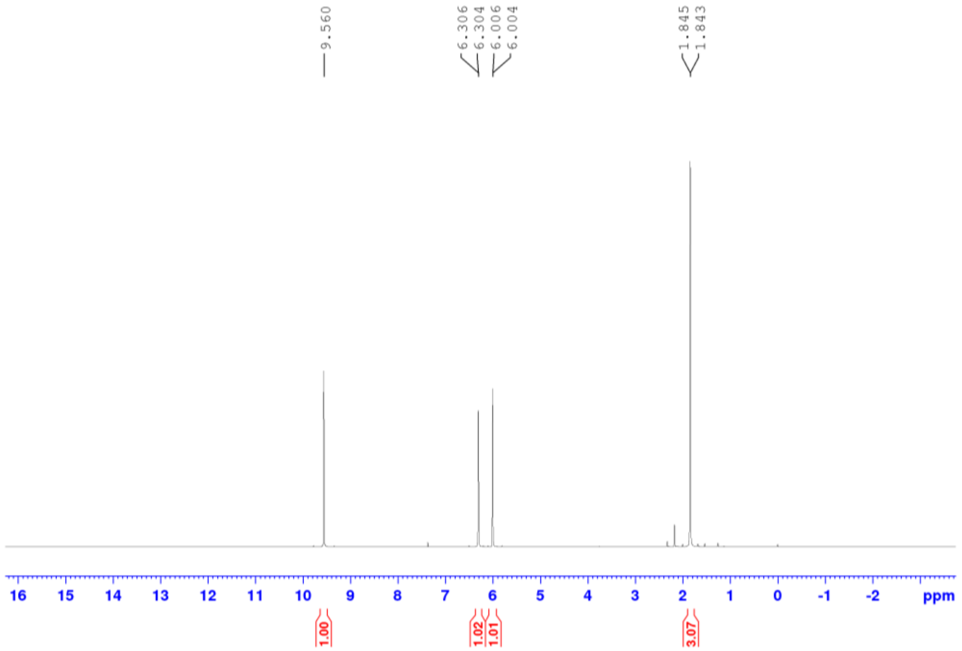

Supplementary Figure 37. ^{1}H NMR spectra of compound **2k**.

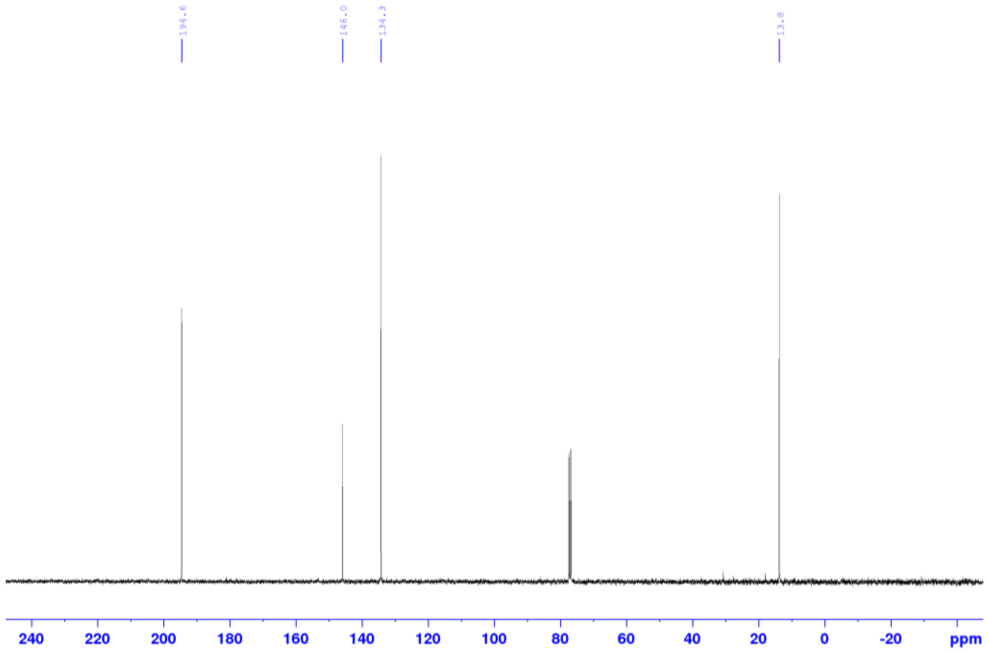

Supplementary Figure 38. ^{13}C NMR spectra of compound **2k**.

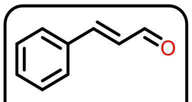

2l

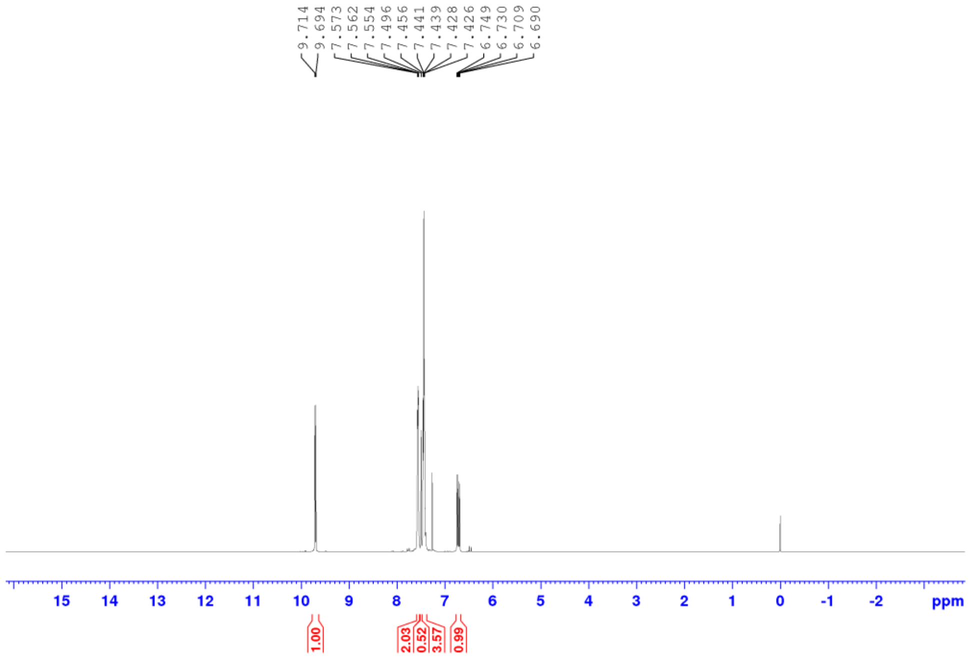

Supplementary Figure 39. ^{1}H NMR spectra of compound **2l**.

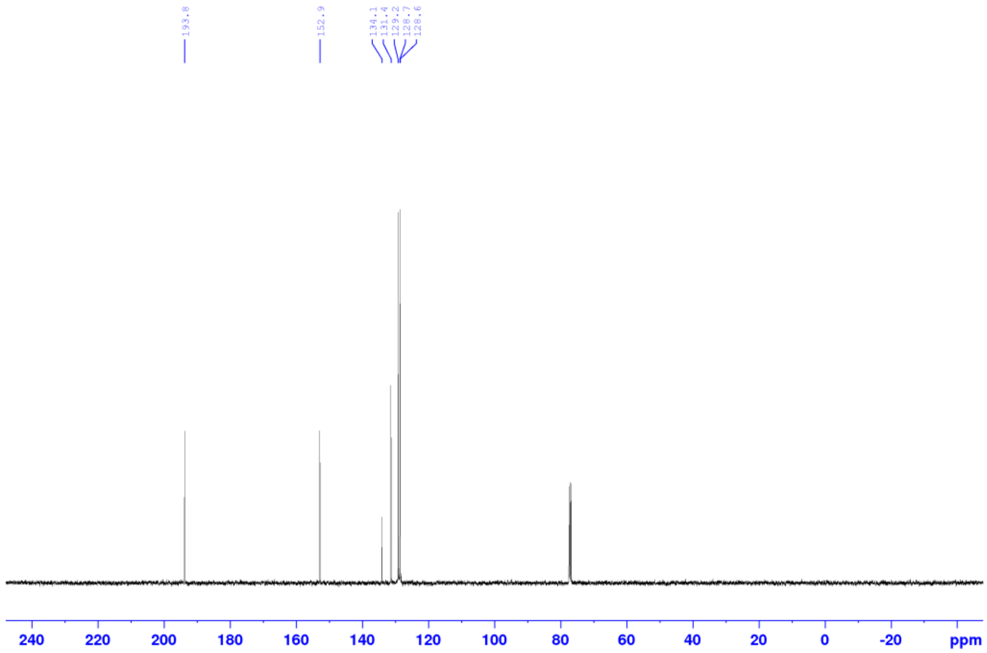

Supplementary Figure 40. ^{13}C NMR spectra of compound **2l**.

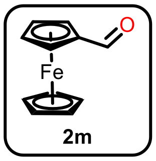

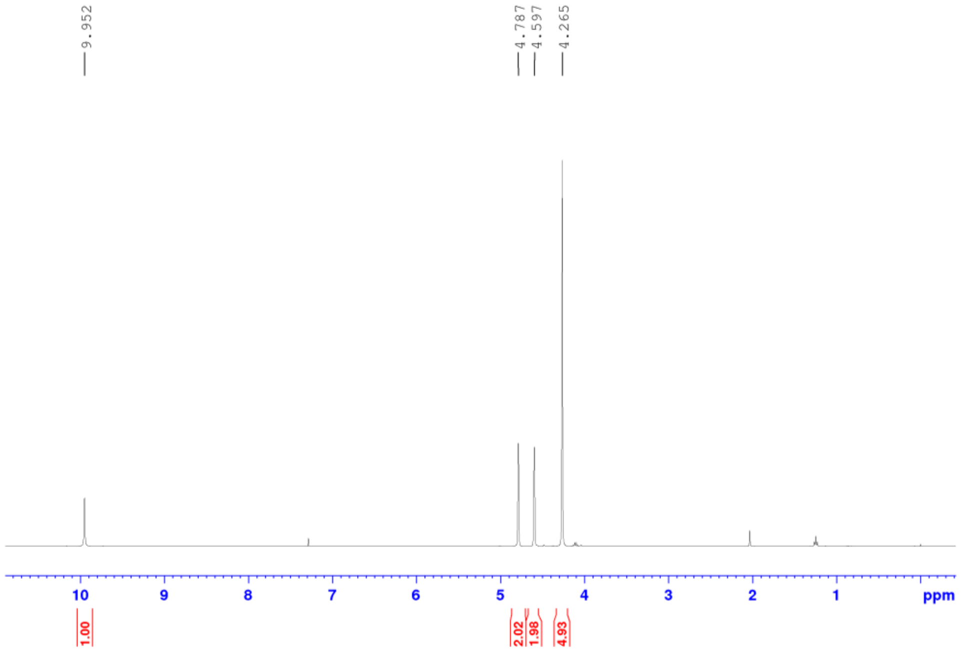

Supplementary Figure 41. ^{1}H NMR spectra of compound **2m**.

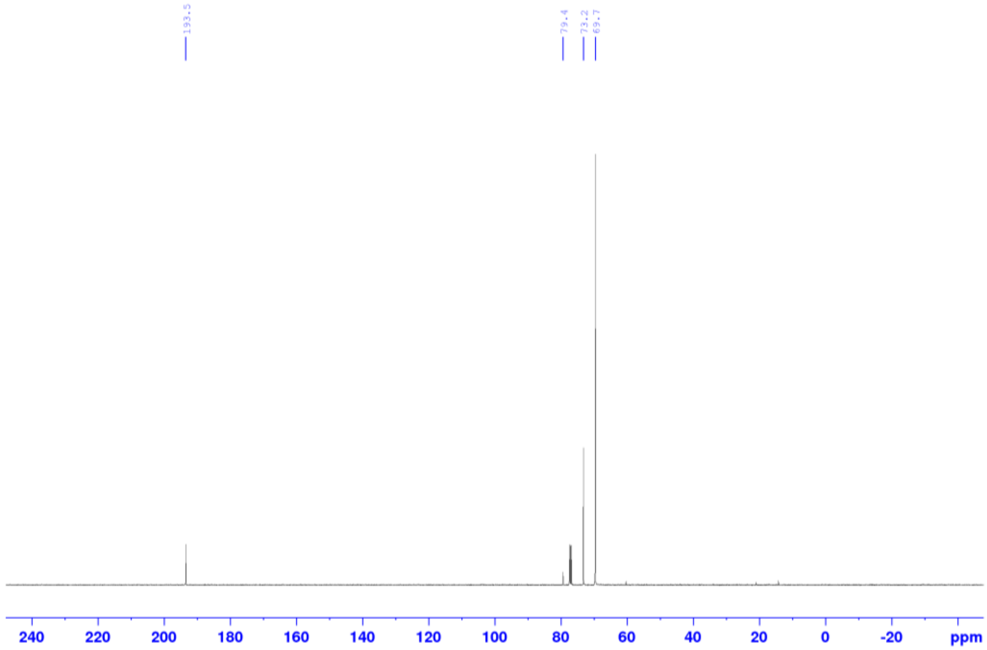

Supplementary Figure 42. ^{13}C NMR spectra of compound **2m**.

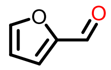

2n

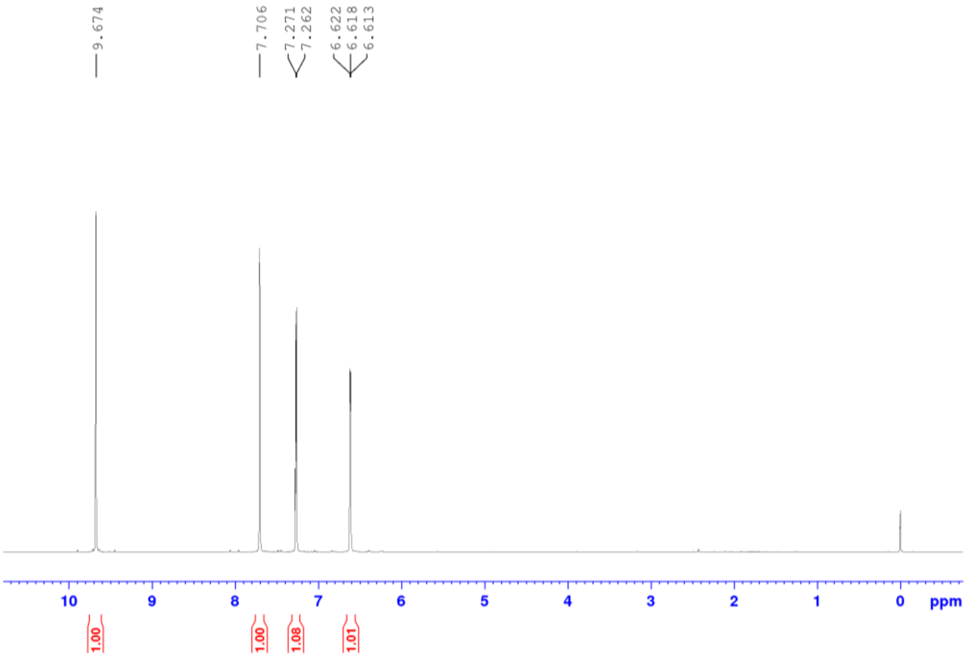

Supplementary Figure 43. ^{1}H NMR spectra of compound **2n**.

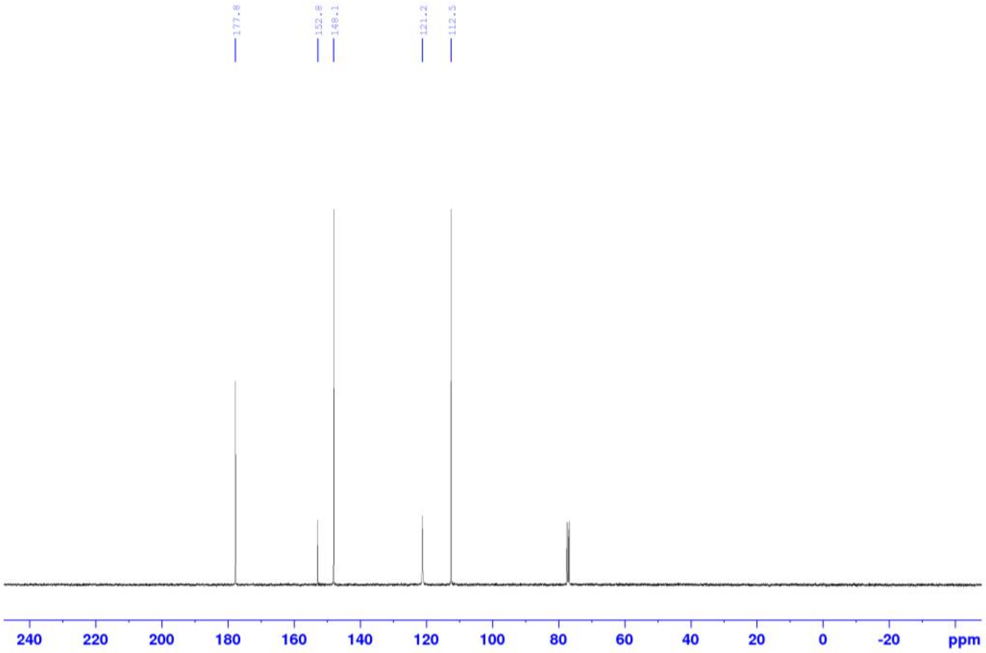

Supplementary Figure 44. ^{13}C NMR spectra of compound **2n**.

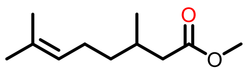

3a

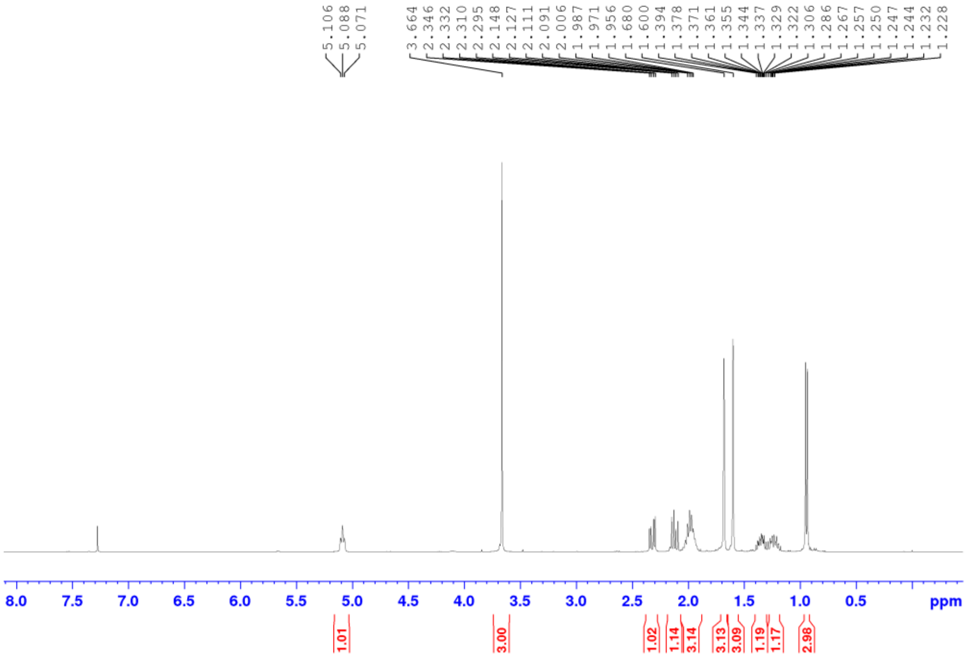

Supplementary Figure 45. ^{1}H NMR spectra of compound **3a**.

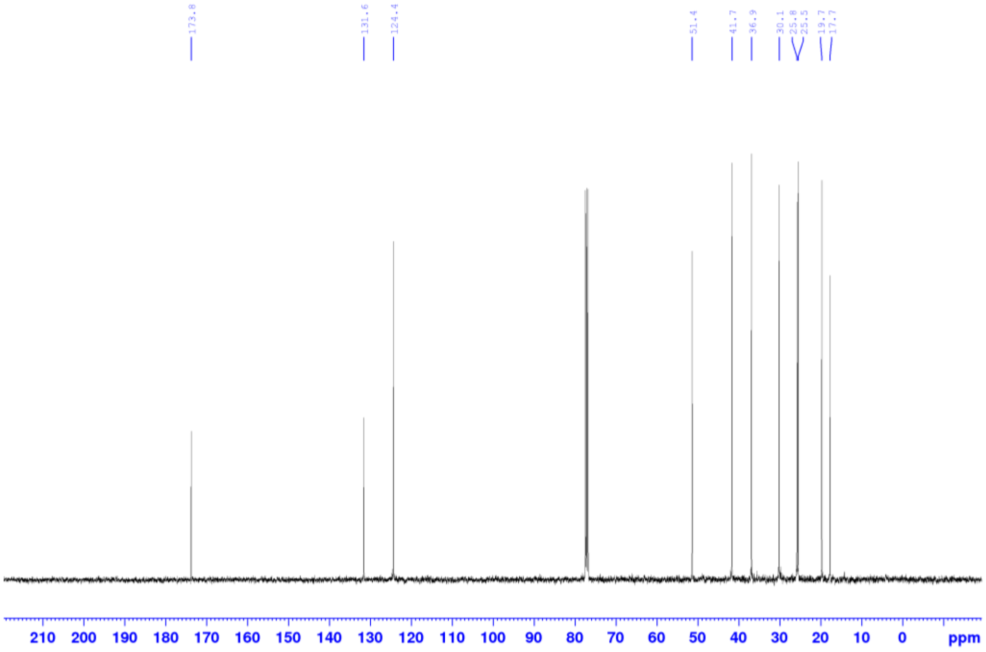

Supplementary Figure 46. ^{13}C NMR spectra of compound **3a**.

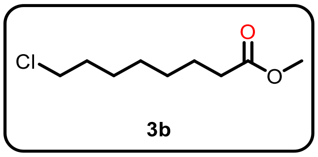

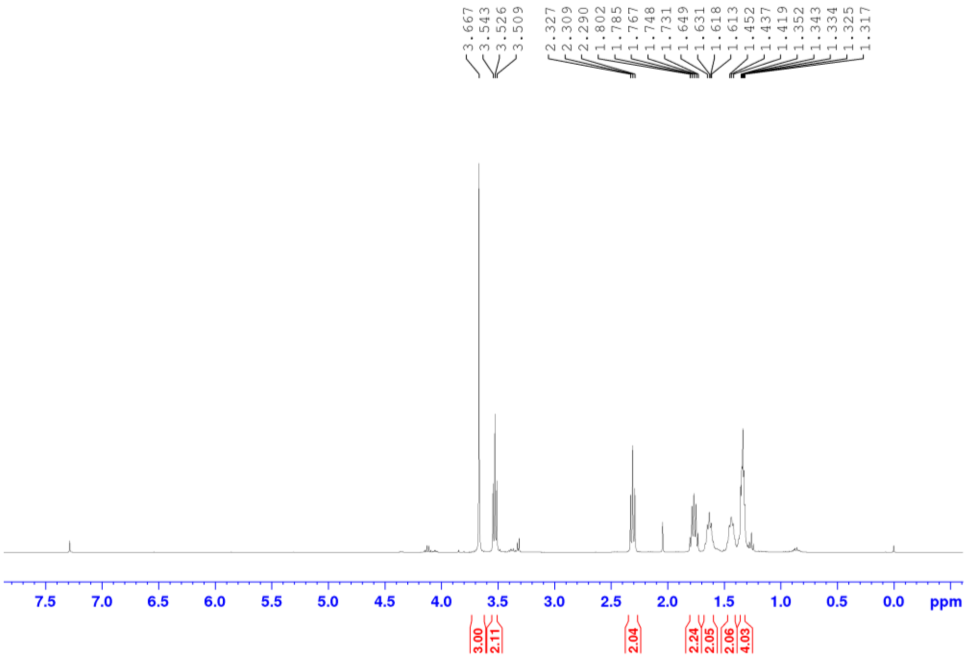

Supplementary Figure 47. ^{1}H NMR spectra of compound **3b**.

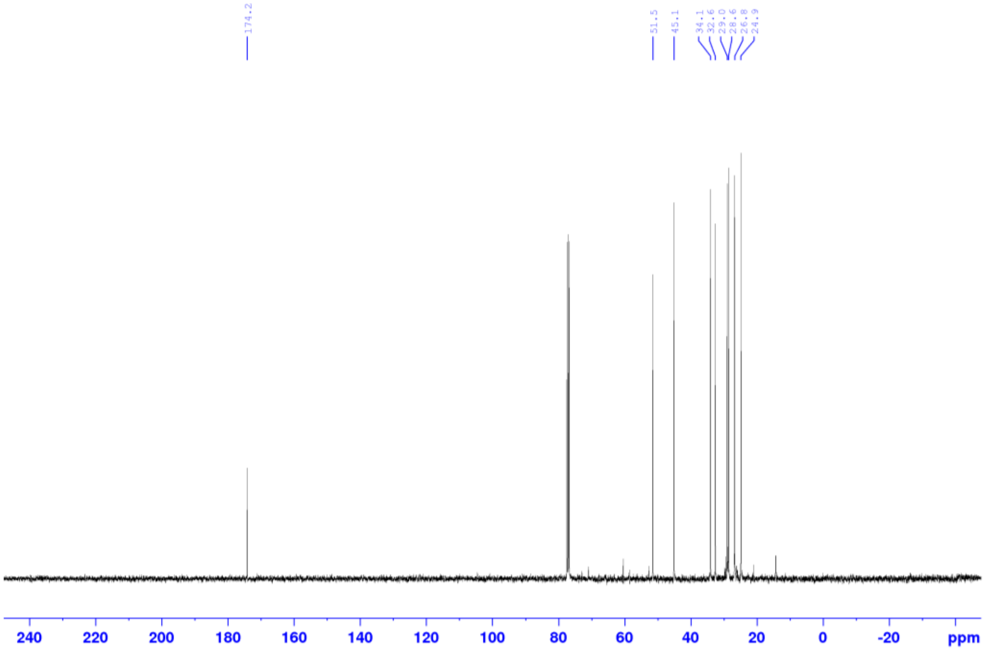

Supplementary Figure 48. ^{13}C NMR spectra of compound **3b**.

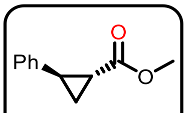

3c

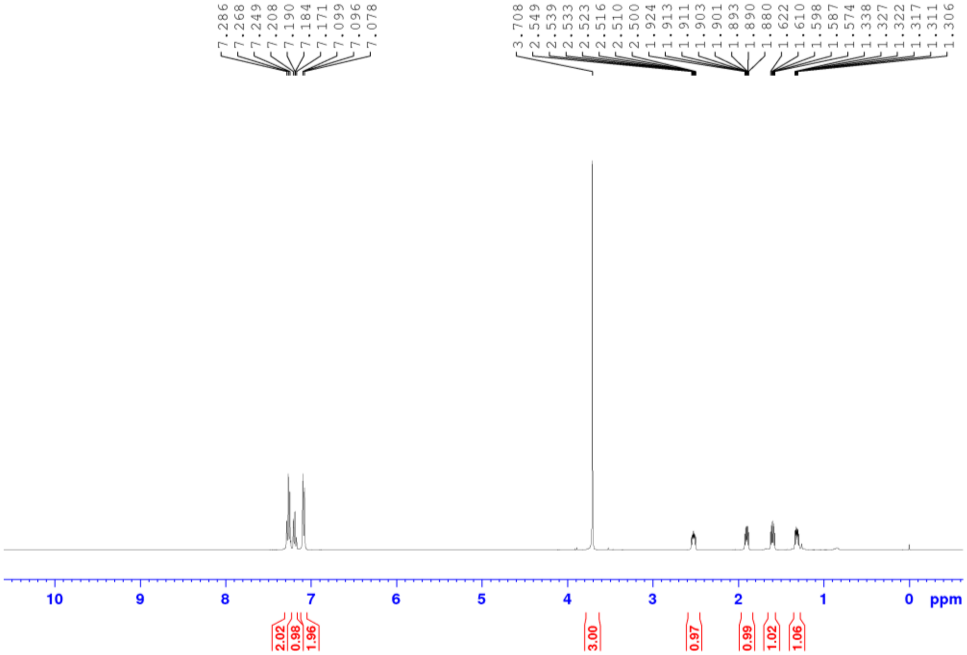

Supplementary Figure 49. ^{1}H NMR spectra of compound **3c**.

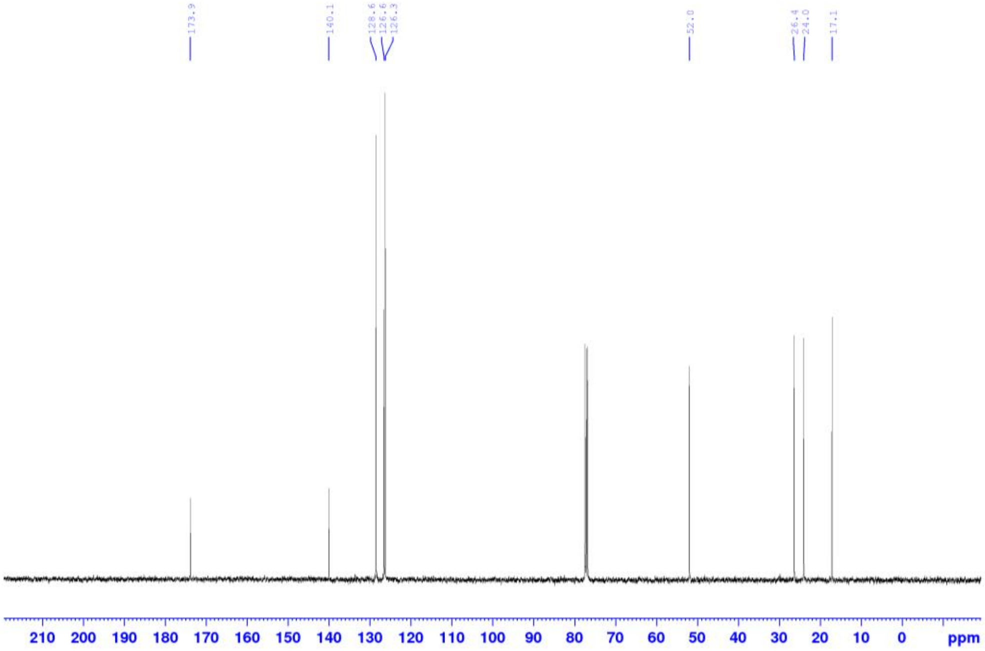

Supplementary Figure 50. ^{13}C NMR spectra of compound **3c**.

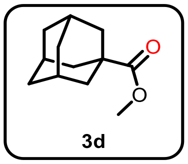

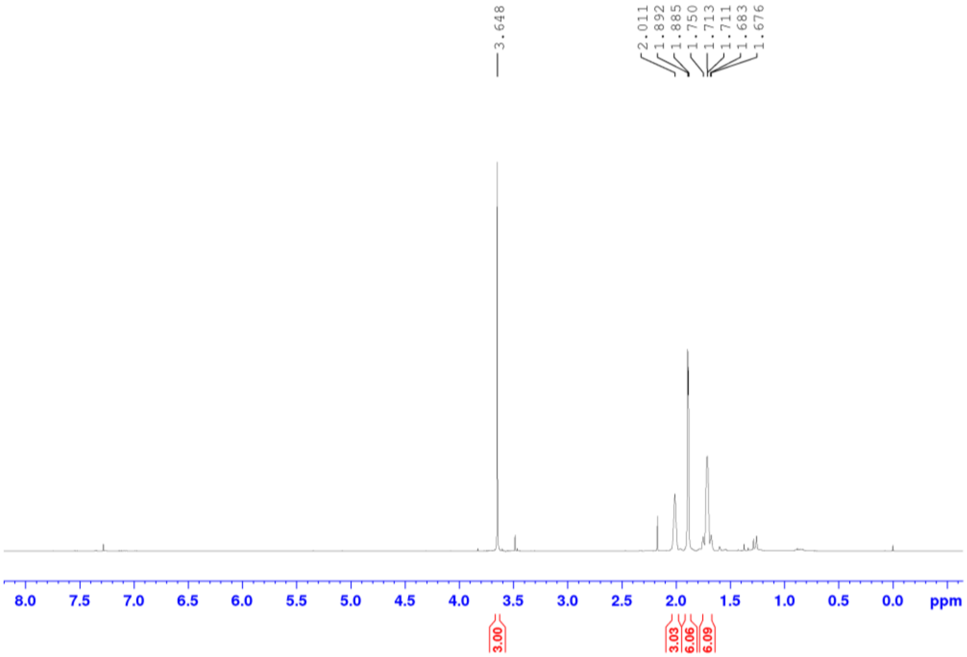

Supplementary Figure 51. ^{1}H NMR spectra of compound **3d**.

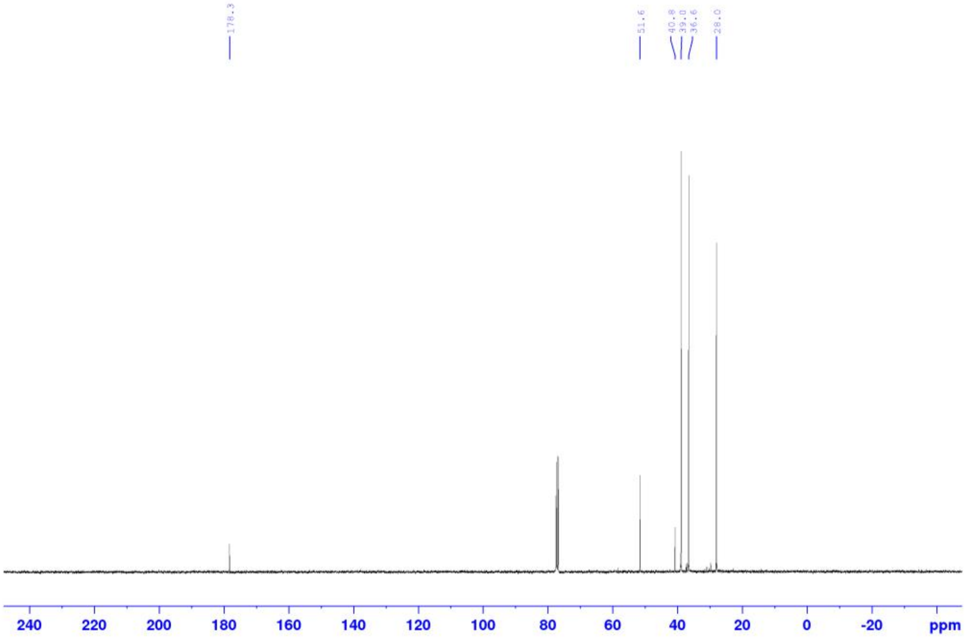

Supplementary Figure 52. ^{13}C NMR spectra of compound **3d**.

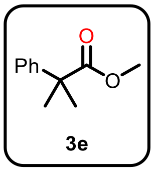

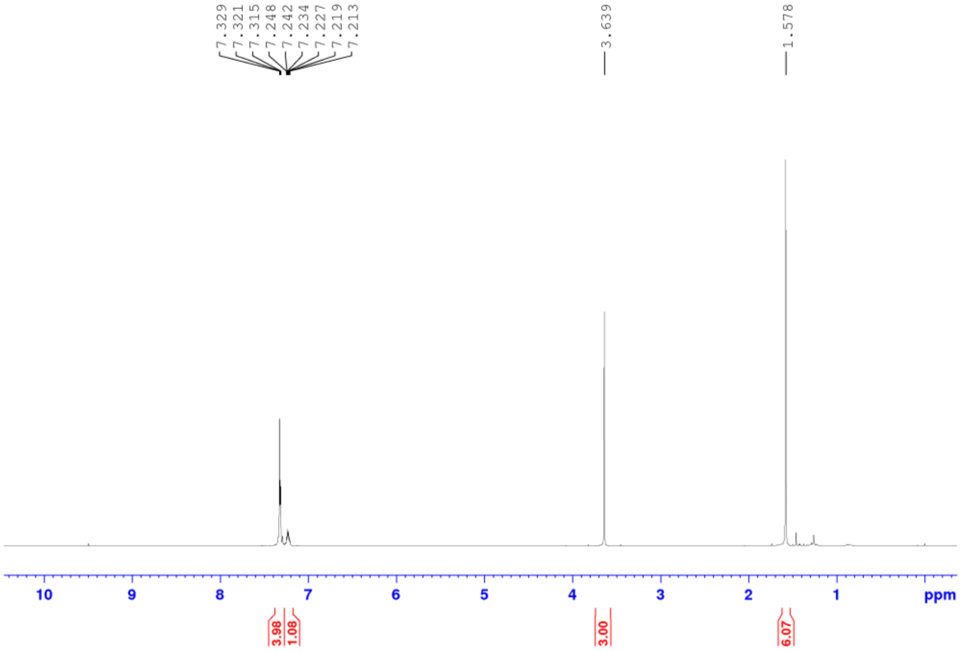

Supplementary Figure 53. ^{1}H NMR spectra of compound **3e**.

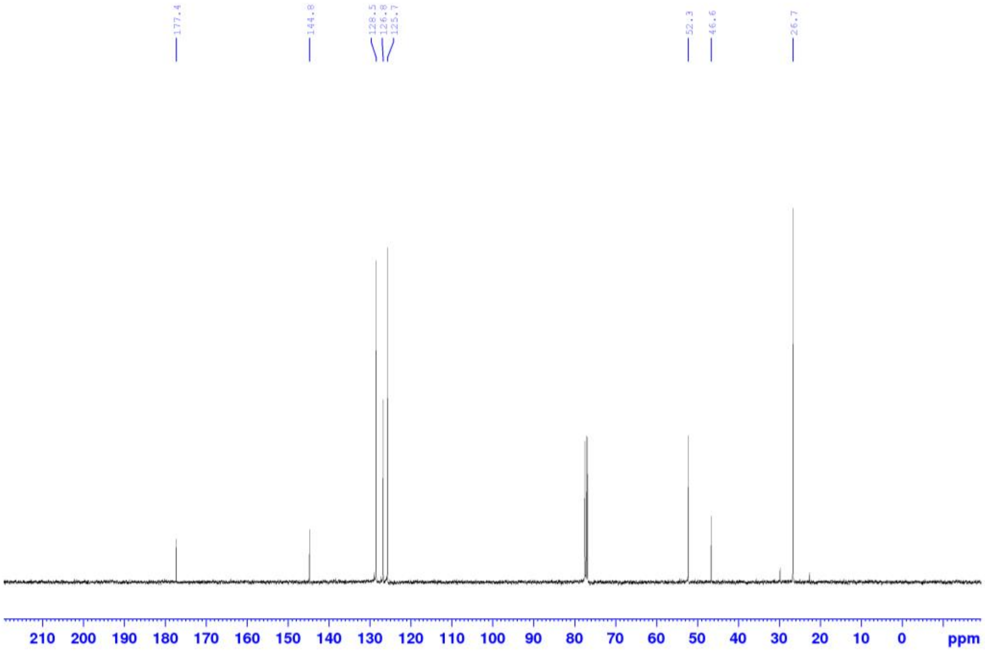

Supplementary Figure 54. ^{13}C NMR spectra of compound **3e**.

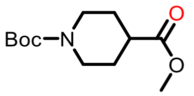

3f

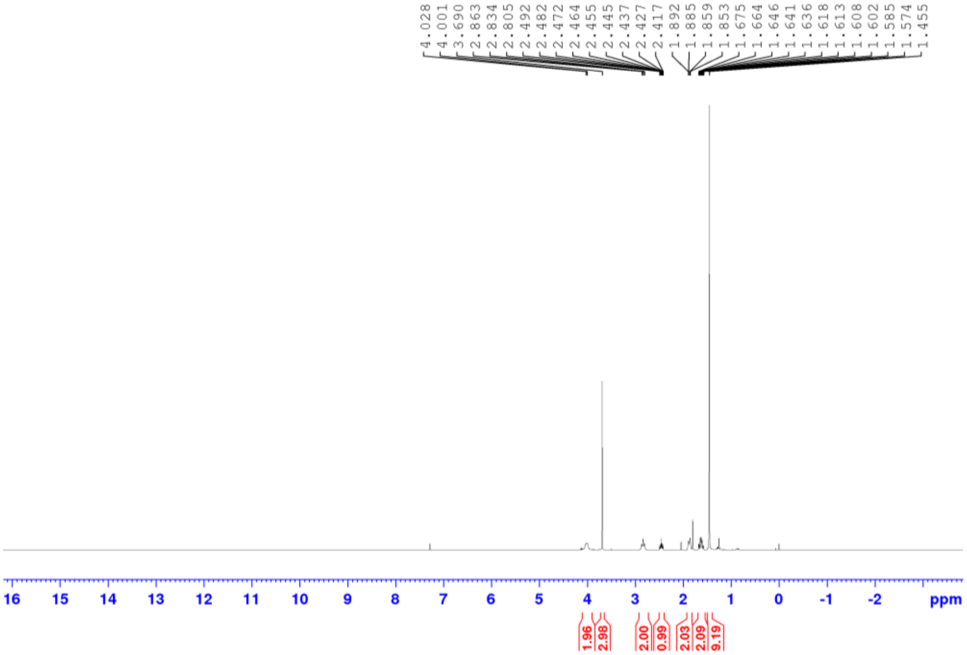

Supplementary Figure 55. ^{1}H NMR spectra of compound **3f**.

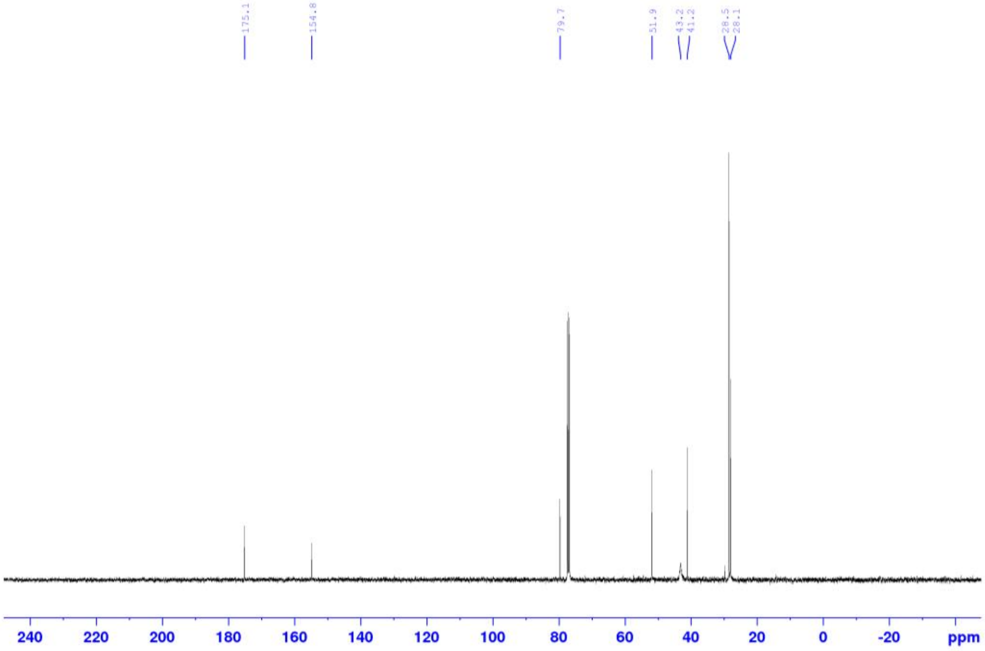

Supplementary Figure 56. ^{13}C NMR spectra of compound **3f**.

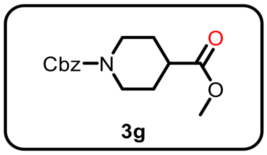

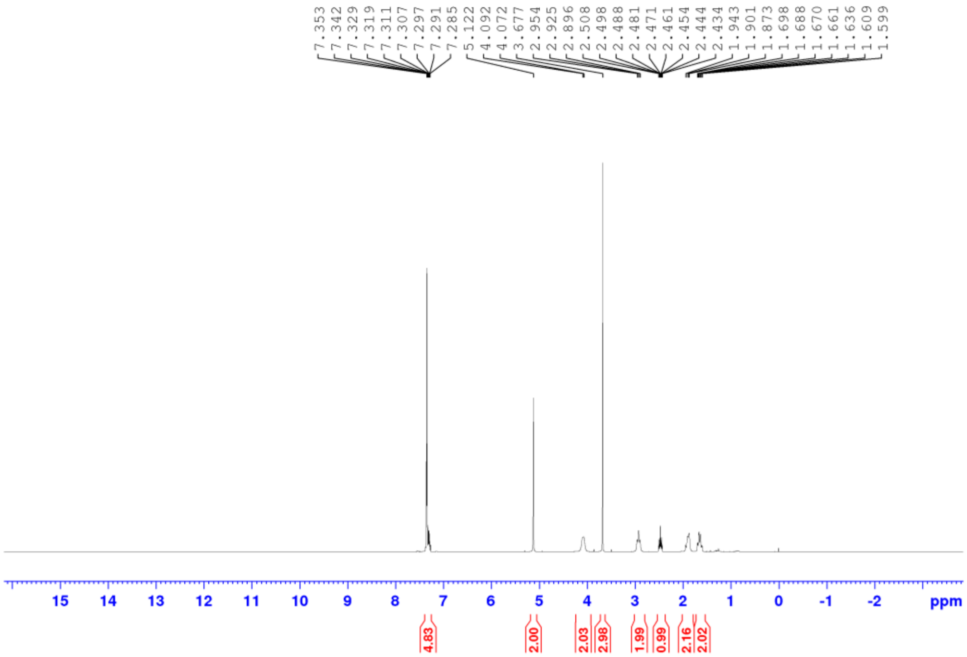

Supplementary Figure 57. ^{1}H NMR spectra of compound **3g**.

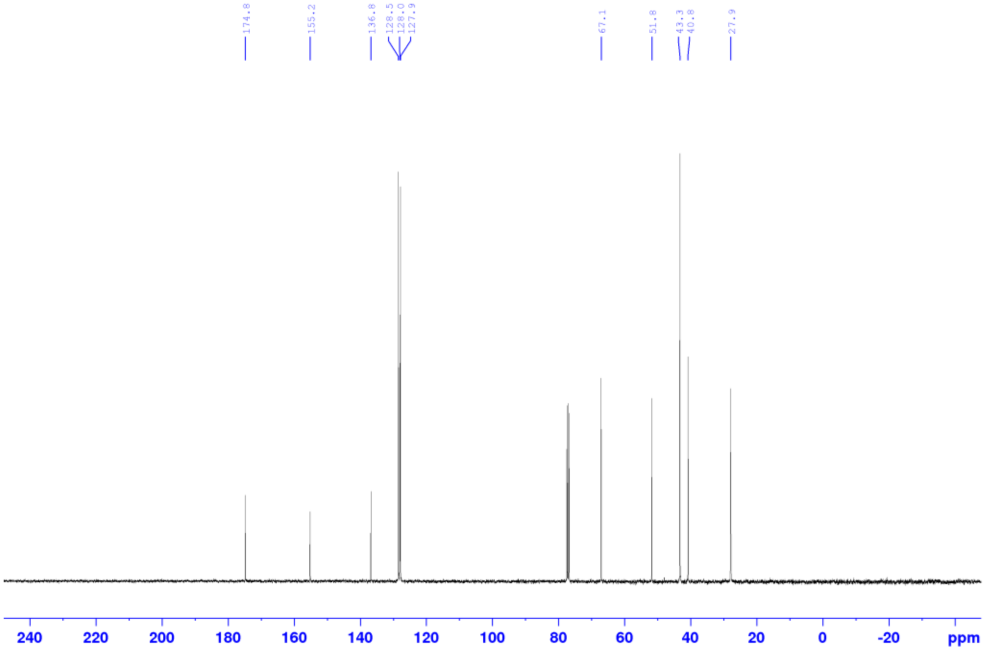

Supplementary Figure 58. ^{13}C NMR spectra of compound **3g**.

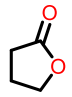

3h

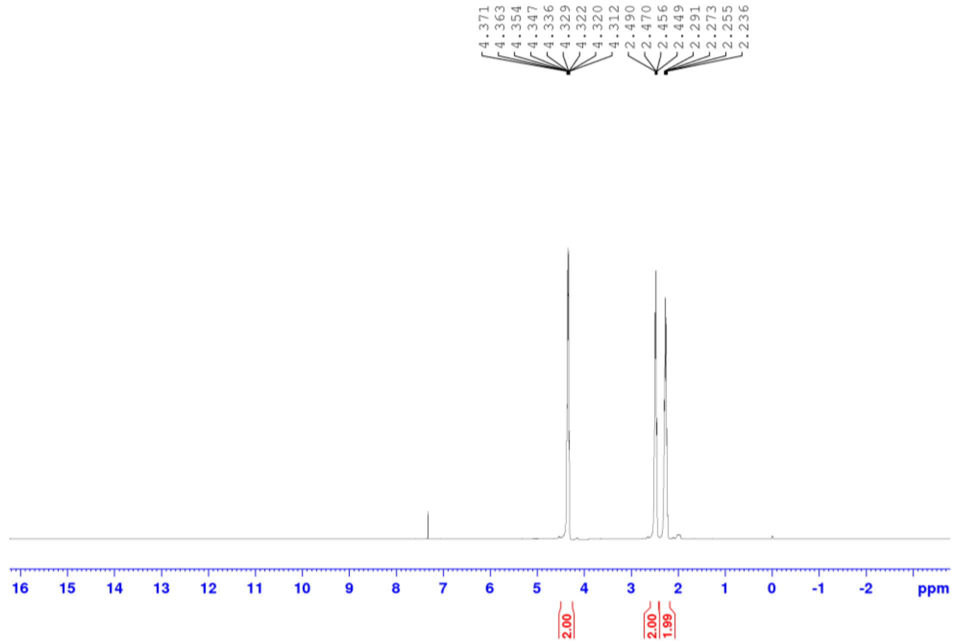

Supplementary Figure 59. ^{1}H NMR spectra of compound **3h**.

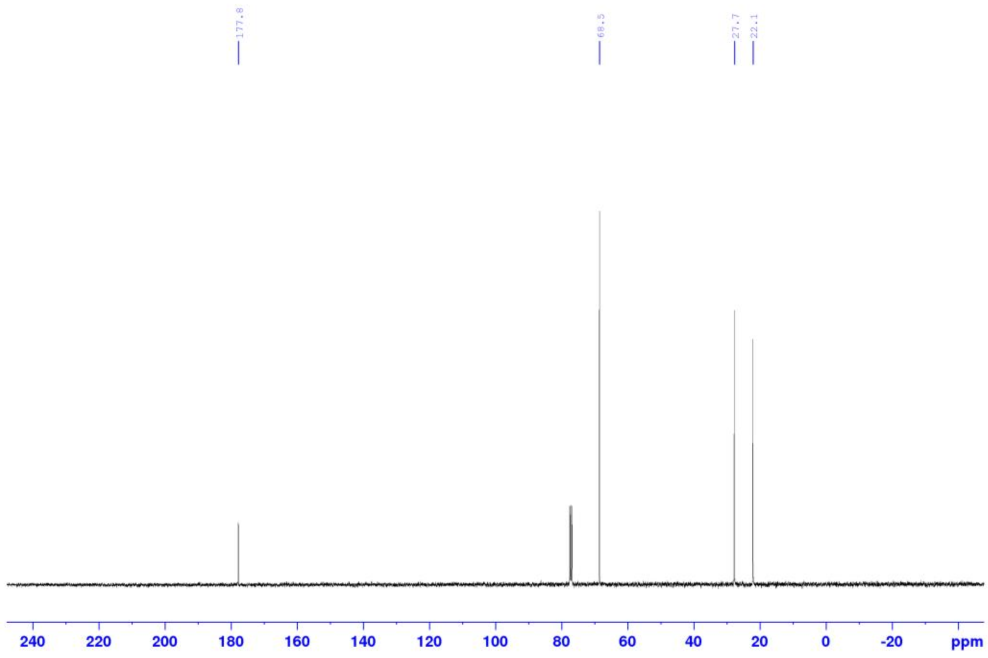

Supplementary Figure 60. ^{13}C NMR spectra of compound **3h**.

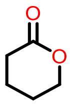
3i

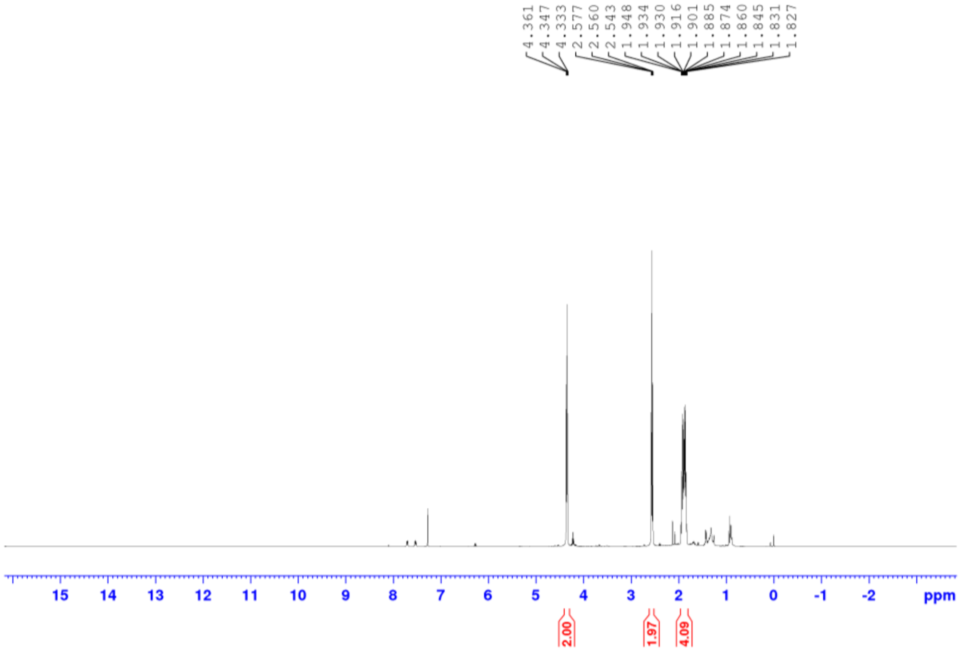

Supplementary Figure 61. ^{1}H NMR spectra of compound **3i**.

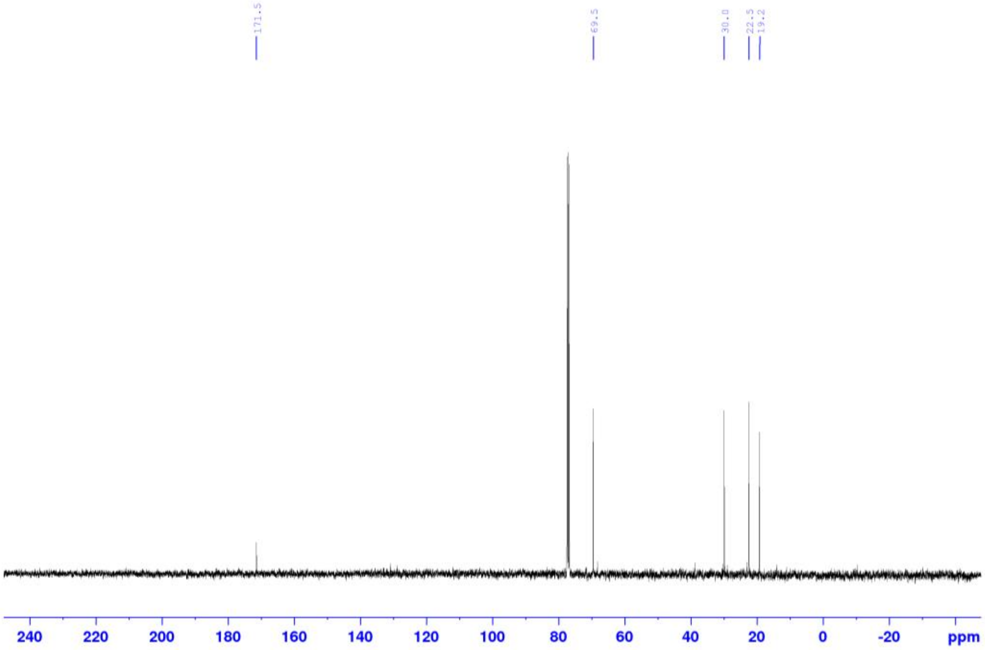

Supplementary Figure 62. ^{13}C NMR spectra of compound **3i**.

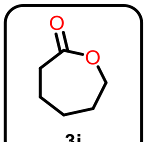

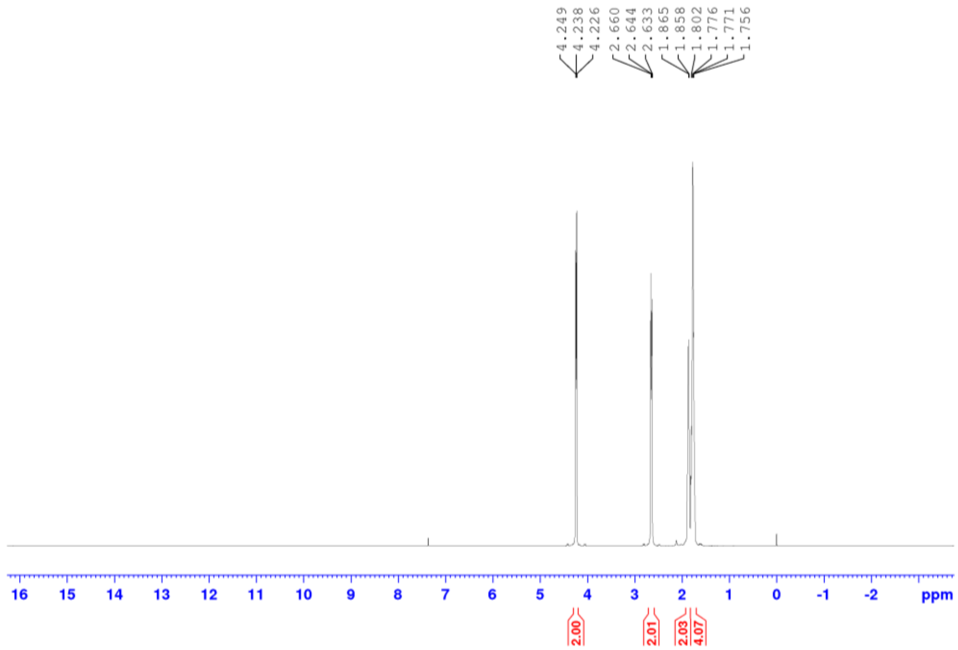

Supplementary Figure 63. ^{1}H NMR spectra of compound **3j**.

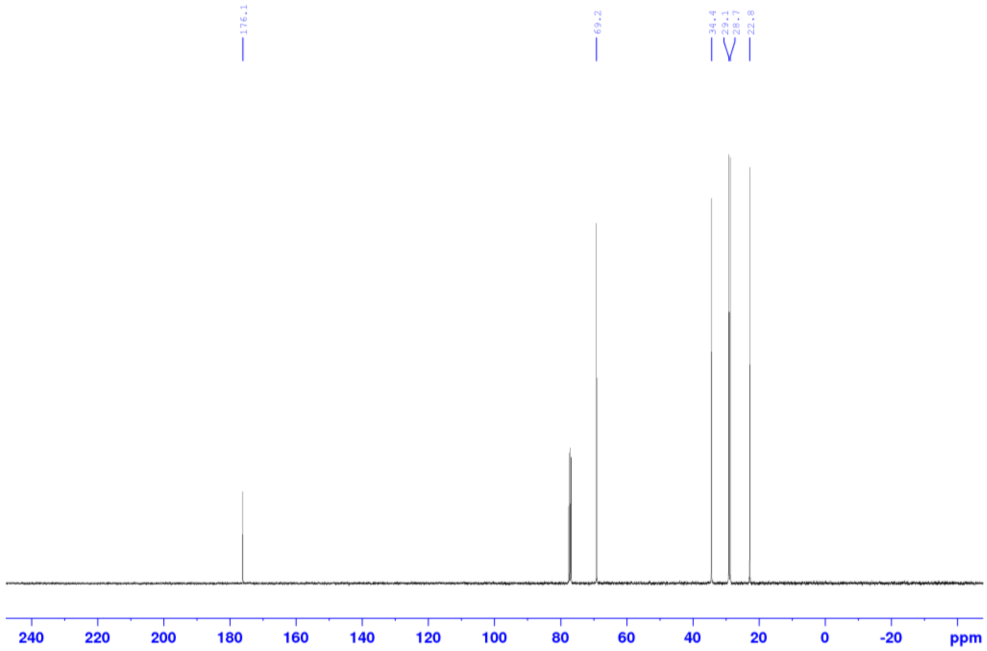

Supplementary Figure 64. ^{13}C NMR spectra of compound **3j**.

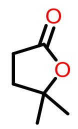

3k

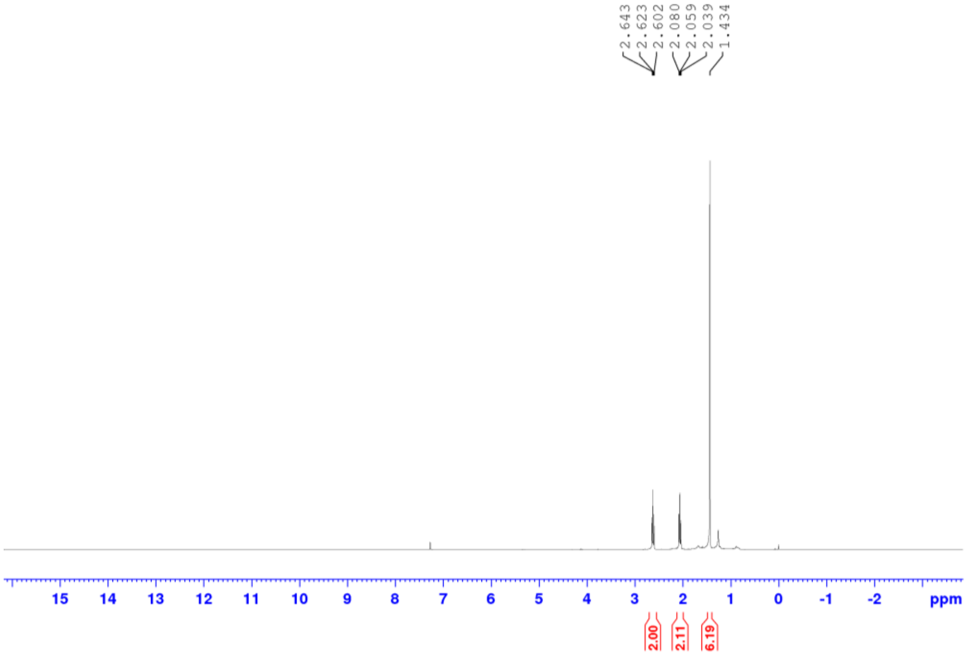

Supplementary Figure 65. ^{1}H NMR spectra of compound **3k**.

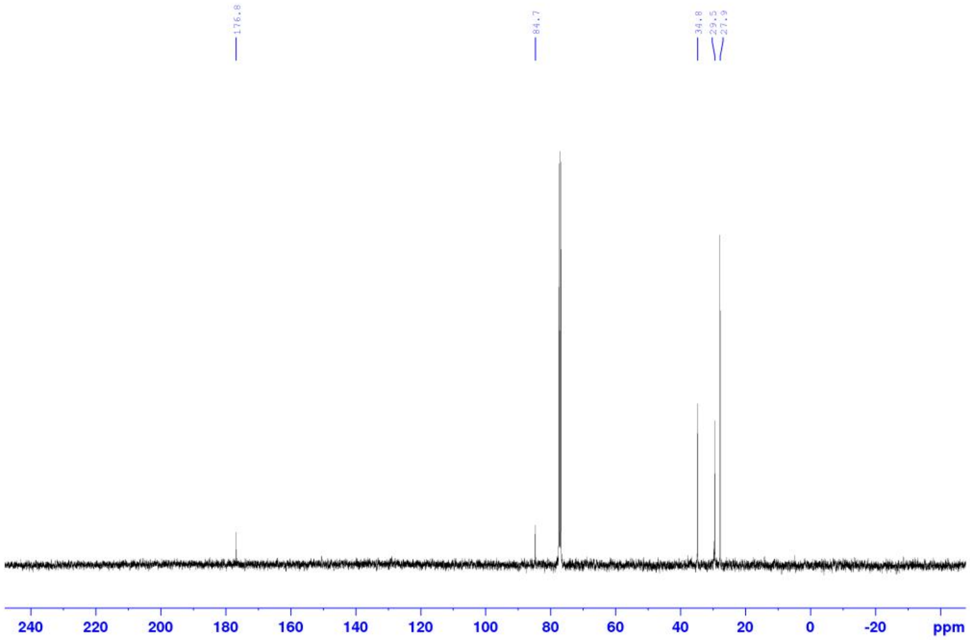

Supplementary Figure 66. ^{13}C NMR spectra of compound **3k**.

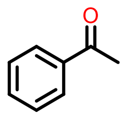

4aa

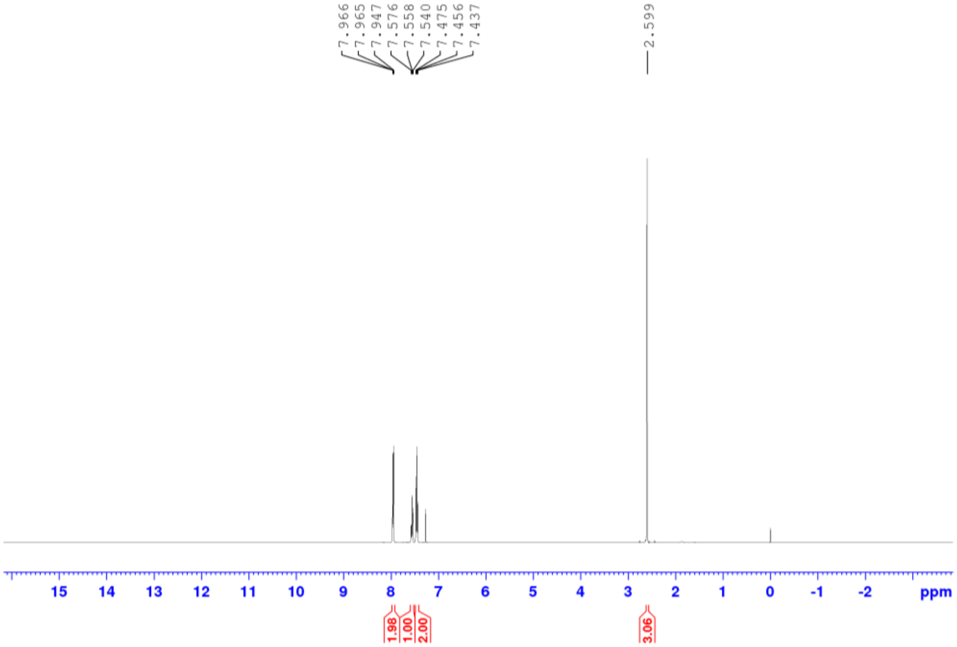

Supplementary Figure 67. ^{1}H NMR spectra of compound **4aa**.

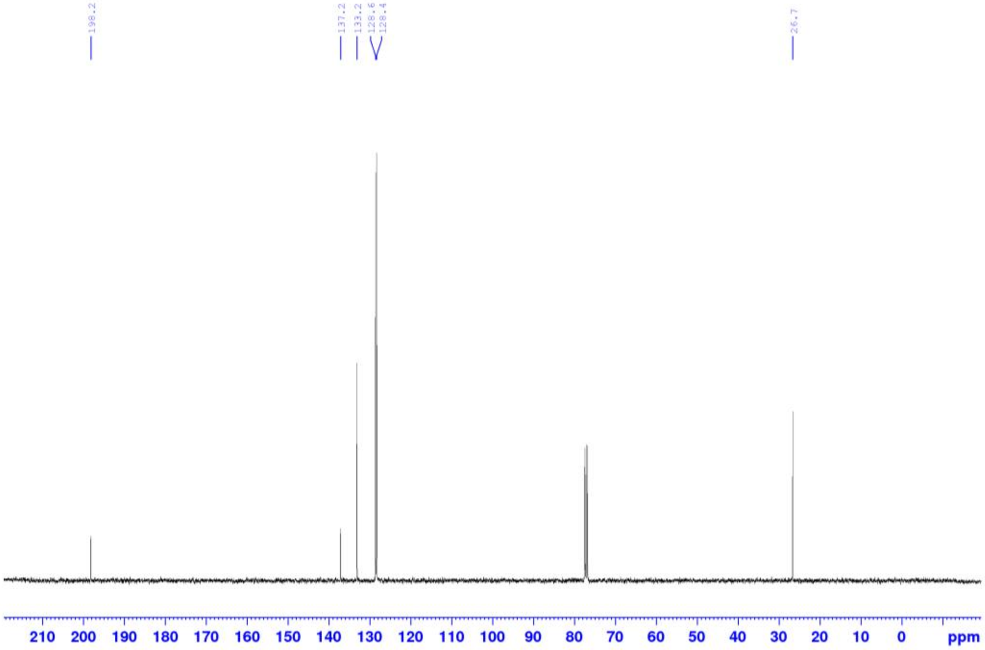

Supplementary Figure 68. ^{13}C NMR spectra of compound **4aa**.

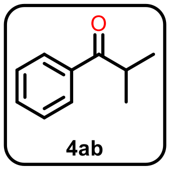

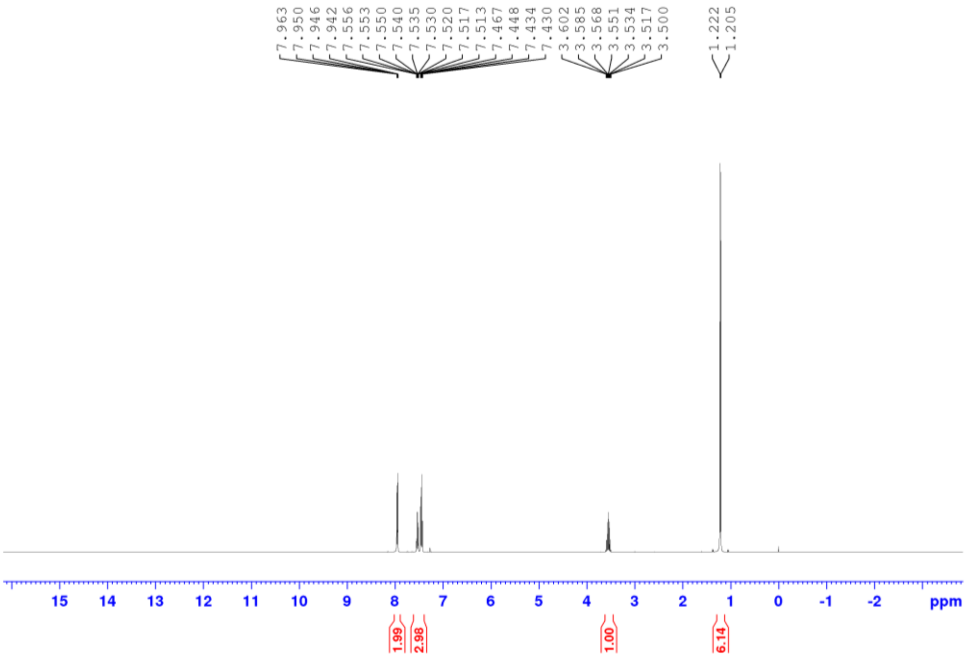

Supplementary Figure 69. ^{1}H NMR spectra of compound **4ab**.

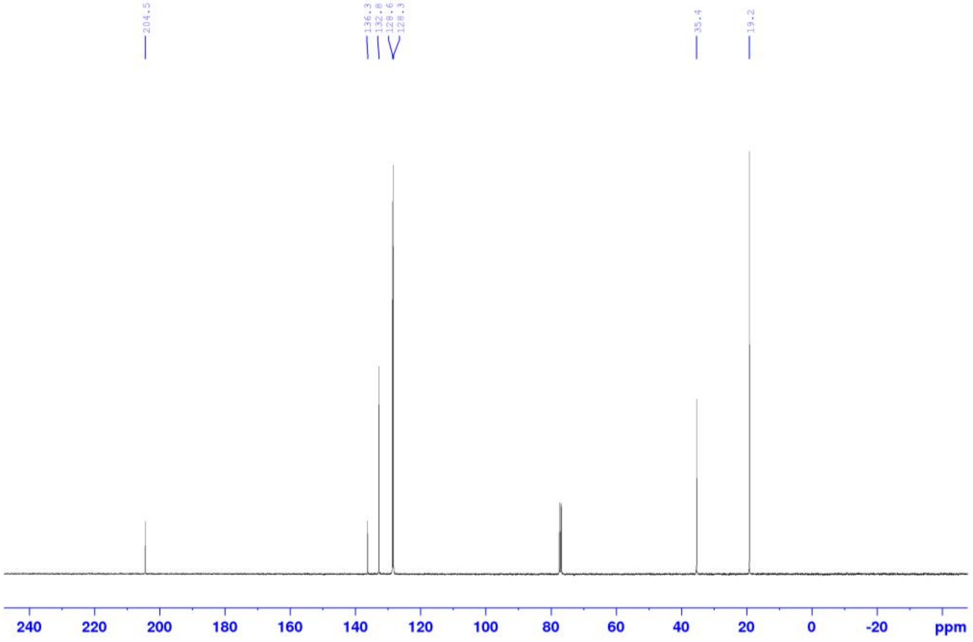

Supplementary Figure 70. ^{13}C NMR spectra of compound **4ab**.

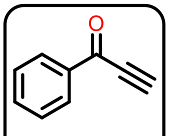

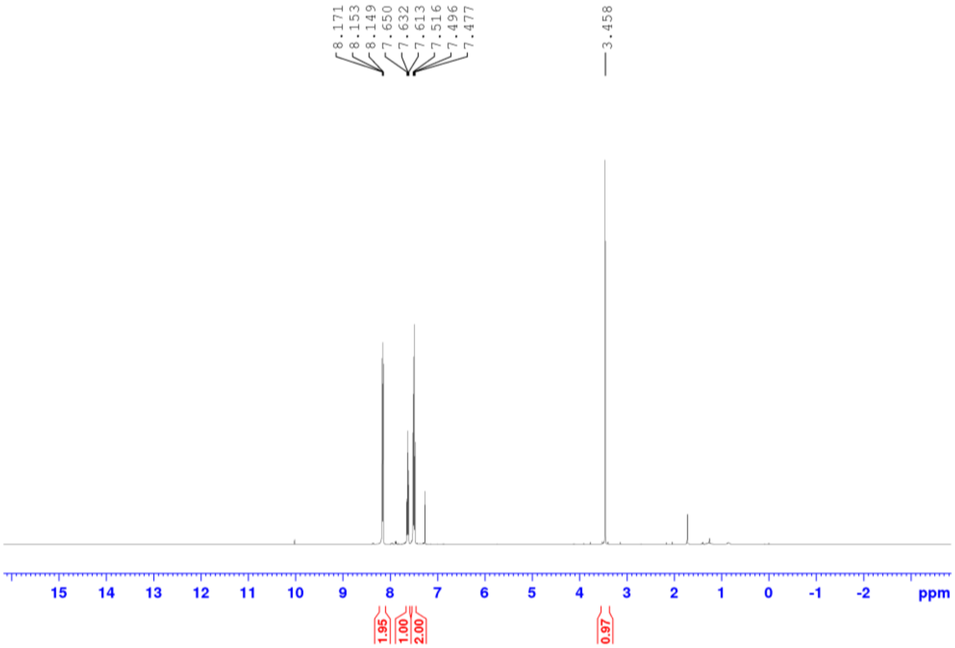

Supplementary Figure 71. ^{1}H NMR spectra of compound **4ac**.

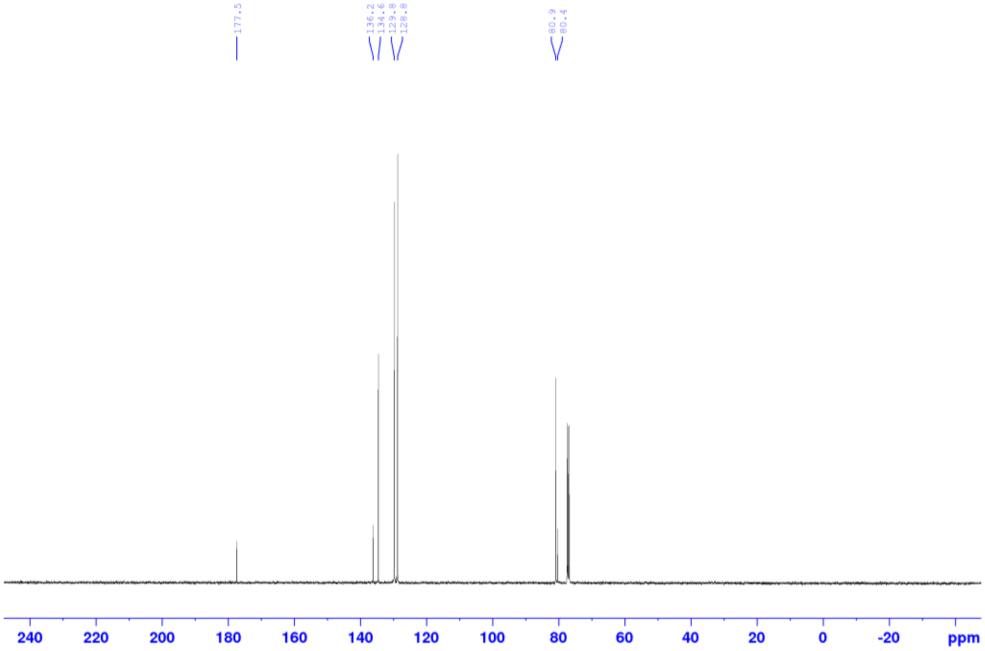

Supplementary Figure 72. ^{13}C NMR spectra of compound **4ac**.

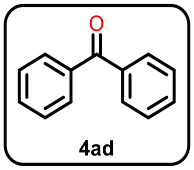

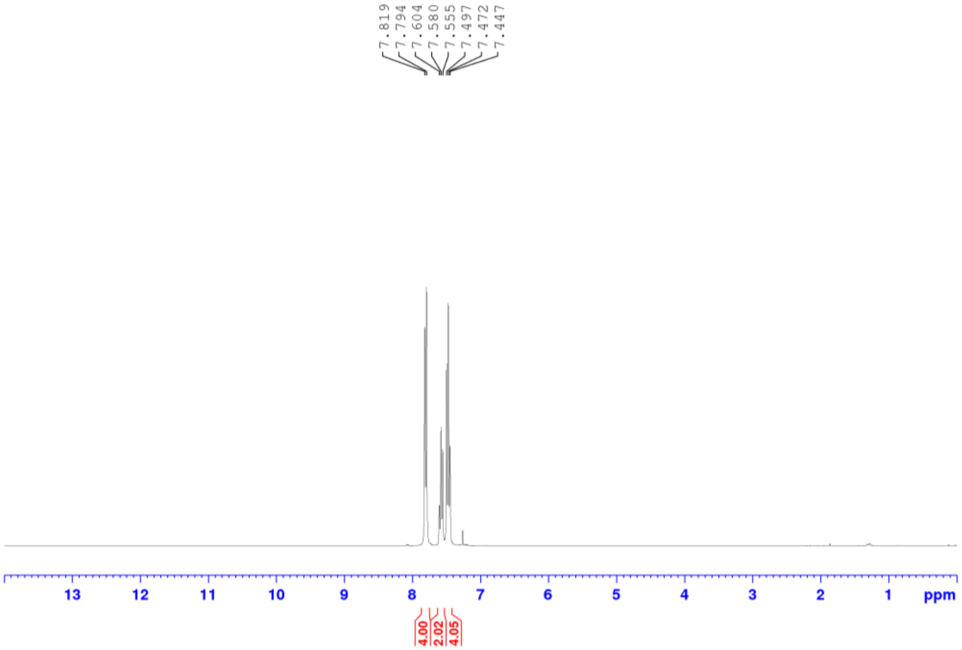

Supplementary Figure 73. ^{1}H NMR spectra of compound **4ad**.

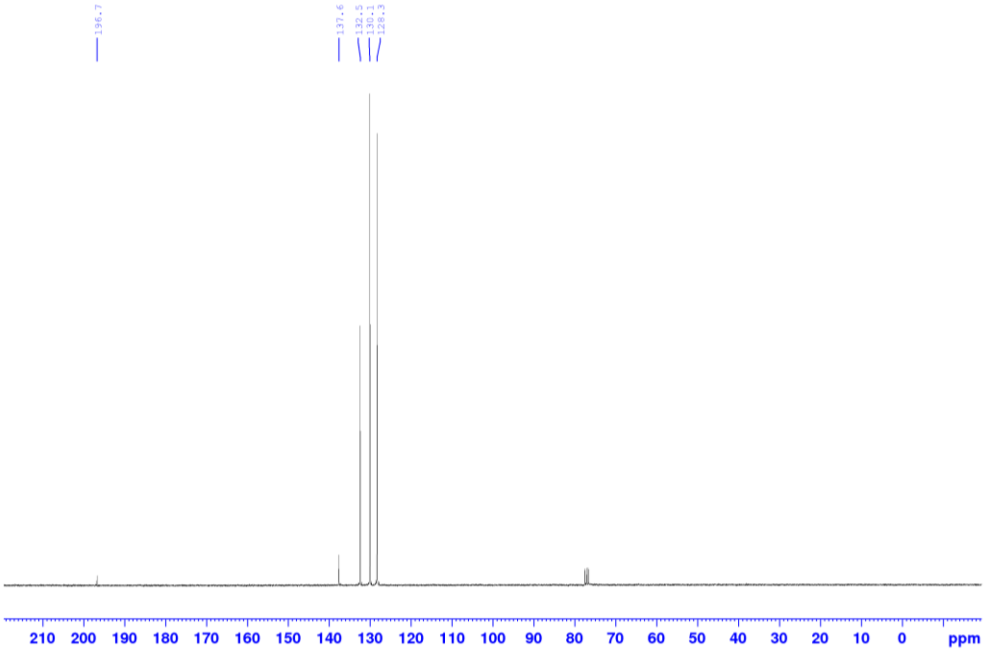

Supplementary Figure 74. ^{13}C NMR spectra of compound **4ad**.

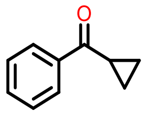

4ae

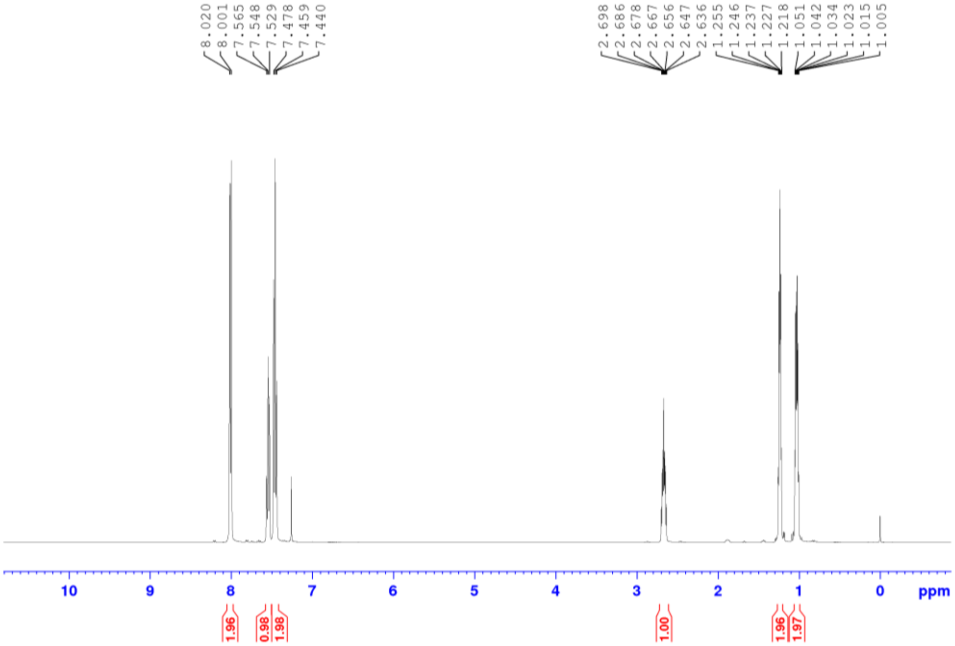

Supplementary Figure 75. ^{1}H NMR spectra of compound **4ae**.

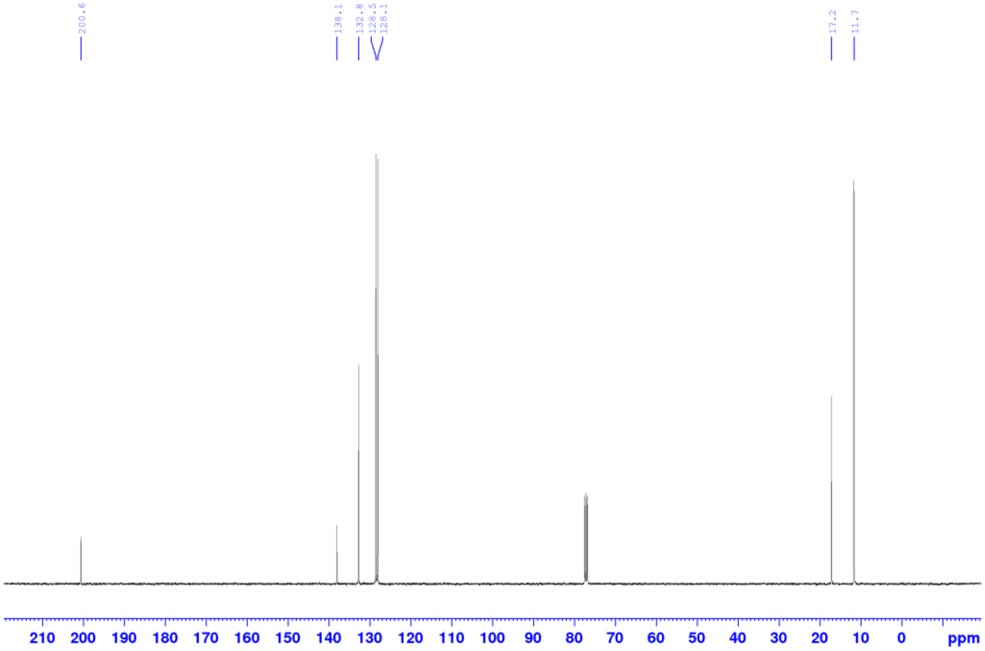

Supplementary Figure 76. ^{13}C NMR spectra of compound **4ae**.

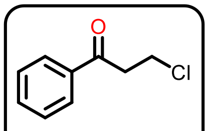

4af

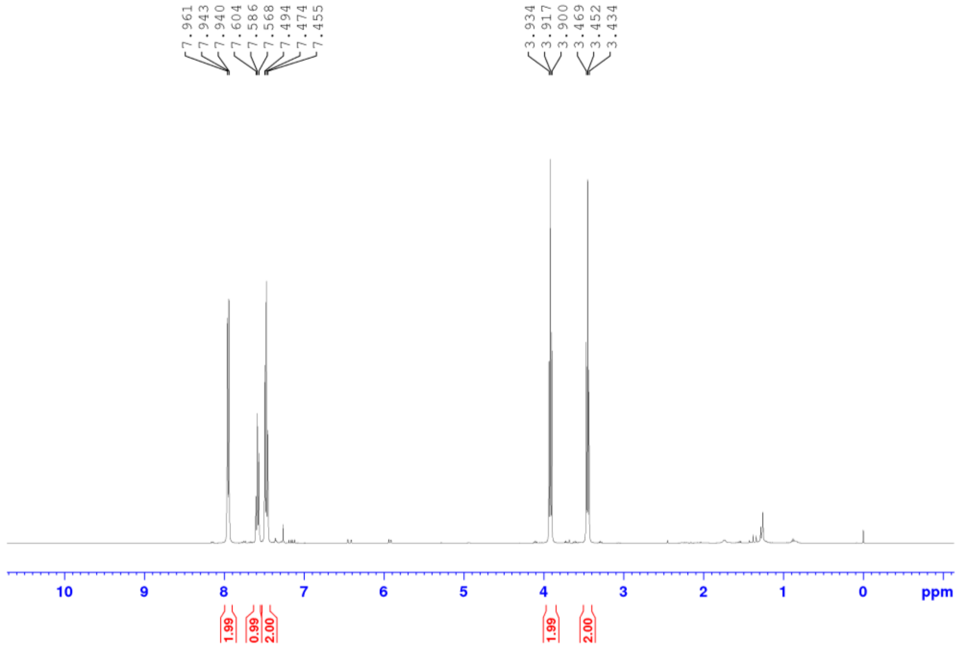

Supplementary Figure 77. ^{1}H NMR spectra of compound **4af**.

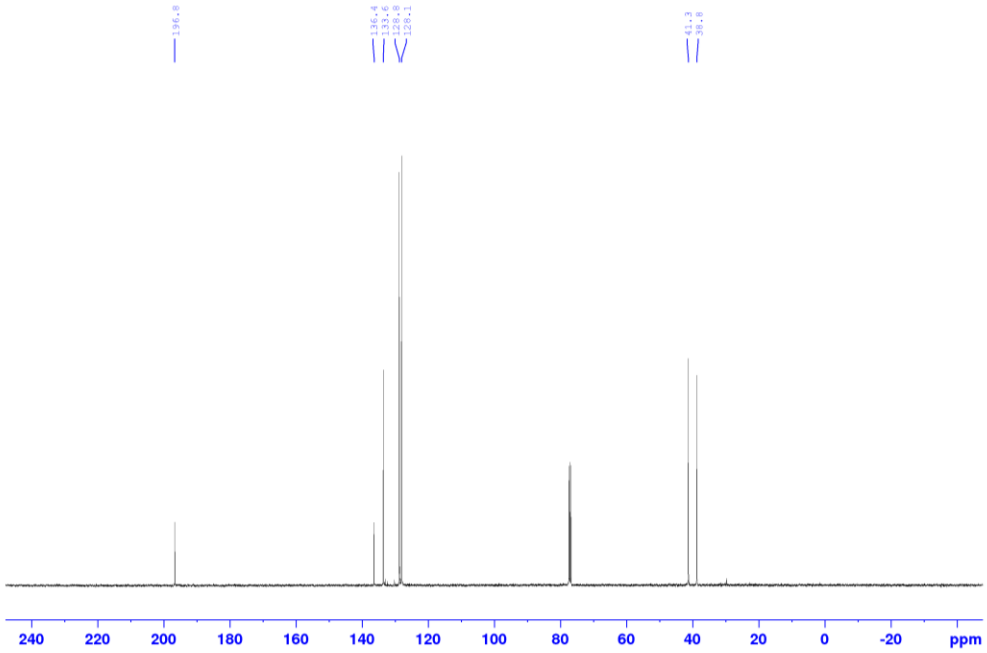

Supplementary Figure 78. ^{13}C NMR spectra of compound **4af**.

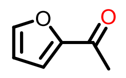

4b

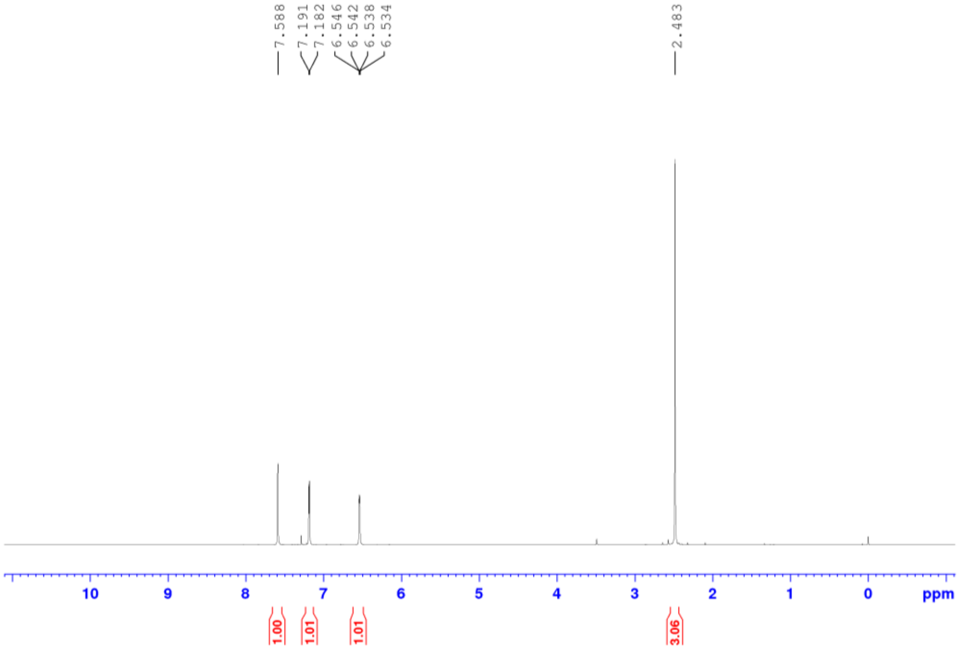

Supplementary Figure 79. ^{1}H NMR spectra of compound **4b**.

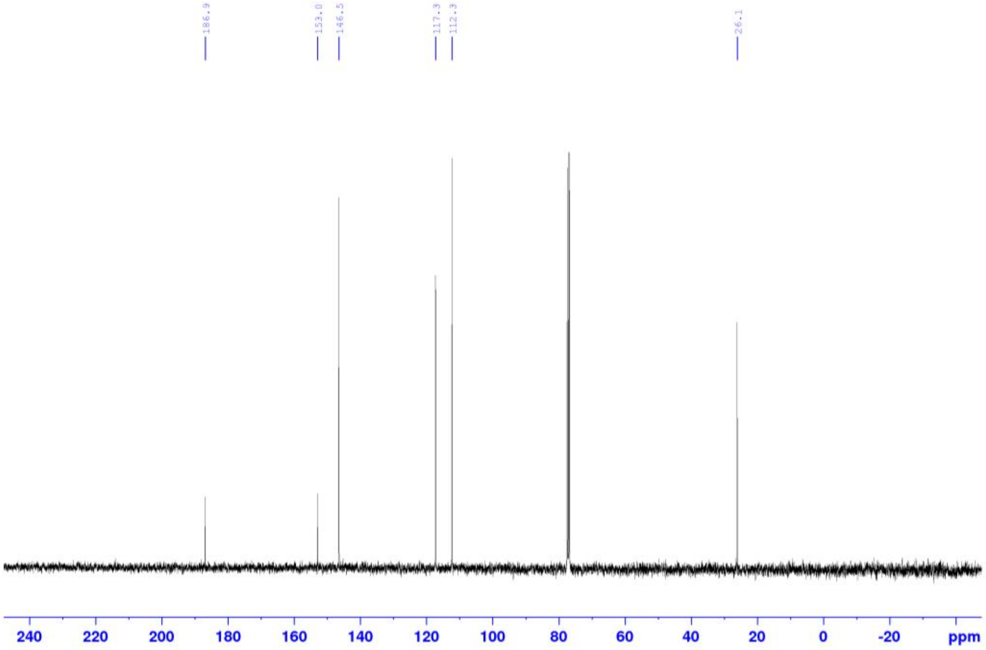

Supplementary Figure 80. ^{13}C NMR spectra of compound **4b**.

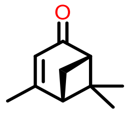

4c

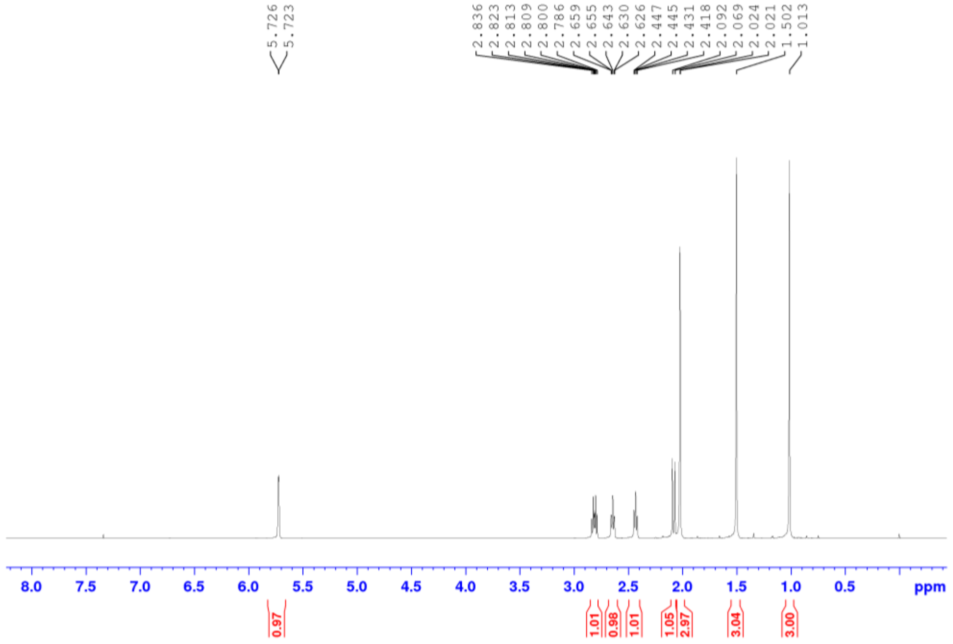

Supplementary Figure 81. ^{1}H NMR spectra of compound **4c**.

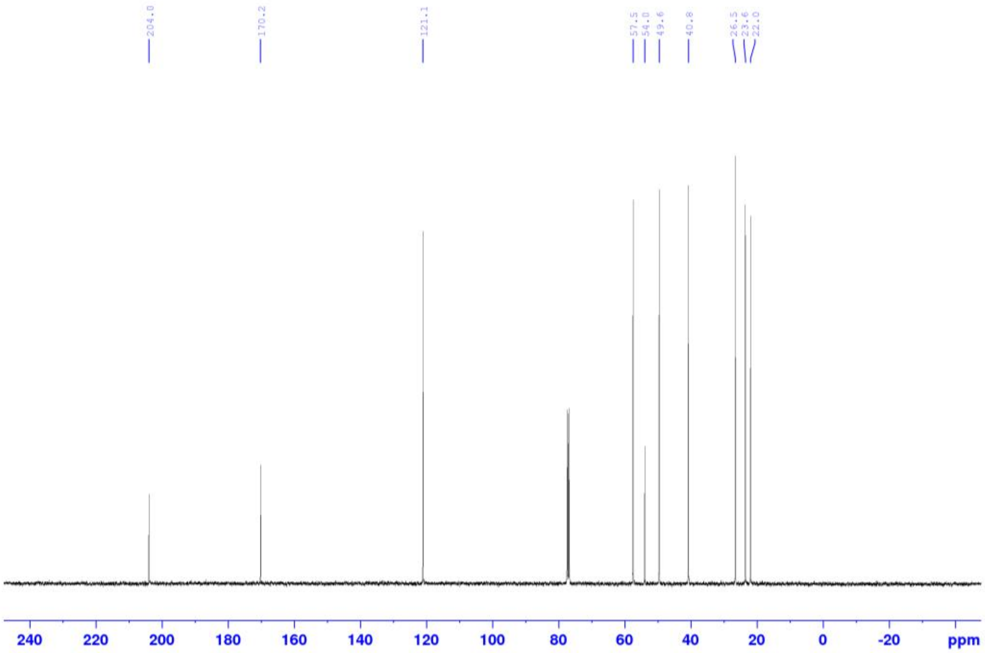

Supplementary Figure 82. ^{13}C NMR spectra of compound **4c**.

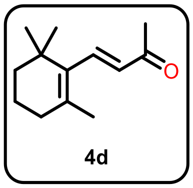

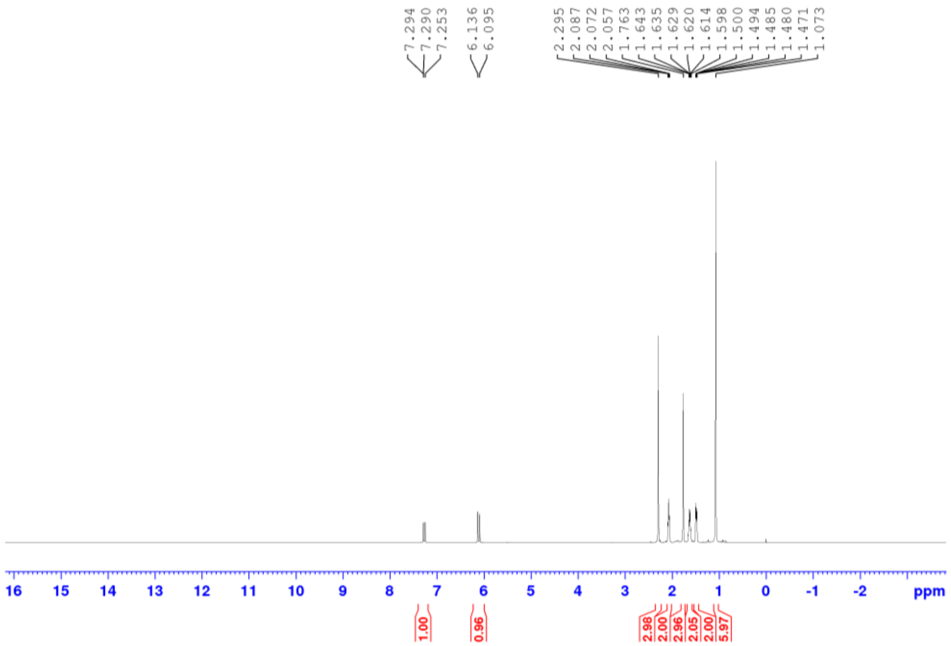

Supplementary Figure 83. ^{1}H NMR spectra of compound **4d**.

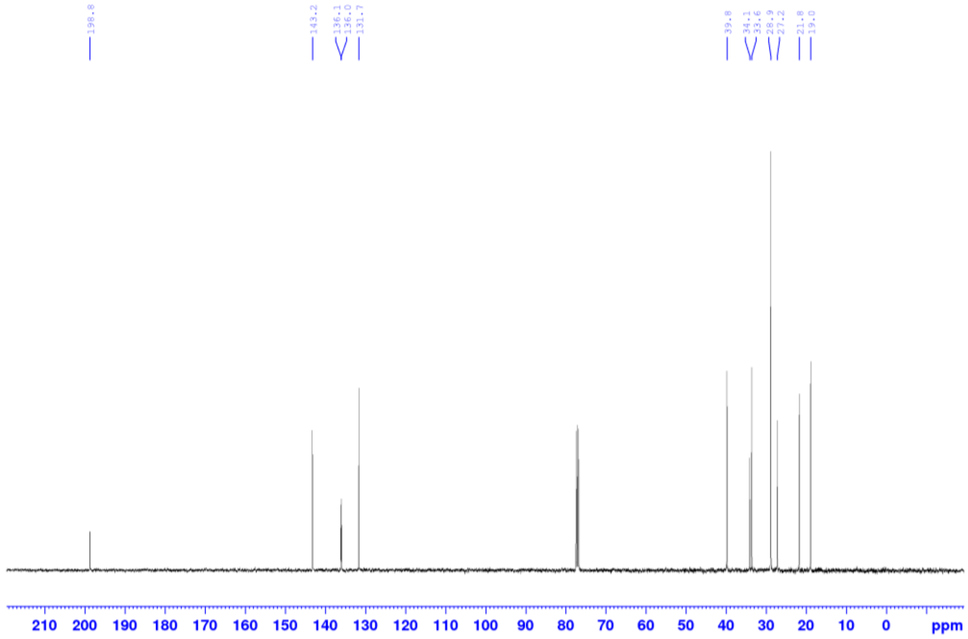

Supplementary Figure 84. ^{13}C NMR spectra of compound **4d**.

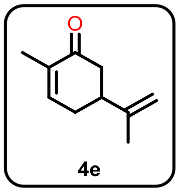

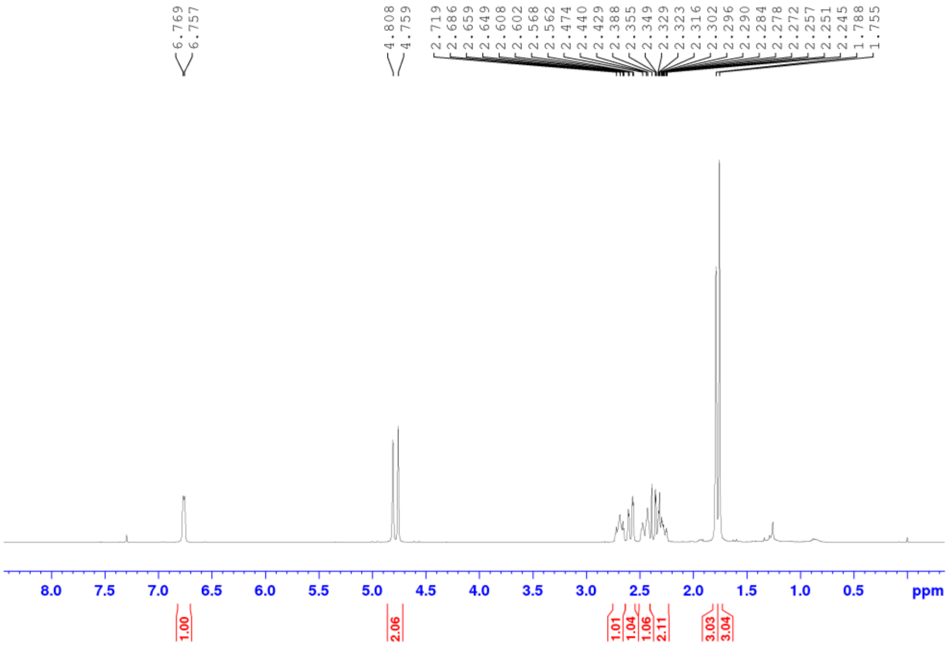

Supplementary Figure 85. ^{1}H NMR spectra of compound **4e**.

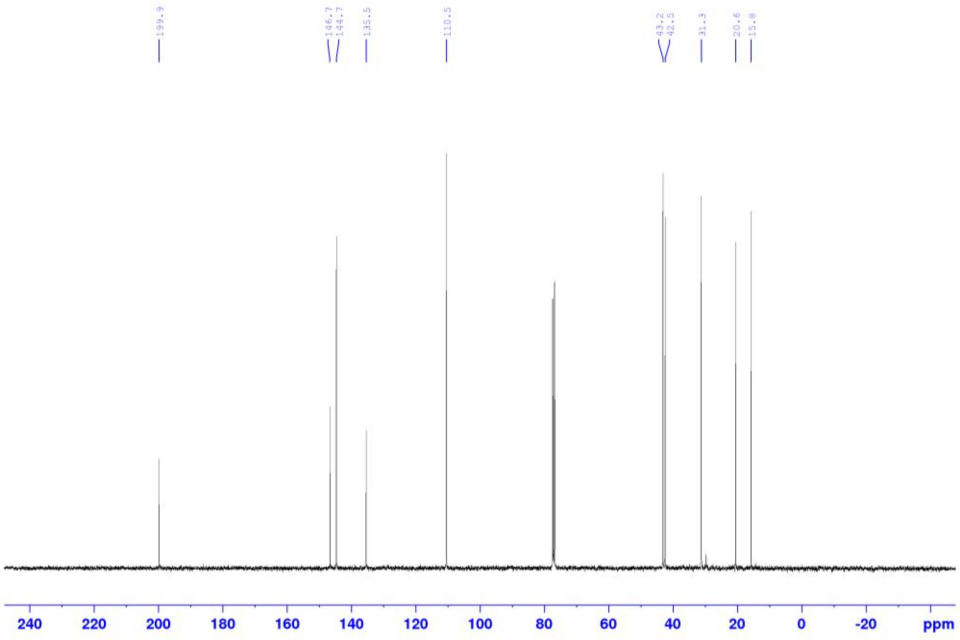

Supplementary Figure 86. ^{13}C NMR spectra of compound **4e**.

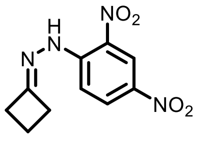

4fa

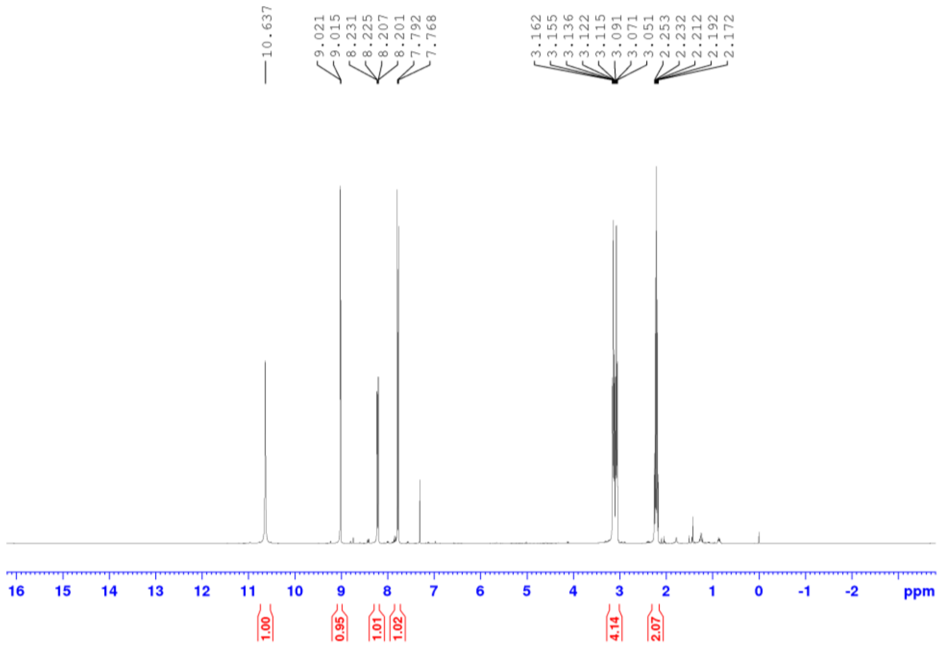

Supplementary Figure 87. ^{1}H NMR spectra of compound **4fa**.

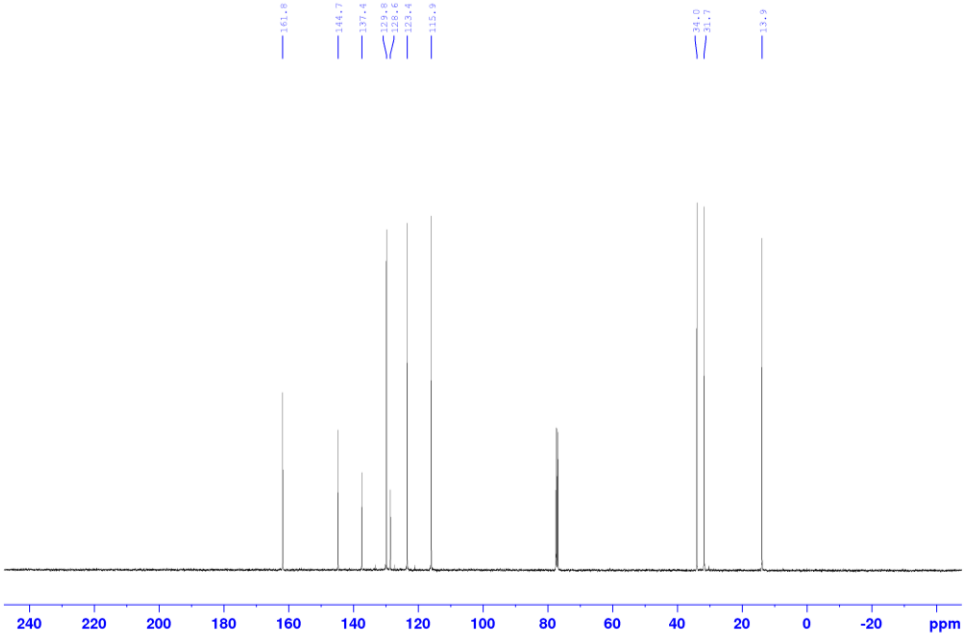

Supplementary Figure 88. ^{13}C NMR spectra of compound **4fa**.

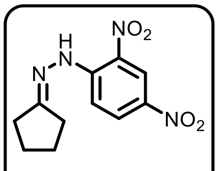

4fb

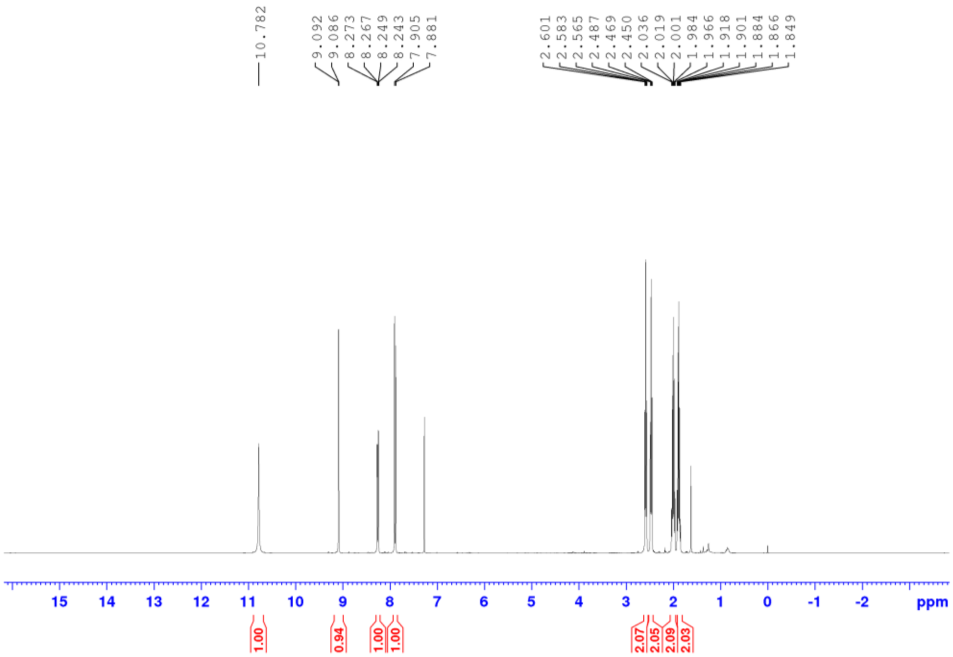

Supplementary Figure 89. ^{1}H NMR spectra of compound **4fb**.

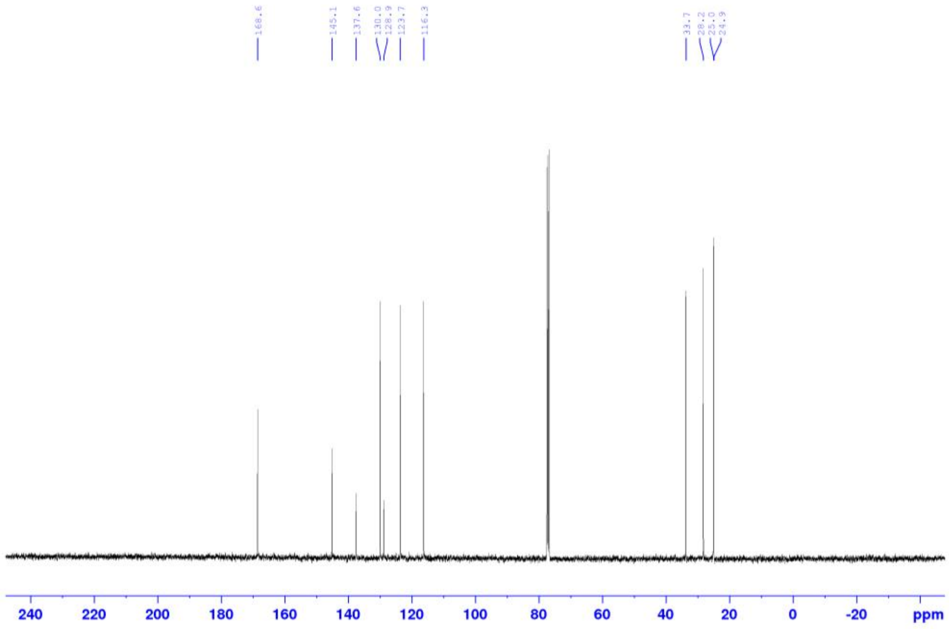

Supplementary Figure 90. ^{13}C NMR spectra of compound **4fb**.

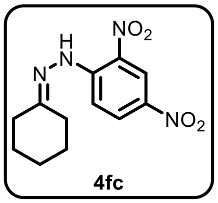

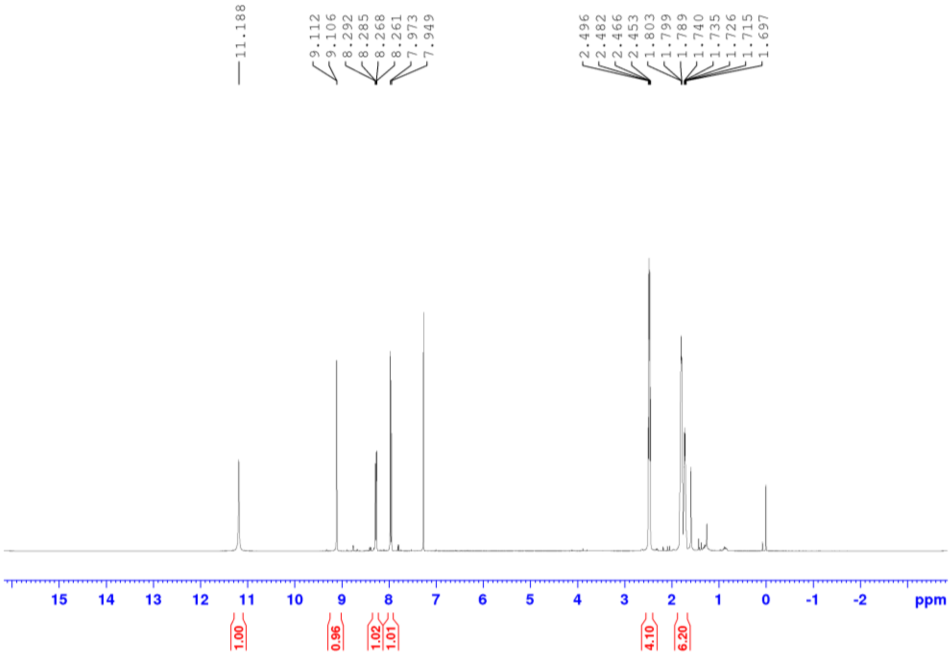

Supplementary Figure 91. ^{1}H NMR spectra of compound **4fc**.

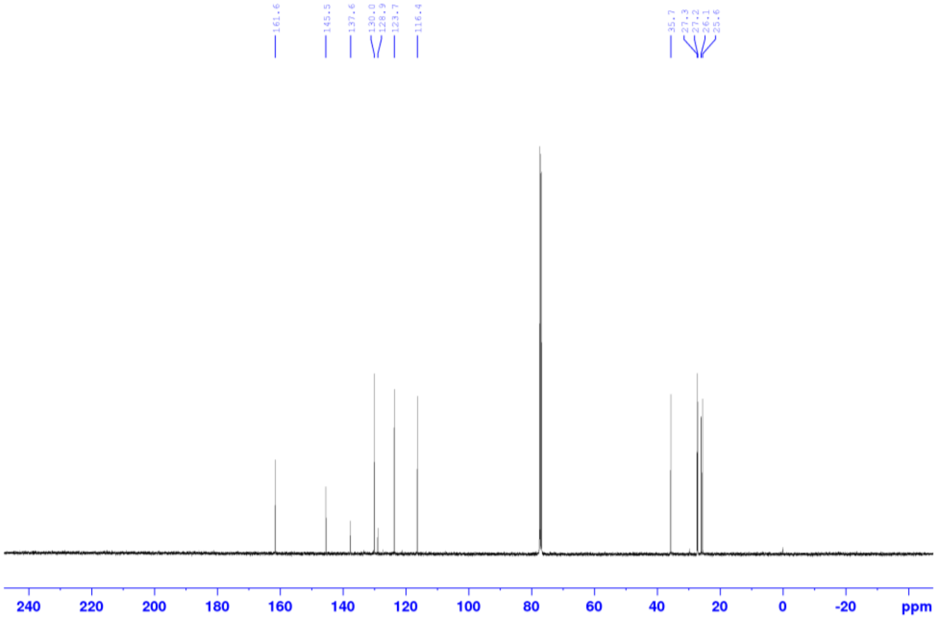

Supplementary Figure 92. ^{13}C NMR spectra of compound **4fc**.

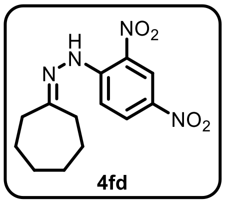

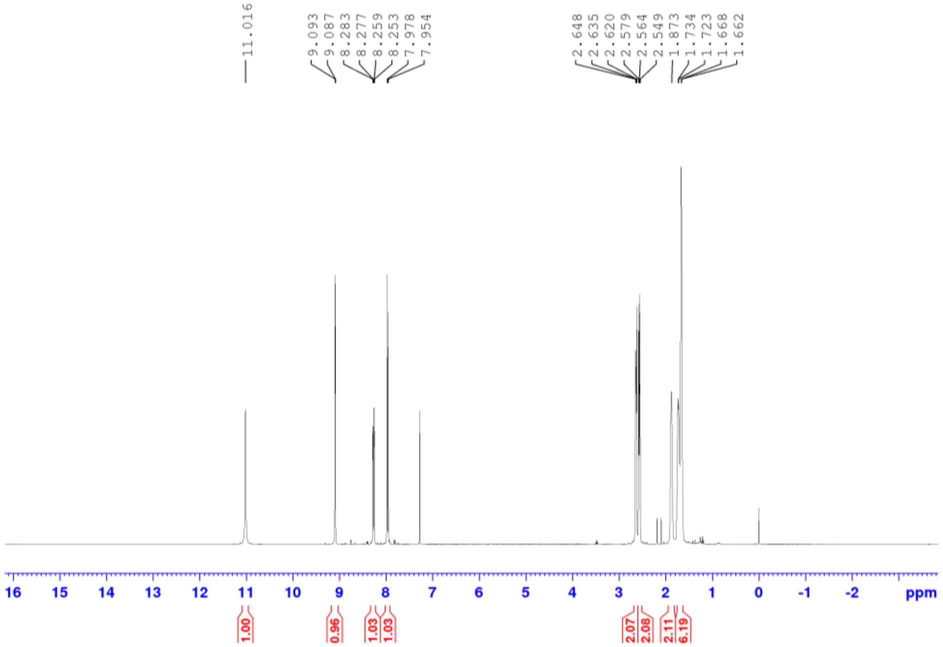

Supplementary Figure 93. ^{1}H NMR spectra of compound **4fd**.

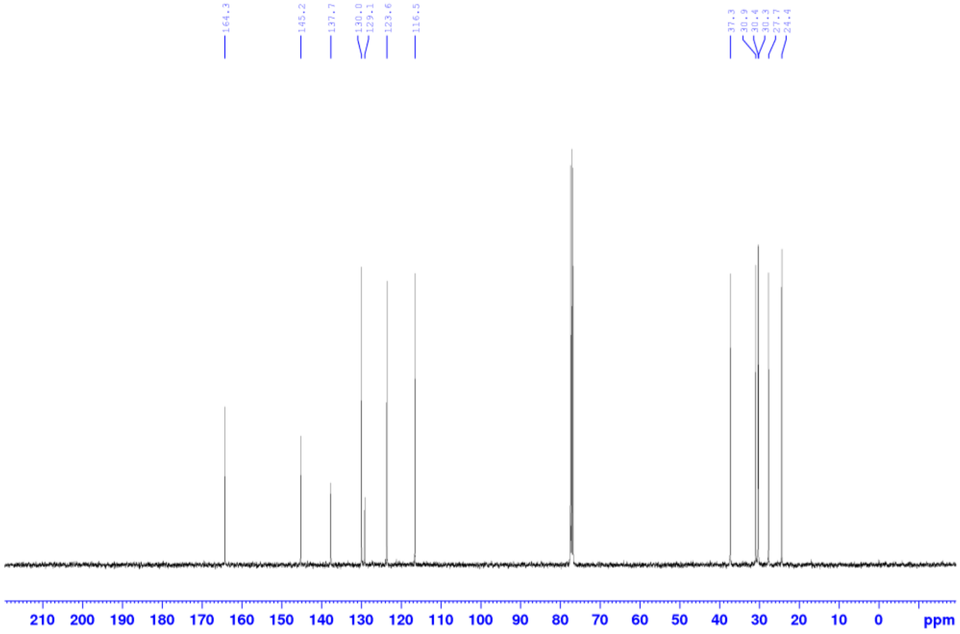

Supplementary Figure 94. ^{13}C NMR spectra of compound **4fd**.

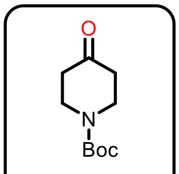

4ga

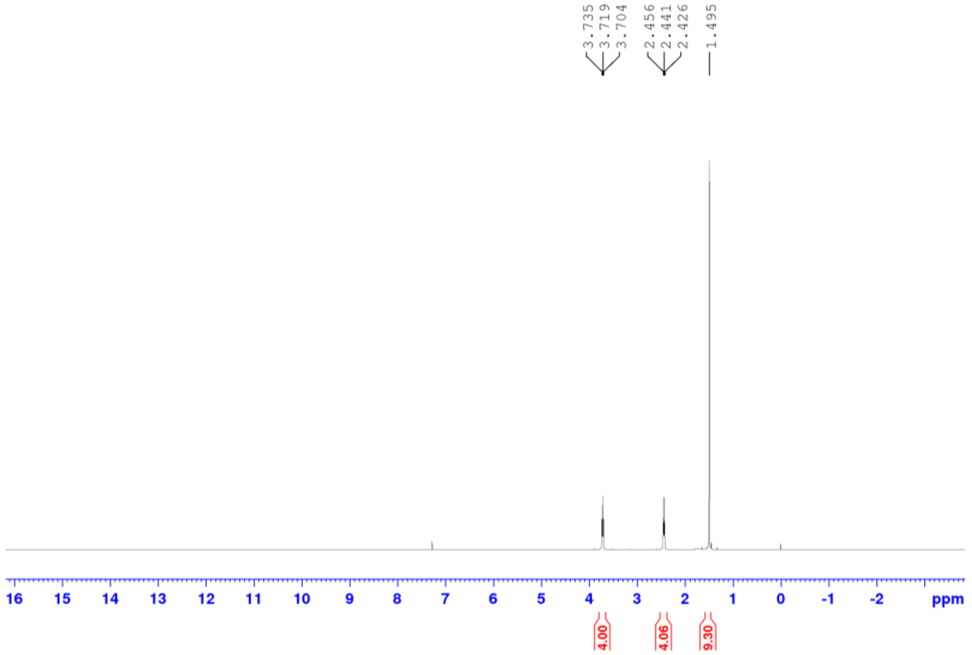

Supplementary Figure 95. ^{1}H NMR spectra of compound **4ga**.

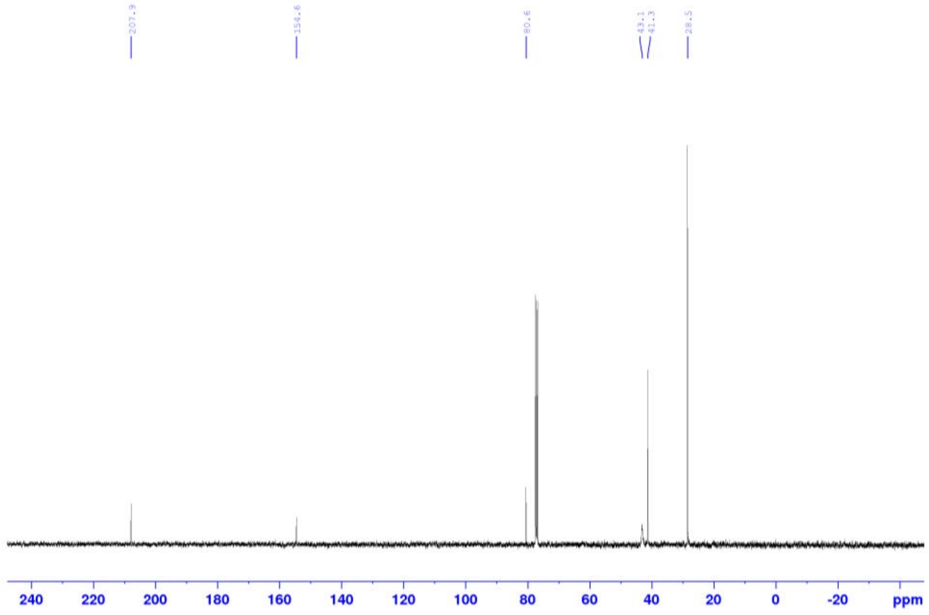

Supplementary Figure 96. ^{13}C NMR spectra of compound **4ga**.

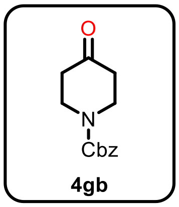

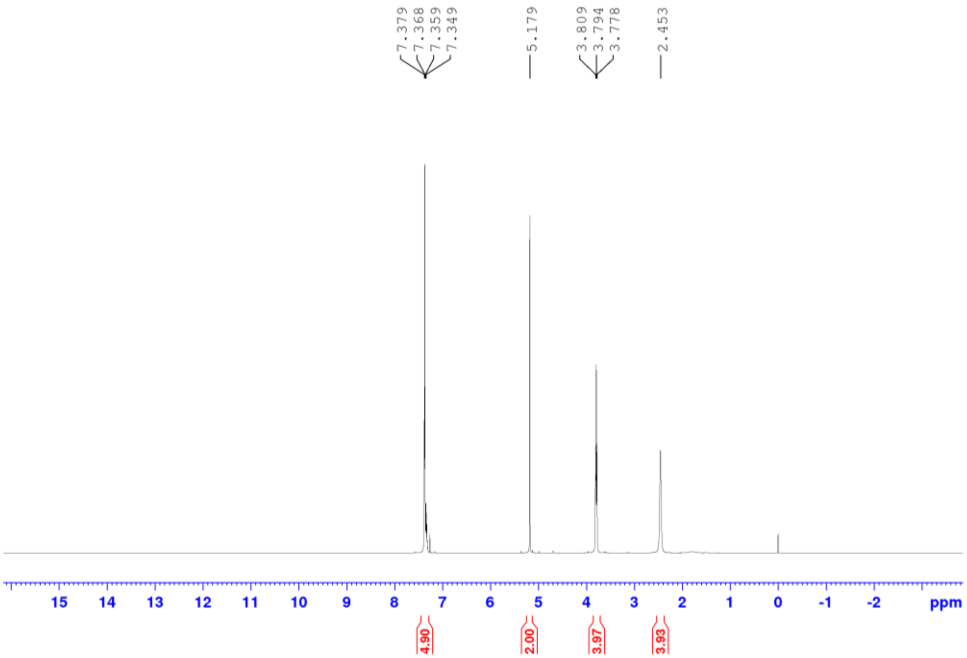

Supplementary Figure 97. ^{1}H NMR spectra of compound **4gb**.

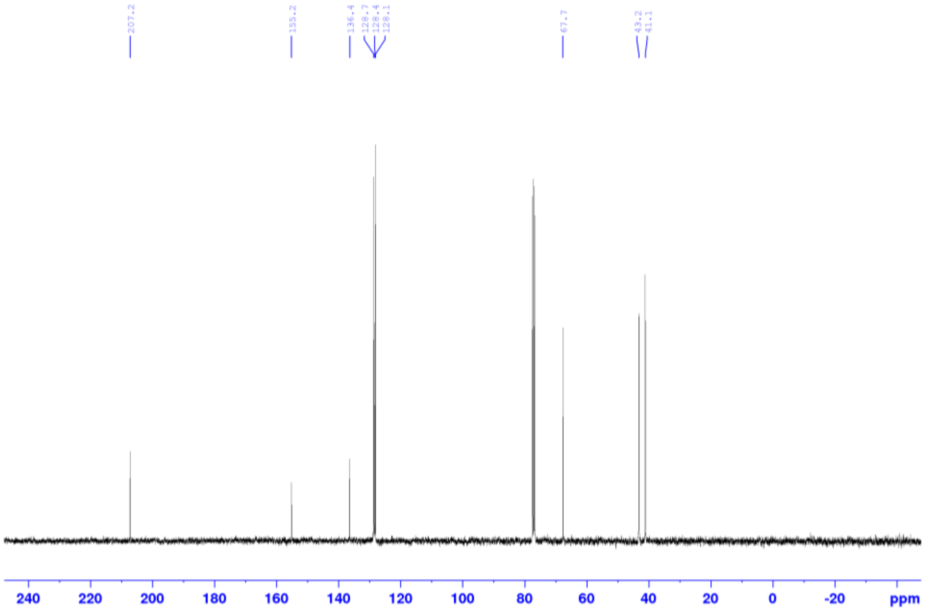

Supplementary Figure 98. ^{13}C NMR spectra of compound **4gb**.

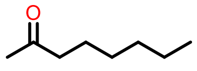

4h

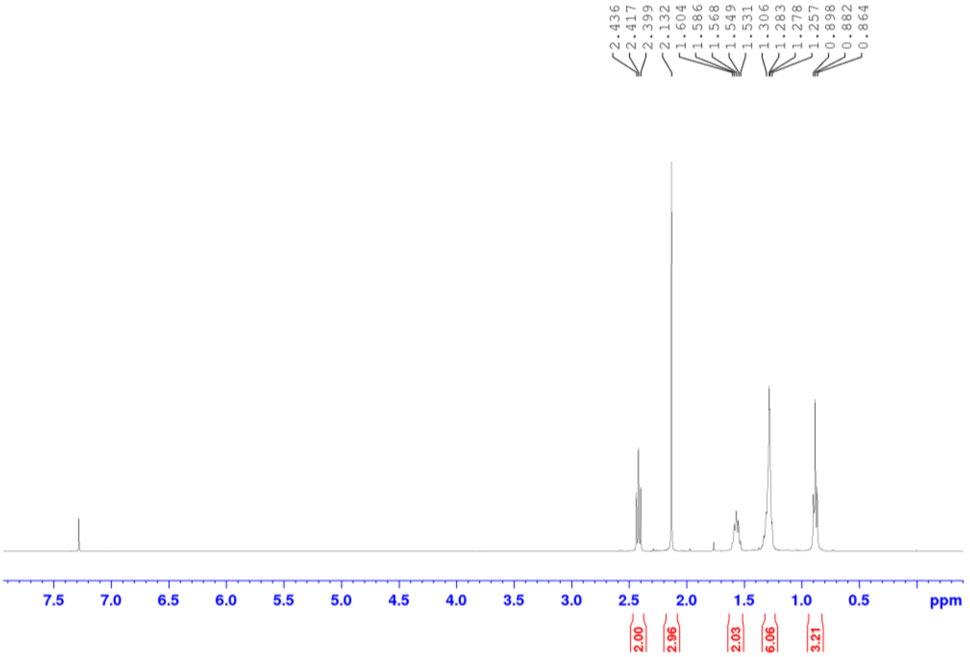

Supplementary Figure 99. ^{1}H NMR spectra of compound **4h**.

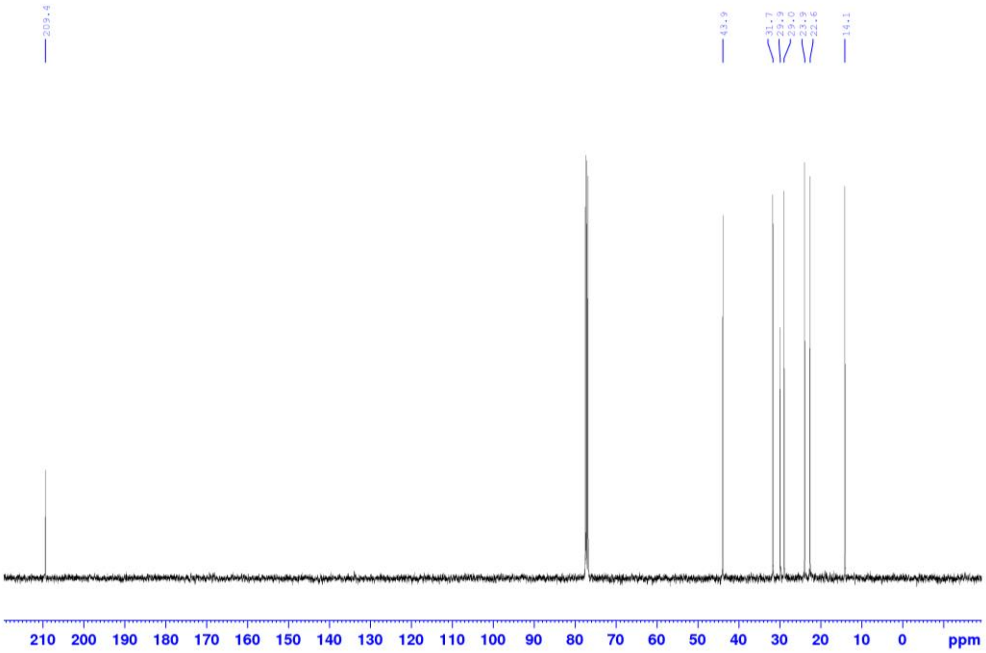

Supplementary Figure 100. ^{13}C NMR spectra of compound **4h**.

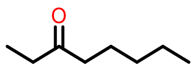

4i

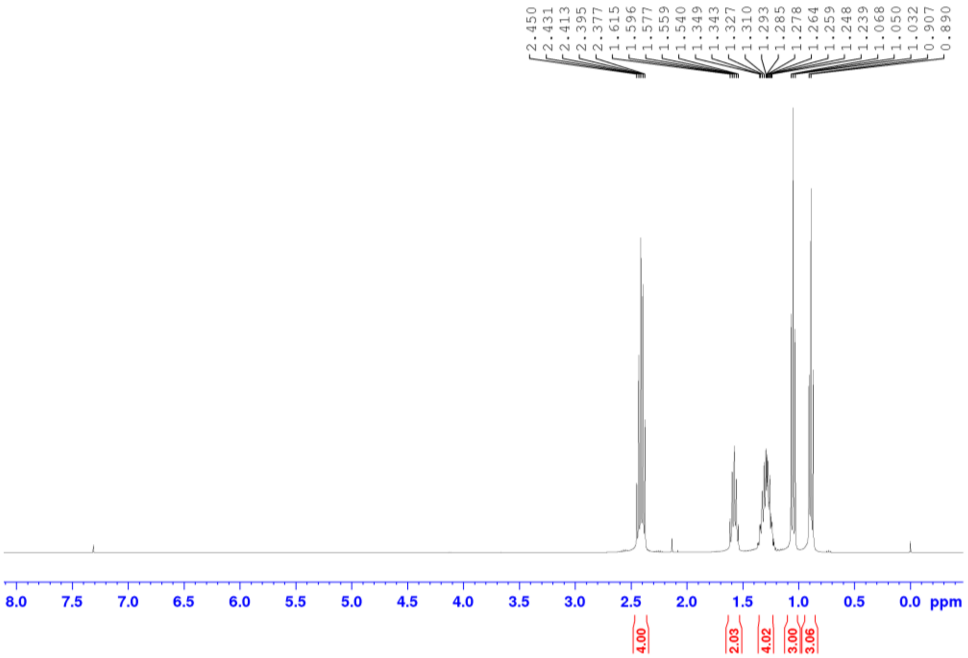

Supplementary Figure 101. ^{1}H NMR spectra of compound **4i**.

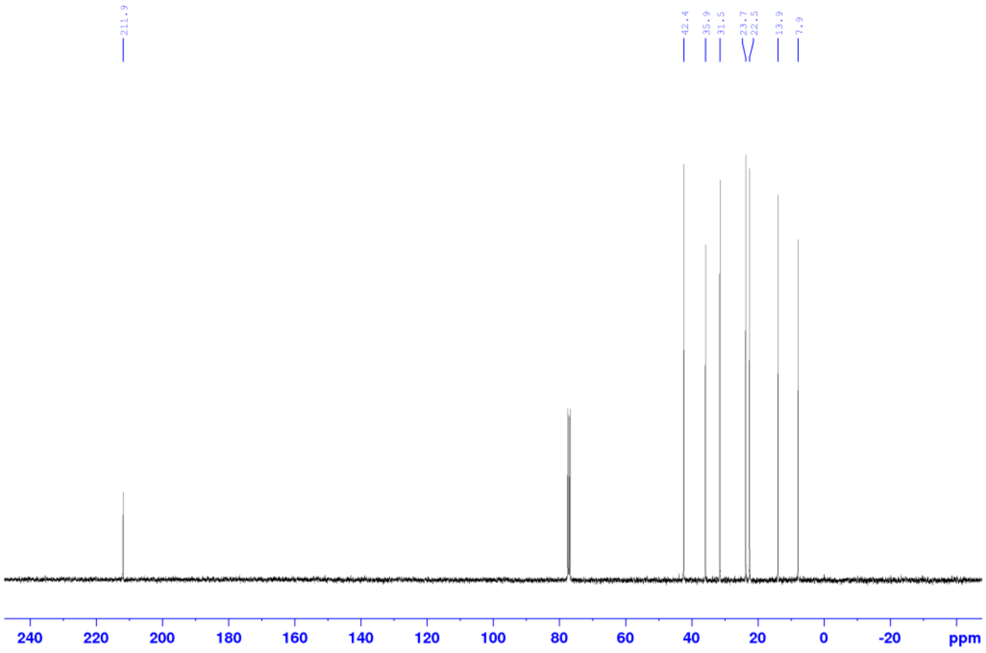

Supplementary Figure 102. ^{13}C NMR spectra of compound **4i**.

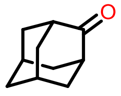

4j

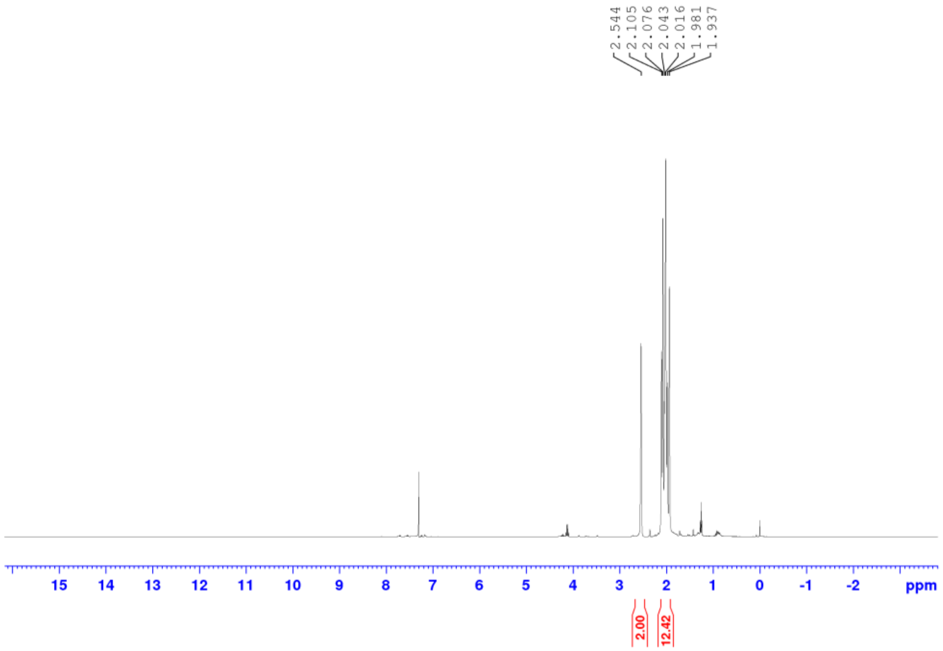

Supplementary Figure 103. ^{1}H NMR spectra of compound **4j**.

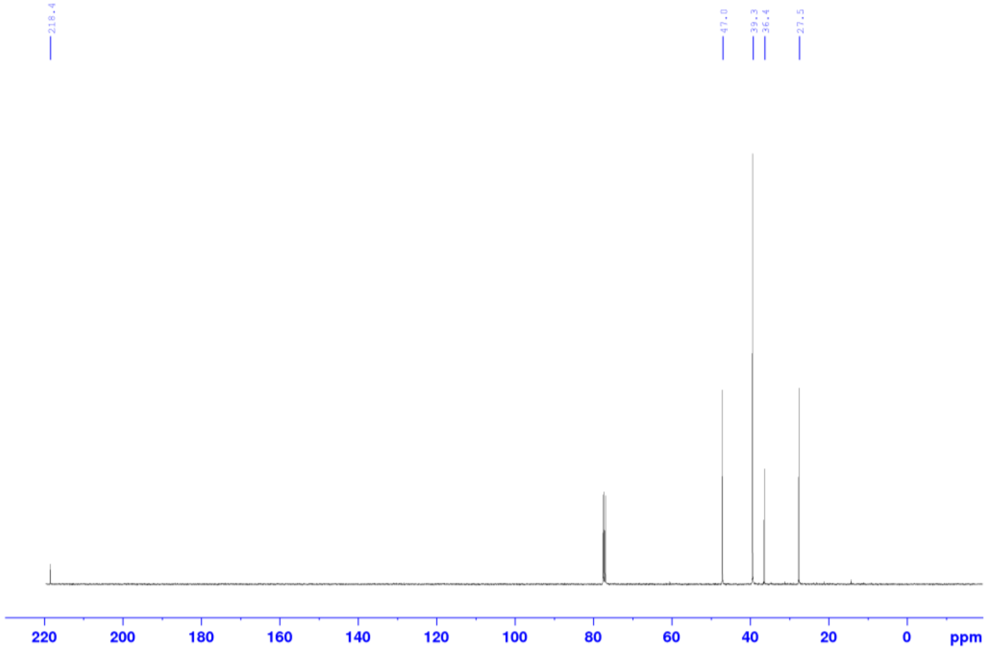

Supplementary Figure 104. ^{13}C NMR spectra of compound **4j**.

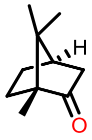

4k

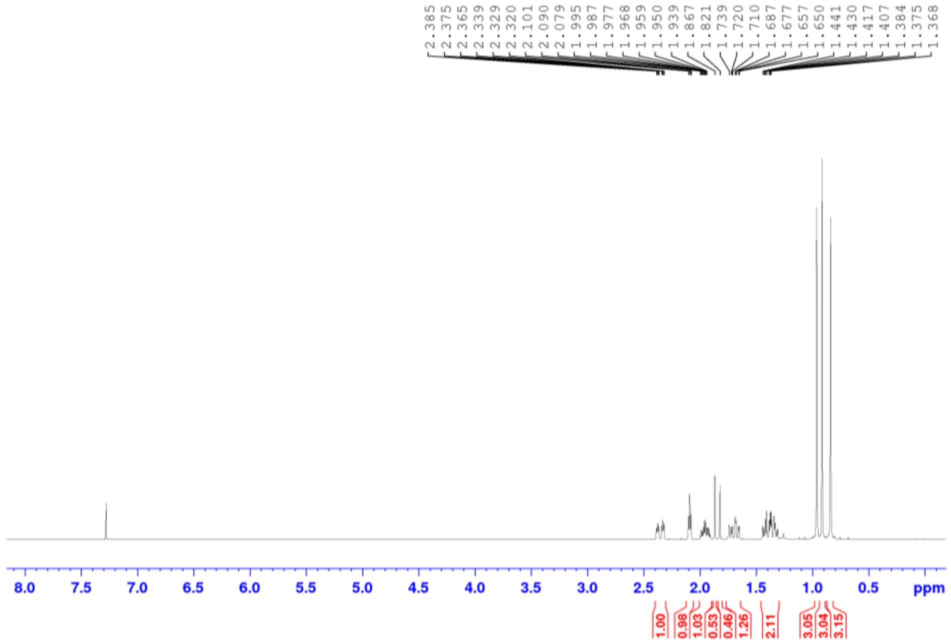

Supplementary Figure 105. ^{1}H NMR spectra of compound **4k**.

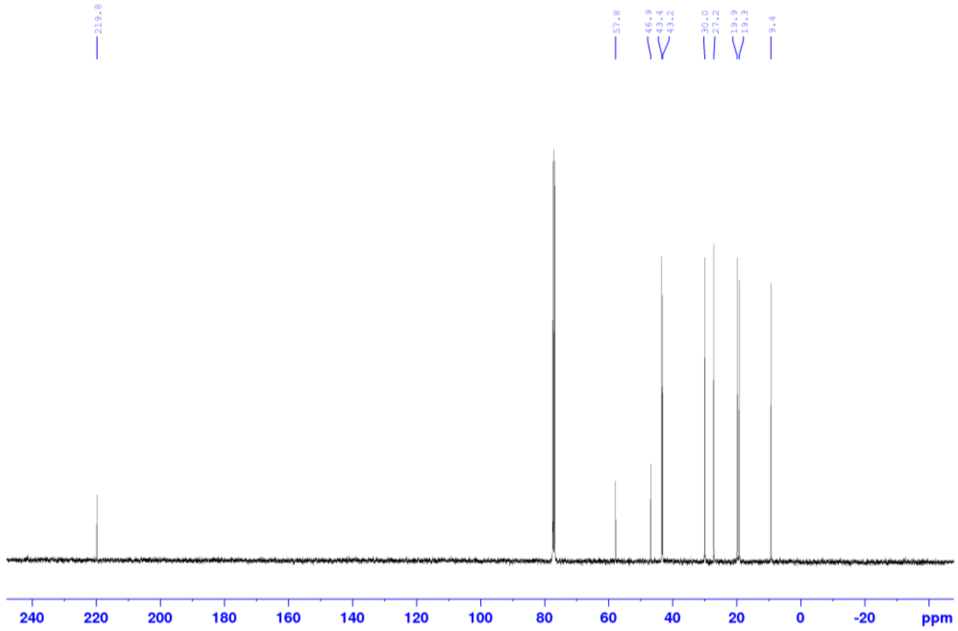

Supplementary Figure 106. ^{13}C NMR spectra of compound **4k**.

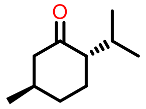

4l

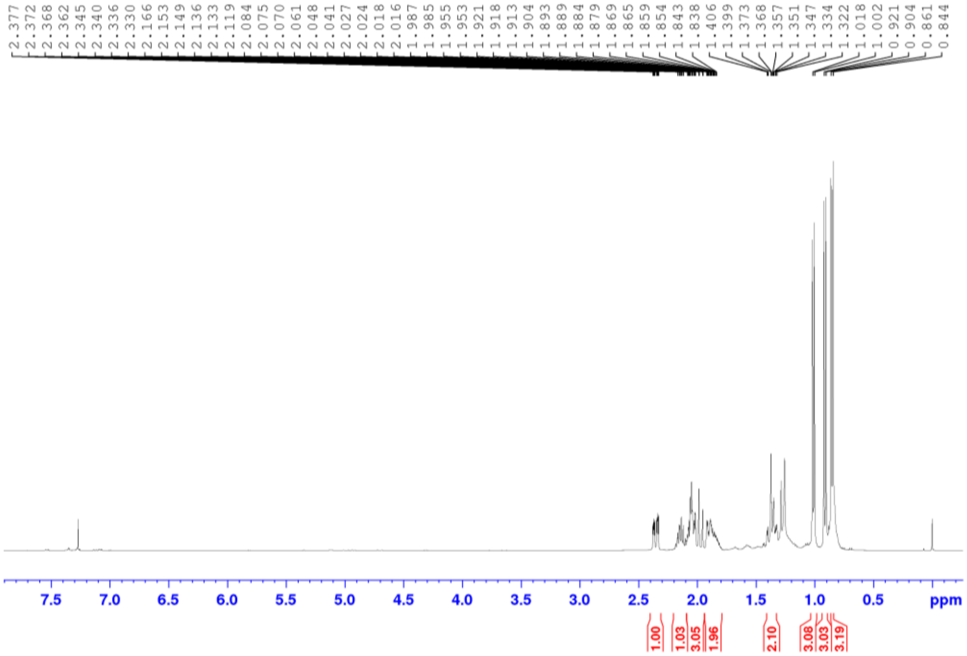

Supplementary Figure 107. ^{1}H NMR spectra of compound **4l**.

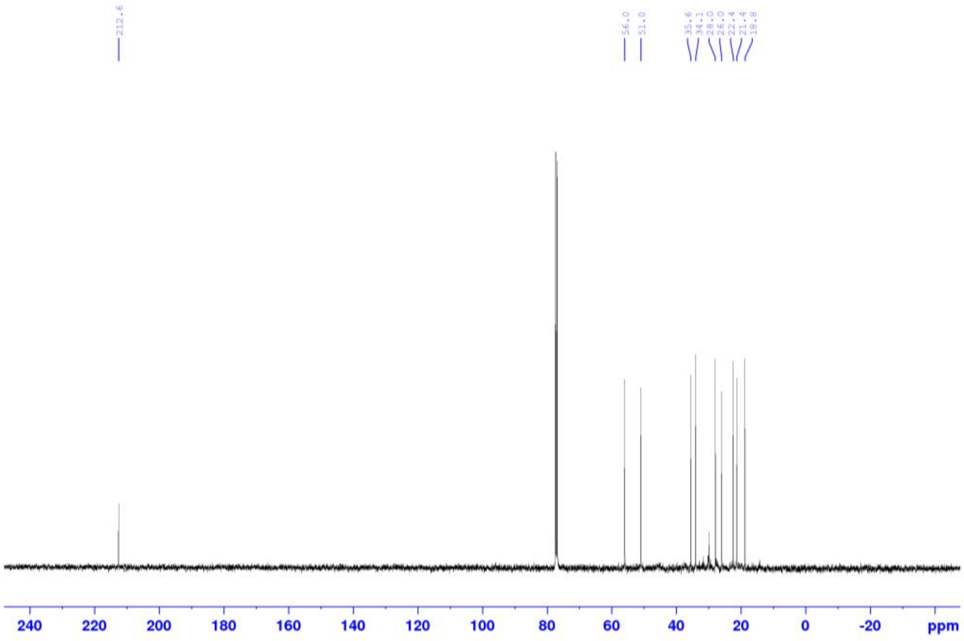

Supplementary Figure 108. ^{13}C NMR spectra of compound **4l**.

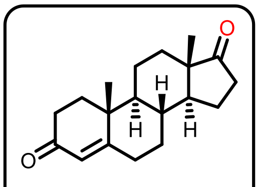

4m

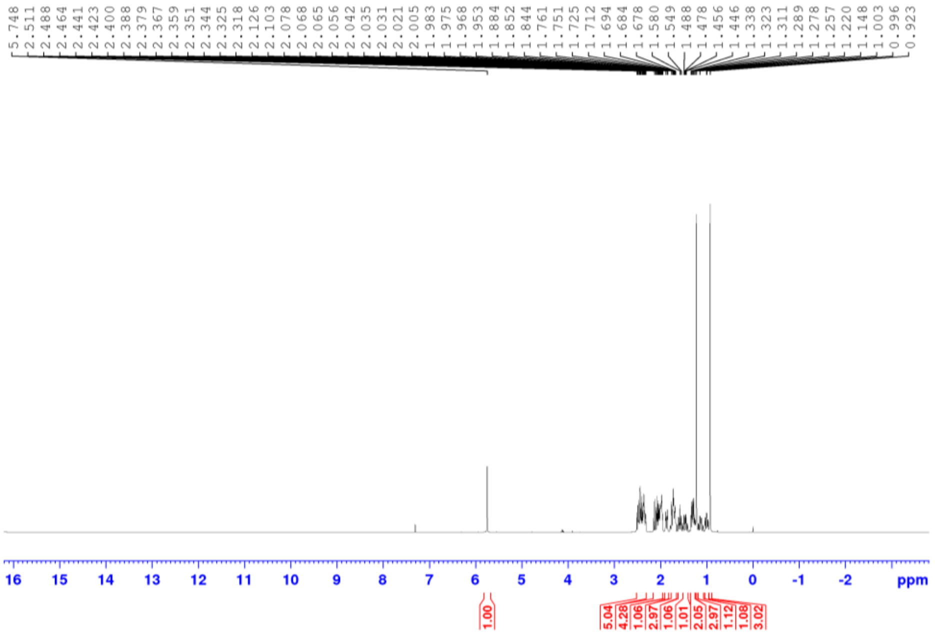

Supplementary Figure 109. ^{1}H NMR spectra of compound **4m**.

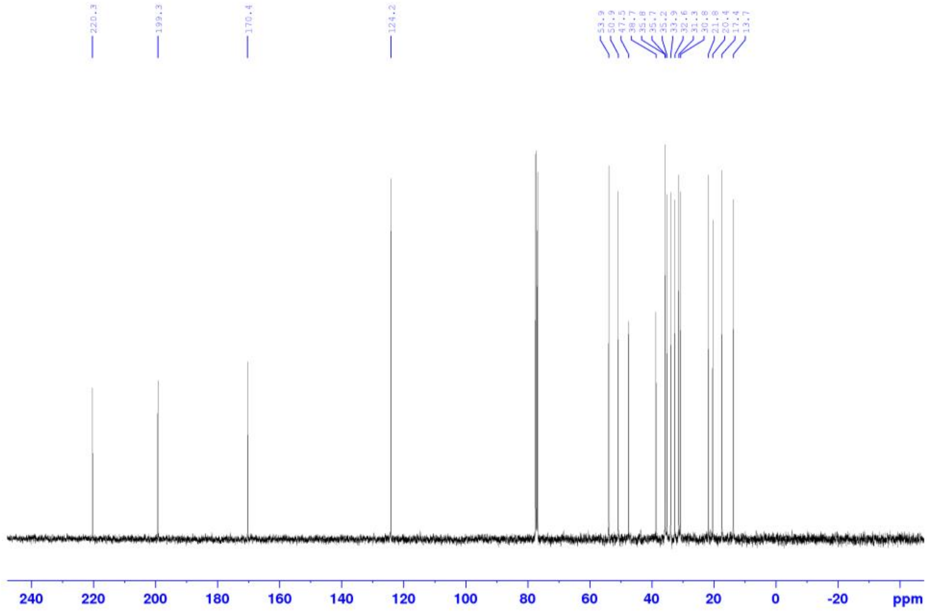

Supplementary Figure 110. ^{13}C NMR spectra of compound **4m**.

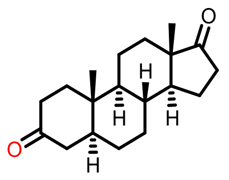

4n

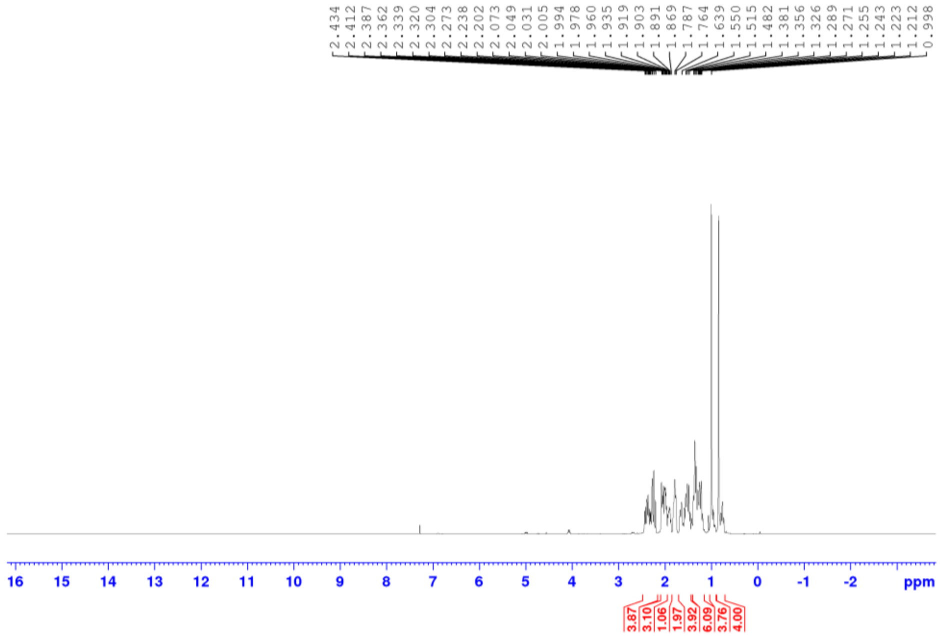

Supplementary Figure 111. ^{1}H NMR spectra of compound **4n**.

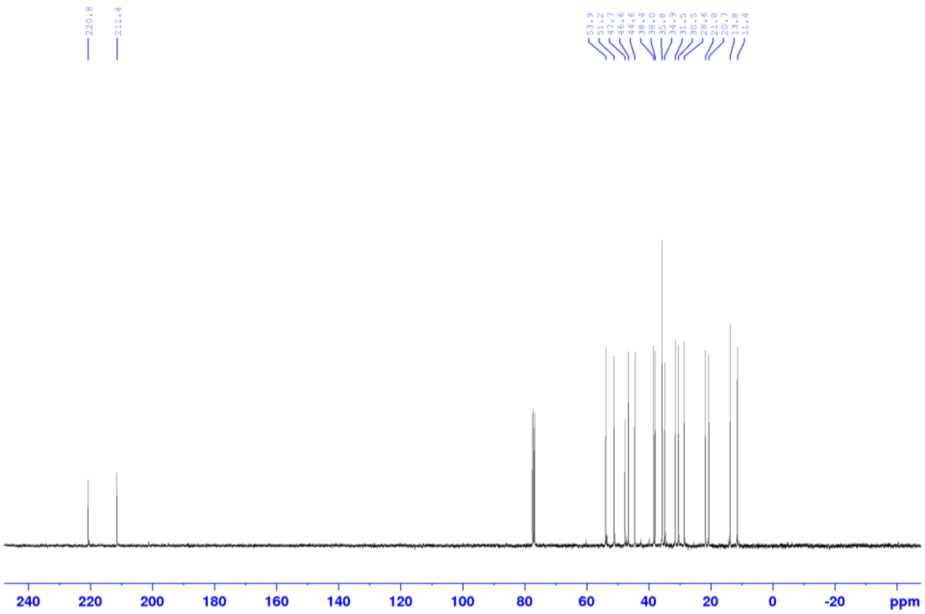

Supplementary Figure 112. ^{13}C NMR spectra of compound **4n**.

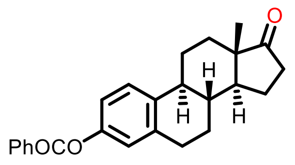

4oa

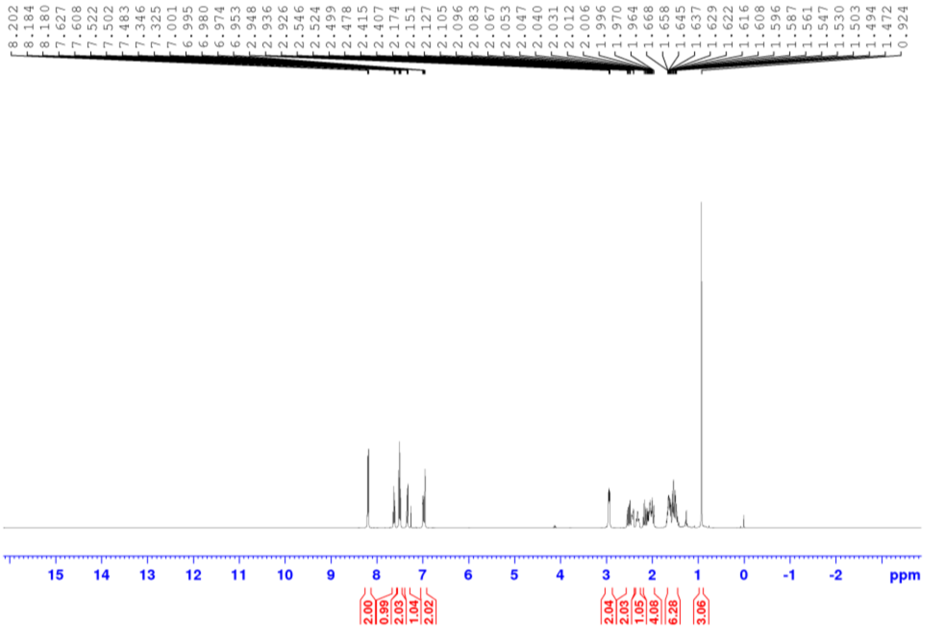

Supplementary Figure 113. ^{1}H NMR spectra of compound **4oa**.

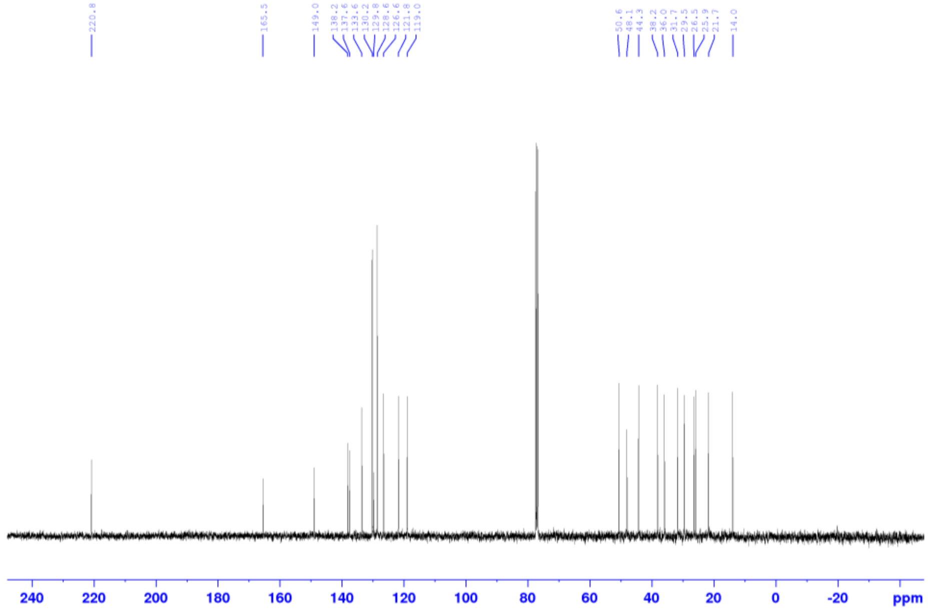

Supplementary Figure 114. ^{13}C NMR spectra of compound **4oa**.

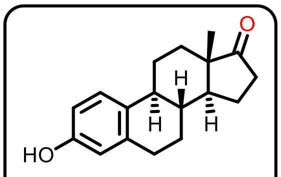

4ob

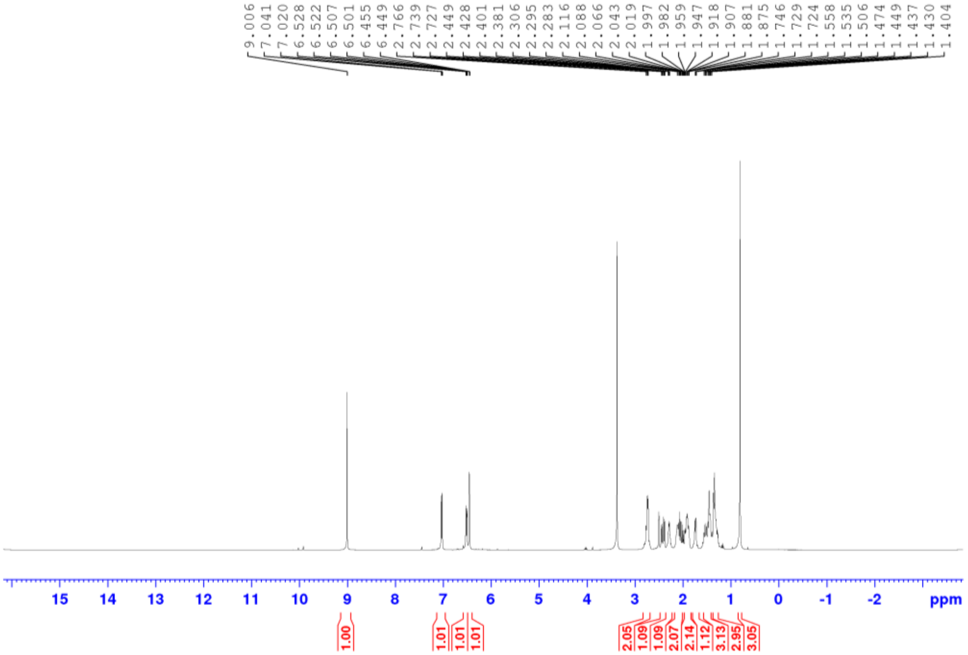

Supplementary Figure 115. ^{1}H NMR spectra of compound **4ob**.

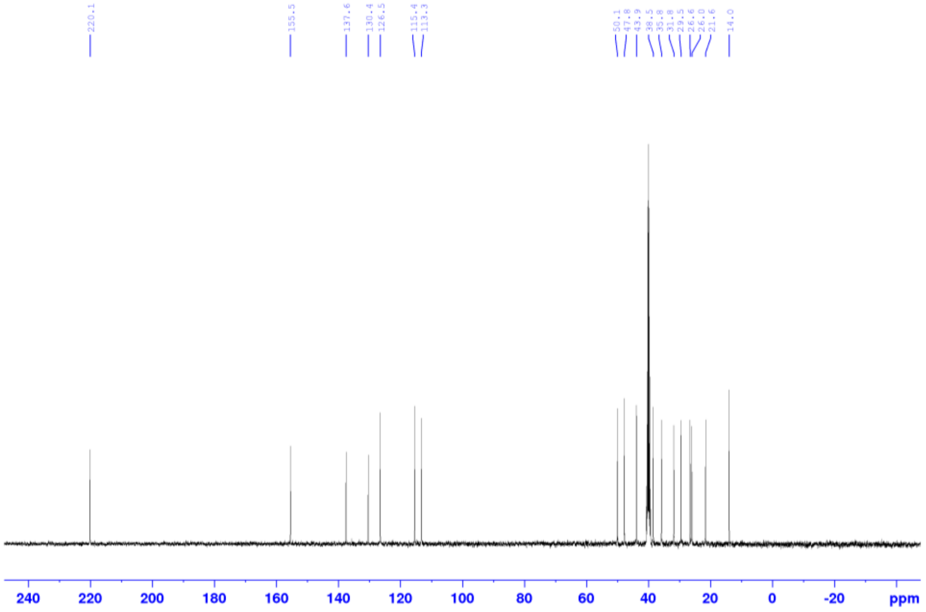

Supplementary Figure 116. ^{13}C NMR spectra of compound **4ob**.

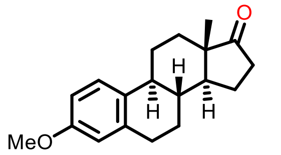

4oc

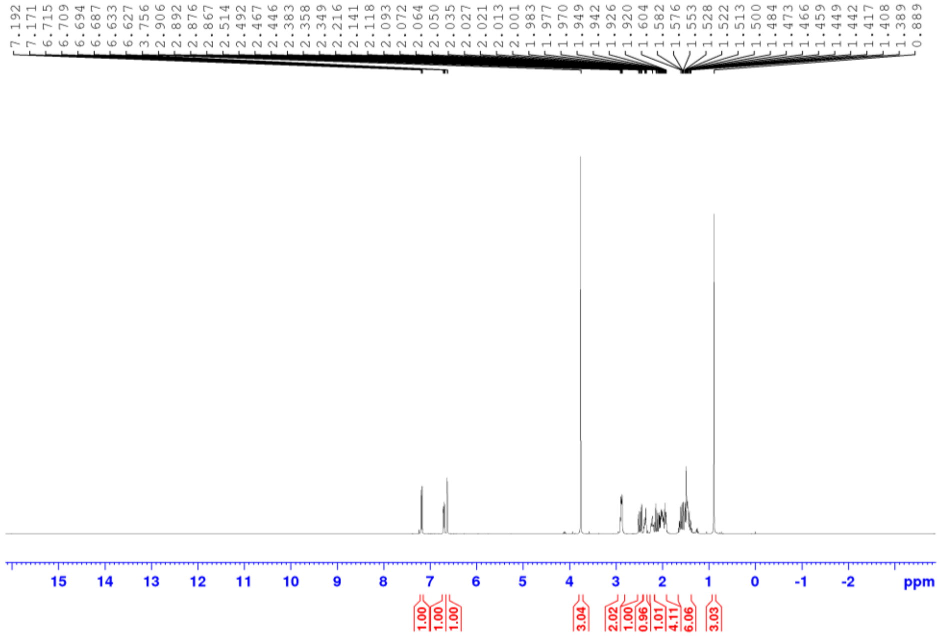

Supplementary Figure 117. ^{1}H NMR spectra of compound **4oc**.

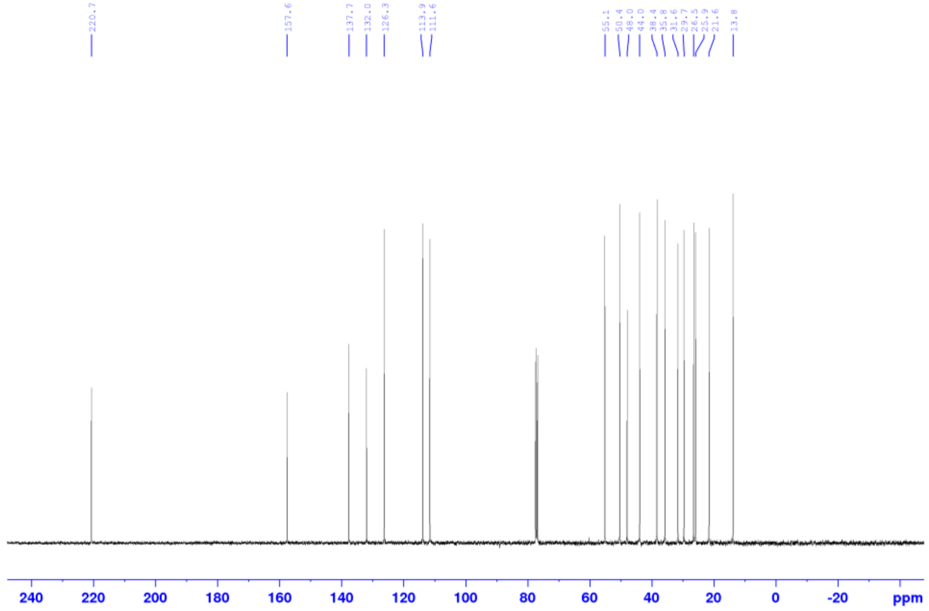

Supplementary Figure 118. ^{13}C NMR spectra of compound **4oc**.

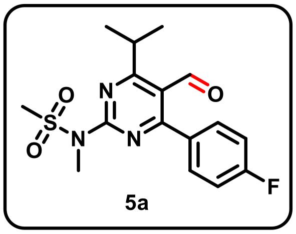

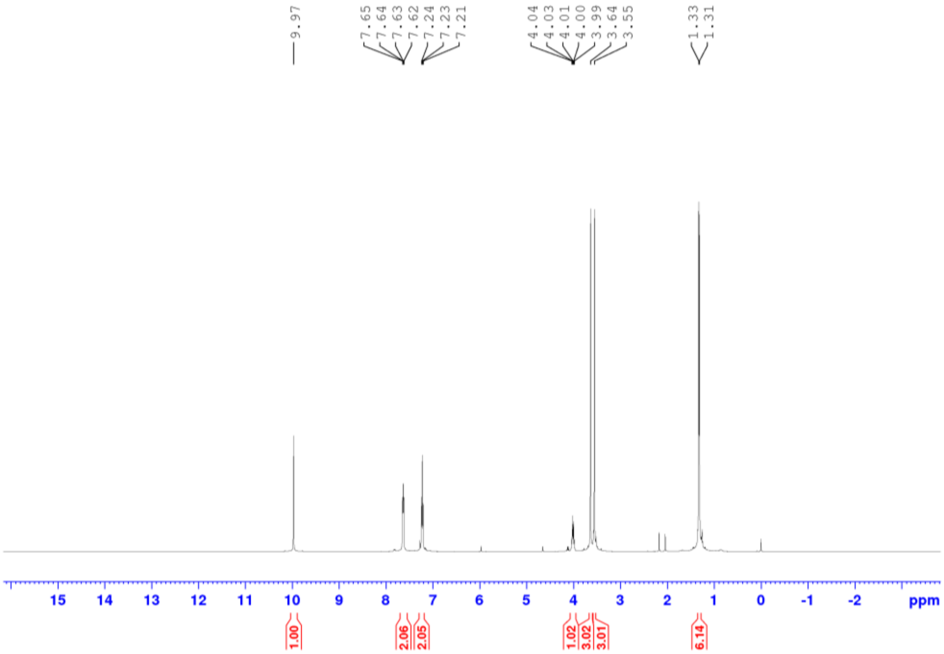

Supplementary Figure 119. ^{1}H NMR spectra of compound **5a**.

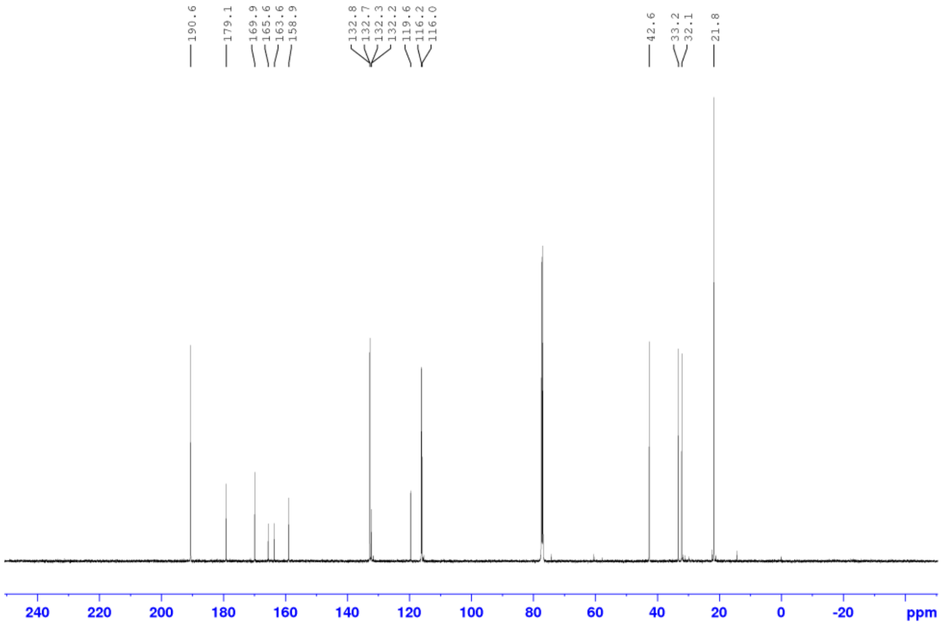

Supplementary Figure 120. ^{13}C NMR spectra of compound **5a**.

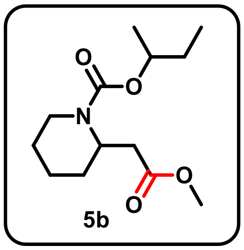

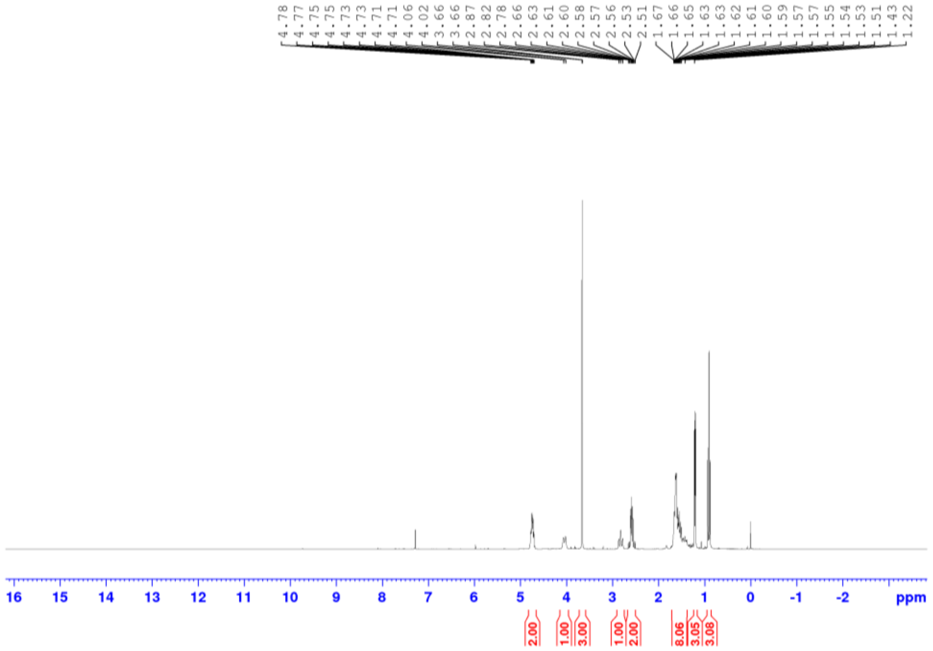

Supplementary Figure 121. ^{1}H NMR spectra of compound **5b**.

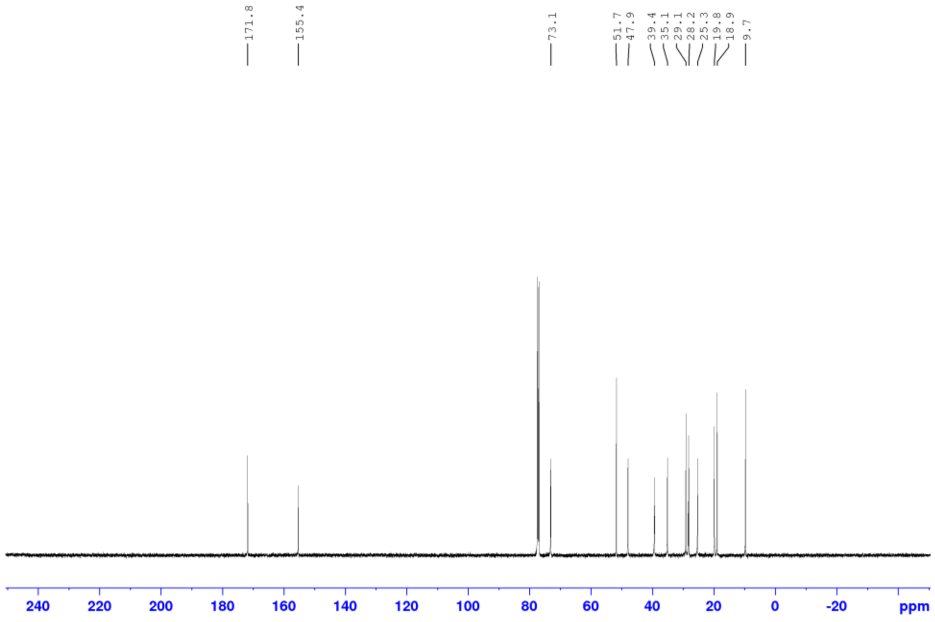

Supplementary Figure 122. ^{13}C NMR spectra of compound **5b**.

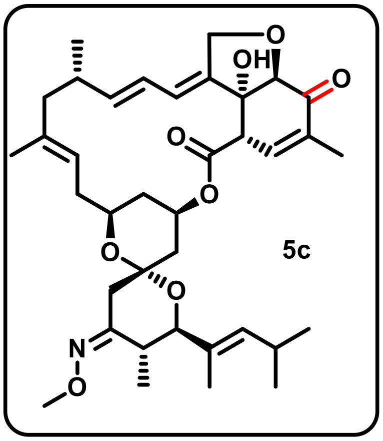

5c

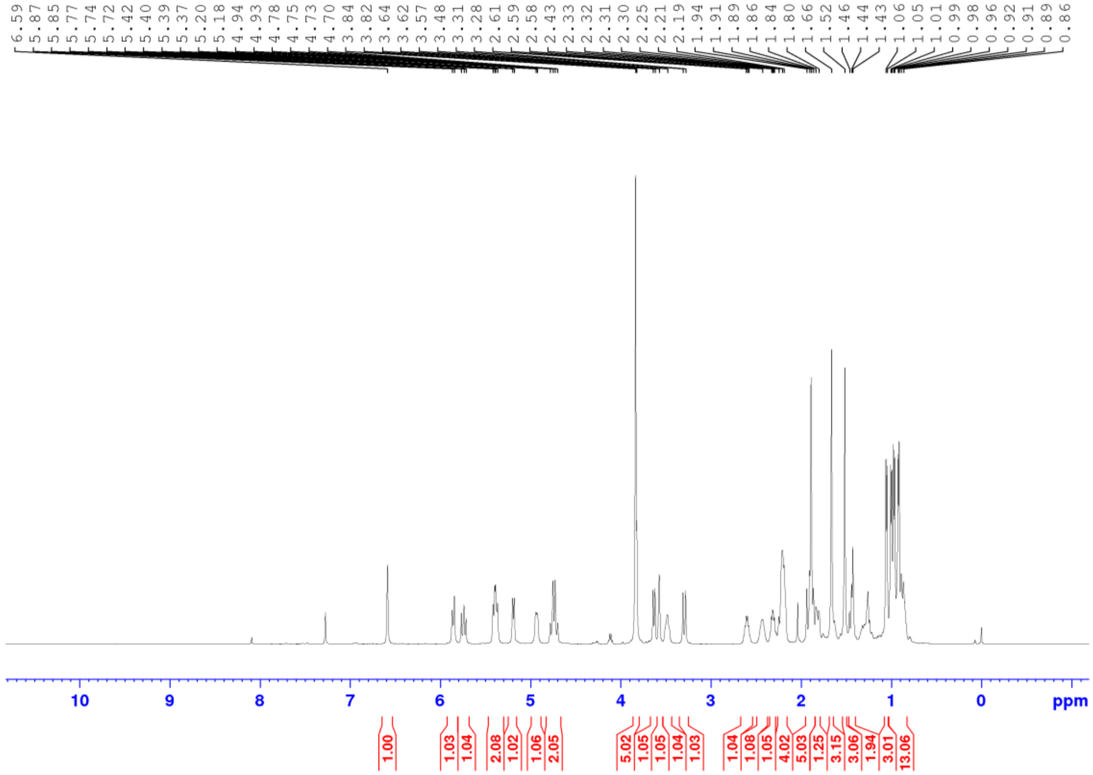

Supplementary Figure 123. ^{1}H NMR spectra of compound **5c**.

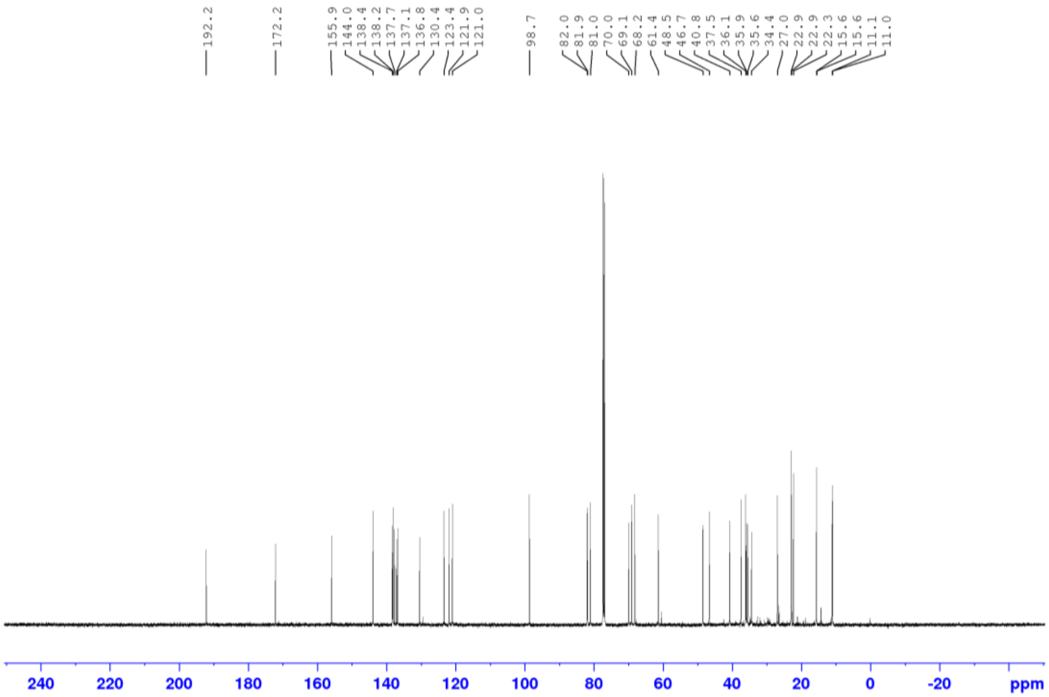

Supplementary Figure 124. ^{13}C NMR spectra of compound **5c**.

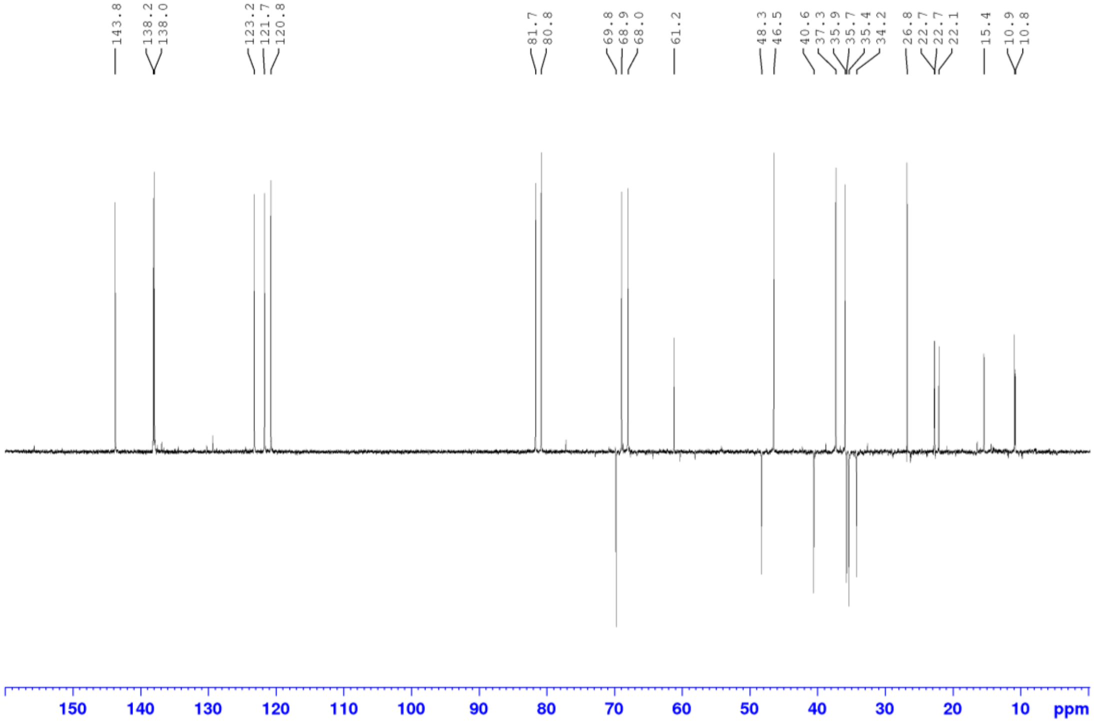

Supplementary Figure 125. DEPT 135 NMR spectra of compound **5c**.

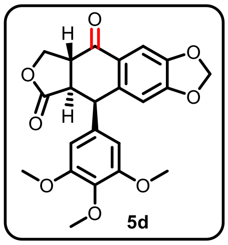

5d

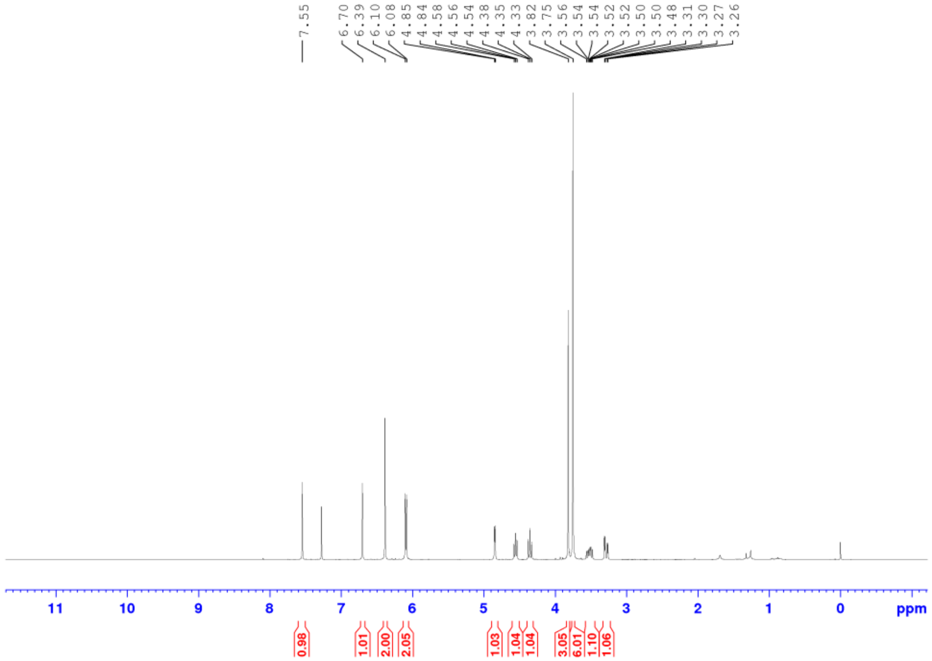

Supplementary Figure 126. ^{1}H NMR spectra of compound **5d**.

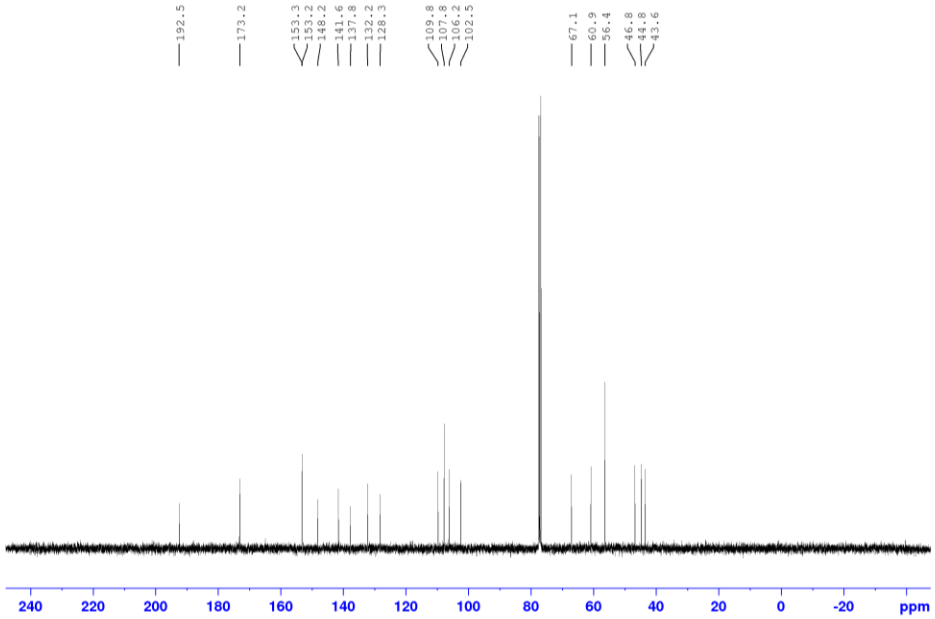

Supplementary Figure 127. ^{13}C NMR spectra of compound **5d**.

4. Supplementary References

1. Qin, Y., Zhang, L., Lv, J., Luo, S. & Cheng, J.-P. Bioinspired organocatalytic aerobic C-H oxidation of amines with an ortho-quinone catalyst. *Org. Lett.*, **17**, 1469-1472 (2015).
2. Tsukakoshi, K. et al. Esterification of PQQ enhances blood-brain barrier permeability and inhibitory activity against amyloidogenic protein fibril formation. *ACS Chem. Neurosci.* **9**, 2898-2903 (2018).
3. Lumpe, H. & Daumann, L. J. Studies of redox cofactor pyrroloquinoline quinone and its interaction with lanthanides(III) and calcium(II). *Inorg. Chem.* **58**, 8432-8441 (2019).
4. Frisch, M. J. et al. Gaussian 09 revision A.02. Gaussian, Inc., Wallingford CT (2009).
5. Lee, C., Yang, W. & Parr, R. G. Development of the Colic-Salvetti correlation-energy formula into a functional of the electron density. *Phys. Rev. B* **37**, 785-789 (1988).
6. Becke, A. D. A new mixing of Hartree–Fock and local density-functional theories. *J. Chem. Phys.* **98**, 1372-1377 (1993).
7. Stephens, P. J., Devlin, F. J., Chabalowski, C. F. & Frisch, M. J. Ab initio calculation of vibrational absorption and circular dichroism spectra using density functional force fields. *J. Phys. Chem.* **98**, 11623-11627 (1994).
8. Schaefer, A. Horn, H. & Ahlrichs, R. Fully optimized contracted Gaussian basis sets for atoms Li to Kr. *J. Chem. Phys.* **97**, 2571-2577 (1992).
9. Cances, E., Mennucci, B. & Tomasi, J. A new integral equation formalism for the polarizable continuum model: Theoretical background and applications to isotropic and anisotropic dielectrics. *J. Chem. Phys.* **107**, 3032-3041 (1997).
10. Tomasi, J., Mennucci, B. & Cances, E. The IEF version of the PCM solvation method: an overview of a new method addressed to study molecular solutes at the QM ab initio level. *J. Mol. Struct. Theochem.* **464**, 211-226 (1999).
11. Tomasi, J. Mennucci, B. & Cammi, R. Quantum mechanical continuum solvation models. *Chem. Rev.* **105**, 2999-3094 (2005).
12. Neese, F. ORCA-an ab initio, DFT and semiempirical SCFMO package, Version 2.6.35. University of Bonn, Germany, (2007).
13. Neese, F. Prediction of electron paramagnetic resonance g values using coupled perturbed Hartree–Fock and Kohn–Sham theory. *J. Chem. Phys.* **115**, 11080-11096 (2001).
14. Neese, F. Efficient and accurate approximations to the molecular spin-orbit coupling operator and their use in molecular *g*-tensor calculations. *J. Chem. Phys.* **122**, 34107 (2005).
15. Bethe, H. & Salpeter, E. *Quantum mechanics of one- and two-electron atoms.* (Springer, Berlin, 1957).
16. Breit, G. The effect of retardation on the interaction of two electrons. *Phys. Rev.* **34**, 553-573 (1929).
17. Stoll, S. & Schweiger, A. EasySpin, a comprehensive software package for spectral simulation and analysis in EPR. *J. Magn. Reson.* **178**, 42-55 (2006).
18. Ge, J.-J. et al. Transition-metal-free deacylative cleavage of unstrained $C(sp^3)$–$C(sp^2)$ bonds: cyanide-free access to aryl and aliphatic nitriles from ketones and aldehydes. *Org. Lett.* **18**, 228-231 (2016).
19. Frisch, M. J.; Trucks, G. W.; Schlegel, H. B.; Scuseria, G. E.; Robb, M. A.; Cheeseman, J. R.; Scalmani, G.; Barone, V.; Mennucci, B.; Petersson, G. A.; Nakatsuji, H.; Caricato, M.; Li, X.; Hratchian, H. P.; Izmaylov, A. F.; Bloino, J.; Zheng, G.; Sonnenberg, J. L.; Hada, M.; Ehara, M.; Toyota, K.; Fukuda, R.; Hasegawa, J.; Ishida, M.; Nakajima, T.; Honda, Y.; Kitao, O.; Nakai, H.; Vreven, T.; Montgomery, J. A.; Peralta, J. E.; Ogliaro, F.; Bearpark, M.; Heyd, J.

J.; Brothers, E.; Kudin, K. N.; Staroverov, V. N.; Keith, T.; Kobayashi, R.; Normand, J.; Raghavachari, K.; Rendell, A.; Burant, J. C.; Iyengar, S. S.; Tomasi, J.; Cossi, M.; Rega, N.; Millam, J. M.; Klene, M.; Knox, J. E.; Cross, J. B.; Bakken, V.; Adamo, C.; Jaramillo, J.; Gomperts, R.; Stratmann, R. E.; Yazyev, O.; Austin, A. J.; Cammi, R.; Pomelli, C.; Ochterski, J. W.; Martin, R. L.; Morokuma, K.; Zakrzewski, V. G.; Voth, G. A.; Salvador, P.; Dannenberg, J. J.; Dapprich, S.; Daniels, A. D.; Farkas, O.; Foresman, J. B.; Ortiz, J. V.; Cioslowski, J.; and Fox, D. J. Gaussian 09, Revision D.01, Gaussian, Inc., Wallingford CT, **2013**.
20. CYLview, 1.0b; Legault, C. Y. Université de Sherbrooke, **2009** (http://www.cylview.org)
21. Lu, T. & Chen, F. Multiwfn: A multifunctional wavefunction analyzer. *J. Comput. Chem.* **33**, 580-592 (2012).
22. Zhao, Y. & Truhlar. D. The M06 suite of density functionals for main group thermochemistry, thermochemical kinetics, noncovalent interactions, excited states, and transition elements: two new functionals and systematic testing of four M06-class functionals and 12 other functionals *Theor. Chem. Acc.* **120**, 215-241 (2008).
23. Marenich, A. V. Cramer, C. J. & Truhlar, D. G. Universal solvation model based on solute electron density and on a continuum model of the solvent defined by the bulk dielectric constant and atomic surface tensions. *J. Phys. Chem. B.* **113**, 6378-6396 (2009).
24. Eichkorn, K. Weigend, F. Truetler, O. & Ahlrichs, R. Auxiliary basis sets for main row atoms and transition metals and their use to approximate Coulomb potentials. *Theor. Chem. Acc.* **97**, 119-124 (1997).
25. Weigend, F. & Ahlrichs, R. Balanced basis sets of split valence, triple zeta valence and quadruple zeta valence quality for H to Rn: Design and assessment of accuracy. *Phys. Chem. Chem. Phys*. 7, 3297-3305 (2005).
26. Weigend, F. Accurate Coulomb-fitting basis sets for H to Rn. *Phys. Chem. Chem. Phys*. **8**, 1057-1065 (2006).
27. Marcus, R. A. On the theory of oxidation‐reduction reactions involving electron transfer. I. *J. Chem. Phys*. **24**, 966-978 (1956).
28. Marcus, R. A. Detection of free radical absorption spectra by chemical modulation. *J. Chem. Phys*. **24**, 962-972 (1956).
29. Marcus, R. A. On the theory of oxidation‐reduction reactions involving electron transfer. III. Applications to data on the rates of organic redox reactions. *J. Chem. Phys.* **26**, 872-877 (1957).
30. Hush, N. S. Adiabatic rate processes at electrodes. I. Energy‐Charge relationships. *J. Chem. Phys*. **28**, 962-972 (1958).
31. Marcus, R. A. On the theory of electrochemical and chemical electron transfer processes. *Can. J. Chem*. **37**, 155-163 (1959).
32. Hush, N. S. Adiabatic theory of outer sphere electron-transfer reactions in solution. *Trans. Faraday Soc*. **57**, 557-580 (1961).
33. Marcus, R. A. The second R. A. Robinson memorial lecture. Electron, proton and related transfers. *Faraday Discuss. Chem. Soc*. **74**, 7-15 (1982).
34. Marcus, R. A. & Sutin, N. Directing charge transfer in perylene based light-harvesting antenna molecules. *Biochim. Biophys. Acta, Rev. Bioenerg*. **811**, 265-322(1985).
35. Costentin, C. Robert, M. & Savéant, J.-M. Electrochemical concerted proton and electron transfers. Potential-dependent rate constant, reorganization factors, proton tunneling and isotope effects. *J. Electroanal. Chem.* **2**, 197-206 (2006).

36. Costentin, C. Robert, M. Savéant, J.-M. & Tard, C. H-bond relays in proton-coupled electron transfers. Oxidation of a phenol concerted with proton transport to a distal base through an OH relay. *Phys. Chem. Chem. Phys.* **13**, 5353-5358 (2011).

37. Romanenko, G. V.; Fokin, S. V.; Letyagin, G. A.; Bogomyakov, A. S. & Ovcharenko, V. I. Structure and magnetic properties of lanthanide compounds with the 3,6-di(tert-butyl)-1,2-benzoquinone radical anion. *J. Struct. Chem.* **60**, 1139-1148 (2019).

38. Methanol and ethanol are also active substrates for the current catalysis. In the oxidation of aliphatic alcohols (methanol used as solvent), we could observe the signal of methyl formate in the NMR spectroscopy of the crude reaction mixture (Supplementary Figure 12&13).

39. Hoover, J. M. & Stahl, S. S. Highly practical copper(I)/TEMPO catalyst system for chemoselective aerobic oxidation of primary alcohols. *J. Am. Chem. Soc.* **133**, 16901-16910 (2011).

40. Feng, Q. & Song, Q. Aldehydes and ketones formation: copper-catalyzed aerobic oxidative decarboxylation of phenylacetic acids and alpha-hydroxyphenylacetic acids. *J. Org. Chem.* **79**, 1867-1871 (2014).

41. Jiang, M., Yang, H. & Fu, H. Visible-light photoredox borylation of aryl halides and subsequent aerobic oxidative hydroxylation. *Org. Lett.* **18**, 5248-5251 (2016).

42. Steves, J. E. & Stahl, S. S. Copper(I)/ABNO-catalyzed aerobic alcohol oxidation: alleviating steric and electronic constraints of Cu/TEMPO catalyst systems. *J. Am. Chem. Soc.* **135**, 15742-15745 (2013).

43. Vinogradova, E. V., Park, N. H., Fors, B. P. & Buchwald, S. L. Palladium-catalyzed synthesis of N-aryl carbamates. *Org. Lett.* **15**, 1394-1397 (2013).

44. Yang, F. & Ackermann, L. Ruthenium-catalyzed C-H oxygenation on aryl Weinreb amides. *Org. Lett.* **15**, 718-720 (2013).

45. Li, Y., Jardine, K. J., Tan, R., Song, D. & Dong, V. M. Palladium-catalyzed intramolecular carboesterification of olefins. *Angew. Chem. Int. Ed.* **48**, 9690-9692 (2009).

46. Liu, J. & Ma, S. Iron-catalyzed aerobic oxidation of allylic alcohols: the issue of horizontal line bond isomerization. *Org. Lett.* **15**, 5150-5153 (2013).

47. Zhang, X.-L. et al. Dehydrogenative *β*-arylation of saturated aldehydes using transient directing groups. *Org. Lett.* **21**, 2731-2735 (2019).

48. Dolui, P., Hazra, S., Deb, M. & Elias, A. J. Picolinamide assisted oxidation of CH_2 groups bound to organic and organometallic compounds using ferrocene as a catalyst. *Organometallics* **38**, 2015-2021 (2019).

49. Whittaker, A. M. & Dong, V. M. Nickel-catalyzed dehydrogenative cross-coupling: direct transformation of aldehydes into esters and amides. *Angew. Chem. Int. Ed.* **54**, 1312-1315 (2015).

50. Powell, A. B. & Stahl, S. S. Aerobic oxidation of diverse primary alcohols to methyl esters with a readily accessible heterogeneous Pd/Bi/Te catalyst. *Org. Lett.* **15**, 5072-5075 (2013).

51. Phelan, J. P. et al. Redox-neutral photocatalytic cyclopropanation via radical/polar crossover. *J. Am. Chem. Soc.* **140**, 8037-8047 (2018).

52. Hama, T. & Hartwig, J. F. Palladium-catalyzed α-arylation of esters with chloroarenes. *Org. Lett.* **10**, 1549-1552 (2008).

53. Chen, Y., Su, L. & Gong, H. Copper-catalyzed and Indium-mediated methoxycarbonylation of unactivated alkyl iodides with balloon CO. *Org. Lett.* **21**, 4689-4693 (2019).

54. Ma, X. & Herzon, S. B. Synthesis of ketones and esters from heteroatom-functionalized alkenes by cobalt-mediated hydrogen atom transfer. *J. Org. Chem.* **81**, 8673-8695 (2016).

55. Xie, X. & Stahl, S. S. Efficient and selective Cu/nitroxyl-catalyzed methods for aerobic oxidative lactonization of diols. *J. Am. Chem. Soc.* **137**, 3767-3770 (2015).
56. Tan, X. et al. Silver-catalyzed decarboxylative bromination of aliphatic carboxylic acids. *Org. Lett.* **19**, 1634-1637 (2017).
57. Wang, X., Liu, R., Jin, Y. & Liang, X. TEMPO/HCl/$NaNO_2$ catalyst: a transition-metal-free approach to efficient aerobic oxidation of alcohols to aldehydes and ketones under mild conditions. *Chem.-Eur. J.* **14**, 2679-2685 (2008).
58. Moriyama, K., Takemura, M. & Togo, H. Direct and selective benzylic oxidation of alkylarenes via C-H abstraction using alkali metal bromides. *Org. Lett.* **14**, 2414-2417 (2012).
59. Zheng, M., Wu, F., Chen, K. & Zhu, S. Styrene as 4π-component in Zn(II)-catalyzed intermolecular diels-alder/ene tandem reaction. *Org. Lett.* **18**, 3554-3557 (2016).
60. Kamijo, S., Tao, K., Takao, G., Tonoda, H. & Murafuji, T. Photoinduced oxidation of secondary alcohols using 4-benzoylpyridine as an oxidant. *Org. Lett.* **17**, 3326-3329 (2015).
61. Yu, H. et al. Iron-catalyzed oxidative functionalization of C(sp^3)-H bonds under bromide-synergized mild conditions. *Chem. Commun.* **55**, 7840-7843 (2019)/
62. Hu, Q., Lin, G. S., Duan, W. G., Huang, M. & Lei, F. H. Synthesis and biological activity of novel (Z)- and (E)-verbenone oxime esters. *Molecules* **22**, 1678 (2017).
63. Gandomkar, S. et al. Biocatalytic enantioselective oxidation of sec-allylic alcohols with flavin-dependent oxidases. *Adv. Synth. Catal.* **361**, 5264-5271 (2019).
64. Ogura, K., Yamashita, M., Suzuki, M., Furukawa, S. & Tsuchihashi, G.-I. A versatile synthesis of four-, five-, and six-membered cyclic ketones using methyl methylthiomethyl sulfoxide. *Bull. Chem. Soc. Jpn.* **57**, 1637-1642 (1984).
65. Shipilovskikh, S. A., Rubtsov, A. E. & Malkov, A. V. Oxidative dehomologation of aldehydes with oxygen as a terminal oxidant. *Org. Lett.* **19**, 6760-6762 (2017).
66. Gilissen, P. J., Blanco-Ania, D. & Rutjes, F. Oxidation of secondary methyl ethers to ketones. *J. Org. Chem.* **82**, 6671-6679 (2017).
67. Huang, Q. et al. Regioselective wacker-type oxidation of internal olefins in (t)BuOH using oxygen as the sole oxidant and (t)BuONO as the organic redox cocatalyst. *Org. Lett.* **22**, 965-969 (2020).
68. Fryszkowska, A. et al. Development of a chemoenzymatic process for dehydroepiandrosterone acetate synthesis. *Org. Process Res. Dev.* **20**, 1520-1528 (2016).
69. Zhang, W., Carpenter, K. L. & Lin, S. Electrochemistry broadens the scope of flavin photocatalysis: photoelectrocatalytic oxidation of unactivated alcohols. *Angew. Chem. Int. Ed.* **59**, 409-417 (2020).
70. Li, G., Lei, P. & Szostak, M. Transition-metal-free esterification of amides via selective N-C cleavage under mild conditions. *Org. Lett.* **20**, 5622-5625 (2018).
71. Foucher, V., Guizzardi, B., Groen, M. B., Light, M. & Linclau, B. A novel, versatile D-->BCD steroid construction strategy, illustrated by the enantioselective total synthesis of estrone. *Org. Lett.* **12**, 680-683 (2010).
72. Steves, J. E. et al. Process Development of CuI/ABNO/NMI-Catalyzed Aerobic Alcohol Oxidation. *Org. Process Res. Dev.* **19**, 1548-1553 (2015).
73. Xiao, J., Cong, X.-W., Yang, G.-Z., Wang, Y.-W. & Peng, Y. Divergent asymmetric syntheses of podophyllotoxin and related family members via stereoselective reductive Ni-catalysis. *Org. Lett.* **20**, 1651-1654 (2018).
