# Supplementary material for: Bio-inspired lanthanum-ortho-quinone catalysis for aerobic alcohol oxidation: semi-quinone anionic radical as redox ligand
Source: Nat Commun. 2022 Jan 20;13:428. doi: 10.1038/s41467-022-28102-4 (PMC8776754; doi:10.1038/s41467-022-28102-4)
Supplement: Supplementary file 2 — Source Data [file 41467_2022_28102_MOESM2_ESM.docx]

Inventory of Supplementary Information

[Table of Contents 1](#_Toc90468032)

[1.Supplementary Notes 2](#_Toc90468033)

[Supplementary Table 1. Screening of Metal 4](#_Toc90468034)

[Supplementary Table 2. Screening of Lanthanum Catalysts 5](#_Toc90468035)

[Supplementary Table 3. Screening of Additive 5](#_Toc90468036)

[Supplementary Table 4. Screening of Iodine Source 6](#_Toc90468037)

[Supplementary Table 5. Screening of Solvent 6](#_Toc90468038)

[Supplementary Table 6. Screening of Acid and Base 7](#_Toc90468039)

[Supplementary Figure 1. Screening of different quinone catalysts. 8](#_Toc90468040)

[Supplementary Table 7. Control Experiments. 9](#_Toc90468041)

[Supplementary Figure 2. EPR Simulation of Semiquinone-La Complex. 11](#_Toc90468042)

[Supplementary Figure 3. The shift of reductive potential of *o*-Q by adding various rare earth elements. 13](#_Toc90468043)

[Supplementary Table 8. Reduction potential of *o*-Q by adding various rare earth salts 13](#_Toc90468044)

[Supplementary Figure 4. UV-Vis spectrum. 14](#_Toc90468045)

[Supplementary Figure 5. Reaction monitor by UV-Vis spectrum. . 15](#_Toc90468046)

[Supplementary Figure 6. UV-Vis spectrum of reoxidation of catechol *o*-Q_red_ under basic condition. 16](#_Toc90468047)

[Supplementary Figure 7. Time course of reoxidation of catechol *o*-Q_red_ with lanthanum under basic condition. 17](#_Toc90468048)

[Supplementary Figure 8. Time course of reoxidation of catechol *o*-Q_red_ with lanthanum under basic and anaerobic condition. 18](#_Toc90468049)

[Supplementary Figure 9. Time course of reoxidation of catechol *o*-Q_red_ without lanthanum under basic condition. 19](#_Toc90468050)

[Supplementary Figure 10. ^1^H NMR of PhCD_2_OH in CDCl_3_. 20](#_Toc90468051)

[Supplementary Figure 11. ^1^H NMR of BnOD in CD_3_CN. 21](#_Toc90468052)

[Supplementary Table 9. Kinetic data for KIE experiments. 21](#_Toc90468053)

[Supplementary Table 10. Kinetic data for Hammett plot. 22](#_Toc90468054)

[Supplementary Figure 12. NMR spectroscopy of the crude reaction mixture of aliphatic alcohol oxidation. 23](#_Toc90468055)

[Supplementary Figure 13. NMR spectroscopy of the crude reaction mixture of ethanol oxidation. 23](#_Toc90468056)

[Supplementary Figure 14 Visual Kinetic analysis^a^ 24](#_Toc90468057)

[Supplementary Figure 15. The spin density of the [La(SQ)_2_]^+^ complex (Int-1). 26](#_Toc90468058)

[Supplementary Figure 16. The reaction profile for the proposed dehydrogenation process. 28](#_Toc90468059)

[3.Supplementary Methods 41](#_Toc90468060)

[Supplementary Figure 17. ^1^H NMR spectra of compound 2a. 58](#_Toc90468061)

[Supplementary Figure 18. ^13^C NMR spectra of compound 2a. 58](#_Toc90468062)

[Supplementary Figure 19. ^1^H NMR spectra of compound 2b. 59](#_Toc90468063)

[Supplementary Figure 20. ^13^C NMR spectra of compound 2b. 59](#_Toc90468064)

[Supplementary Figure 21. ^1^H NMR spectra of compound 2c. 60](#_Toc90468065)

[Supplementary Figure 22. ^13^C NMR spectra of compound 2c. 60](#_Toc90468066)

[Supplementary Figure 23. ^1^H NMR spectra of compound 2d. 61](#_Toc90468067)

[Supplementary Figure 24. ^13^C NMR spectra of compound 2d. 61](#_Toc90468068)

[Supplementary Figure 25. ^1^H NMR spectra of compound 2e. 62](#_Toc90468069)

[Supplementary Figure 26. ^13^C NMR spectra of compound 2e. 62](#_Toc90468070)

[Supplementary Figure 27. ^1^H NMR spectra of compound 2f. 63](#_Toc90468071)

[Supplementary Figure 28. ^13^C NMR spectra of compound 2f. 63](#_Toc90468072)

[Supplementary Figure 29. ^1^H NMR spectra of compound 2g. 64](#_Toc90468073)

[Supplementary Figure 30. ^13^C NMR spectra of compound 2g. 64](#_Toc90468074)

[Supplementary Figure 31. ^1^H NMR spectra of compound 2h. 65](#_Toc90468075)

[Supplementary Figure 32. ^13^C NMR spectra of compound 2h. 65](#_Toc90468076)

[Supplementary Figure 33. ^1^H NMR spectra of compound 2i. 66](#_Toc90468077)

[Supplementary Figure 34. ^13^C NMR spectra of compound 2i. 66](#_Toc90468078)

[Supplementary Figure 35. ^1^H NMR spectra of compound 2j. 67](#_Toc90468079)

[Supplementary Figure 36. ^13^C NMR spectra of compound 2j. 67](#_Toc90468080)

[Supplementary Figure 37. ^1^H NMR spectra of compound 2k. 68](#_Toc90468081)

[Supplementary Figure 38. ^13^C NMR spectra of compound 2k. 68](#_Toc90468082)

[Supplementary Figure 39. ^1^H NMR spectra of compound 2l. 69](#_Toc90468083)

[Supplementary Figure 40. ^13^C NMR spectra of compound 2l. 69](#_Toc90468084)

[Supplementary Figure 41. ^1^H NMR spectra of compound 2m. 70](#_Toc90468085)

[Supplementary Figure 42. ^13^C NMR spectra of compound 2m. 70](#_Toc90468086)

[Supplementary Figure 43. ^1^H NMR spectra of compound 2n. 71](#_Toc90468087)

[Supplementary Figure 44. ^13^C NMR spectra of compound 2n. 71](#_Toc90468088)

[Supplementary Figure 45. ^1^H NMR spectra of compound 3a. 72](#_Toc90468089)

[Supplementary Figure 46. ^13^C NMR spectra of compound 3a. 72](#_Toc90468090)

[Supplementary Figure 47. ^1^H NMR spectra of compound 3b. 73](#_Toc90468091)

[Supplementary Figure 48. ^13^C NMR spectra of compound 3b. 73](#_Toc90468092)

[Supplementary Figure 49. ^1^H NMR spectra of compound 3c. 74](#_Toc90468093)

[Supplementary Figure 50. ^13^C NMR spectra of compound 3c. 74](#_Toc90468094)

[Supplementary Figure 51. ^1^H NMR spectra of compound 3d. 75](#_Toc90468095)

[Supplementary Figure 52. ^13^C NMR spectra of compound 3d. 75](#_Toc90468096)

[Supplementary Figure 53. ^1^H NMR spectra of compound 3e. 76](#_Toc90468097)

[Supplementary Figure 54. ^13^C NMR spectra of compound 3e. 76](#_Toc90468098)

[Supplementary Figure 55. ^1^H NMR spectra of compound 3f. 77](#_Toc90468099)

[Supplementary Figure 56. ^13^C NMR spectra of compound 3f. 77](#_Toc90468100)

[Supplementary Figure 57. ^1^H NMR spectra of compound 3g. 78](#_Toc90468101)

[Supplementary Figure 58. ^13^C NMR spectra of compound 3g. 78](#_Toc90468102)

[Supplementary Figure 59. ^1^H NMR spectra of compound 3h. 79](#_Toc90468103)

[Supplementary Figure 60. ^13^C NMR spectra of compound 3h. 79](#_Toc90468104)

[Supplementary Figure 61. ^1^H NMR spectra of compound 3i. 80](#_Toc90468105)

[Supplementary Figure 62. ^13^C NMR spectra of compound 3i. 80](#_Toc90468106)

[Supplementary Figure 63. ^1^H NMR spectra of compound 3j. 81](#_Toc90468107)

[Supplementary Figure 64. ^13^C NMR spectra of compound 3j. 81](#_Toc90468108)

[Supplementary Figure 65. ^1^H NMR spectra of compound 3k. 82](#_Toc90468109)

[Supplementary Figure 66. ^13^C NMR spectra of compound 3k. 82](#_Toc90468110)

[Supplementary Figure 67. ^1^H NMR spectra of compound 4aa. 83](#_Toc90468111)

[Supplementary Figure 68. ^13^C NMR spectra of compound 4aa. 83](#_Toc90468112)

[Supplementary Figure 69. ^1^H NMR spectra of compound 4ab. 84](#_Toc90468113)

[Supplementary Figure 70. ^13^C NMR spectra of compound 4ab. 84](#_Toc90468114)

[Supplementary Figure 71. ^1^H NMR spectra of compound 4ac. 85](#_Toc90468115)

[Supplementary Figure 72. ^13^C NMR spectra of compound 4ac. 85](#_Toc90468116)

[Supplementary Figure 73. ^1^H NMR spectra of compound 4ad. 86](#_Toc90468117)

[Supplementary Figure 74. ^13^C NMR spectra of compound 4ad. 86](#_Toc90468118)

[Supplementary Figure 75. ^1^H NMR spectra of compound 4ae. 87](#_Toc90468119)

[Supplementary Figure 76. ^13^C NMR spectra of compound 4ae. 87](#_Toc90468120)

[Supplementary Figure 77. ^1^H NMR spectra of compound 4af. 88](#_Toc90468121)

[Supplementary Figure 78. ^13^C NMR spectra of compound 4af. 88](#_Toc90468122)

[Supplementary Figure 79. ^1^H NMR spectra of compound 4b. 89](#_Toc90468123)

[Supplementary Figure 80. ^13^C NMR spectra of compound 4b. 89](#_Toc90468124)

[Supplementary Figure 81. ^1^H NMR spectra of compound 4c. 90](#_Toc90468125)

[Supplementary Figure 82. ^13^C NMR spectra of compound 4c. 90](#_Toc90468126)

[Supplementary Figure 83. ^1^H NMR spectra of compound 4d. 91](#_Toc90468127)

[Supplementary Figure 84. ^13^C NMR spectra of compound 4d. 91](#_Toc90468128)

[Supplementary Figure 85. ^1^H NMR spectra of compound 4e. 92](#_Toc90468129)

[Supplementary Figure 86. ^13^C NMR spectra of compound 4e. 92](#_Toc90468130)

[Supplementary Figure 87. ^1^H NMR spectra of compound 4fa. 93](#_Toc90468131)

[Supplementary Figure 88. ^13^C NMR spectra of compound 4fa. 93](#_Toc90468132)

[Supplementary Figure 89. ^1^H NMR spectra of compound 4fb. 94](#_Toc90468133)

[Supplementary Figure 90. ^13^C NMR spectra of compound 4fb. 94](#_Toc90468134)

[Supplementary Figure 91. ^1^H NMR spectra of compound 4fc. 95](#_Toc90468135)

[Supplementary Figure 92. ^13^C NMR spectra of compound 4fc. 95](#_Toc90468136)

[Supplementary Figure 93. ^1^H NMR spectra of compound 4fd. 96](#_Toc90468137)

[Supplementary Figure 94. ^13^C NMR spectra of compound 4fd. 96](#_Toc90468138)

[Supplementary Figure 95. ^1^H NMR spectra of compound 4ga. 97](#_Toc90468139)

[Supplementary Figure 96. ^13^C NMR spectra of compound 4ga. 97](#_Toc90468140)

[Supplementary Figure 97. ^1^H NMR spectra of compound 4gb. 98](#_Toc90468141)

[Supplementary Figure 98. ^13^C NMR spectra of compound 4gb. 98](#_Toc90468142)

[Supplementary Figure 99. ^1^H NMR spectra of compound 4h. 99](#_Toc90468143)

[Supplementary Figure 100. ^13^C NMR spectra of compound 4h. 99](#_Toc90468144)

[Supplementary Figure 101. ^1^H NMR spectra of compound 4i. 100](#_Toc90468145)

[Supplementary Figure 102. ^13^C NMR spectra of compound 4i. 100](#_Toc90468146)

[Supplementary Figure 103. ^1^H NMR spectra of compound 4j. 101](#_Toc90468147)

[Supplementary Figure 104. ^13^C NMR spectra of compound 4j. 101](#_Toc90468148)

[Supplementary Figure 105. ^1^H NMR spectra of compound 4k. 102](#_Toc90468149)

[Supplementary Figure 106. ^13^C NMR spectra of compound 4k. 102](#_Toc90468150)

[Supplementary Figure 107. ^1^H NMR spectra of compound 4l. 103](#_Toc90468151)

[Supplementary Figure 108. ^13^C NMR spectra of compound 4l. 103](#_Toc90468152)

[Supplementary Figure 109. ^1^H NMR spectra of compound 4m. 104](#_Toc90468153)

[Supplementary Figure 110. ^13^C NMR spectra of compound 4m. 104](#_Toc90468154)

[Supplementary Figure 111. ^1^H NMR spectra of compound 4n. 105](#_Toc90468155)

[Supplementary Figure 112. ^13^C NMR spectra of compound 4n. 105](#_Toc90468156)

[Supplementary Figure 113. ^1^H NMR spectra of compound 4oa. 106](#_Toc90468157)

[Supplementary Figure 114. ^13^C NMR spectra of compound 4oa. 106](#_Toc90468158)

[Supplementary Figure 115. ^1^H NMR spectra of compound 4ob. 107](#_Toc90468159)

[Supplementary Figure 116. ^13^C NMR spectra of compound 4ob. 107](#_Toc90468160)

[Supplementary Figure 117. ^1^H NMR spectra of compound 4oc. 108](#_Toc90468161)

[Supplementary Figure 118. ^13^C NMR spectra of compound 4oc. 108](#_Toc90468162)

[Supplementary Figure 119. ^1^H NMR spectra of compound 5a. 109](#_Toc90468163)

[Supplementary Figure 120. ^13^C NMR spectra of compound 5a. 109](#_Toc90468164)

[Supplementary Figure 121. ^1^H NMR spectra of compound 5b. 110](#_Toc90468165)

[Supplementary Figure 122. ^13^C NMR spectra of compound 5b. 110](#_Toc90468166)

[Supplementary Figure 123. ^1^H NMR spectra of compound 5c. 111](#_Toc90468167)

[Supplementary Figure 124. ^13^C NMR spectra of compound 5c. 112](#_Toc90468168)

[Supplementary Figure 125. DEPT 135 NMR spectra of compound 5c. 112](#_Toc90468169)

[Supplementary Figure 126. ^1^H NMR spectra of compound 5d. 113](#_Toc90468170)

[Supplementary Figure 127. ^13^C NMR spectra of compound 5d. 113](#_Toc90468171)

[4.Supplementary References 114](#_Toc90468172)
